# Safety of Short-Term Treatments with Oral Chloroquine and Hydroxychloroquine in Patients with and without COVID-19: A Systematic Review

**DOI:** 10.3390/ph15050634

**Published:** 2022-05-21

**Authors:** Sergio Marin, Alba Martin Val, Maite Bosch Peligero, Cristina Rodríguez-Bernuz, Ariadna Pérez-Ricart, Laia Vilaró Jaques, Roger Paredes, Josep Roca, Carles Quiñones

**Affiliations:** 1 Pharmacy Department, Hospital Universitari Germans Trias i Pujol, 08916 Badalona, Spain; amartinv.germanstrias@gencat.cat (A.M.V.); mtbosch.germanstrias@gencat.cat (M.B.P.); crodriguezb.germanstrias@gencat.cat (C.R.-B.); ariadna.perezr@catsalut.cat (A.P.-R.); laiavil88@gmail.com (L.V.J.); cquinonesr.germanstrias@gencat.cat (C.Q.); 2 Department of Pharmacology, Toxicology and Therapeutic Chemistry, University of Barcelona, 08034 Barcelona, Spain; 3 Catalan Health System, North Barcelona Metropolitan Area, Pharmacy Department, 08172 Sant Cugat del Vallès, Spain; 4 IrsiCaixa AIDS Institute, Hospital Universitari Germans Trias i Pujol, 08916 Badalona, Spain; rparedes@irsicaixa.es; 5 Infectious Diseases Department, Hospital Universitari Germans Trias i Pujol, 08916 Badalona, Spain; 6 Epidemiology Unit, Hospital Universitari Germans Trias i Pujol, 08916 Badalona, Spain; pep.roca@gmail.com; 7 Clinical Pharmacy and Pharmacotherapy Unit, Department of Pharmacy, Pharmaceutical Technology and Physical Chemistry, University of Barcelona, 08034 Barcelona, Spain

**Keywords:** chloroquine, hydroxychloroquine, adverse reactions, drug safety, systematic review

## Abstract

Chloroquine (CQ) and hydroxychloroquine (HCQ) have recently become the focus of global attention as possible treatments for Coronavirus Disease 2019 (COVID-19). The current systematic review aims to assess their safety in short treatments (≤14 days), whether used alone or in combination with other drugs. Following the PRISMA and SWiM recommendations, a search was conducted using four health databases for all relevant English-, Chinese-, and Spanish-language studies from inception through 30 July 2021. Patients treated for any condition and with any comparator were included. The outcomes of interest were early drug adverse effects and their frequency. A total of 254 articles met the inclusion criteria, including case and case-control reports as well as cross-sectional, cohort, and randomised studies. The results were summarised either qualitatively in table or narrative form or, when possible (99 studies), quantitatively in terms of adverse event frequencies. Quality evaluation was conducted using the CARE, STROBE, and JADAD tools. This systematic review showed that safety depended on drug indication. In COVID-19 patients, cardiac adverse effects, such as corrected QT interval prolongation, were relatively frequent (0–27.3% and up to 33% if combined with azithromycin), though the risk of torsade de pointes was low. Compared to non-COVID-19 patients, COVID-19 patients experienced a higher frequency of cardiac adverse effects regardless of the regimen used. Dermatological adverse effects affected 0–10% of patients with autoimmune diseases and COVID-19. A broad spectrum of neuropsychiatric adverse effects affected patients treated with CQ for malaria with variable frequencies and some cases were reported in COVID-19 patients. Gastrointestinal adverse effects occurred regardless of drug indication affecting 0–50% of patients. In conclusion, CQ and HCQ are two safe drugs widely used in the treatment of malaria and autoimmune diseases. However, recent findings on their cardiac and neuropsychiatric adverse effects should be considered if these drugs were to be proposed as antivirals again.

## 1. Introduction

Chloroquine (CQ) and hydroxychloroquine (HCQ), two safe drugs widely used in the treatment of malaria and autoimmune diseases, have become a global focus of attention due to early findings on their antiviral effectiveness against the novel SARS-CoV-2 coronavirus, which leads to what is known as coronavirus disease 2019 (COVID-19) [1,2]. In the context of the absence of specifically approved drugs for the treatment of SARS-CoV-2 pneumonia, previous evidence of the effects of CQ against coronaviruses [3,4], findings on the effects of CQ and HCQ on SARS-CoV-2 in vitro [1,2], and positive preliminary observational findings in China [5,6], justified clinical research on these drugs [7,8]. At the outbreak of the COVID-19 pandemic, these drugs were authorised as part of national emergency use programmes or clinical trials by the US Food and Drug Administration (FDA) and the European Medicines Agency (EMA) for patients affected by SARS-CoV-2 pneumonia [9,10], and the massive use of these treatments spread to different countries. Clinical research on and the use of these drugs were focused on three aspects: (i) treatment of patients with SARS-CoV-2 pneumonia, (ii) post-exposure prophylaxis of contacts [11], and (iii) prevention of SARS-CoV-2 infection among healthcare personnel [12].

Short-course CQ and HCQ regimens have traditionally been considered safe. Mild to moderate toxicity may occur occasionally, with symptoms including headache, malaise, dizziness, visual alterations, mild gastrointestinal and neurologic events, and itching being more or less common depending on the population treated [13]. However, increasing concern arose when these drugs were used in patients with COVID-19, whether alone or in combination with other drugs, due to their cardiac and neuropsychiatric adverse effects [14,15]. Large randomised clinical trials have ruled out the benefits of CQ or HCQ for COVID-19 outcomes and there is ongoing concern about the consequences that these treatments may have in patients with or without COVID-19 [16,17,18].

Besides short-term CQ and HCQ regimens, these drugs have been widely used for the long-term treatment of autoimmune inflammatory diseases. HCQ is currently recommended to treat systemic lupus erythematosus (SLE), and CQ and HCQ have been used for decades to treat rheumatoid arthritis (RA) and are commonly considered safe [14,15].

We hypothesise that the toxicity of short-course CQ or HCQ is low, but that an additive effect could occur when they are used in combination with other drugs or in concrete clinical conditions. The present work, therefore, has two main objectives: first, it seeks to assess and summarise the available literature on the early toxicity of CQ and HCQ alone or in combination with other drugs that have been used to treat COVID-19 in different clinical situations (such as malaria and other parasitic infections, or autoimmune conditions); and second, more specifically, it will assess the impact of drug combinations and pathological situations on the frequency of adverse drug effects in short-course regimens with CQ or HCQ.

## 2. Methods

We performed a systematic review of the literature on the safety of short-course treatments with CQ and HCQ with the goal of assessing the adverse effects of these drugs either alone or in combination with other drugs used to treat COVID-19, whether antivirals such as remdesivir, lopinavir plus ritonavir (LPVr), boosted darunavir (DRV), tenofovir, favipiravir, arbidol and ribavirin, antibiotics such as levofloxacin or azithromycin (AZM), immunomodulatory agents such as baricitinib, tocilizumab (TCZ), sarilumab, anakinra and interferons, corticosteroids such as dexamethasone, prednisone, prednisolone and methylprednisolone, anticoagulant heparins or low-weight heparins, or neutralizing antibodies and serotherapies. In our selection criteria we included case reports as well as case-control, cross-sectional, cohort, and randomised studies. Adverse effects associated with short-course regimens (≤14 days) were assessed by including both studies reporting information on short-term CQ and HCQ treatment regimens (e.g., for malaria) and those reporting long-term treatment regimens (e.g., for autoimmune diseases) in which early adverse effects were assessed or described. The main outcome of interest was the safety of CQ and HCQ and the frequency of adverse drug reactions during short-course regimens.

This systematic review was carried out in accordance with the PRISMA (Preferred Reporting Items for Systematic Reviews and Meta-Analyses) methodology [19] and was registered in the International Prospective Register of Systematic Reviews of the Centre for Reviews and Dissemination (PROSPERO) (registration number: CRD42020180708) [20].

### 2.1. Literature Search

We searched MEDLINE using PubMed, Embase using Ovid, CENTRAL (the Cochrane Central register of Controlled Trials), and LILACS (Literatura Latinoamericana y del Caribe en Ciencias de la Salud) for articles published from inception through 30 July 2021. We applied two searches, one designed to identify studies describing the safety of CQ or HCQ by itself and another designed to identify studies in which CQ or HCQ were combined with the drugs mentioned above. The combined MeSH and search terms used in the two search strategies in MEDLINE through PubMed are described in Tables S1 and S2 of Supplementary Material. Equivalent search strategies were applied for the Embase, CENTRAL, and LILACS searches. Once these searches were completed and all relevant articles obtained, the list of references at the end of each article was checked to identify additional relevant studies. No date restrictions were imposed. Only articles published in Spanish, Chinese, or English were included. Abstracts, posters, or book chapters were not included. Although a specific search was not performed on preprint databases, those articles in preprint form found through database searches or reference checks were included in the selection process.

### 2.2. Selection Process

Articles identified through the preliminary search were then screened for relevance to our study in two steps. First, the title and abstract of each article were independently checked by two reviewers for at least minimally relevant information on CQ, HCQ, and their safety. The resulting two lists of articles were compared and any differences were resolved by a third reviewer. The second step involved evaluating the full text of each article to confirm its relevance for this review. Articles were only included in the final set if they reported cases of adverse drug reactions to CQ or HCQ, or were case-control, cross-sectional, cohort, or randomised studies that reported information on the safety of these drugs for adult patients 18 years or older (it only was admitted if part of the population included adolescents ≥12 years in large cross-sectional, cohort, or randomised studies, not in the cases). Articles were excluded if they reported adverse drug reactions that occurred beyond the first 14 days of treatment; if they were related to intoxications (intakes of more than five times the Defined Daily Dose of 0.5 g of CQ base or 0.516 g of HCQ base) [21]; if the route of administration was not the oral route; if they were related to work-related exposure; if they were surveys of health professionals; if they assessed the validity of a diagnostic or screening technique; if they contained preclinical data (including in vitro or animal experimentation); if they were protocols, surveys, reviews, systematic reviews, scoping reviews, or meta-analyses; if the adverse drug reaction was associated with a combination of drugs other than those mentioned; if they did not indicate the temporal relationship between drug intake and the appearance of adverse drug reactions; or if they contained duplicate information (the same case or sample of patients reported in separate articles). Two reviewers performed this process independently. Subsequently, the results were compared, and discrepancies were resolved by a third reviewer to produce the final set of articles for synthesis.

### 2.3. Data Collection and Data Items

Working separately, two reviewers extracted from each of the articles a set of specific data about study design, participants, quality, and the results in a specific datasheet. The two resulting data compilations were compared, and any discrepancies were discussed and resolved with the participation of a third reviewer. We gathered data related to the design, the participants, the quality, and the results of each study. The final data selected for synthesis in this systematic review can be seen in Supplementary Material.

### 2.4. Quality Assessment, Risk of Bias in Individual Studies and across Studies

The CARE (CAse REport) Checklist was used to evaluate the quality of reporting in case reports and case series reports [22]. Case-control, cross-sectional, and cohort studies were assessed using the combined STROBE (STrengthening the Reporting of OBservational studies in Epidemiology) checklist [23]. The different items on the checklists mentioned were rated as “Yes” (=1 point), “Partly” (=0.5 point), “No” (=0 points), or “Not applicable”. We calculated an overall score for the quality of each study by dividing the total number of points scored per article by the number of items to produce a percentage. A low score indicated low quality, hence a higher risk of bias. We considered studies scoring between 75% and 100% to be of high quality, those between 50% and 74% of moderate quality, and those below 50% of low quality. If more than 50% of the assessed items were rated as “Not applicable”, the study was dismissed for quality assessment but not eliminated from our dataset. The quality of randomised studies was judged using the JADAD scale, which assigns a score ranging from 0 to 5 points such that the higher the score, the better the methodological quality [24]

### 2.5. Data Synthesis and Summary Measures

#### 2.5.1. Case Series, Case Reports, and Case-Control Studies

Data from these studies were synthesised in either table or narrative form. Case and case series reports were grouped according to drug or drugs reported (CQ or HCQ alone or in combination), drug indication, and the organ system affected by an adverse effect. No study was eliminated based on the risk of bias. Case-control studies were presented in a table showing reported adverse drug reactions. Presentation was ordered according to quality evaluation scores, presence of probability scales such as the Naranjo Adverse Drug Reaction Probability Scale for case and case series reports [25], and relevance of the evidence.

#### 2.5.2. Cross-Sectional, Cohort, and Randomised Studies

We presented the synthesised evidence following the SWiM (Synthesis Without Meta-analysis) guidelines [26] in table and narrative form. Studies were divided into studies on the safety of CQ or HCQ alone and studies in which CQ and/or HCQ were combined with one of the eligible drugs and also grouped according to drug indication. The rationale for this grouping of studies was our focus on drug safety and the influence of drug combinations and indications on this outcome. Initially, we did not use a standardised metric to present exposure and/or direction effects or *p* values, so we reported these effects in their original format, namely as mean differences, standardised mean differences, risk ratios, odds ratios, or risk differences. However, whenever possible, we calculated the frequency of each toxicity for each study, and a summary reporting the frequency of adverse events reported as a range of percentages was presented for each group mentioned above. Those studies reporting data on pregnant patients were synthesised using narratives and tables but not included in the quantitative data synthesis. In the case of combinations, only those cases in which more than one study reported enough data were included for quantitative data synthesis. Combinations in which only one study was found were presented separately. Whenever possible, data on adverse drug reactions frequency-adjusted for patient status or comorbidities or any other confounding factors were considered. Due to the heterogeneity of the populations included, drug exposure data, reported adverse drug reactions, and study methodologies, we did not consider a meta-analysis of the outcome effects. Studies were prioritised according to quality evaluation scores, sample size, and relevance of the evidence.

## 3. Results

**Figure 1:**
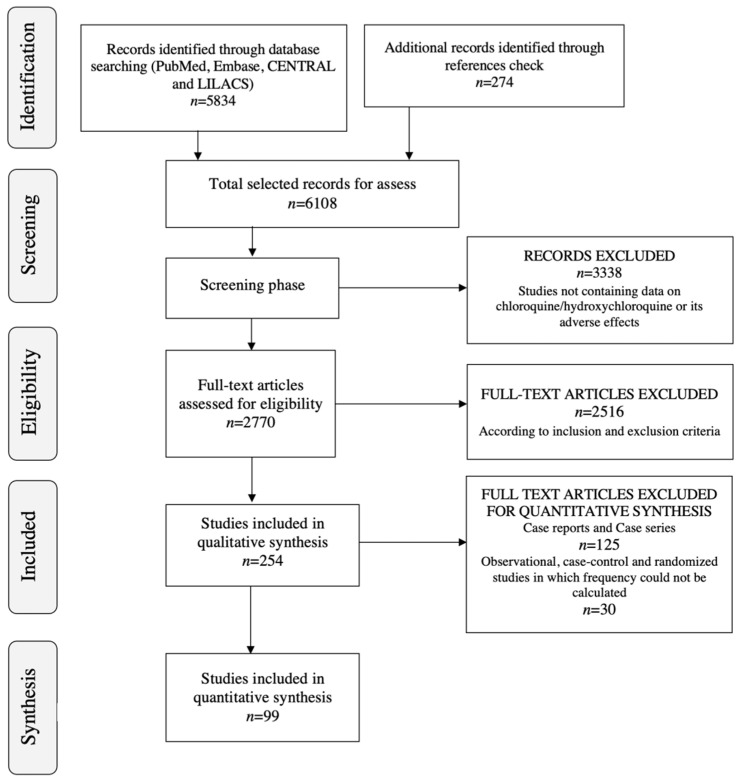
Safety of chloroquine and hydroxychloroquine. Selection process, flow diagram.

The preliminary online database search yielded a set of 6108 articles, of which 2942 articles were identified through MEDLINE using PubMed, 1977 were identified through Embase using Ovid, 683 were identified through CENTRAL, 232 were identified through LILACS, and 274 more were identified by checking article reference lists. Of this initial set of 6108, 3338 articles were excluded in the title and abstract screening process. Of the remaining 2770 articles, an additional 2516 were excluded for the content eligibility reasons described above (eligibility criteria are summarised in Table S3 of Supplementary Material). The full selection process yielded a final set of 254 studies for this systematic review (Figure 1) [27,28,29,30,31,32,33,34,35,36,37,38,39,40,41,42,43,44,45,46,47,48,49,50,51,52,53,54,55,56,57,58,59,60,61,62,63,64,65,66,67,68,69,70,71,72,73,74,75,76,77,78,79,80,81,82,83,84,85,86,87,88,89,90,91,92,93,94,95,96,97,98,99,100,101,102,103,104,105,106,107,108,109,110,111,112,113,114,115,116,117,118,119,120,121,122,123,124,125,126,127,128,129,130,131,132,133,134,135,136,137,138,139,140,141,142,143,144,145,146,147,148,149,150,151,152,153,154,155,156,157,158,159,160,161,162,163,164,165,166,167,168,169,170,171,172,173,174,175,176,177,178,179,180,181,182,183,184,185,186,187,188,189,190,191,192,193,194,195,196,197,198,199,200,201,202,203,204,205,206,207,208,209,210,211,212,213,214,215,216,217,218,219,220,221,222,223,224,225,226,227,228,229,230,231,232,233,234,235,236,237,238,239,240,241,242,243,244,245,246,247,248,249,250,251,252,253,254,255,256,257,258,259,260,261,262,263,264,265,266,267,268,269,270,271,272,273,274,275,276,277,278,279,280].

### 3.1. Study Characteristics and Results of Individual Studies

**Figure 2:**
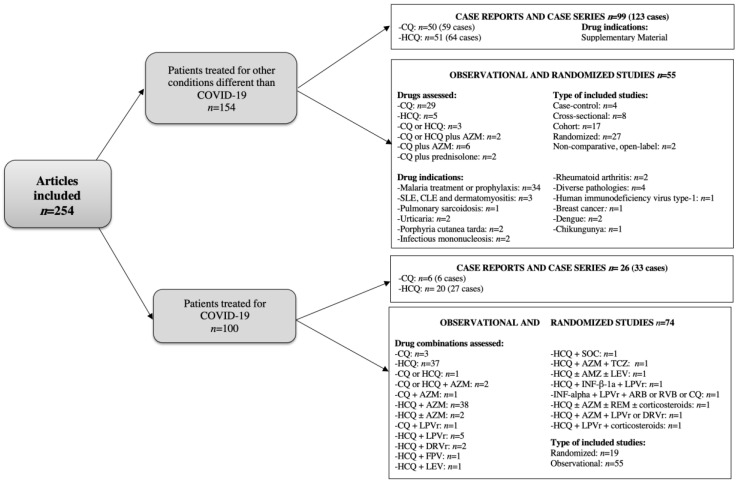
Study characteristics and results of individual studies, flow diagram.

Figure 2 describes the main characteristics of the articles included.

#### 3.1.1. Patients Treated for Conditions Other Than COVID-19

##### Case and Case Series Reports

A total of 99 articles reporting 123 cases were found [27,28,29,30,31,32,33,34,35,36,37,38,39,40,41,42,43,44,45,46,47,48,49,50,51,52,53,54,55,56,57,58,59,60,61,62,63,64,65,66,67,68,69,70,71,72,73,74,75,76,77,78,79,80,81,82,83,84,85,86,87,88,89,90,91,92,93,94,95,96,97,98,99,100,101,102,103,104,105,106,107,108,109,110,111,112,113,114,115,116,117,118,119,120,121,122,123,124,125]. These cases are summarised in Table 1 and Table 2 and described more fully in Tables S4–S12 of Supplementary Material part 2.

##### Case-Control, Cross-Sectional, Cohort, and Randomised Studies

Forty-seven articles reported data on the safety of CQ or HCQ when used alone and nine others provided data for when CQ or HCQ were combined with one of the drugs of interest [126,127,128,129,130,131,132,133,134,135,136,137,138,139,140,141,142,143,144,145,146,147,148,149,150,151,152,153,154,155,156,157,158,159,160,161,162,163,164,165,166,167,168,169,170,171,172,173,174,175,176,177,178,179,180]. Table 3 and Table 4 show the frequency of adverse drug reactions as reported in these studies. Data from case-control and other observational studies in which the frequency could not be calculated are presented in Table 5. Full data from these studies can be found in Tables S13–S20 of Supplementary Material part 2.

#### 3.1.2. Patients Treated for COVID-19

##### Case and Case Series Reports

A total of 26 articles reporting cases related to the safety of HCQ or CQ during treatment for COVID-19 were found [181,182,183,184,185,186,187,188,189,190,191,192,193,194,195,196,197,198,199,200,201,202,203,204,205,206]. Table 6 synthesises the data from the cases reporting HCQ and CQ adverse drug reactions in COVID-19-affected patients and Table S21 of Supplementary Material part 2 provides full details.

##### Case-Control, Cross-Sectional, Cohort, and Randomised Studies

A total of 74 articles reporting data on the safety of HCQ or CQ in patients treated for COVID-19 were found [207,208,209,210,211,212,213,214,215,216,217,218,219,220,221,222,223,224,225,226,227,228,229,230,231,232,233,234,235,236,237,238,239,240,241,242,243,244,245,246,247,248,249,250,251,252,253,254,255,256,257,258,259,260,261,262,263,264,265,266,267,268,269,270,271,272,273,274,275,276,277,278,279,280]. Table 7 and Table 8 show the frequency of adverse drug reactions as reported in these studies. The full data is provided in Table S22 of the Supplementary Material part 2.

**Table 1:** Summary of studies included: case reports and case series reports related to HCQ adverse events.

Organ Affected	N° of Patients (n° of Studies)	Adverse Effect (N° of Patients) (First Author, Year)	Indication of HCQ (n° of Patients)	Long-Term Consequences (n° of Patients)
Dermatological	41 (28)	DRESS (3) (Volpe A et al., 2008, Randhawa A et al., 2018, Girijala RL et al., 2019) [48,60,62]	Seronegative polyarthritis (2) Suspected Sjögren’s like process (1)	Resolution (3)
		Severe pruritus (1) (Gül U et al., 2006) [45]	Discoid lupus erythematosus (1)	Resolution (1)
		Pemphigus vulgaris (1) (Ghaffarpour G et al., 2006) [44]	Rheumatoid arthritis (1)	After 3 w the lesions cleared with only a mild post-inflammatory hyperpigmentation (1)
		Sweet’s syndrome (1) (Manzo C et al., 2019) [63]	Sjögren syndrome (1)	Resolution (1)
		Inverse psoriasis (1) (Ullah A et al., 2019) [64]	Rheumatoid arthritis (1)	Resolution (1)
		Mild cutaneous eruptions (1) (Matsuda T et al., 2017) [57]	Lupus erythematosus (1)	Resolution (1)
		AGEP (11) (Assier-Bonnet et al., 1996, Evans CC et al., 2004, Atzori L et al., 2007, Bailey K et al., 2013, Soria A et al., 2015, Pearson KC et al., 2016, Mercogliano C et al., 2018, Matsuda-Hirose H et al., 2020) [38,43,47,51,54,55,59,65]	SLE and related disorders (3) NA (1) Erythematous facial dermatitis (1) Photosensitivity (1) Arthritis and related rheumatic disorders (4) Mucinosis (1)	Resolution (10) NA (1)
		Acute pustular psoriasis (1) (Welsch MJ et al., 2003) [42]	Sjögren syndrome (1)	Resolution (1)
		Stevens-Johnson syndrome (1) (Leckie MJ et al., 2002) [41]	Rheumatoid arthritis (1)	The rash improved but persisted (1)
		Erythema multiforme (1) (Abou Assalie N et al., 2017) [56]	SLE (1)	Resolution (1)
		Fatal toxic epidermal necrolysis (2) (Murphy M et al., 2001, Cameron MC et al., 2014) [40,52]	Seropositive nodular rheumatoid disease (1) SLE (1)	Death (2)
		Psoriasis (1) (Gray RG et al., 1985) [34]	Seronegative rheumatoid arthritis (1)	Resolution (1)
		Severe psoriasis exacerbation (1) (Luzar MJ et al., 1982) [31]	Psoriatic arthropathy (1)	Resolution (1)
		Hypersensitivity rash (5) (Mates M et al., 2006, Awad P et al., 2013) [46,50]	Arthritis and related rheumatic disorders (4) Chronic cutaneous lupus (1)	Resolution (1) NA (4)
		Erythema annulare centrifugum (1) (Hudson LD et al., 1985) [35]	Suspected SLE (1)	Resolution (1)
		Pustular eruption (1) (Pastushenko I et al., 2015) [53]	Rheumatoid arthritis (1)	Resolution (1)
		Photosensitivity (2) (Soria A et al., 2015) [54]	Rheumatism (1) Autoimmune bullous skin disease (1)	NA (2)
		AGEP/DRESS (1) (Soria A et al., 2015) [54]	Granuloma annulare (1)	Resolution (1)
		Urticaria (2) (Soria A et al., 2015) [54]	Jessner-Kanof (1) Cutaneous lupus erythematous (1)	Resolution (2)
		MPE (2) (Soria A et al., 2015) [54]	Gougerot-Sjögren syndrome (1) Cutaneous lupus erythematous (1)	Resolution (2)
		Generalised pustular rash (1) (Lotem M et al., 1990) [36]	Pemphigus erythematosus (1)	Resolution (1)
Psychiatric	2 (2)	Psychosis (1) (Ward WQ et al., 1985) [74]	Lupus erythematosus (1)	Resolution (1)
		Auditory and visual hallucination (1) (Ganjei Z et al., 2021) [125]	Discoid lupus erythematosus (1)	Resolution (1)
Neurologic	1 (1)	Significant psychomotor agitation (1) (Manzo C et al., 2017) [63]	Rheumatoid arthritis (1)	Resolution (1)
Cardiac	3 (3)	Complete heart block (1) (Comín-Colet J et al., 2001) [96]	SLE (1)	Resolution (1)
		Implanted pacemaker failure (1) (Huang PH et al., 2003) [97]	Rheumatoid arthritis (1)	Resolution (1)
		QT-interval prolongation (1) Morgan ND et al., 2013 [99]	SLE (1)	QT relatively normal after a year (1)
Hematologic and metabolic	4 (4)	Hypoglycaemic coma (1) (Shojania K et al., 1999) [101]	Rheumatoid polyarthritis (1)	Resolution (1)
		Hypoglycaemia (1) (Winter EM et al., 2011) [102]	Osteoarthritis (1)	Resolution (1)
		Thrombocytopaenia (1) (Demir D et al., 2014) [104]	Used erroneously as a pain killer (1)	Resolution (1)
		Thrombotic thrombocytopaenic purpura (1) (Fromm LM et al., 2017) [105]	Rheumatoid arthritis (1)	Death related to cardiac failure (1)
Hepatic	4 (4)	Severe acute hepatitis (1) (Giner Galvañ V et al., 2007) [111]	Arthritis (1)	Resolution (1)
		Liver injury (1) (Sunkara B et al., 2018) [112]	Subacute cutaneous lupus erythematosus (1)	Resolution (1)
		Fulminant hepatic failure (1) (Makin AJ et al., 1994) [114]	SLE (1)	Death (1)
		Bullous rash and acute hepatitis (1) (Kutz DC et al., 1995) [115]	SLE (1)	Resolution (1)
Other	6 (6)	Porphyria variegata precipitation (1) (Baler GR et al., 1976) [116]	SLE (1)	Resolution (1)
		Severe vacuolar myopathy (1) (Bolaños-Meade J et al., 2005) [119]	cGVHD (1)	Resolution (1)
		Anaphylaxis (1) (Donado CD et al., 2010) [121]	SLE (1)	Resolution (1)
		Two episodes of urinary incontinence (1) (Carnovale C et al., 2013) [122]	Rheumatoid arthritis (1)	Resolution (1)
		Diffuse interstitial lung disease (1) (Català R et al., 2015) [123]	Polymorphic light eruption (1)	Resolution (1)
		Acute eosinophilic pneumonia (1) (Ishiguro Y et al., 2019) [124]	Chilblain lupus erythematosus (1)	Resolution (1)
Sense organs	3 (3)	Severe positional vertigo (1) (Prince DS et al., 1975) [107]	Rheumatoid arthritis (1)	Resolution (1)
		Severe vestibular toxicity (1) (Malik MK et al., 1977) [108]	Malaria (1)	Bilateral complete canal paresis (1)
		Complete ageusia (1) (Fleury O et al., 2009) [110]	SLE (1)	Resolution (1)

AGEP: Acute generalised exanthematous pustulosis, cGVHD: chronic graft-versus-host disease, DRESS: Drug rash with eosinophilia and systemic symptoms, HCQ: Hydroxychloroquine, MPE = maculopapular exanthema, NA: not available/not applicable, SLE: systemic lupus erythematosus, w: weeks.

**Table 2:** Summary of studies included: case reports and case series reports related to CQ adverse events.

Organ Affected	N° of Patients (n° of Studies)	Adverse Effect (n° of Patients) (First Author, Year)	Indication of CQ (n° of Patients)	Long-Term Consequences (n° of Patients)
Dermatological	12 (12)	Exacerbation of psoriasis and arthritis (1) (Fisher S, 1961) [27]	Psoriasis (1)	Death due to toxaemia from staphylococcic peritonitis (1)
		Eczema (1) (Skog E, 1975) [28]	Malaria prophylaxis (1)	NA (1)
		Toxic epidermal necrolysis (1) (Kanwar AJ, 1976) [29]	Suspected malaria (1)	Resolution (1)
		Exacerbation of psoriasis (1) (Olsen TG, 1981) [30]	Malaria (1)	Resolution (1)
		Severe pruritus (1) (Spencer HC, 1982) [32]	Malaria (1)	Resolution (1)
		Pruritus (1) (Bhasin V, 1984) [33]	Malaria (1)	Resolution (1)
		Erythrodermic psoriasis (1) (Vestey JP, 1992) [37]	Psoriasis (1)	Psoriasis remained well controlled with usual treatment (1)
		Pustular eruption (1) (Wilairatana P, 1998) [39]	Malaria (1)	After discontinuation, the eruption quickly resolved with mild desquamation (1)
		Stevens–Johnson syndrome (1) (Das JK, 2011) [49]	Malaria (1)	Resolution (1)
		Photosensitivity (1) (Soria A, 2015) [54]	SLE (1)	NA (1)
		Palmo-plantar exfoliation (1) (Nair PA, 2017) [58]	Malaria (1)	NA (1)
		Urticaria (1) (Balamurugesan K, 2019) [61]	Malaria (1)	Resolution (1)
Psychiatric	20 (15)	Psychosis (9) (Burrell Z, 1958; Dornhorst AC, 1963; Rab SM, 1963; Oscar L, 1964; Kabir SM, 1969; Bomb BS, 1975; Ward WQ, 1985; Choughule A, 2019) [66,67,68,69,70,71,74,81]	Acute myocardial infarction (1) Rheumatoid arthritis (1) Hepatic or intestinal amoebiasis (4) Malaria (3)	Resolution (8) NA (1)
		Moderate to severe depression (2) (Das EM, 1981) [72]	Malaria (2)	Resolution (2)
		Mania (5) (Akhtar S, 1993; Plesnicar BK, 2013) [75,78]	Malaria (4) Rheumathoid arthritis (1)	Resolution (3) Disorder remains beyond 5 months (1) Mild attention deficit and memory difficulties (1)
		Psychotic disorder with symptoms of depersonalization and anxiety (1) (Telgt DS, 2005) [76]	Malaria (1)	Resolution (1)
		Organic delusional (schizophrenia-like) disorder (1) (Sahoo S, 2007) [77]	Malaria (1)	Resolution (1)
		Exacerbation of bipolar disorder (maniac episode with psychotic features) (1) (Bogaczewicz J, 2014) [79]	SLE and arthritis (1)	Resolution (1)
		Paranoid-like disorder (1) (Bogaczewicz A, 2016) [80]	SLE (1)	Resolution (1)
Neurologic	15 (11)	Seizures (4), grand mal seizure (1) (Torrey EF, 1968; Martin AN 2016) [82,92]	Hepatic or intestinalamoebiasis (4) Prophylactic treatment of gastrointestinal parasitic infection (1)	Resolution (4) NA (1)
		Involuntary movements (1) (Umez-Eronini EM, 1977) [83]	Fever (1)	Resolution (1)
		Akathisia and persistent protrusion of the tongue (1) (Singh RP, 1981) [84]	Malaria (1)	Resolution (1)
		Auditory hallucinations, acute psychotic behaviour, difficulty in swallowing, protrusion of the tongue, and marked extrapyramidal rigidity (1) (Singh RP, 1981) [84]	Malaria (1)	Resolution (1)
		Serious tonic-clonic convulsion (1) (Fish DR, 1988) [85]	Malaria prophylaxis (1)	Serious consequences (1)
		Severe cerebral ataxia with extrapyramidal movements (1) (James RF, 1988) [86]	Malaria (1)	Resolution (1)
		Transient global amnesia (1) (Cras P, 1990) [87]	Malaria prophylaxis (1)	Resolution (1)
		Retinopathy and persisting mild ocular myasthenia (1) (De Bleecker J, 1991) [88]	Malaria (1)	Symptoms persisted more than 10 years after drug discontinuation (1)
		Tonic-clonic seizures (2) (Adamolekun B, 1992, Ebenso BE, 1998) [89,91]	Suspected malaria (1) Erythema nodosum leprosum (1)	Resolution (2)
		Non-convulsive status epilepticus (1) (Mülhauser P, 1995) [90]	Malaria prophylaxis (1)	Resolution (1)
Cardiac	3 (3)	Cardiovascular collapse (1) (Sogani RK, 1986) [94]	Dermatologic problem (1)	NA (1)
		Cardiac arrhythmia (1) (Siqueira-Batista R, 1998) [95]	Malaria (1)	Resolution (1)
		Syncopal attacks and torsade de pointes (1) (Yelve K, 2012) [98]	Hepatic and intestinal amoebiasis (1)	Resolution (1)
Hematologic and metabolic	2 (2)	Hypoglycaemia (1) (Abu-Shakra M, 1994) [100]	Psoriatic arthritis (1)	Resolution (1)
		Methaemoglobinaemia (1) (Rizvi I, 2012) [103]	Fever (1)	Resolution (1)
Sense organs	3 (3)	Diplopia and persistent blurred near vision (1) (Rubin ML, 1970) [106]	Hypercalcemia associated with sarcoidosis (1)	Resolution (1)
		Vestibular toxicity (1) (Malik MK, 1977) [108]	Malaria (1)	NA (1)
		Loss of hearing (1) (Dwivedi GS,1978) [109]	Malaria (1)	Tinnitus and hearing loss have so far persisted for 5.5 months without improvement (1)
Hepatic	1 (1)	Hepatotoxic reaction (1) (Liu AC, 1995) [113]	Malaria prophylaxis (1)	Resolution (1)
Other	3 (3)	CQ overdose with severe headache, dizziness on standing, nausea and blurred vision (1) (Davis TM, 2003) [117]	Malaria (1)	Resolution (1)
		Severe myopathy (1) (Richter JG, 2003) [118]	SLE with arthralgia and renal involvement (1)	NA (1)
		Acute eosinophilic pneumonitis (1) (Knudsen L, 2009) [120]	Mild rosacea (1)	NA (1)

CQ: Chloroquine, NA: not available/not applicable, SLE: systemic lupus erythematosus.

**Table 3:** Summary of included studies reporting data on the frequency of early adverse events of CQ or HCQ alone (except COVID-19).

First Author, Year	Type of Study	Drug, Sample Size	Gastrointestinal Disorders			Hepatobiliary		Neurological	Sense Organs	Dermatological	Other
			Diarrhea	Nausea/Vomiting	Abdominal Pain/Dyspepsia	Bilirubin/GGT Increase	Transaminase Increase	Headache/Dizziness			
Malaria treatment and prophylaxis											
Weinke T, 1992 [126]	OBS	CQ *n* = 301	NA	NA	4.7%	NA	NA	Headache 0.3% Dizziness 0.3%	Tinnitus 0.7%	Exanthema 0% Pruritus 3.3%	Psychosis 0% ECG changes 0%
Bussaratid V, 2000 [127]	OBS	CQ *n* = 1189	NA	NA	NA	NA	NA	NA	NA	Pruritus 1.9%	NA
Olayemi O, 2003 [128]	OBS	CQ *n* = 200	NA	NA	NA	NA	NA	NA	NA	Pruritus 64.5%	NA
Gama H, 2009 [129]	OBS	CQ *n* = 542	NA	NA	NA	NA	NA	NA	NA	Pruritus 30.1%	NA
Jeevangi SR, 2010 [130]	OBS	CQ *n* = 128	NA	Nausea 9.4% Vomiting 9.4%	NA	NA	NA	NA	Tinnitus 9.4%	NA	Anorexia 9.4%
Ballut PC, 2013 [131]	OBS	CQ *n* = 510	NA	NA	NA	NA	NA	NA	NA	Pruritus 20.4%	NA
Gozal D, 1991 [138]	RAN	CQ *n* = 78	3.8%	Nausea 11.5% Vomiting 8.9%	24.4%	NA	NA	Headache 10.2%	Visual disturbances 1.3%	Pruritus 1.3%	Anorexia 16.7% Oral ulcers 19.2%
McClean K, 1992 [139]	RAN	CQ *n* = 18	NA	NA	NA	NA	NA	NA	NA	Pruritus 44.4%	NA
Yanze MF, 2001 [171]	RAN	CQ *n* = 60	8.3%	Nausea 10.0% Vomiting 3.3%	11.7%	NA	NA	Headache 3.3% Dizziness 5.0%	NA	Pruritus 5.0%	NA
Dunne MW, 2005 [140]	RAN	CQ *n* = 102	NA	Nausea 4.9% Vomiting 7.8%	NA	NA	NA	Headache 1.0%	NA	Cutaneous drug eruption 2.9% Pruritus 7.8%	Myalgia 0%
Tagbor H, 2006 [172]	RAN	CQ *n* = 225	NA	Nausea 22.2% Vomiting 31.1%	NA	NA	NA	Dizziness 43.1%	NA	Pruritus 39.1%	Weakness 47.1%
Ratcliff A, 2007 [141]	RAN	CQ *n* = 40	NA	Vomiting 10.0%	NA	NA	NA	NA	NA	NA	NA
Massaga JJ, 2008 [142]	RAN	CQ *n* = 20	NA	NA	30%	NA	0%	Headache 25.0%	NA	NA	Weakness 20.0% Fever 15.0%
Dunne MW, 2005 (2) [146]	RAN	CQ *n* = 16	6%	Nausea 0.0% Vomiting 0.0%	NA	NA	NA	Dizziness 19.0%	NA	Pruritus 19.0%	Pharyngitis 6.0% Fatigue 13.9%
Poravuth Y, 2011 [149]	RAN	CQ *n* = 228	NA	Vomiting 1.8%	NA	0%	0.43%	Headache 1.3% Dizziness 2.2%	NA	NA	Fatigue 0.4% Anorexia 0.9% QT prolongation 2.7%
Watt G, 1988 [151]	RAN	CQ *n* = 10	20%	Vomiting 10.0%	NA	NA	NA	NA	NA	Pruritus 10.0%	NA
Systemic lupus erythematosus and cutaneous lupus erythematosus											
Kishi CJ, 2018 [13]	OBS	HCQ *n* = 31	6.4%	NA	3.2%	NA	NA	Dizziness 3.2%	Visual disturbances 3.2%	Erythema 6.4%	NA
Gonzalez CD, 2019 [134]	OBS	HCQ/CQ *n* = 136	NA	NA	NA	NA	NA	NA	NA	Cutaneous drug eruption 4.0%	NA
Chasset F, 2018 [132]	OBS	HCQ/CQ *n* = 64	NA	NA	NA	NA	NA	NA	NA	Exanthema 1.6%c	NA
Pulmonary sarcoidosis											
Baltzan M, 1999 [145]	RAN	CQ *n* = 23	NA	NA	4.3%	NA	NA	NA	NA	Cutaneous drug eruption 4.3%	Anxiety 4.3%
Rheumatoid arthritis											
Haar D, 1993 [143]	RAN	HCQ *n* = 28	NA	NA	3.6%	NA	NA	NA	NA	NA	NA
Dermatomyositis											
Gonzalez CD, 2019 [134]	OBS	HCQ/CQ *n* = 44	NA	NA	NA	NA	NA	NA	NA	Cutaneous drug eruption 5.0%	NA
Refractory chronic urticaria and chronic autoimmune urticaria											
Seth S, 2017 [137]	OBS	HCQ *n* = 45	NA	NA	NA	NA	NA	NA	NA	Pruritus 2.2%	NA
Reeves GE, 2004 [148]	RAN	HCQ *n* = 9	NA	NA	NA	NA	NA	NA	NA	NA	Significant toxicity 0%
Porphyria cutanea tarda											
Petersen CS, 1992 [135]	OBS	HCQ *n* = 72	NA	Nausea 20.8% Vomiting 12.5%	20.8%	Icterus 1.4%	95.8%	Headache 25.0%	NA	NA	Arthralgia 5.5% Hepatomegaly 2.8% Myalgia 26.4%
Rossmann-Ringdahl, 2007 [136]	OBS	CQ *n* = 57	NA	NA	NA	NA	100%	NA	NA	NA	NA
Human immunodeficiency virus type 1											
Sperber K, 1995 [147]	RAN	HCQ *n* = 19	NA	NA	NA	NA	NA	NA	NA	NA	Adverse reactions 0%
Breast cancer											
Arnaout A, 2019 [150]	RAN	CQ *n* = 46	17.4%	Nausea and/or abdominal cramps 23.9%	NA	NA	NA	Dizziness 8.7%	Visual symptoms 8.7% Documented visual changes 0% Auditory symptoms 2.2%	NA	Fatigue 2.2% Muscle weakness 8.7% Dry mouth 4.3%
Chikungunya acute infection											
De Lamballerie X, 2008 [154]	RAN	CQ *n* = 27	NA	NA	NA	NA	NA	NA	NA	NA	Mild adverse reactions (mainly nausea and pruritus) 25.9%
Dengue											
Tricou V, 2010 [155]	RAN	CQ *n* = 153	NA	Vomiting 4.1%	NA	NA	NA	NA	NA	NA	NA
Borges MC, 2013 [156]	RAN	CQ *n* = 19	NA	NA	NA	NA	NA	NA	Blurred vision 5.2%	NA	Loss of consciousness 5.2%
Infectious mononucleosis											
Cowley RG, 1962 [152]	OBS	CQ *n* = 20	Gastrointestinal complaints (anorexia, nausea, vomiting) 60%			NA	NA	NA	NA	NA	NA
Schumacher HR, 1963 [153]	OBS	CQ *n* = 5	NA	NA	NA	NA	NA	NA	NA	NA	Complications 0%

CQ: chloroquine, ECG: electrocardiogram, GGT: gamma-glutamyl transferase, HCQ: hydroxychloroquine, NA: not available/not applicable, OBS: observational, RAN: randomised.

**Table 4:** Summary of studies: early adverse events of CQ in combination with AZM in patients affected by malaria or who received prophylactic treatment.

Type of Study, Arm and Sample Size	Randomised AZM Plus CQ *n* = 114	Randomised AZM Plus CQ *n* = 113	Randomised AZM Plus CQ *n* = 1446	Randomised AZM Plus CQ *n* = 64	Single-Arm AZM Plus CQ *n* = 168	Single-Arm AZM (2 g) Plus CQ *n* = 110	Randomised AZM(1 g) Plus CQ *n* = 197	Randomised AZM (0.5 g) Plus CQ *n* = 81
Author, year	Sagara I, 2014 [173]		Kimani J, 2016 [179]	Dunne MW, 2005 [146]	Phiri K, 2016 [180]	Kshirsagar NA, 2017 [174]		
Any AEs	78.1%	70.8%	68.9%	20%	NA	44%	26%	10%
Abdominal pain/discomfort	7.0%	11.5%	8.3–8.5%	NA	NA	0%	3%	0%
Asthenia	5.3%	8.0%	16.6%	NA	NA	NA	NA	NA
Blood/lymphatic disorders	NA	NA	14.3%	NA	NA	NA	NA	NA
Dehydration	NA	NA	NA	NA	NA	4%	0%	0%
Diarrhea	5.3%	9.7%	14.2%	3%	NA	12%	4%	0%
Dizziness	9.6%	15.9%	32.0%	0%	19.6%	NA	NA	NA
Fatigue	0%	3.5%	5.6%	NA	4.2%	NA	NA	NA
Gastritis	NA	NA	NA	NA	NA	4%	2%	1%
Headache	13.2%	17.7%	20.7%	NA	6.0%	0%	2%	0%
Infections	NA	NA	30.1%	Pharyngitis 0%	Parasitic infection 7.1% Upper respiratory infection 4.2%	NA	NA	NA
Nausea	7.9%	8.8%	14.9%	6%	3.6%	30%	0%	0%
Pain	1.8%	5.3%	NA	NA	NA	NA	NA	NA
Palpitations	2.6%	0%	NA	NA	NA	NA	NA	NA
Paraesthesia	NA	NA	NA	NA	NA	0%	3%	0%
Pruritus	50.9%	28.3%	NA	2%	Pruritus 7.7% Generalised pruritus 5.4%	4%	15%	6%
Visual disorders	NA	NA	10.1%	NA	NA	NA	NA	NA
Vomiting	15.8%	3.5%	45.2%	8%	20.8%	18%	4%	1%

AEs: adverse events, AZM: azithromycin, CQ: chloroquine, g: grams, NA: not available/not applicable.

**Table 5:** Adverse effects reported on case-control studies and observational studies not included in data synthesis.

First Author, Year	Drug, Indication	Adverse Effect
CQ or HCQ alone		
Obasikene G, 2012 [162]	CQ malaria	Ototoxicity
Ajayi AA, 1989 [157]	CQ malaria	Pruritus
Castro-Cavadía CJ, 2020 [166]	CQ malaria	AEs were confused in frequency and intensity with malaria symptoms and signs
Schneider C, 2013 [163]	CQ malaria	Neuropsychiatric disorder
Sarathi P, 2014 [165]	CQ malaria	Psychiatric manifestation
Dugué A, 2004 [167]	HCQ NA	Muscular adverse events
Sidoroff A, 2007 [169]	CQ and HCQ NA	AGEP
George AO, 2004 [161]	CQ malaria	Pruritus
Patel KJ, 2007 [168]	CQ NA	Gastritis
Emerole CG, 2014 [164]	CQ malaria	Loss of visual acuity
Ajayi AA, 1998 [175]	CQ malaria	Pruritus
Katugampola G,1990 [158]	CQ malaria	Worsening of psoriasis
Frías Salcedo JA, 1992 [159]	CQ malaria	Visual and gastrointestinal disturbances or pruritus and headache
Yanze MF, 2001 [171]	CQ malaria	Headache, diarrhea, abdominal pain, nausea, pruritus, dizziness, and vomiting
Walsh DS, 1999 [170]	CQ malaria	Abdominal discomfort and diarrhea
Garcia P, 2020 [280]	HCQ COVID-19	Psychiatric disorders
CQ or HCQ combined with other drug		
Vouri SM, 2020 [177]	CQ and HCQ plus AZM autoimmune disease	Sudden cardiac arrest, ventricular arrhythmias, and cardiac symptoms
Sarayani A, 2021 [178]	CQ and HCQ plus AZM NA	CQ and HCQ appeared not to be associated with a safety risk related to torsade de pointes or QT prolongation when used alone, when used with AZM they were associated with a potential safety risk
Ajayi AA, 1991 [175]	CQ plus prednisolone malaria	Pruritus
Adebayo RA, 1997 [176]	CQ plus prednisolone malaria	Pruritus

AE: adverse event, AGEP: acute generalised exanthematous pustulosis, AZM: azithromycin, COVID-19: Coronavirus Disease 2019, CQ: chloroquine, HCQ: hydroxychloroquine, NA: not available/not applicable, TCZ: tocilizumab.

**Table 6:** Summary of studies included: COVID-19 case reports related to chloroquine or hydroxychloroquine adverse effects in patients with suspected or confirmed COVID-19 or who were prophylactically treated.

Organ Affected	Number of Patients	Adverse Effect, Drug Combination If Required (Number of Patients) (First Author, Year)	Long-Term Consequences
CQ			
Cardiac	2	Major QT prolongation and recurrent torsade de pointes (1) (Szekely Y, 2020) [188]	ECGs showed gradual normalization of QT interval
		Wide complex tachycardia, along with AZM (1) (Gracia-Ramos AE, 2021) [189]	Death after cardiac arrest
Hematologic and metabolic	1	G6PD deficiency-associated haemolysis and methaemoglobinaemia (1) (Kuipers MT, 2020) [184]	The patient’s methaemoglobin normalized within 6 days
Psychiatric	3	Psychotic symptoms, along with AZM (1) (Benjelloun R, 2020) [195]	Resolution after 48 h
		Acute and intense anxiety, along with AZM (1) (Benjelloun R, 2020) [195]	No
		Psychosis episode (1) (Ambar Akkaoui M, 2021) [193]	NA
HCQ			
Cardiac	6	Right bundle brunch block and critically prolonged QTc (1) (Asli R, 2020) [181]	Resolution
		QT interval prolongation in a patient on AZM (1) (Mitra RL, 2020) [186]	Death owing to progressive metabolic acidosis and multiorgan system failure
		QTc prolongation and torsade de pointes, along with dexamethasone (1) (Aslam W, 2021) [190]	NA
		Suspected HCQ-induced sinus bradycardia and QTc interval prolongation (1) (Kang Y, 2020) [191]	A temporary pacemaker was implanted
		QTc prolongation, along with AZM (1) (Patel J, 2020) [192]	No
		Sinus bradycardia, along with AZM and corticosteroids (1) (Patel J, 2020) [192]	No
Dermatological	12	Psoriasis exacerbation (1) (Kutlu Ö, 2020) [185]	NA
		AGEP with erythema multiforme-like lesions (1) (Robustelli Test E, 2020) [187]	Slow but progressive resolution
		Rash (1) (Kurd R, 2020) [202]	NA
		AGEP (1) (Enos T, 2020) [199]	Resolved with prednisone after 38 days
		AGEP (1) (Delaleu J, 2020) [198]	NA
		Erythema multiforme (1) (Monte-Serrano J, 2020) [197]	NA
		Urticaria with maculopapular rash, palmoplantar itching (1) (Sardana K, 2020) [196]	NA
		Urticaria (1) (Sardana K, 2020) [196]	NA
		Palmoplantar itching (1) (Sardana K, 2020) [196]	NA
		DRESS syndrome, along with AZM and LPVr (1) (Castro Jiménez A, 2021) [200]	NA
		Purpuric erythematous rash with non-follicular pustules, on the trunk and limps, with intense involvement of armpits and scalp (1) (Abadías-Granado I, 2021) [201]	No
		Purpuric erythematous rash with non-follicular pustules and targetoid lesions on the back (1) (Abadías-Granado I, 2021) [201]	No
Hematologic, muscular and metabolic	6	Worsening of haemolysis (1) (Beauverd Y, 2020) [183]	NA
		Haemolysis in a G6DP-deficient patient (1) (Maillart E, 2020) [204]	NA
		Haemolytic anemia in a G6DP-deficient patient (1) (Aguilar J, 2020) [206]	NA
		Acute haemolytic anemia in a G6DP-deficiency patient (1) (Chaney SI, 2020) [205]	NA
		Thrombotic thrombocytopaenic purpura (1) (Arıkan F, 2020) [203]	No
Hepatic	1	Hepatotoxicity (1) (Falcão MB, 2020) [182]	NA
Ophthalmology	1	Myasthenic syndrome (1) (Koc G, 2020) [194]	No
Gastrointestinal	1	Nausea, vomiting, diarrhea (1) (Patel J, 2020) [192]	NA

AGEP: acute generalised exanthematous pustulosis, AZM: azithromycin, CQ: chloroquine, DRESS: drug rash with eosinophilia and systemic symptoms, ECGs: electrocardiograph, G6PD: glucose-6-phosphate dehydrogenase, HCQ: hydroxychloroquine, LPVr: lopinavir plus ritonavir, NA: not available/not applicable.

**Table 7:** Frequency of adverse effects of CQ or HCQ alone when treating COVID-19 reported in observational and randomised studies.

First Author, Year	Type of Study, Drug and Sample Size	Cardiac						Gastrointestinal Disorders			Hepatobiliary		Neurological	Sense Organs	Dermatological	Other
		QT Prolongation	Prolonged QTc ≥ 500 ms	Prolongued QTc ≥ 60 ms	Ventricular Arrythmia	Torsade Depointes	Arrhythmogenic Death	Diarrhea	Nausea/Vomiting	Abdominal Pain/Dyspepsia	Bilirubin/GGT Increase	Transaminase Increase	Headache/Dizziness			
Seyhan AU, 2020 [241]	OBS HCQ *n* = 51	NA	1.96%	1.96%	0%	NA	NA	NA	NA	NA	NA	NA	NA	NA	NA	NA
Abella BS, 2021 [270]	RAN HCQ *n* = 132	NA	NA	NA	NA	NA	NA	32%	9%	6%	NA	NA	Headache 0% Dizziness 2%	NA	Rash 5%	Paraesthesia 2%
Bernardini A, 2021 [246]	OBS HCQ *n* = 40	40%	0%	NA	NA	NA	NA	NA	NA	NA	NA	NA	NA	NA	NA	NA
Furtado RHM, 2020 [247]	RAN HCQ *n* = 183	21%	NA	NA	CRVA 3%	NA	0%	NA	NA	24%	3%	NA	NA	NA	NA	NA
Satlin MJ, 2020 [250]	OBS HCQ *n* = 153	NA	NA	NA	Monomorphic VT 0.6%	0%	NA	NA	NA	NA	NA	Grade 3 11% Grade 4 9%	NA	NA	NA	NA
Hsia BC, 2020 [253]	OBS HCQ *n* = 40	NA	NA	NA	NA	0%	0%	NA	NA	NA	NA	NA	NA	NA	NA	NA
	OBS CQ *n* = 5	NA	NA	NA	NA	0%	0%	NA	NA	NA	NA	NA	NA	NA	NA	NA
Skipper CP, 2020 [252]	RAN HCQ *n* = 212	NA	NA	NA	NA	NA	NA	23.6%	31.1%	NA	NA	NA	Headache 0.9% Dizziness 9.4%	Ringing in ears 3.8% Changes in vision 1.9 Taste, dry mouth 0%	Rash 2.8	NA
Boulware DR, 2020 [271]	RAN HCQ *n* = 414	NA	NA	NA	NA	NA	NA	NA	22.9%	23.2%	NA	NA	3.7%	Tinnitus 2.3% Visual changes 0.9% Taste change or dry mouth 0.9%	Skin reaction 1.1%	NA
Falcão F, 2020 [239]	OBS HCQ *n* = 20	10%	NA	NA	NA	NA	NA	5%	Nausea 10% Vomiting 5%	0%	Liver cholestasis 0%	10%	NA	Ocular disorders 0%	Skin and subcutaneous disorders 10%	NA
Sogut O, 2021 [240]	OBS HCQ *n* = 152	64.5%	0%	0%	NA	NA	0%	22.3%			NA	NA	16.4%	NA	Itching and redness 2.6%	NA
Mitjà O, 2021 [268]	RAN HCQ *n* = 1116	NA	NA	NA	NA	NA	NA	42.6%			NA	NA	21.7%	NA	NA	General disorder: myalgia, fatigue, malaise 8.6%
Barnabas RV, 2021 [269]	RAN HCQ *n* = 407	NA	NA	NA	NA	NA	NA	NA	3.4%	6.1%	NA	NA	Headache 1.2% Dizziness 1.5%	Taste change or dry mouth 0.2% Visual changes 1% Tinnitus 0%	Rash 2.7%	Fatigue 1%
Nagaraja BS, 2020 [272]	OBS HCQ *n* = 156	NA	NA	NA	NA	NA	NA	7.22%	Nausea 10.24% Vomiting 1.20%	7.22%	NA	NA	Headache 6% Dizziness 3.6%	Tinnitus 0.6% Transient visual blurring 2.4%	Hair fall 1.8% Oral ulcer 1.2% Itching 0.6%	Psychiatric 4.8% Nightmare 0.6% Nervousness 1.20% Fatigue, lethargyWeakness 7.2%
Özdemir IH, 2020 [256]	OBS HCQ *n* = 45	NA	NA	NA	NSVT 0% SVT 0% VF 0%	0%	0%	NA	NA	NA	NA	NA	NA	NA	NA	NA
Cavalcanti AB, 2021 [257]	RAN HCQ *n* = 221	14.6%	NA	NA	VT 0%	NA	NA	NA	Nausea 4.5% Vomiting 0%	NA	2.5%	8.5%	NA	Hypoacusia 0%	Itching 0.5%	Hypoglycaemia 0.5%
Çap M, 2020 [258]	OBS HCQ *n* = 66	6%	NA	NA	NA	0%	0%	NA	NA	NA	NA	NA	NA	NA	NA	NA
Lauriola M, 2020 [259]	OBS HCQ *n* = 17	NA	NA	NA	NA	NA	0%	NA	NA	NA	NA	NA	NA	NA	NA	NA
Ramireddy A, 2020 [264]	OBS HCQ *n* = 10	NA	NA	NA	NA	0%	0%	NA	NA	NA	NA	NA	NA	NA	NA	NA
Tanriverdİ E, 2021 [260]	OBS HCQ *n* = 30	NA	NA	NA	0%	0%	0%	NA	NA	NA	NA	NA	NA	NA	NA	NA
Arshad S, 2020 [261]	OBS HCQ *n* = 1202	NA	NA	NA	NA	0%	NA	NA	NA	NA	NA	NA	NA	NA	NA	NA
Pereira MR, 2020 [265]	OBS HCQ *n* = NA	0%	NA	NA	0%	0%	0%	NA	NA	NA	NA	NA	NA	NA	NA	NA
Jain S, 2020 [230]	OBS HCQ *n* = 415	23.6%	NA	NA	NA	0%	0%	NA	NA	NA	NA	NA	NA	NA	NA	NA
Hor CP, 2020 [228]	OBS HCQ *n* = 2	100%	NA	NA	0%	0%	0%	NA	NA	NA	NA	NA	NA	NA	NA	NA
Paccoud O, 2020 [227]	OBS HCQ *n* = 38	5.3%	NA	NA	NA	NA	NA	2.6%	NA	NA	NA	NA	Headache 2.6%	NA	NA	NA
Lagier JC, 2020 [226]	OBS HCQ *n* = 101	NA	NA	2%	NA	0%	0%	NA	NA	NA	NA	NA	NA	NA	NA	NA
Reis G, 2021 [233]	RAN HCQ *n* = 207	NA	NA	NA	NA	NA	0%	NA	NA	NA	NA	NA	NA	NA	NA	NA
Faruqui AR, 2021 [267]	OBS HCQ *n* = 1303 (HCQ + AZ: 0.8%; CQ: 0.5%)	NA	NA	NA	NA	NA	NA	NA	Nausea 8.7% Vomiting 1.4%	7.0%	NA	NA	NA	Photosensitivity 0.5%	NA	NA
Eftekhar SP, 2021 [236]	OBS HCQ *n* = 29	10.3%	NA	NA	0%	3.4%	NA	NA	NA	NA	NA	NA	NA	NA	NA	NA
Mazzanti A, 2020 [229]	OBS HCQ *n* = 50	NA	NA	NA	NA	0%	0%	NA	NA	NA	NA	NA	NA	NA	NA	NA
Karolyi M, 2021 [231]	OBS CQ *n* = 20	NA	NA	NA	NA	NA	NA	0%	5%	NA	NA	10%	NA	NA	NA	NA
Mitjà O, 2020 [232]	RAN HCQ *n* = 136	NA	NA	NA	NA	NA	0%	88.1%			NA	NA	37.5%	Ear and labyrinth disorders 3% Eye disorders 3%	6.5%	Psychiatric disorders 1.2%
Lofgren SM, 2020 [266]	RAN HCQ once-daily *n* = 576	NA	NA	NA	NA	NA	0%	Upset stomach or nausea 25.3% Diarrhea, vomiting, or abdominal pain 22.7%			NA	NA	Headache 2.6% Irritability, dizziness, vertigo 6.8%	Tinnitus 2.8% Visual changes 1.2% Taste change or dry mouth 0.5%	Skin reaction 1.7%	Panic 0%
	RAN HCQ once-weekly *n* = 473	NA	NA	NA	NA	NA	0%	Upset stomach or nausea 17.5% Diarrhea, vomiting, or abdominal pain 12.9%			NA	NA	Irritability, dizziness, vertigo 5.7%	Tinnitus 2.1% Visual changes 1.5%	Skin reaction 2.7%	Sleep disturbance 2.1%
	RAN HCQ twice-weekly *n* = 463	NA	NA	NA	NA	NA	0%	Upset stomach or nausea 19.4% Diarrhea, vomiting, or abdominal pain 17.1%			NA	NA	Irritability, dizziness, vertigo 5.2%	Tinnitus 1.5% Visual changes 0.9%	Skin reaction 5.0%	Sleep disturbance 1.5%
Bessière F, 2020 [207]	OBS HCQ *n* = 22	NA	5%	NA	NA	0%	NA	NA	NA	NA	NA	NA	NA	NA	NA	NA
Mercuro NJ, 2020 [215]	OBS HCQ *n* = 37	NA	19%	8%	NA	0%	0%	NA	NA	NA	NA	NA	NA	NA	NA	NA
Rosenberg ES, 2020 [218]	OBS HCQ *n* = 271	14.4%	NA	NA	NA	NA	NA	17.0%	NA	NA	NA	NA	NA	NA	NA	NA
Saleh M, 2020 [219]	OBS HCQ or CQ *n* = 82	NA	8.5%	NA	NA	0%	0%	NA	NA	NA	NA	NA	NA	NA	NA	NA
Van den Broek MPH 2020 [220]	RAN CQ *n* = 95	NA	23%	NA	NA	0%	NA	NA	NA	NA	NA	NA	NA	NA	NA	NA
Chen Z, 2020 [222]	RAN HCQ *n* = 31	NA	NA	NA	NA	NA	NA	NA	NA	NA	NA	NA	Headache 3.2%	NA	3.2%	NA
Huang M, 2020 [223]	RAN CQ *n* = 10	NA	NA	NA	NA	NA	NA	50.0%	Nausea 40% Vomiting 50%	10%	NA	NA	Headache 0% Dizziness 0%	NA	10%	Psychosis 0%
Fernández-Ruiz M, 2020 [212]	OBS HCQ *n* = 4	NA	25%	NA	NA	0%	0%	NA	NA	NA	NA	NA	NA	NA	NA	NA
Chen J, 2020 [224]	OBS HCQ *n* = 15	NA	NA	NA	NA	NA	NA	13.3%	NA	NA	6.66%	NA	NA	NA	NA	Weakness 6.6%

AEs: adverse effects, AF: atrial fibrillation, BBB: bundle branch block, CRVA: clinically rellevant ventricular arryhtmia, COVID-19: coronavirus disease 2019, CQ: chloroquine, ECG: electrocardiograph, HCQ: hydroxychloroquine, ms: milliseconds, NA: not available/not applicable, NSVT: non-sustained ventricular tachycardia, OBS: observational, QTc: corrected QT interval, RAN: randomised, SVT: sustained ventricular tachycardia, SVT: supraventricular tachycardia, VF: ventricula fibrillation, VT: ventricular tachycardia.

**Table 8:** Adverse effects of CQ or HCQ in combination with other drugs used to treat COVID-19 reported in observational and randomised studies.

First Author, Year	Type of Study, Drug and Sample Size	Cardiac						Gastrointestinal			Hepatobiliary		Neurological	Sense Organs	Dermatological	Other
		QT Prolongation	Prolonged QTc ≥ 500 ms	Prolongued QTc ≥ 60 ms	Ventricular Arrythmia	Torsade de Pointes	Arrhythmogenic Death	Diarrhea	Nausea/Vomiting	Abdominal Pain/Dyspepsia/Other	Bilirubin Increase/GGT Increase	Transaminase Increase	Headache/Dizziness			
Gao X, 2020 [273]	OBS INF-alpha + LPVr + arbidol or rivabirin or CQ *n* = 26	NA	NA	NA	NA	NA	NA	11.5%	NA	NA	Abnormal liver function 61.5%	NA	NA	NA	Rash 7.7%	Dyslipidemia 42.3%
Seyhan AU, 2020 [241]	OBS HCQ + AZM *n* = 93	NA	1.07%	2.15%	0%	NA	NA	NA	NA	NA	NA	NA	NA	NA	NA	NA
Lamback EB, 2021 [237]	OBS HCQ + AZM *n* = 101	7.9%	NA	NA	NA	NA	NA	7.9%			NA	NA	NA	NA	NA	NA
Saleh M, 2020 [245]	OBS HCQ ± AZM *n* = 6.476 (HCQ *n* = 2847 HCQ + AZM *n* = 3629)	NA	NA	NA	VF 0.06% SMVT 0.08% NSMVT 0.27% SPVT 0.015% NSPVT 0%	0.015%	NA	NA	NA	NA	NA	NA	NA	NA	NA	NA
Bernardini A, 2021 [246]	OBS HCQ + AZM *n* = 53	70%	8%	NA	NA	NA	NA	NA	NA	NA	NA	NA	NA	NA	NA	NA
Furtado RHM, 2020 [247]	RAN HCQ + AZM *n* = 214	20%	NA	NA	CRVA 3%	NA	NA	NA	NA	25%	Bilirrubin increase > 50% 4%	NA	NA	NA	NA	NA
Giaime P, 2020 [248]	OBS HCQ + AZM *n* = 21	NA	4.8%	NA	NA	NA	NA	NA	19%	NA	NA	NA	NA	Visual impairment 0%	Dermatitis 0%	Hypoglicaemia 23.8%
Kalligeros M, 2020 [249]	OBS HCQ + AZM *n* = 32	3.1%	9.4%	NA	VC 0.9%	0%	NA	NA	NA	NA	NA	NA	NA	NA	NA	Seizure 3.1%
Kelly M, 2021 [276]	OBS HCQ + AZM *n* = 82	13.4%	NA	NA	NA	NA	NA	NA	NA	NA	NA	Elevated liver function tests 65%	NA	NA	NA	NA
Hsia BC, 2020 [253]	OBS HCQ + AZM *n* = 33	NA	NA	NA	NA	0%	0%	NA	NA	NA	NA	NA	NA	NA	NA	NA
	OBS CQ + AZM *n* = 4	NA	NA	NA	NA	0%	0%	NA	NA	NA	NA	NA	NA	NA	NA	NA
Moschini L, 2021 [251]	OBS HCQ + AZM *n* = 52	NA	13% (day 3), 20% (day 7)	NA	MVA 1.9%	NA	NA	NA	NA	NA	NA	NA	NA	NA	NA	NA
	OBS HCQ + DRVr *n* = 61	NA	NA	NA	MVA 1.6%	NA	NA	NA	NA	NA	NA	NA	NA	NA	NA	NA
O’Connell TF, 2021 [238]	OBS HCQ + AZM *n* = 415	NA	21%	NA	0%	NA	NA	NA	NA	NA	NA	NA	NA	NA	NA	NA
Falcão F, 2020 [239]	OBS HCQ + AZM *n* = 52	5.7%	NA	NA	NA	NA	NA	1.9%	Nausea 3.8% Vomiting 1.9%	3.8%	Bilirrubin increase 0% GGT increase 1.9% Liver cholestasis 7.7%	3.84% Hepatotoxicity 7.7%	NA	Ocular disorders 1.9%	Skin and subcutaneous disorders1.9%	NA
	OBS HCQ + LPVr *n* = 22	0%	NA	NA	NA	NA	NA	40.9%	Nausea 4.5% Vomiting 4.5%	4.5%	Bilirrubin increase 13.6% GGT increase 13.6% Liver cholestasis 4.5%	54.54% Hepatotoxicity 0%	NA	Ocular disorders 4.5%	Skin and subcutaneous disorders 0%	NA
	OBS HCQ + AZM + LPVr *n* = 7	14.2%	NA	NA	NA	NA	NA	71.4%	Nausea 14.2% Vomiting 0%	0%	Bilirrubin increase 14.3% GGT increase 14.2% Liver cholestasis 14.2%	42.8% Hepatotoxicity 14.2%	NA	Ocular disorders 0%	Skin and subcutaneous disorders0%	NA
Chen CP, 2020 [274]	RAN HCQ ± AMZ ± OSM ± LEV *n* = 21	0%	NA	NA	NA	NA	NA	5.3%	5.3%	Gastritis 5.3%	NA	NA	Headache 21.1% Dizziness 5.3%	Photophobia 5.3%	NA	NA
Meriglier E, 2021 [242]	OBS HCQ + LPVr *n* = 21	NA	NA	NA	NA	NA	NA	23.8%	9.52%	NA	NA	0%	Headache 0%	NA	0%	NA
	OBS HCQ + DRVr *n* = 25	NA	NA	NA	NA	NA	NA	32%	0%	NA	NA	4%	Headache 0%	NA	0%	NA
Self WH, 2020 [275]	RAN HCQ ± AZM ± REM ± corticosteroids *n* = 242	NA	5.9%	NA	2.1%	NA	NA	NA	NA	NA	NA	20.7%	NA	NA	NA	Seizure 0.4% Symptomatic hypoglycaemia 4.1%
Fteiha B, 2021 [243]	OBS HCQ± AZM *n* = 90	NA	7.8%	12%	NA	NA	NA	NA	NA	NA	NA	NA	NA	NA	NA	NA
RECOVERY Collaborative Group, 2020 [244]	RAN HCQ± AZM *n* = 1561	NA	NA	NA	VT or fibrillation 0.7%	0.064%	NA	NA	NA	NA	NA	NA	NA	NA	NA	NA
Nagaraja BS, 2020 [272]	OBS HCQ + AZM *n* = 7	NA	NA	NA	NA	NA	NA	28.6%			NA	NA	NA	NA	NA	NA
Echarte-Morales J, 2020 [254]	OBS HCQ + AZM *n* = 54	9.3%	3.7%	11.1%	NA	NA	0%	NA	NA	NA	NA	NA	NA	NA	NA	NA
	OBS HCQ + AZM + LPVr *n* = 114	11.4%	6.1%	18.4%	NA	NA	0%	NA	NA	NA	NA	NA	NA	NA	NA	NA
Jiménez-Jáimez J, 2020 [255]	OBS HCQ + AZM + LPVr or DRVr *n* = 114	NA	1.8%	NA	NA	0%	0%	NA	NA	NA	NA	NA	NA	NA	NA	NA
	OBS HCQ ± AZM *n* = 105	NA		NA	NA	0%	0%	NA	NA	NA	NA	NA	NA	NA	NA	NA
Özdemir IH, 2020 [256]	OBS HCQ + AZM *n* = 56	NA	NA	NA	NSVT 0% SVT 0%	0%	0%	NA	NA	NA	NA	NA	NA	NA	NA	NA
Cavalcanti AB, 2021 [257]	RAN HCQ + AZM *n* = 217	14.7%	NA	NA	VT 0%	NA	NA	NA	Nausea 2.5% Vomiting 0%	NA	Bilirrubin increase 0.4%	10.9%	NA	Hypoacusia 0%	Itching 0%	Hypoglycaemia 0%
Çap M, 2020 [258]	OBS HCQ + FVP *n* = 66	3%	NA	NA	NA	0%	0%	NA	NA	NA	NA	NA	NA	NA	NA	NA
Rodriguez-Garcia JL, 2020 [277]	OBS HCQ + LPVr + corticosteroids *n* = 50	NA	NA	NA	NA	NA	NA	NA	NA	NA	NA	NA	NA	NA	NA	Delirium 4% Hyperglycemic decompensation 10%
Ip A, 2020 [278]	OBS HCQ ± AZM (HCQ: *n* = 441 HCQ + AZM *n* = 1473)	NA	NA	NA	NA	NA	NA	NA	NA	NA	NA	NA	NA	NA	NA	NA
Lauriola M, 2020 [259]	OBS HCQ + AZM *n* = 297	NA	NA	NA	NA	NA	0%	NA	NA	NA	NA	NA	NA	NA	NA	NA
Tanriverdİ E, 2021 [260]	OBS HCQ + AZM *n* = 26	NA	NA	NA	0%	0%	0%	NA	NA	NA	NA	NA	NA	NA	NA	NA
Arshad S, 2020 [261]	OBS HCQ + AZM *n* = 783	NA	NA	NA	NA	0%	NA	NA	NA	NA	NA	NA	NA	NA	NA	NA
Bun SS, 2020 [262]	OBS HCQ + AZM *n* = 71	NA	2.8%	NA	NA	NA	0%	NA	NA	NA	NA	NA	NA	NA	NA	NA
Dastan F, 2020 [279]	RAN HCQ + INF-β-1a + LPVr *n* = 20	NA	NA	NA	NA	NA	0%	NA	NA	NA	0%	0%	NA	NA	NA	NA
Maraj I, 2020 [263]	OBS HCQ + AZM *n* = 91	23%	14%	NA	2.2%	NA	NA	NA	NA	NA	NA	NA	NA	NA	NA	NA
Ramireddy A, 2020 [264]	OBS HCQ + AZM *n* = 61	11.4%	NA	NA	NA	0%	0%	NA	NA	NA	NA	NA	NA	NA	NA	NA
Pereira MR, 2020 [265]	OBS HCQ + AZM *n* = NA	0%	NA	NA	0%	0%	0%	NA	NA	NA	NA	NA	NA	NA	NA	NA
Uğurlu Ilgin B, 2021 [235]	OBS HCQ + OSM + AZM *n* = 43	42.85%	8.8%	NA	0%	0%	0%	NA	NA	NA	NA	NA	NA	NA	NA	NA
	OBS HCQ + OSM + LEV *n* = 48			NA	0%	0%	0%	NA	NA	NA	NA	NA	NA	NA	NA	NA
Hor CP, 2020 [228]	OBS HCQ + AZM *n* = 11	NA	NA	9.1%	0%	0%	0%	NA	NA	NA	NA	NA	NA	NA	NA	NA
Lagier JC, 2020 [226]	OBS HCQ + AZM ≥ 3 days *n* = 3119 HCQ + AZM < 3 days *n* = 218	NA	0.03%	0.6%	NA	0%	0%	NA	NA	NA	NA	NA	NA	NA	NA	NA
Dabbous HM, 2021 [234]	OBS HCQ + OSM *n* = 50	NA	NA	NA	NA	NA	NA	NA	NA	NA	NA	0%	NA	NA	NA	NA
Eftekhar SP, 2021 [236]	OBS HCQ + AZM *n* = 143	24.5%	NA	NA	1.4%	0.7%	NA	NA	NA	NA	NA	NA	NA	NA	NA	NA
Mazzanti A, 2020 [229]	OBS HCQ + AZM *n* = 39	NA	NA	NA	NA	0%	0%	NA	NA	NA	NA	NA	NA	NA	NA	NA
	OBS HCQ + LPVr *n* = 53	NA	NA	NA	NA	0%	0%	NA	NA	NA	NA	NA	NA	NA	NA	NA
	OBS HCQ + LPVr + AZM *n* = 9	NA	NA	NA	NA	0%	0%	NA	NA	NA	NA	NA	NA	NA	NA	NA
Borba MGS, 2020 [221]	RAN CQ low dose + AZM ± OSM *n* = 40	NA	7.9%	0%	NA	NA	NA	NA	NA	NA	NA	NA	NA	NA	NA	NA
	RAN CQ high dose + AZM ± OSM *n* = 41	NA	21.2%	5.8%	NA	NA	NA	NA	NA	NA	NA	NA	NA	NA	NA	NA
Tang W, 2020 [211]	RAN HCQ + SOC *n* = 70	NA	NA	NA	NA	NA	NA	10%	NA	NA	NA	NA	NA	NA	NA	NA
Chong VH, 2020 [208]	OBS HCQ + LPVr *n* = 11	NA	18.2%	NA	NA	0%	0%	NA	NA	NA	NA	NA	NA	NA	NA	NA
Fernández-Ruiz M, 2020 [212]	OBS HCQ + LPVr *n* = 6	NA	NA	NA	NA	NA	NA	NA	NA	16.7%	NA	NA	NA	NA	NA	NA
Colaneri M, 2020 [225]	OBS HCQ + AZM + TCZ *n* = 21	NA	NA	NA	NA	NA	0%	NA	NA	NA	NA	NA	NA	NA	NA	NA
Saleh M, 2020 [245]	OBS CQ or HCQ + AZM *n* = 119	NA	9.2%	NA	NA	0%	0%	NA	NA	NA	NA	NA	NA	NA	NA	NA
Rosenberg ES, 2020 [218]	OBS HCQ + AZM *n* = 735	11.0%	NA	NA	NA	NA	NA	11.6%	NA	NA	NA	NA	NA	NA	NA	NA
Molina JM, 2020 [217]	OBS HCQ + AZM *n* = 11	9.09%	NA	NA	NA	NA	NA	NA	NA	NA	NA	NA	NA	NA	NA	NA
Million M, 2020 [216]	OBS HCQ + AZM *n* = 1061	NA	0%	0.8%	NA	0%	0%	1.1%	Nausea 0.2% Vomiting 0.1%	Abdominal pain 0.3%	NA	NA	Headache 0.3%	Transient blurred vision 0.2%	Erythematous and bullous rash 0.1%	Insomnia 0.2%
Bessière F, 2020 [207]	OBS HCQ + AZM *n* = 18	NA	33%	NA	NA	0%	NA	NA	NA	NA	NA	NA	NA	NA	NA	NA
Chorin E, 2020 [209]	OBS HCQ + AZM *n* = 251	NA	13%	NA	NA	0.4%	NA	NA	NA	NA	NA	NA	NA	NA	NA	NA
Cipriani A, 2020 [210]	OBS HCQ + AZM *n* = 22	NA	4.54%	18%	4.54%	NA	0%	NA	NA	NA	NA	NA	NA	NA	NA	NA
Gautret P, 2020 [213]	OBS HCQ + AZM *n* = 80	NA	NA	NA	NA	NA	NA	5.0%	2.5%	NA	NA	NA	NA	Blurred vision 1.2%	NA	NA
Mahévas M, 2020 [214]	OBS HCQ ± AZM *n* = 84	NA	1.2%	8.3%	NA	NA	NA	NA	NA	NA	NA	NA	NA	NA	NA	NA
Mercuro NJ, 2020 [215]	OBS HCQ + AZM *n* = 53	NA	21%	13%	NA	1.88%	NA	NA	NA	NA	NA	NA	NA	NA	NA	NA

AF: atrial fibrillation, AVB: atrioventricular block, AZM: azithromycin, BBB: bundle branch block, COVID-19: coronavirus disease 2019, CQ: chloroquine, CRVA: clinically rellevant ventricular arryhtmia, DRVr: darunavir/ritonavir, ECG: electrocardiograph, GGT: gamma-glutamyl transferase, HCQ: hydroxychloroquine, INF-β: interferon-beta, LEV: levofloxacin, LPVr: lopinavir/ritonavir, MVA: malignant ventricular arryhtmia, NA: not available/not applicable, NSMVT: non-sustained monomorphic ventricular tachycardia, NSPVT: non-sustained polymorphic ventricular tachycardia, NSVT: non-sustained ventricular tachycardia, ms: milliseconds, OBS: observational, OSM: oseltamivir, QTc: corrected QT interval, RAN: randomised, REM: remdesivir, SMVT: sustained monomorphic ventricular tachycardia, SOC: standard of care, SPVT: sustained polimorphic ventricular tachycardia, SVT: sustained ventricular tachycardia, SVT: supraventricular tachycardia, TCZ: tocilizumab, VC: ventricular contractions, VF: ventricular fibrillation, VT: ventricular tachycardia.

### 3.2. Quality Assessment

For case series and case reports, overall CARE Checklist scores ranged from 34% to 100%. Only two studies could not be assessed because more than 50% of the checklist items were judged “Not applicable”, one of them reporting a case of acute psychosis after CQ administration and the other reporting a case of acute generalised exanthematous pustulosis with HCQ [47,66]. A total of 73 studies were rated as high quality, 40 as moderate quality, and 11 as low quality. Although 11 articles were rated as having low reporting quality, the adverse effects were clearly described in all cases, so this did not affect their inclusion in the qualitative synthesis of the results. A total of 84 observational studies were assessed using the combined STROBE checklist. A total of 47 studies were rated as high quality, 29 as moderate quality, and 6 as low quality. The quality of 2 other studies could not be assessed because more than 50% of the checklist items were “Not applicable”. In the case of the randomised studies, 46 articles including 49 clinical trials were assessed using the JADAD scale. A total of 16 studies received scores less than 3, whereas 33 studies received scores greater than or equal to 3.

### 3.3. Data Synthesis of the Systematic Review Findings

The study data on the frequency of adverse events was quantitatively synthesised and is reported as a range of percentages ordered by indication and drug combination in Table 9. Table 5 shows the adverse effects reported in the case-control studies as well as those that could not be added to the data synthesis.

#### 3.3.1. Patients Treated for Conditions Other Than COVID-19

Of the 47 studies reporting data on CQ or HCQ alone, 31 provided data that could be added to the quantitative data synthesis [126,127,128,129,130,131,132,133,134,135,136,137,138,139,140,141,142,143,144,145,146,147,148,149,150,151,152,153,154,155,156,157,158,159,160,161,162,163,164,165,166], but in the case of 15 others, the frequency could not be calculated so they were not included [167,168,169,170,171,172,173,174,175,176,177,178,179,180,181]. The data from one additional study reported data from pregnant patients and was likewise excluded from the quantitative synthesis [172]. Of the nine studies reporting data on CQ or HCQ combined with other drugs, three reported data that could be added to the quantitative data synthesis [146,173,174], whereas four others did not report adverse event frequency [175,176,177,178], and two reported data from pregnant patients [179,180]. One article reported data that could be added to our synthesis both for CQ alone and for CQ plus AZM [146].

#### 3.3.2. Patients Treated for COVID-19

Out of a total of 74 studies, 66 provided data that could be added to the quantitative synthesis [207,208,209,210,211,212,213,214,215,216,217,218,219,220,221,222,223,224,225,226,227,228,229,230,231,232,233,234,235,236,237,238,239,240,241,242,243,244,245,246,247,248,249,250,251,252,253,254,255,256,257,258,259,260,261,262,263,264,265,266,267,268,269,270,271,272]. Seven of these studies contained data on COVID-19 prophylactic treatments [266,267,268,269,270,271,272]. The remaining studies lacked information about these drug combinations (e.g., they were the only studies reporting data in this specific combination) or reported data with which the frequency could not be calculated and were therefore not included in the quantitative synthesis [273,274,275,276,277,278,279,280].

**Table 9:** Systematic review findings synthesised by the frequency of adverse events reported as a range of percentages.

Treatment Indication	Malaria Treatment and Prophylaxis	Autoimmune Diseases	Porphyria Cutanea Tarda	Malaria Treatment and Prophylaxis	COVID-19 Prophylaxis	COVID-19				
Drug	CQ	CQ/HCQ	CQ/HCQ	CQ plus AZM	HCQ	CQ/HCQ	CQ/HCQ + AZM	HCQ + LPVr	HCQ + DRVr	HCQ + AZM + LPVr
Cardiac adverse events										
Arrhythmia	NA	NA	NA	NA	0–0.2% (1)	0–16.2% (3)	0–20.4% (3)	NA	NA	NA
Palpitations	NA	NA	NA	0–2.6% (2)	0.4–2.4% (3)	NA	NA	NA	NA	NA
Cardiac arrest	NA	NA	NA	NA	NA	0–13.7% (3)	7–15.5% (2)	NA	NA	NA
ECG changes	0% (1)	NA	NA	NA	NA	0–27.3% (2)	0–27.1% (2)	19.0% (1)	0–16% (1)	NA
Prolonged QTc ≥500 ms	NA	NA	NA	NA	NA	0–25% (8)	0–33% (18)	18.2% (1)	NA	6.1% (1)
QTc change ≥60 ms	NA	NA	NA	NA	NA	0–8% (4)	0–18% (9)	NA	NA	18.4% (1)
Torsade de pointes	NA	NA	NA	NA	NA	0–3.4% (19)	0–1.88% (19)	0% (2)	0% (1)	0% (2)
Arrhythmogenic deaths	NA	NA	NA	NA	0% (1)	0% (19)	0% (18)	0% (2)	0% (1)	0% (3)
Dermatological adverse events										
Cutaneous Drug Eruptions	2.9% (1)	4.0–6.4% (4)	NA	NA	0.6–5% (5)	0.6–10.0% (9)	0–1.9% (4)	0% (2)	0% (1)	0% (1)
Exanthema	0% (1)	1.6% (1)	NA	NA	NA	NA	NA	0% (1)	0% (1)	0% (1)
Pruritus	3.3–64.5% (12)	2.2% (1)	NA	2.0–50.9% (6)	NA	NA	NA	0% (1)	0% (1)	0% (1)
Gastrointestinal adverse events										
Diarrhea	3.8–20.0% (4)	6.4% (1)	NA	0–12.0% (6)	7.2–32% (2)	0–50% (7)	1.1–11.6% (4)	23.8–40.9% (2)	32% (1)	71.4% (1)
Anorexia	0.9–16.7% (3)	NA	NA	NA	4.8% (1)	NA	NA	NA	NA	NA
Nausea	0–22.0% (6)	NA	20.8% (1)	0–30.0% (6)	3.4–25.3% (4)	4.5–40% (3)	0.2–3.8% (3)	4.5–9.5% (2)	0% (1)	14.2% (1)
Vomiting	0–31.1% (9)	NA	12.5% (1)	1.0–18.0% (6)	1.2–1.4% (2)	0–50% (3)	0–1.9% (3)	4.5% (1)	0% (1)	0% (1)
Abdominal pain or discomfort, dyspepsia or GI intolerance	4.7–30.0% (4)	3.2–4.3% (3)	20.8% (1)	0–11.5% (5)	6–23.2% (5)	0–24% (3)	0.3–25.0% (3)	4.5–16.7% (2)	NA	0% (1)
Psychiatric and neurological adverse events										
Anxiety/nervousness	NA	4.3% (1)	NA	NA	0.6–1.2% (1)	NA	NA	NA	NA	NA
Insomnia/Sleep disturbances	NA	NA	NA	NA	1.5–2.1% (1)	NA	0.2% (1)	NA	NA	NA
Psychosis	0% (1)	NA	NA	NA	NA	0% (1)	NA	NA	NA	NA
Dizziness	0.3–43.1% (5)	3.2% (1)	NA	0–15.9% (3)	1.5–3.6% (3)	0–9.4% (2)	NA	NA	NA	NA
Headache	0.3–25.0% (6)	NA	25.0% (1)	0–17.7% (2)	0–6% (4)	0–3.2% (4)	0.3% (1)	0% (1)	0% (1)	NA
Paraesthesia	NA	NA	NA	0–3.0% (3)	2% (1)	NA	NA	NA	NA	NA
Hematologic and metabolic adverse events										
Thrombocytopaenia	NA	NA	NA	NA	NA	0–7% (3)	0–7.1% (3)	4.5–9.1% (2)	NA	0% (1)
Hypoglycaemia	NA	NA	NA	NA	1.1% (1)	0.5% (1)	0–23.8% (2)	0 (1)	0% (1)	NA
Sense organs adverse events										
Blurred vision	NA	NA	NA	NA	NA	NA	0.2–1.2% (2)	NA	NA	NA
Tinnitus	0.7–9.4% (2)	NA	NA	NA	0–2.8% (4)	3.8% (1)	0% (1)	NA	NA	NA
Visual disturbances/ocular disorders	1.3% (1)	3.2% (1)	NA	NA	0.9–2.4% (4)	0–3% (3)	0–1.9% (2)	4.5% (1)	NA	0% (1)
Hepatic adverse events										
Hepatomegaly	NA	NA	2.8% (1)	NA	NA	NA	NA	NA	NA	NA
Icterus/Bilirubin or GGT increase	0% (1)	NA	1.4% (1)	NA	NA	0–6.6% (4)	0–4% (3)	NA	NA	14.3% (1)
Transaminase increase	0–0.43% (2)	NA	95.8–100.0% (2)	NA	NA	0–11% (5)	3.8–10.9% (2)	0–54.5% (3)	4% (1)	42.8% (1)
Other adverse events										
Asthenia/Weakness	20.0–47.1% (2)	NA	NA	5.3–8.0% (2)	7.2% (1)	6.6% (1)	NA	NA	NA	NA
Fatigue	0.4–1.9% (2)	NA	13.9% (1)	0–3.5% (3)	1% (1)	NA	NA	NA	NA	NA
Fever	15.0% (1)	NA	37.5–43.8% (2)	NA	NA	NA	NA	NA	NA	NA
Myalgia	0% (1)	NA	26.4% (1)	NA	NA	NA	NA	NA	NA	NA

AZM: azithromycin; CQ: chloroquine; DRVr: darunavir plus ritonavir; ECG: electrocardiogram; GGT: gamma-glutamyl transferase; GI: gastrointestinal; HCQ: hydroxychloroquine; LPVr: lopinavir plus ritonavir; NA: not available/not applicable; QTc: corrected QT interval.

### 3.4. Summary of the Evidence across Studies

#### 3.4.1. Cardiac Adverse Drug Reactions

##### Cases

Cases of a complete heart block, an implanted pacemaker failure, and a QT-interval prolongation were described in patients treated with HCQ for autoimmune conditions [97,98,100], and cases of cardiovascular collapse, non-specified cardiac arrhythmia, and syncopal attacks with torsade de pointes were described in patients treated with CQ for malaria, amoebiasis, and a dermatological problem [95,96,99]. In patients being treated for COVID-19, six cases of cardiac adverse effects with QT interval prolongation were described, consisting of a case of QT interval prolongation and recurrent torsade de pointes with CQ [188], a case of right bundle branch block and critical QT interval prolongation with HCQ [181], a case of torsade de pointes in a patient treated with HCQ plus dexamethasone [190], a case of suspected HCQ-induced sinus bradycardia and QT interval prolongation [191], a case of QT prolongation in a patient treated with HCQ plus AZM [192], and a case of death due to progressive metabolic acidosis and multiple organ system failure in a patient being treated with HCQ plus AZM [186]. Additionally, a case of death from cardiac arrest in a patient who developed wide complex tachycardia during CQ plus AZM treatment [189], and a case of sinus bradycardia with HCQ plus AZM were reported [192].

##### Observational and Randomised Studies

Studies including patients treated with CQ or HCQ for malaria, autoimmune conditions, or porphyria cutanea tarda (PCT) did not report any cases of cardiac adverse effects. One retrospective cohort study assessing cases of cardiac symptoms, cardiac arrest, and ventricular arrhythmias in patients treated with CQ plus AZM for autoimmune diseases did not find significant differences in these events in comparison with amoxicillin treatment [177]. One study assessing cases of torsade de pointes, QT prolongation, and death in patients treated with CQ or HCQ plus AZM for diverse pathologies did not find any potential safety concerns for HCQ or CQ alone. Conversely, this study found a significant safety risk for torsade de pointes and QT prolongation when AZM was used alone [178]. In two randomised studies on the combination of CQ plus AZM for malaria, only three cases of palpitations were identified after evaluating 227 patients [173]. However, this was not observed in four other studies, which did not describe electrocardiographic evaluations [146,174,179,180]. In subjects with COVID-19, cardiac adverse drug reactions were the most common adverse drug reactions reported. Prolongation of the corrected QT interval ≥500 ms was observed in 0–25% of subjects treated with HCQ or CQ alone [207,212,215,219,220,240,241,246], 0–33% of subjects treated with HCQ or CQ plus AZM [207,209,210,214,215,216,221,226,238,241,245,246,248,249,251,254,262,263], 18.2% of subjects treated with HCQ plus LPVr [208], and 6.1% of subjects treated with HCQ plus AZM plus LPVr [254]. Prolongation of the corrected QT interval ≥60 ms was observed in 0–8% of subjects treated with HCQ alone [215,226,240,241], in 0–18% of subjects treated with HCQ plus AZM [210,214,215,216,221,226,228,241,254], and in 18.4% of subjects treated with HCQ plus AZM plus LPVr [254]. Torsade de pointes was only observed in 5 out of 15,039 patients in CQ or HCQ plus AZM COVID-19 studies [207,209,212,215,216,219,220,226,228,230,235,236,245,249,250,253,255,256,258,260,261,264,265]. No subjects in studies on HCQ plus LPVr or DRVr, or HCQ plus AZM plus LPVr or DRVr developed torsade de pointes [207,208,215,219,220,229,255]. Arrhythmogenic deaths were not reported in any study. The discontinuation of CQ or HCQ due to cardiac adverse drug reactions was observed in 2.4% of patients treated with HCQ or CQ alone [219], in 0–9.5% of patients treated with CQ or HCQ plus AZM [212,214,216,217,219], and in 0–36.4% of patients treated with HCQ plus LPVr [208,212].

#### 3.4.2. Dermatological Adverse Drug Reactions

##### Cases

A total of 41 cases of mild to severe dermatological adverse effects, including 2 cases of fatal toxic epidermal necrolysis, were described in patients treated with HCQ for autoimmune diseases [31,34,35,36,38,40,41,42,43,44,45,46,47,48,50,51,52,53,54,55,56,57,59,60,62,63,64,65]. A smaller number of cases were reported in patients treated with CQ for autoimmune conditions or malaria although one fatal case was reported [27,28,29,30,32,33,37,39,49,54,58,61]. In connection with treatment for COVID-19, 12 cases were reported in patients treated with HCQ including cases of psoriasis exacerbation, rash, acute generalised exanthematous pustulosis (AGEP), drug rash with eosinophilia and systemic symptoms (DRESS), urticaria, palmoplantar itching, and purpuric erythematous rashes [185,187,196,197,198,199,200,201,202].

##### Observational and Randomised Studies

Pruritus has been described as occurring with high frequency (2–64.5%) in black African patients treated with CQ (with or without AZM) for malaria [138,139,140,170,171]. Two studies revealed a favourable effect of prednisolone to prevent pruritus without reporting other adverse effects [160,176]. Severe reactions were less often described in patients treated with CQ for malaria or autoimmune conditions. Erythema, exanthema, and maculopapular and vesiculopapular rashes in patients treated with CQ for malaria [140]; cutaneous drug eruptions, erythema, urticaria, and macular and papular exanthemas in patients treated with CQ or HCQ for SLE; and cutaneous lupus erythematosus (CLE) and dermatomyositis [132,133,134], and a generalised maculopapular rash in one patient treated with CQ for pulmonary sarcoidosis were described [145], with frequencies ranging from 0% to 6.4%. In COVID-19-affected patients, moderate to severe skin reactions were described in 0% to 10.0% of subjects although the highest frequency corresponded to a study that only included 10 patients [216,222,223,232,239,240,248,252,257]. We did not find increases in skin adverse effects when HCQ or CQ were combined with AZM, DRVr, or LPVr [239,242]. In the majority of the studies included that referred to patients with malaria, PCT, or COVID-19, there was no reference at all to dermatological toxicities.

#### 3.4.3. Neurologic and Psychiatric Adverse Drug Reactions

##### Cases

A broad spectrum of neurological and psychiatric events was described in patients treated with CQ for malaria, amoebiasis, arthritis, acute myocardial infarction, erythema nodosum leprosum, or COVID-19 [66,67,68,69,70,71,72,73,74,75,76,77,78,79,80,81,82,83,84,85,86,87,88,89,90,91,92,125,193,195]. Nevertheless, in the case of treatment with HCQ, only one case of psychomotor agitation in a patient with RA and one case of psychosis in a patient with SLE were reported [74,93].

##### Observational and Randomised Studies

Anxiety was reported in one patient treated with CQ in a study on pulmonary sarcoidosis [145], and anxiety and nervousness in patients treated with HCQ for COVID-19 prophylaxis [272]. Insomnia was reported in 0.18% of patients with HCQ plus AZM for COVID-19 [216], and sleep disturbances were reported in patients treated with HCQ for COVID-19 prophylaxis [266,272]. Dizziness was reported in 3.2% of patients with SLE treated with HCQ [133], 0.3–19% of patients with malaria treated with CQ [126,146,171], 0–15.9% of patients treated with CQ plus AZM for malaria [140,173], 1.5–3.6% of patients treated for COVID-19 prophylaxis [266,269,270,272], 9.4% of patients treated with HCQ for COVID-19 [252], and 0–0.3% of patients treated with HCQ plus AZM for COVID-19. Headache was reported in 0.3–25% of patients with malaria treated with CQ [126,138,140,142,171], 0–17.7% of patients treated with CQ plus AZM for malaria [173,174], 25% of patients with PCT treated with HCQ [135], 0–3.2% of patients treated with CQ or HCQ [222,223,227,252], and 0.28% for patients treated with HCQ plus AZM for COVID-19 [216], but was not reported in patients with autoimmune conditions. Paraesthesia was reported in 0–3% of patients treated with CQ plus AZM for malaria [174] and 2% of patients treated with HCQ for COVID-19 prophylaxis [270], but not reported in other conditions. One study assessing cases of depression as well as accidents/injuries in patients treated with CQ or HCQ plus AZM for diverse pathologies did not find potentially meaningful pharmacovigilance signs for CQ or HCQ, either alone or in combination with AZM [178]. However, a pharmacovigilance analysis suggested that COVID-19 patients exposed to HCQ could suffer psychiatric disorders and that HCQ was associated with an increased risk of reporting psychiatric disorders compared with other treatments [280]. Psychosis was not observed in patients treated with CQ for malaria nor in patients with COVID-19 [126,223] and was not reported in the rest of the studies.

#### 3.4.4. Gastrointestinal and Hepatic Adverse Drug Reactions

##### Cases

Prior to COVID-19, five cases of liver injury were reported, one involving treatment with CQ and four involving HCQ, including one fatal case [111,112,113,114,115]. One case of hepatotoxicity in a COVID-19-affected patient treated with HCQ was also reported [182].

##### Observational and Randomised Studies

Nausea and vomiting affected 0–11.5% of patients treated with CQ for malaria [130,138,140,141,146,171,172], 12.5–20.8% of patients treated with CQ or HCQ for PCT [135], 0–30% of patients treated with CQ plus AZM for malaria [146,173,174], 40–50% of patients treated with CQ for COVID-19 (data from a small study of 10 patients) [223], 0–31.3% of patients treated with HCQ for COVID-19 [239,252,257], 0–19% of patients treated with HCQ plus AZM for COVID-19 [213,216,231,239,248,257], 4.5–9.5% of patients treated with HCQ plus LPVr for COVID-19 [239,242], and 0–14.2% of patients treated with HCQ plus AZM plus LPVr for COVID-19 [239], Diarrhea was reported in 3.8–8.3% of patients treated for malaria with CQ [138,146,171], 6.4% of patients treated with HCQ for autoimmune conditions [133], and 0–12% of patients treated with CQ plus AZM for malaria [133,146,173,174,179,180]. In the case of COVID-19 patients, diarrhea was observed in 50% of patients treated with CQ [223], 0–23.6% of patients treated with HCQ [218,224,227,231,239,252], 1.1–11.6% of patients treated with HCQ plus AZM [213,216,218,239], 23.8–40.9% of patients treated with HCQ plus LPVr [239,242], 32% of patients treated with HCQ plus DRVr [242], 71.4% of patients treated with HCQ plus AZM plus LPVr [239], and 10% of patients treated with HCQ plus standard of care (SOC) including antiviral agents, antibiotics, or systemic glucocorticoids [211]. Abdominal discomfort or pain or other gastrointestinal discomforts were reported in 4.7–30.0% of patients treated with CQ for malaria [126,138,142,171], 3.2–4.3% of patients treated with CQ or HCQ for autoimmune conditions [133,145], 20.0% of patients treated with HCQ for PCT [135], 0–11.5% of patients treated with CQ plus AZM for malaria [173,174], 10% of patients with COVID-19 treated with CQ [223], 0–24% of patients treated with HCQ [239,247], 0.3–25% of patients treated with HCQ plus AZM [216,239,247], and 4.5–16.7% of patients treated with HCQ plus LPVr [212,239]. Dyspepsia and gastritis affected 3.6% of patients treated with HCQ for RA [143] and 1–4% of patients treated with CQ plus AZM for malaria [174]. Transaminase increase and other hepatic disorders were reported in the majority of patients treated with CQ or HCQ and affected by PCT but observed less often in patients treated for other conditions such as malaria, autoimmune conditions, or COVID-19 [135,136].

#### 3.4.5. Other Findings

Cases of hypoglycaemia (including a case of hypoglycaemic coma) [99,100,101,102], thrombocytopaenia [104], thrombotic thrombocytopaenic purpura [105,203], methaemoglobinaemia, sense organ adverse effects (including cases of severe positional vertigo [107], severe vestibular toxicity [108], loss of hearing [109], complete ageusia [110], diplopia and blurred vision) [106], porphyria variegata [116], severe myopathies [118,119], myasthenic syndrome [194], anaphylaxis [121], urinary incontinence [122], diffuse interstitial lung disease [123], and acute eosinophilic pneumonias [120], were described in connection with short-term CQ and HCQ treatments.

Various cases of haemolysis and methaemoglobinaemia were described in patients with glucose-6-phosphate dehydrogenase (G6PD) deficiency treated with HCQ or CQ for COVID-19 [183,184,204,205,206].

## 4. Discussion

The evidence collected does not show that COVID-19 patients treated with CQ or HCQ alone or in combination with studied drugs suffered a greater proportion of dermatologic, gastrointestinal, hepatic, metabolic, or haematological adverse effects compared with subjects receiving these drugs for indications other than COVID-19. However, no clinical benefits were found when those drugs were used to treat or prevent COVID-19 [281,282,283].

Although the ocular toxicity of CQ and HCQ is extremely important in long-term regimens with these drugs [284], it was rarely mentioned in connection with short-term regimens or in the first weeks of long-term regimens. The cases of patients with G6PD deficiency are of special interest because the related toxicity has been shown in such patients with and without COVID-19, and CQ and HCQ treatment must therefore be avoided in these patients [183,184]. During the first days of treatment, gastrointestinal adverse effects should be considered as they were reported in most indications. In the case of cardiac events, it is noteworthy that in more than 70 years of use, only a few cases of early cardiac adverse effects were found [94,95,96,97,98,99]. In contrast, in the much shorter period that has elapsed since the start of the COVID-19 pandemic, a greater number of cardiac cases have been reported involving COVID-19-affected patients regardless of the drug regimen used. In addition, the concomitant use of other drugs such as AZM should be considered. This systematic review shows that cardiac adverse effects such as QTc prolongation were frequent in COVID-19-affected-patients treated with CQ or HCQ (0–27.3%, and up to 33% if combined with AZM), though the risk of torsade de pointes was low. These data were extracted from 55 observational and 18 randomised studies with an overall favourable quality assessment. In 52 of these studies, at least some electrocardiographic changes were reported in patients treated with HCQ or CQ and a consistent and large effect was found. The results of this systematic review coincide with those of the previous one (that only included COVID-19 studies) that showed a significantly higher rate of adverse events with CQ or HCQ treatment but no significant differences in the case of serious adverse events (including cardiac arrhythmias and life-threatening events) [285]. A recent systematic review that focused on the cardiac safety of CQ and HCQ in COVID-19-affected patients has also shown an important association between CQ and HCQ use and the risk of drug-induced QT prolongation with a relatively higher incidence of torsade de pointes, ventricular tachycardia, or cardiac arrest [286]. Beyond the medication effects, several cardiac manifestations have been described in patients with COVID-19 including acute myopericarditis, acute coronary syndrome, congested heart failure, cardiogenic shock, and cardiac arrhythmias as a result of the injuries caused by the virus and systemic inflammation [287]. These cardiac manifestations were not only shown with CQ or HCQ use, but also with other drugs used in the treatment of COVID-19 such as corticosteroids, rivabirin, LPVr, and AZM [288,289,290,291]. This suggests that COVID-19 could have a role in these cardiac safety reports. Nevertheless, publication or measurement bias cannot be ruled out in pre-COVID-19 published data since these cardiac effects were rarely assessed or mentioned.

The cardiac abnormalities found in this study could be explained by CQ and HCQ electrophysiological effects, AZM combination, and COVID-19 concurrence. CQ and HCQ can cause acute cardiac functional changes by inhibition of ion channels with membrane-stabilizing effects that can lead to conduction disturbances [14]. Laboratory electrophysiological studies revealed CQ blocked the inward sodium current, the l-type calcium current, and the potassium currents such as the rapid delayed rectifier outward currents explaining prolongations and reductions in maximum velocity of cardiac action potentials and QT interval prolongation [292,293]. Moreover, synergistic effects of HCQ and AZM on the electrophysiological and contractile functions of human-induced pluripotent stem cell-derived cardiomyocytes have been observed in the short-term [294]. Furthermore, AZM might act as a weak CYP3A4 inhibitor involved in the metabolism of these drugs [295]. In addition to CQ, HCQ, and AZM effects, some common clinical concerns in elderly patients with COVID-19, such as dyselectrolythemia or dehydration, could increase the risk of arrhythmias [296,297]. In the case of neuropsychiatric symptoms associated with HCQ and CQ, different pharmacological mechanisms have been proposed such as serotonin or cholinergic imbalances induction or lysosomal dysfunction, although this has not been elucidated to date [280,298].

This systematic review only partially fulfils the proposed objectives. In patients not affected by COVID-19, only the combinations with AZM and glucocorticoids could be assessed, whereas in the case of patients affected by COVID-19, quantitative data synthesis could only be performed in combinations with AZM, LPVr, and boosted DRV but there were no sufficient studies reporting on the other possible combinations. The studies examined here were performed in very different settings and the methodologies used for the assessment of drug safety were very different, thus limiting our ability to compare the results reported. Furthermore, some adverse effects were not monitored with the same intensity in different contexts, so the question remains as to whether they did not occur or were simply not measured. Data synthesis was performed according to drug indication and whether CQ or HCQ were used alone or in combination with a second drug, but not according to how the dose regimen used could have influenced the adverse effects (e.g., drug regimens used in COVID-19 were generally longer than those used in malaria and in higher daily doses than those used in autoimmune diseases). Moreover, this systematic review was not focused on finding differences between CQ and HCQ safety. Despite these limitations, this systematic review clearly suggests that the use of CQ or HCQ tended to increase the cardiac risks for patients being treated for COVID-19, although these rarely resulted in severe consequences and the risk of torsade de pointes was low. Taking into account these considerations, in the future great caution should be exercised when testing potentially arrhythmogenic drugs in patients affected by severe acute viral or inflammatory pathologies.

## 5. Conclusions

Early adverse effects of CQ and HCQ may manifest as cardiac, dermatologic, neuropsychiatric, gastrointestinal, hepatic, metabolic, or haematological events. In the evidence reviewed, the occurrence and frequency of these toxicities were variable depending on the drug indication and the characteristics of the population being treated. Unlike pre-COVID-19 patients who received CQ or HCQ treatment, cardiac adverse drug effects occurred often in COVID-19 patients. Although severe consequences were rarely reported, this data must be considered, especially if CQ or HCQ are combined with other drugs such as AZM. This systematic review provides a comprehensive synthesis of the reported evidence on the short-term safety of CQ and HCQ treatment and provides important data for further research on the use of these drugs.

## Data Availability

The data presented in this study are available in article and Supplementary Material.

## Supplementary Materials

The following supporting information can be downloaded at: https://www.mdpi.com/1424-8247/15/5/634/s1, Table S1 search terms and MeSH terms used in the bibliographic search on the safety of chloroquine and hydroxychloroquine alone; Table S2: search terms and MeSH terms used in the bibliographic search on the safety of chloroquine and hydroxychloroquine in combination with other drugs used for the treatment of COVID-19 disease; Table S3: articles exclusion according to eligibility criteria; Table S4: total of adverse effects related to CQ/HCQ: case reports and case series; Table S5: characteristics of included studies: case reports and case series related to dermatological adverse events; Table S6: characteristics of included studies: case reports and case series related to psychiatric adverse events; Table S7: characteristics of included studies: case reports and case series related to neurologic adverse events; Table S8: characteristics of included studies: case reports and case series related to cardiac adverse events; Table S9: characteristics of included studies: case reports and case series related to hematologic and metabolic adverse events; Table S10: characteristics of included studies: case reports and case series related to sense organs adverse events; Table S11: characteristics of included studies: case reports and case series related to hepatic adverse events; Table S12: characteristics of included studies: case reports and case series related to other adverse events; Table S13: characteristics of included studies: observational studies, patients affected by malaria or who received prophylactic treatment; Table S14: characteristics of included studies: observational studies, patients affected by cutaneous and/or systemic lupus erythematosus and dermatomyositis; Table S15: characteristics of included studies: observational studies, patients affected by porphyria cutanea tarda; Table S16: characteristics of included studies: observational studies, patients affected by diverse pathologies; Table S17: characteristics of included studies: clinical trials, patients affected by malaria or who received prophylactic treatment; Table S18: characteristics of included studies: clinical trials, patients affected by rheumatoid arthritis; Table S19: characteristics of included studies: clinical trials, patients affected by other pathologies; Table S20: characteristics of included studies: clinical trials and observational studies, patients affected by malaria or who received prophylactic treatment with CQ or HCQ in combination with other drugs; Table S21: characteristics of included studies: case reports and case series in patients with COVID-19; Table S22: characteristics of included studies: observational studies and clinical trials (COVID-19 treatment and prophylaxis).
