# Supplementary material for: Safety of Short-Term Treatments with Oral Chloroquine and Hydroxychloroquine in Patients with and without COVID-19: A Systematic Review
Source: Pharmaceuticals (Basel). 2022 May 21;15(5):634. doi: 10.3390/ph15050634 (PMC9144263; doi:10.3390/ph15050634)
Supplement: Supplementary file 1 [file pharmaceuticals-15-00634-s001.zip › pharmaceuticals-1668804-supplementary/Systematic review Supplementary information part 1.pdf]

JOURNAL

Pharmaceuticals

TITLE

Safety of short-term treatments with oral chloroquine and hydroxychloroquine in patients with and without COVID-19: a systematic review

AUTHORS

1. Marin, Sergio (Corresponding Author).

Hospital Universitari Germans Trias i Pujol, Pharmacy Department, Badalona, Catalonia, SPAIN. ORCID ID: 0000-0001-7709-6504

Tel. +34 93 497 88 74. Fax. +34 93 497 89 37.

Email: sergiomarinrubio@gmail.com

2. Martin-Val, Alba.

Hospital Universitari Germans Trias i Pujol, Pharmacy Department, Badalona, Catalonia, SPAIN.

3. Bosch, Maite.

Hospital Universitari Germans Trias i Pujol, Pharmacy Department, Badalona, Catalonia, SPAIN.

University of Barcelona, Biochemistry and Physiology Department, Barcelona, Catalonia, SPAIN.

4. Rodríguez, Cristina.

Hospital Universitari Germans Trias i Pujol, Pharmacy Department, Badalona, Catalonia, SPAIN.

5. Pérez-Ricart, Ariadna.

Catalan Health System, North Barcelona Metropolitan Area, Pharmacy Department, Sant Cugat del Vallès, Catalonia, SPAIN.

Hospital Universitari Germans Trias i Pujol, Pharmacy Department, Badalona, Catalonia, SPAIN.

6. Vilaró Jaques, Laia.

Hospital Universitari Germans Trias i Pujol, Pharmacy Department, Badalona, Catalonia, SPAIN.

7. Paredes, Roger.

Hospital Universitari Germans Trias i Pujol, IrsiCaixa AIDS Institute, Badalona, Catalonia, SPAIN.

Hospital Universitari Germans Trias i Pujol, Infectious Diseases Department, Badalona, Catalonia, SPAIN.

8. Roca, Josep.

Hospital Universitari Germans Trias i Pujol, Epidemiology Unit, Badalona, Catalonia, SPAIN.

9. Quiñones, Carles.

Hospital Universitari Germans Trias i Pujol, Pharmacy Department, Badalona, Catalonia, SPAIN.

University of Barcelona, Clinical Pharmacy and Pharmacotherapy Unit, Department of Pharmacy and Pharmaceutical Technology, and Physical Chemistry, Barcelona, Catalonia, SPAIN.

Supplementary information part 1

Data collection and data items: specific gathered data on the to the design, the participants, the quality and the results of each study.

Case reports/series: (i) study identification: first author, title and journal and year of publication; ii) drug: CQ or HCQ and its salt if available; iii) drug indication; iv) ADR reported; (v) patients characteristics (age, gender and clinical data); (vi) ADR management (discontinuation, clinical management, drug reintroduction, long-term consequences and patient's follow-up); (vii) time of ADR appearance; (viii) cumulative dose; and (ix) presence of an ADR probability scale and its punctuation if available.

Case-control, cross-sectional, cohorts and randomized studies: items from i to v as in case reports/series; and (vi) sample size and study arms; (vii) study aim; (viii) main study characteristics (epidemiological approach, data gathering, time horizon and frame, location); (ix) patients inclusion and exclusion criteria; (x) ADR characteristics (measure, assessment and frequency if available); (xi) time of appearance (if available); (xii) cumulative dose; and (xiii) drug regimens. Data was presented in its original format and metrics.

Table S1: Search terms and MeSH terms used in the bibliographic search on the safety of Chloroquine and Hydroxychloroquine alone.

**Terms related to Chloroquine and Hydroxychloroquine and connected among themselves by "OR"**	**Terms related to Drug Adverse Events and connected among themselves by "OR"**	**Terms related to the Type of publication and connected among themselves by "OR"**
1. "Chloroquine"[Mesh]<br>2. Chloroquine[tiab]<br>3. Chloroquine phosphate[tiab]<br>4. Chloroquine sulfate[tiab]<br>5. Hydroxychloroquine[tiab]<br>6. Aminoquinolin*[tiab]<br>7. "Quinine/analogs and derivatives"[Mesh]<br>8. "Cinchona/analogs and derivatives"[Mesh])	9. "Drug-Related Side Effects and Adverse Reactions"[Mesh]<br>10. "Long Term Adverse Effects"[Mesh]<br>11. "Pharmacovigilance"[Mesh]<br>12. "Drug Monitoring"[Mesh]<br>13. "Adverse Drug Reaction Reporting Systems"[Mesh]<br>14. "Safety-Based Drug Withdrawals"[Mesh]<br>15. "Poisoning"[Mesh]<br>16. "Drug Eruptions"[Mesh]<br>17. "Toxic Optic Neuropathy"[Mesh]<br>18. "Sensation Disorders"[Mesh]<br>19. "Biomarkers, Pharmacological"[Mesh]<br>20. "Photosensitivity Disorders"[Mesh]<br>21. Adverse reaction/<br>22. Adverse effect*<br>23. Drug toxicity/<br>24. Toxici*<br>25. Cardiotox*<br>26. Liver injury/<br>27. Liver disease/<br>28. Liver toxicity/<br>29. Hepatitis/<br>30. Hypersensitiv*<br>31. Allergy/<br>32. Myotoxicity/<br>33. Ototoxicity/	34. "Case Reports"[Publication Type]<br>35. Case Report*[tiab]<br>36. Case Series<br>37. Case Serie*[tiab]<br>38. "Observational Study" [Publication Type]<br>39. "Case-Control Studies"[Mesh]<br>40. "Cohort Studies"[Mesh]<br>41. Retrospect*[tiab]<br>42. Prospecti*[tiab]<br>43. "Cross-Sectional Studies"[Mesh]<br>44. Cross-sectional[tiab]<br>45. "Clinical Trial"[Publication Type]

Terms, detailed in the three columns above, related to Chloroquine and hydroxychloroquine, Drug Adverse Events and the Type of Publication were connected using "AND". Subsequently, the following terms were added using NOT: "Review" [Publication Type], "Systematic Review" [Publication Type] and "Meta-Analysis" [Publication Type].

Table S2: Search terms and MeSH terms used in the bibliographic search on the safety of Chloroquine and Hydroxychloroquine in combination with other drugs used for the treatment of COVID-19 disease.

Terms related to Chloroquine and Hydroxychloroquine and connected among themselves by "OR"	Terms related to Drug Adverse Events and Drug-Drug Interactions and connected among themselves by "OR"	Terms related to the Type of publication and connected among themselves by "OR"
1. "Chloroquine"[Mesh]<br>2. Chloroquine[tiab]<br>3. Hydroxychloroquine[tiab]<br>4. Aminoquinolin*[tiab]<br>5. 4-Aminoquinolin*[tiab]	6. "Drug Interactions"[Mesh]<br>7. "Drug Therapy, Combination"[Mesh]<br>8. Drug-drug interaction*[tiab]<br>9. Drug interactions/<br>10. "Pharmacokinetics"[Mesh]<br>11. "Long QT Syndrome"[Mesh]<br>12. "Torsades de Pointes"[Mesh]<br>13. "Biological Availability"[Mesh]<br>14. "Gastrointestinal Absorption"[Mesh]<br>15. "Cytochrome P-450 Enzyme System"[Mesh]<br>16. "Pharmacogenetics"[Mesh]<br>17. "Pharmacogenomic Variants"[Mesh]<br>18. "Contraindications, Drug"[Mesh]<br>19. "Drug-Related Side Effects and Adverse Reactions"[Mesh]<br>20. "Long Term Adverse Effects"[Mesh]<br>21. "Pharmacovigilance"[Mesh]<br>22. "Drug Monitoring"[Mesh]<br>23. "Adverse Drug Reaction Reporting Systems"[Mesh]<br>24. "Safety-Based Drug Withdrawals"[Mesh]<br>25. "Poisoning"[Mesh]<br>26. "Biomarkers, Pharmacological"[Mesh]<br>27. Adverse reaction/<br>28. Adverse effect*<br>29. Drug toxicity/	30. "Remdesivir" [Supplementary Concept]<br>31. Remdesivir[tiab]<br>32. "HIV Protease Inhibitors"[Mesh]<br>33. "Lopinavir-ritonavir drug combination" [Supplementary Concept]<br>34. Ritonavir[tiab]<br>35. Lopinavir[tiab]<br>36. "Darunavir"[Mesh]<br>37. Darunavir [tiab]<br>38. "Cobicistat"[Mesh]<br>39. Cobicistat[tiab]<br>40. "Tenofovir"[Mesh]<br>41. Tenofovir[tiab]<br>42. "Levofloxacin"[Mesh]<br>43. Levofloxacin[tiab]<br>44. "Favipiravir" [Supplementary Concept]<br>45. Favipiravir[tiab]<br>46. "Arbidol" [Supplementary Concept]<br>47. Arbidol[tiab]<br>48. "Ribavirin"[Mesh]<br>49. Ribavirin[tiab]<br>50. Baricitinib[tiab]<br>51. "Glucocorticoids"[Mesh]<br>52. Glucocorticoid*[tiab]<br>53. "Prednisolone"[Mesh]<br>54. Prednisolone[tiab]<br>55. "Prednisone"[Mesh]<br>56. Prednisone[tiab]<br>57. Methylprednisolone[tiab]<br>58. "Dexamethasone"[Mesh]<br>59. Dexamethasone[tiab]<br>60. "Heparin"[Mesh]<br>61. Dalteparin[tiab]<br>62. Enoxaparin[tiab]<br>63. Nadroparin[tiab]<br>64. Tinzaparin[tiab]<br>65. "Tocilizumab" [Supplementary Concept]<br>66. Tocilizumab[tiab]<br>67. "Sarilumab" [Supplementary Concept<br>68. Sarilumab [tiab]<br>69. "Interleukin 1 Receptor Antagonist Protein"[Mesh]<br>70. Anakinra[tiab]<br>71. "Siltuximab" [Supplementary Concept]<br>72. "Interferon beta-1b"[Mesh]<br>73. Inferferon beta-1b[tiab]<br>74. "Azithromycin"[Mesh]<br>75. Azithromycin[tiab]

		76. "COVID-19 serotherapy" [Supplementary Concept]<br>77. "Antibodies, Neutralizing"[Mesh]

Terms, detailed in the three columns above, related to chloroquine and hydroxychloroquine, drug adverse events and drug-drug interactions and drugs utilized for the treatment of COVID-19 disease were connected using "AND". Subsequently, the following terms were added using NOT: "Review" [Publication Type], "Systematic Review" [Publication Type] and "Meta-Analysis" [Publication Type].

Results: reasons for articles exclusion according to eligibility criteria.

2516 articles were excluded according to eligibility criteria (161 not reported information on adult patients ≥18 years, 826 reported ADRs that occurred beyond the first 14 days of treatment, 37 were related to intoxications, on 18 articles the route of administration was different to the oral route, 2 were related to a labour exposition, 14 were surveys to health professionals, 160 assessed the validity of a diagnostic or screening technique, 9 contained preclinical data, 503 were excluded due to the type of publication reported, in 194 articles the adverse drug reaction was related to a drug combination different than those ones proposed, 21 did not indicate the temporary relation between drug intake and ADR appearance, 552 were duplicate articles and 19 articles were impossible to assess due to lack of information).

Table S3: articles exclusion according to eligibility criteria.

Reasons for articles exclusion according to eligibility criteria	Number of excluded articles
Not reported information on adult patients ≥18 years	161
Reported ADRs that occurred beyond the first 14 days of treatment	826
Intoxications	37
The route of administration was different to the oral route	18
Related to a labour exposition	2
Surveys to health professionals	14
Assessed the validity of a diagnostic or screening technique	160
Contained preclinical data	9
Type of publication reported	503
The adverse drug reaction was related to a drug combination different than those ones proposed	194
Not indicate the temporary relation between drug intake and ADR appearance	21
Duplicate articles	552
Impossible to assess due to lack of information	19
