# Supplementary material for: Safety of Short-Term Treatments with Oral Chloroquine and Hydroxychloroquine in Patients with and without COVID-19: A Systematic Review
Source: Pharmaceuticals (Basel). 2022 May 21;15(5):634. doi: 10.3390/ph15050634 (PMC9144263; doi:10.3390/ph15050634)
Supplement: Supplementary file 1 [file pharmaceuticals-15-00634-s001.zip › pharmaceuticals-1668804-supplementary/Systematic review Supplementary information part 2 .pdf]

JOURNAL

Pharmaceuticals

TITLE

Safety of short-term treatments with oral chloroquine and hydroxychloroquine in patients with and without COVID-19: a systematic review

AUTHORS

1. Marin, Sergio (Corresponding Author).

Hospital Universitari Germans Trias i Pujol, Pharmacy Department, Badalona, Catalonia, SPAIN. ORCID ID: 0000-0001-7709-6504

Tel. +34 93 497 88 74. Fax. +34 93 497 89 37.

Email: sergiomarinrubio@gmail.com

2. Martin-Val, Alba.

Hospital Universitari Germans Trias i Pujol, Pharmacy Department, Badalona, Catalonia, SPAIN.

3. Bosch, Maite.

Hospital Universitari Germans Trias i Pujol, Pharmacy Department, Badalona, Catalonia, SPAIN.

University of Barcelona, Biochemistry and Physiology Department, Barcelona, Catalonia, SPAIN.

4. Rodríguez, Cristina.

Hospital Universitari Germans Trias i Pujol, Pharmacy Department, Badalona, Catalonia, SPAIN.

5. Pérez-Ricart, Ariadna.

Catalan Health System, North Barcelona Metropolitan Area, Pharmacy Department, Sant Cugat del Vallès, Catalonia, SPAIN.

Hospital Universitari Germans Trias i Pujol, Pharmacy Department, Badalona, Catalonia, SPAIN.

6. Vilaró Jaques, Laia.

Hospital Universitari Germans Trias i Pujol, Pharmacy Department, Badalona, Catalonia, SPAIN.

7. Paredes, Roger.

Hospital Universitari Germans Trias i Pujol, IrsiCaixa AIDS Institute, Badalona, Catalonia, SPAIN.

Hospital Universitari Germans Trias i Pujol, Infectious Diseases Department, Badalona, Catalonia, SPAIN.

8. Roca, Josep.

Hospital Universitari Germans Trias i Pujol, Epidemiology Unit, Badalona, Catalonia, SPAIN.

9. Quiñones, Carles.

Hospital Universitari Germans Trias i Pujol, Pharmacy Department, Badalona, Catalonia, SPAIN.

University of Barcelona, Clinical Pharmacy and Pharmacotherapy Unit, Department of Pharmacy and Pharmaceutical Technology, and Physical Chemistry, Barcelona, Catalonia, SPAIN.

Supplementary information part 2

Table S4. Total of adverse effects related to CQ/HCQ: case reports and case series

**Organ affected**	**Number of patients, n (number of studies, n)**	**CQ:<br>Patients, n (studies, n)<br>- Indications**	**HCQ:<br>Patients, n (studies, n)<br>- Indications**
Dermatological	53 (39)*	12 (12):<br>- Psoriasis 2 (2)<br>- Malaria treatment or prophylaxis 9 (9)<br>- SLE 1 (1)	41 (28)**<br>- Psoriatic arthropathy 1 (1)<br>- Arthritis and related rheumatic disorders 17 (15)<br>- SLE and related disorders 11 (10)<br>- Pemphigus erythematosus 1 (1)<br>- Sjögren's syndrome and related disorders 4 (4)<br>- NA 1 (1)<br>- Granuloma annulare 1 (1)<br>- Erythematous facial dermatitis 1 (1)<br>- Photosensitivity 1 (1)<br>- Jessner-Kanof 1 (1)<br>- Mucinosis 1 (1)<br>- Autoimmune bullous skin disease 1 (1)
Psychiatric	22 (17)	20 (15)<br>- Acute myocardial infarction 1 (1)<br>- Rheumatoid arthritis 2 (2)<br>- Hepatic or intestinal amebiasis 4 (3)<br>- Malaria 11 (7)<br>- SLE and arthritis 1 (1)<br>- SLE 1 (1)	2 (2)<br>- Lupus erythematosus 1 (1)<br>- Discoid lupus erythematosus 1 (1)
Neurologic	16 (12)	15 (11)<br>- Hepatic or intestinal amebiasis 4 (1)<br>- Fever 1 (1)<br>- Malaria treatment or prophylaxis 8 (7)<br>- Erythema nodosum leprosum 1 (1)<br>- Prophylactic treatment of gastrointestinal parasitic infection 1 (1)	1 (1)<br>- Rheumatoid arthritis 1 (1)
Cardiac	6 (6)	3 (3)<br>- Dermatologic problem 1 (1)<br>- Malaria 1 (1)<br>- Hepatic and intestinal amebiasis 1 (1)	3 (3)<br>- SLE 2 (2)<br>- Rheumatoid arthritis 1 (1)
Hematologic and metabolic	6 (6)	2 (2)<br>- Psoriatic arthritis 1 (1)<br>- Fever 1(1)	4 (4)<br>- Arthritis and related rheumatic disorders 3 (3)<br>- Used erroneously as a pain killer 1 (1)
Sense organs	6 (5)*	3 (3)<br>- Hypercalcemia associated with sarcoidosis 1 (1)<br>- Malaria 2 (2)	3 (3)<br>- Rheumatoid arthritis 1 (1)<br>- Malaria 1 (1)<br>- SLE 1 (1)
Hepatic	5 (5)	1 (1)<br>- Malaria prophylaxis 1 (1)	4 (4)<br>- Arthritis 1 (1)<br>- SLE and related disorders 3 (3)
Other	9 (9)	3 (3)<br>- Malaria 1 (1)<br>- SLE with arthralgia and renal involvement 1 (1)<br>- Mild rosacea 1 (1)	6 (6)<br>- SLE and related disorders 3 (3)<br>- cGVHD 1 (1)<br>- Rheumatoid arthritis 1 (1)<br>- Polymorphic light eruption 1 (1)

*****One study reported cases with CQ and cases with HCQ.
******One study reported cases with different indications.
Abbreviations: cGVHD: chronic graft-versus-host disease; CQ: Chloroquine; HCQ: Hydroxychloroquine; NA: not available/not applicable; SLE: systemic lupus erythematosus.

Table S5. Characteristics of included studies: case reports and case series related to dermatological adverse events

Study ID: title, first author, journal of publication, year	Drug	Indication	Adverse reaction reported	Adverse effect management<br>(a) Discontinuation<br>(b) Management<br>(c) Reintroduction<br>(d) Long-term consequences<br>(e) Follow up	Population characteristics:<br>(a) Age (years)<br>(b) Gender<br>(c) Clinical characteristics	Other:<br>(a) Time of appearance<br>(b) Cumulative dose<br>(c) Regimen	Adverse Drug Reaction Probability Scale:<br>(a) Scale<br>(b) Score (probability category)	Quality assess-ment[a] (%)
Psoriatic erythroderma, rheumatoid arthritis, and death, as a sequence to a drug reaction. Fisher S. J Maine Med Assoc. 1961	CQ	Psoriasis	Exacerbation of psoriasis and arthritis	(a) No<br>(b) Steroid therapy, large doses of salicilates and starch baths and petrolatum<br>(c) Yes<br>(d) Death due to toxemia from staphylococcic peritonitis<br>(e) NA	(a) 45<br>(b) Female<br>(c) Lameness and aching on arising for years. Thought to have diabetes, hypertension, and hypothyroidism	(a) 2 days<br>(b) 0.75 g<br>(c) 0.125 g TD	(a) NA<br>(b) NA	63
Systemic eczematous contact-type dermatitis induced by iodochlorhydroxyquin and chloroquine phosphate. Skog E. Contact Dermatitis. 1975	CQ phosphate	Malaria prophylaxis	Eczema	(a) Yes<br>(b) NA<br>(c) NA<br>(d) NA<br>(e) NA	(a) 56<br>(b) Female<br>(c) NA	(a) 2 w<br>(b) 0.6 g<br>(c) 0.3 g weekly	(a) NA<br>(b) NA	35
Toxic epidermal necrolysis-drug induced (report of 2 cases). Kanwar AJ. Indian J Dermatol. 1976	CQ	Suspected malaria	Toxic epidermal necrolysis	(a) Yes<br>(b) Parenteral steroids, antibiotics and supportive measures<br>(c) NA<br>(d) NA<br>(e) Improving within a week	(a) 30<br>(b) Male<br>(c) NA	(a) 10 min<br>(b) NA<br>(c) The patient was given tablet of CQ	(a) NA<br>(b) NA	64
Chloroquine and psoriasis. Olsen TG. Ann Intern Med. 1981	CQ phosphate	Malaria	Exacerbation of psoriasis	(a) NA<br>(b) Increase in PUVA therapy. Methotrexate 2,5 mg/12 h for 36 h once weekly<br>(c) No<br>(d) No<br>(e) Under fair control	(a) 58<br>(b) Male<br>(c) Psoriasis well controlled with PUVA	(a) 2 w<br>(b) 1 g<br>(c) 0.5 g weekly	(a) NA<br>(b) NA	54
Hydroxychloroquine in psoriatic arthropathy: exacerbations of psoriatic skin lesions. Luzar MJ. J Rheumatol. 1982	HCQ	Psoriatic arthropathy	Severe exacerbation of psoriatic skin lesions	(a) Yes<br>(b) Topical corticosteroids<br>(c) No<br>(d) No<br>(e) Returned to baseline over 3-4 w	(a) 27<br>(b) Female<br>(c) NA	(a) 14 days<br>(b) 5.6 g<br>(c) 0.2 g BD	(a) NA<br>(b) NA	69
Chloroquine-associated pruritus in a European. Spencer HC. Br Med J (Clin Res Ed). 1982	CQ phosphate	Malaria	Severe pruritus	(a) No<br>(b) Antihistamines<br>(c) Yes<br>(d) No<br>(e) Itching continued for about 55 h	(a) 23<br>(b) Female<br>(c) NA	(a) 1 day<br>(b) 0.9 g<br>(c) 0.6 g initially and then 0.3 g at 8 h	(a) NA<br>(b) NA	48

Role of pantothenic acid in chloroquine induced skin toxicity. Bhasin V. J Indian Med Assoc. 1984	CQ phosphate	Malaria	Pruritus	(a) Yes<br>(b) Calcium pantothenate injection<br>(c) NA<br>(d) No<br>(e) Immediate response to treatment	(a) 35<br>(b) Male<br>(c) NA	(a) 3 days<br>(b) 2.5 g<br>(c) NA	(a) NA<br>(b) NA	67
Hydroxychloroquine provocation of psoriasis. Gray RG. J Rheumatol. 1985	HCQ	Seronegative rheumatoid arthritis	Psoriasis	(a) Yes<br>(b) Topical corticosteroids<br>(c) No<br>(d) No<br>(e) Skin lesions abated completely	(a) 59<br>(b) Female<br>(c) No history of psoriasis or other rash, photosensitivity, drug hypersensitivity, serositis, Raynaud's phenomenon, alopecia or oral ulcers; family history was negative for psoriasis or spondyloarthropathy	(a) 14 days<br>(b) 11.8 g<br>(c) 0.4 g QD	(a) NA<br>(b) NA	74
Erythema annulare centrifugum: an unusual case due to hydroxychloroquine sulfate. Hudson LD. Cutis. 1985	HCQ sulphate	Suspected SLE	Erythema annulare centrifugum	(a) Yes<br>(b) NA<br>(c) No<br>(d) No<br>(e) The eruption cleared at 1 year	(a) 56<br>(b) Female<br>(c) NA	(a) NA<br>(b) NA<br>(c) 0.4 g QD	(a) NA<br>(b) NA	80
Generalized pustular drug rash induced by hydroxychloroquine. Lotem M. Acta Derm Venereol. 1990	HCQ	Pemphigus erythematosus	Generalized pustular rash	(a) Yes<br>(b) Prednisone<br>(c) No<br>(d) No<br>(e) The skin became cleaner during the following 4 w. During the year following this episode, no further skin lesions appeared	(a) 69<br>(b) Male<br>(c) Ischemic heart disease	(a) 14 days<br>(b) 2.8 g<br>(c) 0.2 g QD	(a) NA<br>(b) NA	76
Psoriasis worsened by antimalarial prophylaxis. Vestey JP. J Infect. 1992	CQ phosphate	Psoriasis	Erythrodermic psoriasis	(a) Yes<br>(b) Topical and oral corticosteroids. Dressings with coal tar paste<br>(c) No<br>(d) No<br>(e) Psoriasis remained well controlled with usual treatment	(a) 45<br>(b) Male<br>(c) NA	(a) 6 days<br>(b) 0.5 g<br>(c) 500 mg weekly	(a) NA<br>(b) NA	81
Acute generalized exanthematous pustulosis induced by hydroxychloroquine. Assier-Bonnet H. Dermatology. 1996	HCQ	Seronegative polyarthritis	AGEP	(a) Yes<br>(b) NA<br>(c) No<br>(d) No<br>(e) The disease was rapidly self-limiting without treatment and there was no relapse of the eruption after 19 months	(a) 36<br>(b) Female<br>(c) No history or family psoriasis	(a) 12 days<br>(b) 2.4 g<br>(c) 0.2 g QD	(a) NA<br>(b) NA	72
Pustular eruption in a malaria patient treated with chloroquine. Wilairatana P. Int J Dermatol. 1998	CQ phosphate	Malaria	Pustular eruption	(a) Yes<br>(b) NA<br>(c) No<br>(d) No<br>(e) After discontinuation the eruption quickly resolved with mild desquamation	(a) 42<br>(b) Female<br>(c) Good health and no history of skin disease	(a) 1 day<br>(b) 1.8 g<br>(c) A total dose of 1.8 g over 6h	(a) NA<br>(b) NA	76

Fatal toxic epidermal necrolysis associated with hydroxychloroquine. Murphy M. Clin Exp Dermatol. 2001	HCQ	Seropositive nodular rheumatoid disease	Fatal toxic epidermal necrolysis	(a) Yes<br>(b) NA<br>(c) No<br>(d) Death<br>(e) NA	(a) 39<br>(b) Female<br>(c) Lymphopenia, positive ribonucleoprotein antibody, fatty liver with hepatic alteration, intolerant of other second-line agents, including a psorisiform rash with gold. The patient volunteered no other history of skin disease	(a) 14 days<br>(b) 11.2 g (inadvertently the patient took twice the prescribed dose of HCQ, intake < 5 times the DDD of 0.516 g of HCQ base)<br>(c) 0.4 g BD	(a) NA<br>(b) NA	64
Stevens-Johnson syndrome in association with hydroxychloroquine treatment for rheumatoid arthritis. Leckie MJ. Rheumatology (Oxford). 2002	HCQ	Rheumatoid arthritis	Stevens-Johnson syndrome	(a) Yes<br>(b) IV followed by oral and topical steroids and emolients<br>(c) No<br>(d) NA<br>(e) The rash improved but persisted	(a) 65<br>(b) Female<br>(c) Hypertension	(a) 2 w<br>(b) 5.6 g<br>(c) 0.2 g BD	(a) NA<br>(b) NA	68
Acute pustular psoriasis complicated by leukocytoclastic vasculitis. Welsch MJ. J Drugs Dermatol. 2003	HCQ	Sjögren's syndrome	Acute pustular psoriasis	(a) Yes<br>(b) Oral corticosteroids and acetretin<br>(c) No<br>(d) NA<br>(e) Markedly improved and no recurrence of psoriatic lesions occurred	(a) 65<br>(b) Female<br>(c) Hypertension, chronic renal insufficiency, hyperlipidemia, gout, arthritis and diverticulitis	(a) 10 days<br>(b) NA<br>(c) NA	(a) NA<br>(b) NA	69
Acute generalized exanthematous pustulosis precipitated by hydroxychloroquine. Evans CC. J Am Acad Dermatol. 2004	HCQ	SLE	AGEP	(a) Yes<br>(b) NA<br>(c) No<br>(d) No<br>(e) Resolution of the skin lesions after approximately 3 w	(a) 28<br>(b) Female<br>(c) NA	(a) 14 days<br>(b) 5.6 g<br>(c) 0.2 g BD	(a) NA<br>(b) NA	64
Chloroquine/hydroxy-chloroquine-induced pemphigus. Ghaffarpour G. Int J Dermatol. 2006	HCQ	Rheumatoid arthritis	Pemphigus vulgaris	(a) Yes<br>(b) Topical steroid<br>(c) No<br>(d) No<br>(e) After 3 w the lesions cleared with only a mild post-inflammatory hyperpigmentation	(a) 52<br>(b) Female<br>(c) General health was good	(a) 14 days<br>(b) NA<br>(c) NA	(a) NA<br>(b) NA	90
A case of hydroxychloroquine induced pruritus. Gül U. Eur J Dermatol. 2006	HCQ	Discoid lupus erythematosus	Severe pruritus	(a) Yes<br>(b) NA<br>(c) Yes<br>(d) Severe pruritus 1 w after the reinitiation<br>(e) Pruritus resolved in 1 w	(a) 36<br>(b) Female<br>(c) NA	(a) 10 days<br>(b) 4 g<br>(c) 0.4 g QD	(a) NA<br>(b) NA	78
Desensitization to hydroxychloroquine—experience of 4 patients. Mates M. J Rheumatol. 2006	HCQ	Seronegative inflammatory arthritis	Hypersensitivity rash	(a) Yes<br>(b) Etoricoxib<br>(c) Yes (desensitization)<br>(d) NA<br>(e) Rash gradually subsided	(a) 64<br>(b) Female<br>(c) NA	(a) 10 days<br>(b) 4 g<br>(c) 0.4 g QD	(a) NA<br>(b) NA	57

		Inflammatory arthritis		(a) Yes<br>(b) Antihistamine<br>(c) Yes (desensitization)<br>(d) NA<br>(e) The rash gradually subsided	(a) 34<br>(b) Female<br>(c) Oral and ophthalmic sicca symptoms, fatigue, lupus erythematosus and Sjögren's syndrome	(a) 8 days<br>(b) 3.2 g<br>(c) 0.4 g QD	(a) NA<br>(b) NA	
		Polyarticular onset juvenile chronic arthritis		(a) Yes<br>(b) NA<br>(c) Yes (desensitization)<br>(d) NA<br>(e) Gradual resolution of the rash. After rechallenge with HCQ 50 mg, resulted in a maculopapular rash and mild dyspnea within 20 minutes	(a) 21<br>(b) Female<br>(c) NA	(a) 2 w<br>(b) NA<br>(c) NA	(a) NA<br>(b) NA	
		Mild arthritis		(a) Yes<br>(b) Prednisone<br>(c) Yes (desensitization)<br>(d) NA<br>(e) The rash gradually disappeared	(a) 41<br>(b) Female<br>(c) Lupus erythematosus	(a) 1 w<br>(b) NA<br>(c) NA	(a) NA<br>(b) NA	
*Acute generalized exanthematous pustulosis: the experience of an Italian drug-surveillance centre. Atzori L. G Ital Dermatol Venereol. 2007	HCQ	NA	AGEP	(a) NA<br>(b) NA<br>(c) No<br>(d) NA<br>(e) Negative drug patch test	(a) 48<br>(b) Female<br>(c) NA. Not taking any other drug	(a) 3 days<br>(b) NA<br>(c) NA	(a) NA<br>(b) NA	NA
Hydroxychloroquine-induced DRESS syndrome. Volpe A. Clin Rheumatol. 2008	HCQ	Seronegative polyarthritis	DRESS	(a) Yes<br>(b) Prednisolone 12 mg/day was prescribed with tapering of the dose over 4 w<br>(c) No<br>(d) No<br>(e) At discharge from the hospital the abnormal laboratory values were improving. The rash eventually resolved after 60 days	(a) 62<br>(b) Male<br>(c) Alcoholic steatohepatitis and hypercholesterolemia on rosuvastatin	(a) 14 days<br>(b) 5.6 g<br>(c) 0.4 g QD	(a) NA<br>(b) NA	92
Mucous membrane grafting for the post-Steven-Johnson syndrome symblepharon: a case report. Das JK. Indian J Ophthalmol. 2011	CQ phosphate	Malaria	Steven–Johnson syndrome	(a) NA<br>(b) The erythematous lesions resolved completely at 4 w with medications. The patient was taken up for surgery and superficial lamellar dissection of the cornea was done<br>(c) NA<br>(d) NA<br>(e) The patient showed remarkable recovery and maintained till the 2.5-year follow-up without any other sequelae	(a) 18<br>(b) Female<br>(c) NA	(a) 5 days<br>(b) NA<br>(c) NA	(a) NA<br>(b) NA	87

Reacción adversa a hidroxicloroquina. Awad P. Rev Chilena Dermatol. 2013	HCQ	Chronic cutaneous lupus	Cutaneous rash	(a) NA<br>(b) Prednisone and chlorpheniramine<br>(c) NA<br>(d) NA<br>(e) Favourable evolution with decrease in pruritus and regression of skin lesions	(a) 62<br>(b) Female<br>(c) NA	(a) 14 days<br>(b) 2.8 g<br>(c) 0.2 g QD	(a) NA<br>(b) NA (probable)	65
Acute generalized exanthematous pustulosis induced by hydroxychloroquine: first case report in canada and review of the literature. Bailey K. J Cutan Med Surg. 2013	HCQ	Lupus erythematosus	AGEP	(a) Yes<br>(b) Methylprednisolone IV, oral prednisone, cefazoline, piperacillin/tazobactam<br>(c) NA<br>(d) No<br>(e) Skin lesions continued to resolve	(a) 48<br>(b) Female<br>(c) Without personal or family history of psoriasis	(a) 2 w<br>(b) 2.8 g<br>(c) 0.2 g QD	(a) NA<br>(b) NA	79
Hydroxychloroquine-induced fatal toxic epidermal necrolysis complicated by angioinvasive rhizopus. Cameron MC. Dermatol Online J. 2014	HCQ	SLE	Toxic epidermal necrolysis	(a) Yes<br>(b) Immunoglobulin IV, broadspectrum IV antibiotics and supportive care<br>(c) No<br>(d) Death<br>(e) NA	(a) 30<br>(b) Female<br>(c) Stevens-Johnson syndrome	(a) 3 days<br>(b) NA<br>(c) NA	(a) NA<br>(b) NA	91
Acute cutaneous pustular eruption due to hydroxychloroquine. Pastushenko I. Med Clin (Barc). 2014	HCQ	Rheumatoid arthritis	Pustular eruption	(a) Yes<br>(b) Histopathologic examination<br>(c) No<br>(d) No<br>(e) Resolution at 14 days	(a) 42<br>(b) Female<br>(c) NA	(a) 15 days<br>(b) 3 g<br>(c) 0. 2 g QD	(a) NA<br>(b) NA	63
Cutaneous adverse drug reactions with antimalarials and allergological skin tests. Soria A. Dermatology. 2015	HCQ	Rheumatism	Photosensitivity	(a) Yes<br>(b) Skin test and oral provocation test<br>(c) NA<br>(d) Recurrence after the 48 h of oral provocation test<br>(e) NA	(a) 45<br>(b) Female<br>(c) NA	(a) 10 days<br>(b) NA<br>(c) NA	(a) Naranjo[b]<br>(b) 8 (probable)	80
	HCQ	Granuloma annulare	AGEP/DRESS	(a) Yes<br>(b) Skin test<br>(c) NA<br>(d) No<br>(e) Twelve days to recovery	(a) 60<br>(b) Female<br>(c) NA	(a) 10 days<br>(b) NA<br>(c) NA	(a) Naranjo[b]<br>(b) 8 (probable)	
	HCQ	Erythematous facial dermatitis	AGEP	(a) Yes<br>(b) Skin test<br>(c) NA<br>(d) No<br>(e) Seven days to recovery	(a) 52<br>(b) Male<br>(c) NA	(a) 3 days<br>(b) NA<br>(c) NA	(a) Naranjo[b]<br>(b) 8 (probable)	
	HCQ	Photosensitivity	AGEP	(a) Yes<br>(b) Skin test<br>(c) NA<br>(d) No<br>(e) Ten days to recovery	(a) 48<br>(b) Female<br>(c) NA	(a) 7 days<br>(b) NA<br>(c) NA	(a) Naranjo[b]<br>(b) 8 (probable)	

	HCQ	Jessner-Kanof	Urticaria	(a) Yes<br>(b) Skin test and oral provocation test<br>(c) NA<br>(d) No<br>(e) NA	(a) 52<br>(b) Male<br>(c) NA	(a) 10 days<br>(b) NA<br>(c) NA	(a) Naranjo[b]<br>(b) 8 (probable)	
	HCQ	Gougerot-Sjögren syndrome	MPE	(a) Yes<br>(b) Skin test and oral provocation test<br>(c) NA<br>(d) No<br>(e) NA	(a) 66<br>(b) Male<br>(c) NA	(a) 2 days<br>(b) NA<br>(c) NA	(a) Naranjo[b]<br>(b) 8 (probable)	
	HCQ	Rheumatoid arthritis	AGEP	(a) Yes<br>(b) Skin test<br>(c) NA<br>(d) No<br>(e) Eleven days to recovery	(a) 45<br>(b) Female<br>(c) NA	(a) 15 days<br>(b) NA<br>(c) NA	(a) Naranjo[b]<br>(b) 6 (probable)	
	HCQ	Mucinosis	AGEP	(a) Yes<br>(b) Skin test<br>(c) NA<br>(d) No<br>(e) NA	(a) 66<br>(b) Female<br>(c) NA	(a) 8 days<br>(b) NA<br>(c) NA	(a) Naranjo[b]<br>(b) 6 (probable)	
	HCQ	Autoimmune bullous skin disease	Photosensivity	(a) Yes<br>(b) Skin test<br>(c) NA<br>(d) NA<br>(e) NA	(a) 61<br>(b) Male<br>(c) NA	(a) 6 days<br>(b) NA<br>(c) NA	(a) Naranjo[b]<br>(b) 6 (probable)	
	CQ	SLE	Photosensivity	(a) Yes<br>(b) Skin test<br>(c) NA<br>(d) NA<br>(e) NA	(a) 27<br>(b) Female<br>(c) NA	(a) 14 days<br>(b) NA<br>(c) NA	(a) Naranjo[b]<br>(b) 6 (probable)	
	HCQ	Cutaneous lupus erythematous	MPE and face edema	(a) Yes<br>(b) Skin test and oral provocation test<br>(c) NA<br>(d) No<br>(e) NA	(a) 26<br>(b) Female<br>(c) NA	(a) 5 days<br>(b) NA<br>(c) NA	(a) Naranjo[b]<br>(b) 6 (probable)	
	HCQ	Cutaneous lupus erythematous	Urticaria	(a) Yes<br>(b) Skin test and oral provocation test<br>(c) NA<br>(d) No<br>(e) NA	(a) 27<br>(b) Female<br>(c) NA	(a) 1 h<br>(b) NA<br>(c) NA	(a) Naranjo[b]<br>(b) 5 (probable)	
Prolonged pustular eruption from hydroxychloroquine: an unusual case of acute generalized exanthematous	HCQ	Rheumatoid arthritis	AGEP	(a) Yes<br>(b) Methylprednisolone IV, topical wet wraps with triamcinolone 0.1% and hydrocortisone 2.5% cream<br>(c) No	(a) 50<br>(b) Female<br>(c) NA	(a) 2 w<br>(b) 6 g<br>(c) 0.2 g BD	(a) NA<br>(b) NA	86

pustulosis. Pearson KC. Cutis. 2016				(d) No<br>(e) The eruption resolved and the patient was back to her baseline prednisone dosage of 5 mg/day				
Hydroxychloroquine-induced erythema multiforme. Abou Assalie N. J Clin Rheumatol. 2017	HCQ	SLE	Erythema multiform e	(a) Yes<br>(b) Methylprednisolone 1 g IV during 3 days followed by oral prednisone<br>(c) No<br>(d) No<br>(e) Normal skin without any lesions	(a) 25<br>(b) Female<br>(c) Hashimoto thyroiditis and autoimmune haemolytic anaemia	(a) 12 days<br>(b) 5.6 g<br>(c) 0.2 g BD	(a) NA<br>(b) NA	85
Early cutaneous eruptions after oral hydroxychloroquine in a lupus erythematosus patient: a case report and review of the published work. Matsuda T. J Dermatol. 2018	HCQ	Lupus erythema tosus	Mild cutaneou s eruptions	(a) No (patient's decision)<br>(b) Topical steroid<br>(c) Yes<br>(d) No<br>(e) Disappeared within 1 w	(a) 30<br>(b) Female<br>(c) NA	(a) 2 w<br>(b) 2.8 g<br>(c) 0.2 g QD	(a) NA<br>(b) NA	77
Palmoplantar exfoliation due to chloroquine. Nair PA. Indian J Pharmacol. 2017	CQ	Malaria	Palmo-plantar exfoliatio n	(a) Yes<br>(b) Topical antibiotics and emollients<br>(c) No<br>(d) NA<br>(e) NA	(a) 40<br>(b) Female<br>(c) A history of similar complaint thrice in 2 years after ingestion of medications for fever	(a) After taking tablet of CQ<br>(b) NA<br>(c) NA	(a) Naranjo[b]<br>(b) 8 (probable)	79
AGEP overlap induced by hydroxychloroquine: a case report and literature review. Mercogliano C. J Community Hosp Intern Med Perspect. 2018	HCQ	Rheumat oid factor negative arthritis	AGEP	(a) Yes<br>(b) IV fluid resuscitation, corticoids IV, anti-histamines<br>(c) No<br>(d) No<br>(e) No long term sequelae	(a) 71<br>(b) Female<br>(c) NA	(a) 14 days<br>(b) NA<br>(c) NA	(a) NA<br>(b) NA	94
A case of an acute cutaneous drug reaction with hydroxychloroquine. Randhawa A. Scott Med J. 2018	HCQ	Seronega tive inflamma tory arthritis	Acute DRESS	(a) Yes<br>(b) Oral prednisolone 30 mg/day, topical mometasone ointment and a greasy emollient. A strict fluid balance was kept<br>(c) No<br>(d) No<br>(e) After four days of treatment, the rash became less florid and started to desquamate with peeling and shearing of the skin on the soles of the feet	(a) 63<br>(b) Male<br>(c) NA	(a) 15 days<br>(b) 6 g<br>(c) 0. 2 g BD	(a) NA<br>(b) NA	84
Chloroquine induced urticaria: a newer adverse effect. Balamurugesan K. J Family Med Prim Care. 2019	CQ	Malaria	Urticaria	(a) NA<br>(b) Antihistamines and hydrocortisone<br>(c) NA<br>(d) NA<br>(e) The patient improved symptomatically	(a) 35<br>(b) Male<br>(c) Not known diabetes, hypertension or any other chronic illness. No past history of tuberculosis, asthma, and allergy	(a) Immediately after the first dose<br>(b) CQ 10 mg/kg<br>(c) CQ 10 mg/kg on Day-1, another 10 mg/Kg on Day 2 and 5 mg/Kg on Day 3	(a) NA<br>(b) NA	86

Pustular DRESS syndrome secondary to hydroxychloroquine with EBV Reactivation. Girijala RL. J Drugs Dermatol. 2019	HCQ	Suspected Sjögren's like process	Pustular DRESS	(a) NA<br>(b) Methylprednisolone was tapered from 60 mg/6 h to 80 mg/12 h. The patient received an infusion of infliximab<br>(c) No<br>(d) No<br>(e) Control of the eruption was achieved	(a) 56<br>(b) Female<br>(c) Premature ventricular contractions	(a) 14 days<br>(b) NA<br>(c) NA	(a) NA<br>(b) NA	84
Sweet's syndrome following therapy with hydroxychloroquine in a patient affected with elderly-onset primary Sjögren's syndrome. Manzo C. Medicines (Basel). 2019	HCQ	Sjögren syndrome	Sweet's syndrome	(a) Yes<br>(b) Prednisone 25 mg/day<br>(c) Yes<br>(d) In a few days the same skin lesions reappeared. Withdrawal of HCQ and a new cycle of prednisone allowed for the permanent disappearance of the skin lesions<br>(e) The cutaneous manifestations disappeared completely	(a) 72<br>(b) Female<br>(c) Arterial hypertension (amlodipine 5 mg/day) and non-hemodynamically significant carotid atheromasia (acetylsalicylic acid 100 mg/day)	(a) 14 days<br>(b) 5.6 g<br>(c) 0.4 g BD	(a) Naranjo[b]<br>(b) 10 (definite)	92
Hydroxychloroquine-induced inverse psoriasis. Ullah A. BMJ Case Rep. 2019	HCQ	Rheumatoid arthritis	Inverse psoriasis	(a) Yes<br>(b) Methotrexate therapy<br>(c) No<br>(d) No<br>(e) Complete resolution of the rash and mouth sores in 2 w	(a) 65<br>(b) Female<br>(c) Strong family history of psoriasis	(a) 7 days<br>(b) NA<br>(c) NA	(a) NA<br>(b) NA	83
Acute generalized exanthematous pustulosis induced by hydroxychloroquine successfully treated with etretinate. Matsuda-Hirose H. J Dermatol. 2020	HCQ	SLE	AGEP	(a) Yes<br>(b) Prednisolone and etretinate 20 mg/day<br>(c) No<br>(d) No<br>(e) The eruptions disappeared completely and etretinate was stopped after 84 days of administration. No relapse was seen after several months of follow-up	(a) 31<br>(b) Female<br>(c) NA	(a) 15 days<br>(b) 3 g<br>(c) 0.2 g QD	(a) NA<br>(b) NA	89

Abbreviations: AGEP: Acute generalized exanthematous pustulosis; BD: twice a day; CQ: Chloroquine; DDD: defined daily dose; EBV: Epstein-Barr virus; DRESS: Drug rash with eosinophilia and systemic symptoms; g: grams; h: hours; HCQ: Hydroxychloroquine; IV: intravenous; kg: kilograms; mg: milligrams; MPE= maculopapular exanthema; NA: not available/not applicable; QD: once a day; SLE: systemic lupus erythematosus; TD: three times a day; w: weeks.

[a]Quality assessment: a higher score indicates a higher quality. (%) [Yes (1) + Partly (0.5)/Total applicable] x100
[b]Naranjo, C.A.; Busto, U.; Sellers, E.M.; Sandor, P.; Ruiz, I.; Robert, E.A.; Janecek, E.; Domecq, C.; Greenblatt, D.J. A method for estimating the probability of adverse drug reaction. Clin. Pharmacol. Ther. 1981, 30, 239–245.

*case extracted from an observational study.

Table S6. Characteristics of included studies: case reports and case series related to psychiatric adverse events

Study ID: title, first author, journal of publication, year	Drug	Indication	Adverse reaction reported	Adverse effect management<br>(a) Discontinuation<br>(b) Management<br>(c) Reintroduction<br>(d) Long-term consequences<br>(e) Follow up	Population characteristics:<br>(a) Age (years)<br>(b) Gender<br>(c) Clinical characteristics	Other:<br>(a) Time of appearance<br>(b) Cumulative dose<br>(c) Regimen	Adverse Drug Reaction Probability Scale:<br>(a) Scale<br>(b) Score (probability category)	Quality assess-ment[a] (%)
*Chloroquine and hydroxychloroquine in the treatment of cardiac arrhythmias. Burrell Z. N Engl J Med. 1958	CQ	Acute myocardial infarction	Psychosis	(a) Yes<br>(b) NA<br>(c) No<br>(d) No<br>(e) NA	(a) 70<br>(b) Female<br>(c) NA	(a) 5 days<br>(b) 4 g<br>(c) 0.25 g every 6 h	(a) NA<br>(b) NA	NA
Chloroquine psychosis? Dornhorst AC. The Lancet. 1963	CQ sulphate	Rheumatoid arthritis	Psychosis	(a) Yes<br>(b) Discontinuation<br>(c) No<br>(d) No<br>(e) By the end of the eighth week the patient was substantially normal and had no recurrence	(a) 65<br>(b) Female<br>(c) No previous psychiatric history	(a) 4 days<br>(b) 2.4 g<br>(c) 0.25 BD	(a) NA<br>(b) The evidence that the episode was due to CQ therapy is by no means conclusive, but it seems at least possible that this was the cause	58
Two cases of chloroquine psychosis. Rab SM. Br Med J. 1963	CQ sulphate	Hepatic amebiasis	Psychosis	(a) Yes<br>(b) Chlorpromazine and fluids IV<br>(c) No<br>(d) No<br>(e) The patient became lucid and rational	(a) 34<br>(b) Male<br>(c) No comorbidities	(a) 6 days<br>(b) 6 g CQ sulphate (4.8 g CQ base)<br>(c) 0.25 g QID	(a) NA<br>(b) NA	88
				(a) Yes<br>(b) He was treated with chlorpromazine.<br>(c) No<br>(d) No<br>(e) Within three days his mental status had reverted to normal	(a) 24<br>(b) Male<br>(c) No comorbidities	(a) 6 days<br>(b) 6 g CQ sulphate (4.8 g CQ base)<br>(c) 0.25 g QID		
Toxic psychosis due to quinacrine and chloroquine. Oscar L. JAMA. 1964	CQ phosphate	Intestinal amoebiasis	Psychosis	a) Yes<br>(b) Phenobarbital and methenamine mandelate 1 g/6 h to acidify the urine<br>(c) No<br>(d) No<br>(e) At discharge the patient felt well	(a) 55<br>(b) Male<br>(c) NA	(a) 6 days<br>(b) 4 g<br>(c) 0.5 g BD for two days. Then reduced to 0.25 g BD	(a) NA<br>(b) NA	83
Chloroquine psychosis. Kabir SM. Trans R Soc Trop Med Hyg. 1969	CQ phosphate	Malaria	Psychosis	(a) Yes<br>(b) Chlorpromazine<br>(c) NA<br>(d) No<br>(e) Free of mental symptoms after 3 days and without recurrence	(a) 35<br>(b) Female<br>(c) No previous history of psychiatric illness	(a) 2 days<br>(b) 2 g<br>(c) 0.25 g 4 tablets initially followed by 2 tablets after 6 hours and subsequently 1 tablet BD for two days	(a) NA<br>(b) NA	57

Chloroquine psychosis. Bomb BS. Trans R Soc Trop Med Hyg. 1975	CQ phosphate	Malaria	Psychosis	(a) Yes<br>(b) Chlorpromazine<br>(c) NA<br>(d) NA<br>(e) NA	(a) 45<br>(b) Female<br>(c) Deranged mental functions	(a) 2 h<br>(b) 1 g<br>(c) First dose of 1 g	(a) NA<br>(b) NA	35
Chloroquine—related depression. Das EM. Indian J Psychiatry. 1981	CQ	Malaria	Moderate to severe depression	(a) NA<br>(b) Amitryptyline 100 mg per day<br>(c) NA<br>(d) No<br>(e) Improvement after 4 days of amitryptiline	(a) 40<br>(b) Female<br>(c) No previous history of mental illness or any family loading of mental illness	(a) 4 days<br>(b) 1.5 g<br>(c) Total dose of 1.5 g	(a) NA<br>(b) NA	41
				(a) NA<br>(b) Tricyclic antidepressant<br>(c) NA<br>(d) No<br>(e) Dramatic improvement on the fifth day	(a) 32<br>(b) Female<br>(c) No previous or family history of mental illness	(a) 5 days<br>(b) 1.8 g<br>(c) Administration of 1.8 g	(a) NA<br>(b) NA	
Chloroquine psychosis: a chemical psychosis? Mohan D. J Natl Med Assoc. 1981	CQ sulphate or phosphate	Malaria	Psychosis	(a) Yes<br>(b) Chlorpromazine (25 mg to 300 mg)<br>(c) No<br>(d) No<br>(e) Psychiatric manifestations in the patients lasted for 1 to 2 w and all patients made a quick recovery	(a) From 19 to 40 years old<br>(b) 4 females; 6 males<br>(c) NA	(a) From 3 to 10 days<br>(b) From 2 g to 6 g<br>(c) NA	(a) NA<br>(b) NA	85
Toxic psychosis: a complication of antimalarial therapy. Ward WQ. J Am Acad Dermatol. 1985	HCQ	Lupus erythematosus	Psychosis	(a) Yes<br>(b) The patient was given no medication<br>(c) No<br>(d) No<br>(e) Twelve days after discharge the mental state was completely normal	(a) 40<br>(b) Male<br>(c) No previous history of psychiatric disturbance or other drugs	(a) 10 days<br>(b) 8 g<br>(c) 0. 8 g QD	(a) NA<br>(b) NA	55
Chloroquine induced mania. Akhtar S. Int J Psychiatry Med. 1993	CQ base	Malaria	Mania	(a) Yes<br>(b) Hospital psychiatric assessment. Carbamazepine, haloperidol, trihexyphenidyl and electroconvulsive therapy<br>(c) No<br>(d) Disorder remains beyond 5 months<br>(e) NA	(a) 18<br>(b) Male<br>(c) No history or family psychiatric disorders. No exposure to any psychoactive drug	(a) 3 days<br>(b) 2.4 g<br>(c) Four tablets of CQ (0.6 g base)	(a) NA<br>(b) NA	81
				(a) Yes<br>(b) Hospital psychiatric assessment. Chlorpromazine and electroconvulsive therapy<br>(c) Yes<br>(d) NA<br>(e) No psychiatric symptoms with reintroduction	(a) 18<br>(b) Male<br>(c) Previous history of a psychiatric disorder taking CQ. No exposure to any psychoactive drug. No family psychiatric disorders	(a) 4 days<br>(b) NA<br>(c) 0.6 g base stat and 0.15 g BD for three days	(a) NA<br>(b) NA	

				(a) Yes<br>(b) Haloperidol and trihexyphenidyl<br>(c) No<br>(d) No<br>(e) Remained well at 2 years	(a) 38<br>(b) Male<br>(c) No history or family psychiatric disorders. No exposure to any psychoactive drug	(a) 6 days<br>(b) 1.35 g<br>(c) 3 tablets of 0.15 g in divided dosage of three days duration	(a) NA<br>(b) NA	
				(a) Yes<br>(b) Haloperidol and diazepam<br>(c) Yes<br>(d) No<br>(e) No psychiatric problems at 3 years	(a) 30<br>(b) Male<br>(c) No history or family psychiatric disorders. No exposure to any psychoactive drug	(a) 1 day<br>(b) NA<br>(c) Four tables stat and one BD for four days	(a) NA<br>(b) NA	
Serious psychiatric symptoms after chloroquine treatment following experimental malaria infection. Telgt DS. Ann Pharmacother. 2005	CQ phosphate	Malaria	Psychotic disorder with symptoms of depersonalization and anxiety	(a) No<br>(b) Oxazepam 10 mg/day. The patient refused to take antipsychotic medication<br>(c) No<br>(d) No<br>(e) After 4 months the patient was completely recovered	(a) 34<br>(b) Female<br>(c) No history of somatic diseases, including malaria, and without any personal or family history of mental illness	(a) 1 day<br>(b) 1.2 g<br>(c) 0.6 g followed by 0.3 g after , 24 and 48 h (total dose 25 mg/kg)	(a) Naranjo[b]<br>(b) NA (probable)	85
Chloroquine-induced recurrent psychosis. Sahoo S. Am J Ther. 2007	CQ	Malaria	Organic delusional (schizophrenia-like) disorder (Brief Psychiatric Rating Scale: score of 52/60)	(a) Yes<br>(b) Diazepam IV and injectable antipsychotics followed by oral antipsychotics<br>(c) No<br>(d) No<br>(e) Six months later, the patient was asymptomatic with maintenance of olanzapine (10 mg)	(a) 40<br>(b) Male<br>(c) No history or family psychiatric disorders	(a) 2 days<br>(b) 1.8 g<br>(c) NA	(a) NA<br>(b) NA	74
Two challenge and rechallenge episodes of chloroquine-induced psychotic mania in a patient with rheumatoid arthritis. Plesnicar BK. Akt Rheumatol. 2013	CQ	Reumathoid arthritis	Two mania episodes	(a) Yes<br>(b) Psychiatric hospitalization and risperidone 2 mg/day (first and second episode)<br>(c) Yes<br>(d) Mild attention deficit and memory difficulties<br>(e) Emotionally stable and manic symptoms disappeared	(a) 72<br>(b) Male<br>(c) No psychiatric history. Diabetes mellitus, arterial hypertension and cured prostate cancer	First episode:<br>(a) 2 w<br>(b) 3.5 g<br>(c) 0. 25 g QD<br>Second episode:<br>(a) 2 days<br>(b) 0.5 g<br>(c) 0. 25 g QD	(a) NA<br>(b) NA	66
Exacerbations of bipolar disorder triggered by chloroquine in systemic lupus erythematosus - a case report. Bogaczewicz J. Lupus. 2014	CQ	SLE and arthritis	Exacerbation of bipolar disorder (manic episode	(a) Yes<br>(b) The patient continued the therapy with quetiapine 300 mg/day, lamotrigine 100 mg/day and methylprednisolone 4 mg/day<br>(c) No<br>(d) No	(a) 31<br>(b) Male<br>(c) Neuropsychiatric disorders, depression and aggravation of psychiatric symptoms induced by 3 months of CQ treatment	(a) 4 days<br>(b) 1 g<br>(c) 0.25 g QD	(a) NA<br>(b) NA	100

			with psychotic features)	(e) HCQ was started 200 mg/day and within 1 year no psychiatric symptoms occurred and mood disorder is in remission				
Chloroquine-induced subacute paranoid-like disorder as a complication of dermatological treatment. Bogaczewicz A. Int J Dermatol. 2016	CQ	SLE	Paranoid-like disorder	(a) Yes<br>(b) NA<br>(c) No<br>(d) No<br>(e) All symptoms recovered 2 days after drug discontinuation	(a) 29<br>(b) Female<br>(c) NA	(a) 3 days<br>(b) 0.75 g<br>(c) 0. 25 g QD	(a) Naranjo[b]<br>(b)Authors reported 6 (possible)	83
Chloroquine induced psychosis in an adult patient with amoebic liver abscess: a case report. Choughule A. Indian Journal of Mental Health. 2019	CQ phosphate	Hepatic amebiasis	Psychosis	(a) Yes<br>(b) NA<br>(c) No<br>(d) No<br>(e) NA	(a) 32<br>(b) Male<br>(c) History of alcohol intake	(a) 5 days<br>(b) NA<br>(c) 0. 6 g in divided doses	(a) NA<br>(b) NA	98
A case report of hydroxychloroquine-induced auditory and visual hallucination. Ganjei Z. Int J Clin Pharmacol Ther 2021	HCQ	DLE	Auditory and visual hallucinations, nightmares, occasional decrease in consciousness level	(a) Yes<br>(b) NA<br>(c) NA<br>(d) The hallucinations and other symptoms disappeared within a week after discontinuation of HCQ<br>(e) The patient was followed up for subsequent 3 months and did not show any recurrence	(a) 37<br>(b) Female<br>(c) No history of other diseases, including neurological or dermatological disease. Lack of a history of psychiatric disorders. She had not taken any medication.	(a) A week<br>(b) 1.4 g<br>(c) 0.2 g QD	(a) NA<br>(b) NA	100

Abbreviations: BD: twice a day; CQ: Chloroquine; g: grams; h: hours; HCQ: Hydroxychloroquine; IV: intravenous; mg: milligrams; NA: not available/not applicable; systemic lupus erythematosus; QD: once a day; QID: four times a day; TD: three times a day; w: weeks. DLE: discoid lupus erythematosus

[a]Quality assessment: a higher score indicates a higher quality. (%) [Yes (1) + Partly (0.5)/Total applicable] x100
[b]Naranjo, C.A.; Busto, U.; Sellers, E.M.; Sandor, P.; Ruiz, I.; Robert, E.A.; Janecek, E.; Domecq, C.; Greenblatt, D.J. A method for estimating the probability of adverse drug reaction. Clin. Pharmacol. Ther. 1981, 30, 239–245.

*case extracted from an observational study.

Table S7. Characteristics of included studies: case reports and case series related to neurologic adverse events

Study ID: title, first author, journal of publication, year	Drug	Indication	Adverse reaction reported	Adverse effect management<br>(a) Discontinuation<br>(b) Management<br>(c) Reintroduction<br>(d) Long-term consequences<br>(e) Follow up	Population characteristics:<br>(a) Age (years)<br>(b) Gender<br>(c) Clinical characteristics	Other:<br>(a) Time of appearance<br>(b) Cumulative dose<br>(c) Regimen	Adverse Drug Reaction Probability Scale:<br>(a) Scale<br>(b) Score (probability category)	Quality assess-ment[a] (%)
Chloroquine seizures. Torrey E F. JAMA. 1968	CQ phosphate	Intestinal amebiasis	Seizures	(a) Yes<br>(b) Diphenylhydantoin sodium 100 mg tid and Phenobarbital sodium 16 mg tid<br>(c) No<br>(d) Six months later the EEG tracings were still considered abnormal<br>(e) Both medications have been discontinued with no further seizure	(a) 26<br>(b) Female<br>(c) The patient had no family history of seizure	(a) 12 days<br>(b) 9 g<br>(c) 0.65 g TD for 14 days	(a) NA<br>(b) NA	91
		Hepatic amebiasis	Seizures	(a) Yes<br>(b) Phenobarbital orally<br>(c) Yes<br>(d) No<br>(e) Discharge with no medications and no recurrence	(a) 23<br>(b) Female<br>(c) NA	(a) 6 day<br>(b) 3 g<br>(c) 0.25 g BD for 14 days		
		Hepatic amebiasis	Two seizures episodes	(a) Yes<br>(b) Sedation<br>(c) No<br>(d) No<br>(e) No further seizures	(a) 23<br>(b) Female<br>(c) NA	(a) 2 days<br>(b) 2.25 g<br>(c) 0.5 g BD for two days		
		Intestinal amebiasis	Grand mal seizure	(a) Yes<br>(b) Diphenylhydantoin<br>(c) NA<br>(d) NA<br>(e) NA	(a) 42<br>(b) Female<br>(c) The patient had a brother who had had seizures as a child, but the patient had had none herself	(a) 4 days<br>(b) Uncertain dose, apparently was either 0.75 g or 1 g/day (3 g or 4 g)<br>(c) 0.7 g or 1 g QD for four days		
Chloroquine induced involuntary movements. Umez-Eronini EM. Br Med J. 1977	CQ	Fever	Involuntary movements	(a) Yes<br>(b) Diphenhydramine and methocarbamol<br>(c) No<br>(d) No<br>(e) Symptoms abated during the next 36 h	(a) 26<br>(b) Male<br>(c) No previous history of fits	(a) 1 day<br>(b) 2 tablets<br>(c) 2 tablets	(a) NA<br>(b) NA	34
Neuropsychiatric toxicity of chloroquine. Singh RP. J Indian Med Assoc. 1981	CQ	Malaria	Akathisia and persistent protrusion of the tongue	(a) NA<br>(b) Orphenadrine hydrochloride<br>(c) NA<br>(d) NA<br>(e) Asymptomatic at 3 months	(a) 21<br>(b) Male<br>(c) No previous history of adverse reactions with CQ	(a) 1 day<br>(b) 0.6 g<br>(c) 0.3 g and repeated after 12 h	(a) NA<br>(b) NA	71

			Auditory hallucinations, acute psychotic behaviour, difficulty in swallowing, protusion of the tongue and marked extrapyramidal rigidity	(a) NA<br>(b) Promethazine, procyclidine and thioridazine<br>(c) NA<br>(d) NA<br>(e) Asymptomatic at 3 months	(a) 21<br>(b) Male<br>(c) NA	(a) 2 days<br>(b) 1.2 g<br>(c) 0.3 g BD for 7 days	(a) NA<br>(b) NA	
Convulsions associated with prophylactic antimalarial drugs: implications for people with epilepsy. Fish DR. BMJ. 1988	CQ phosphate	Malaria prophylaxis	Serious tonic-clonic convulsion	(a) Yes<br>(b) NA<br>(c) No<br>(d) Serious consequences<br>(e) NA	(a) 49<br>(b) Female<br>(c) Complex partial seizures well controlled and one previous generalised convulsion	(a) 1 day<br>(b) 0.4 g<br>(c) 0.4 g	(a) NA<br>(b) NA	50
Cerebellar ataxia in patients with malaria treated with chloroquine. James RF. Postgrad Med J. 1988	CQ	Malaria	Severe cereberal ataxia with extrapyramidal movements	(a) NA<br>(b) NA<br>(c) No<br>(d) No<br>(e) Completely recovery after 6-8 w	(a) 35, 40, 42<br>(b) Male<br>(c) No history of numbness in the peripheries. No alcoholics. No chemicals or medications	(a) 2 w<br>(b) NA<br>(c) NA	(a) NA<br>(b) NA	45
Transient global amnesia following ingestion of chloroquine. Cras P. J Neurol Neurosurg Psychiatry. 1990	CQ	Malaria prophylaxis	Transient global amnesia	(a) Yes<br>(b) NA<br>(c) No<br>(d) No<br>(e) NA	(a) 62<br>(b) Male<br>(c) Healthy	(a) 3 h<br>(b) 0.3 g<br>(c) 0.3 g	(a) NA<br>(b) NA	57
Persisting chloroquine-induced myasthenia? De Bleecker J. Acta Clin Belg. 1991	CQ	Malaria	Retinopathy and persisting mild ocular myasthenia	(a) Yes<br>(b) NA<br>(c) NA<br>(d) Symptoms persisted more than 10 years after drug discontinuation<br>(e) NA	(a) 53<br>(b) Female<br>(c) The patient took intermittently CQ for treatment of malaria. History of CQ-induced retinopathy, ocular myasthenic symptoms starting during CQ treatment and cardiac left anterior hemiblock and total right bundle branch block	(a) NA (9-day scheme of 4.5 g three to four times a year from 1958 to 1979)<br>(b) NA<br>(c) 0.9 g on the 1st day, decreasing the dose by 100 mg each day with cessation	(a) NA<br>(b) NA	80

						after the 9th day, three or four times a year		
Seizures associated with chloroquine therapy. Adamolekun B. Cent Afr J Med. 1992	CQ base	Suspected malaria	Tonic-clonic seizures	(a) Yes<br>(b) No<br>(c) No<br>(d) No<br>(e) Seizure free at 3 years follow up	(a) 18<br>(b) Female<br>(c) Previous episode of seizures taking CQ. No aetiological risk factors for epilepsy. There was no familiar history of seizures	(a) 8 h<br>(b) 0.6 g<br>(c) Four tablets (0.6 g bases) in a starting dose	(a) NA<br>(b) NA	84
Chloroquine and nonconvulsive status epilepticus. Mülhauser P. Ann Intern Med. 1995	CQ base	Malaria prophylaxis	Non-convulsive status epilepticus	(a) Yes<br>(b) Carbamazepine<br>(c) No<br>(d) No<br>(e) Remained free of symptoms with normal electroencephalograms 10 days and 6 months after antiepileptic drug discontinuation	(a) 68<br>(b) Female<br>(c) Healthy without history of epilepsy or alcoholism	(a) 12 days<br>(b) 1.2 g<br>(c) 0.1 g QD	(a) NA<br>(b) NA	73
Seizures following chloroquine treatment of type II lepra reaction: a case report. Ebenso BE. Lepr Rev. 1998	CQ base	Erythema nodosum leprosum	Three episodes of generalized tonic-clonic seizures	(a) No<br>(b) Phenytoin<br>(c) NA<br>(d) No<br>(e) Remained seizure free after slowly phenytoin weaning off	(a) 40<br>(b) Male<br>(c) NA	(a) 9 days<br>(b) 3.45 g<br>(c) 0.15 g TD for the first week; 0.15 g BD the second week; 0.15 g QD the third week	(a) NA<br>(b) NA	83
Chloroquine-induced bilateral anterior shoulder dislocation: a unique aetiology for a rare clinical problem. Martin AN. BMJ Case Reports. 2016	CQ	Prophylactic treatment of gastrointestinal parasitic infection	Seizures resulting in bilateral anterior shoulder dislocations	(a) Yes<br>(b) The patient underwent closed reduction of his dislocated shoulders under sedation<br>(c) No<br>(d) No<br>(e) No further neurological follow-up was arranged. Normal physical activity	(a) 30<br>(b) Male<br>(c) No comorbidities	(a) 4 days<br>(b) 4 g<br>(c) 0.5 g BD for the past 4 days	(a) NA<br>(b) NA	95
Psychomotor agitation following treatment with hydroxychloroquine. Manzo C. Drug Saf Case Rep. 2017	HCQ	Rheumatoid arthritis	Significant psychomotor agitation	(a) Yes<br>(b) Promazine 25 mg IM<br>(c) Yes<br>(d) The agitation ceased and there were no relapses or sequelae. The agitation appeared again when reintroducing HCQ. Promazine 30 mg was administered and HCQ was stopped again<br>(e) One year later, the patient presented with neither cognitive impairment nor psychosis, with no further episodes of agitation	(a) 80<br>(b) Female<br>(c) Familiar hypercholesterolemia (pravastatin 20 mg/day), high blood pressure (amlodipine 5 mg/day) and non-hemodynamically significant carotid atheromasia (acetylsalicylic acid 100 mg/day). No psychiatric history and no family history of psychiatric problems	(a) 10 days<br>(b) 2.0 g<br>(c) 0.2 g QD	(a) Naranjo[b]<br>(b) 9 (definite)	84

Abbreviations: BD: twice a day; CQ: Chloroquine; tid: three times a day; g: grams; h: hours; HCQ: Hydroxychloroquine; IM: intramuscular; mg: milligrams; NA: not available/not applicable; QD: once a day; TD: three times a day; w: weeks.

[a]Quality assessment: a higher score indicates a higher quality. (%) [Yes (1) + Partly (0.5)/Total applicable] x100
[b]Naranjo, C.A.; Busto, U.; Sellers, E.M.; Sandor, P.; Ruiz, I.; Robert, E.A.; Janecek, E.; Domecq, C.; Greenblatt, D.J. A method for estimating the probability of adverse drug reaction. Clin. Pharmacol. Ther. 1981, 30, 239–245.

Table S8. Characteristics of included studies: case reports and case series related to cardiac adverse events

**Study ID: title, first author, journal of publication, year**	**Drug**	**Indication**	**Adverse reaction reported**	**Adverse effect management (a) Discontinuation (b) Management (c) Reintroduction (d) Long-term consequences (e) Follow up**	**Population characteristics: (a) Age (years) (b) Gender (c) Clinical characteristics**	**Other: (a) Time of appearance (b) Cumulative dose (c) Regimen**	**Adverse Drug Reaction Probability Scale: (a) Scale (b) Score (probability category)**	**Quality assessment[a] (%)**
Cardiovascular collapse following small dose of chloroquine in healthy young adult. Sogani RK. J Assoc Physicians India. 1986	CQ	Dermatologic problem	Cardiovascular collapse	(a) Yes<br>(b) NA<br>(c) No<br>(d) NA<br>(e) NA	(a) 24<br>(b) NA<br>(c) Previous episode of abrupt feeling of dizziness after CQ ingestion	(a) 3 days<br>(b) 1.5 g<br>(c) 0.25 g BD	(a) NA<br>(b) NA	62
Chloroquine and cardiac arrhythmia: case report. Siqueira-Batista R. East Afr Med J. 1998	CQ	Malaria	Cardiac arrhytmia	(a) NA<br>(b) No, only medical evaluation<br>(c) NA<br>(d) No<br>(e) Outpatient follow up with none abnormality	(a) 62<br>(b) Male<br>(c) NA	(a) 56 h<br>(b) 1.5 g<br>(c) 0.6 g initially and then 0.3 g 6,24,48h after the first dose	(a) NA<br>(b) NA	60
Complete heart block in an adult with systemic lupus erythematosus and recent onset of hydroxychloroquine therapy. Comín-Colet J. Lupus. 2001	HCQ	SLE	Complete heart block	(a) Yes<br>(b) A temporary pacemaker (48 h) was inserted and prednisolone was started<br>(c) No<br>(d) No<br>(e) Two years later the patient remains asymptomatic	(a) 40<br>(b) Female<br>(c) No history of cardiopathy	(a) 2 days<br>(b) 0.8 g<br>(c) 0.2 g BD	(a) NA<br>(b) NA	83
Implanted pacemaker failure caused by the antirheumatic	HCQ sulphate	Rheumatoid arthritis	Implanted	(a) Yes<br>(b) Reuse of prednisolone 5 mg/day<br>(c) No	(a) 67<br>(b) Female	(a) 7 days<br>(b) 2.8 g<br>(c) 0.2 BD	(a) NA<br>(b) NA	79

drug hydroxychloroquine. Huang PH. Lupus. 2003			pacemaker failure	(d) No<br>(e) Recovery of pacing threshold one month later	(c) Pacemaker due to third-degree atrioventricular block implanted for 2 years			
Syncope in a patient being treated for hepatic and intestinal amoebiasis. Yelve K. BJM Case Reports. 2012	CQ	Hepatic and intestinal amebiasis	Syncopal attacks and torsade de pointes	(a) Yes<br>(b) Amiodarone and magnesium sulphate drips<br>(c) No<br>(d) No<br>(e) After 6 months the patient did not have any episodes of syncope and torsade	(a) 63<br>(b) Male<br>(c) No comorbidities	(a) 11 days<br>(b) NA<br>(c) NA	(a) NA<br>(b) NA	97
Suspected hydroxychloroquine-associated QT-interval prolongation in a patient with systemic lupus erythematosus. Morgan ND. J Clin Rheumatol. 2013	HCQ	SLE	QT-interval prolongation	(a) Yes<br>(b) Magnesium sulphate IV<br>(c) No<br>(d) Cardioverter defibrillator<br>(e) QT relatively normal after a year	(a) 41<br>(b) Female<br>(c) Congestive heart failure with systolic left ventricular dysfunction, hypertension, chronic kidney disease stage 5	(a) 1 w<br>(b) 2.8 g (previous poor compliance for several months)<br>(c) 0.2 g BD	(a) NA<br>(b) NA	77

Abbreviations: BD: twice a day; CQ: Chloroquine; g: grams; h: hours; HCQ: Hydroxychloroquine; mg: milligrams; NA: not available/not applicable; SLE: systemic lupus erythematosus; w: weeks.

[a]Quality assessment: a higher score indicates a higher quality. (%) [Yes (1) + Partly (0.5)/Total applicable] x100

Table S9. Characteristics of included studies: case reports and case series related to hematologic and metabolic adverse events

Study ID: title, first author, journal of publication, year	Drug	Indication	Adverse reaction reported	Adverse effect management<br>(a) Discontinuation<br>(b) Management<br>(c) Reintroduction<br>(d) Long-term consequences<br>(e) Follow up	Population characteristics:<br>(a) Age (years)<br>(b) Gender<br>(c) Clinical characteristics	Other:<br>(a) Time of appearance<br>(b) Cumulative dose<br>(c) Regimen	Adverse Drug Reaction Probability Scale:<br>(a) Scale<br>(b) Score (probability category)	Quality assess-ment[a] (%)
Hypoglycemia: an unusual adverse reaction to chloroquine. Abu-Shakra M. Clin Exp Rheumatol. 1994	CQ	Psoriatic arthritis	Two episodes of hypoglycaemia	(a) Yes<br>(b) NA<br>(c) No<br>(d) No<br>(e) No further symptoms of hypoglycaemia	(a) 56<br>(b) Male<br>(c) Hemorrhagic gastric ulcers surgically treated with vagotomy and pyloroplasty	First episode:<br>(a) Soon afterwards<br>(b) NA<br>(c) 0.25 g QD<br>Second episode:<br>(a) 21 days<br>(b) 5.25 g<br>(c) 0.25 g QD	(a) NA<br>(b) NA	77
Hypoglycemia induced by hydroxychloroquine in a type II diabetic treated for polyarthritis. Shojania K. J Rheumatol. 1999	HCQ	Rheumatoid polyarthritis	Hypoglicemic coma	(a) No<br>(b) Dose reduction of HQC and insulin<br>(c) NA<br>(d) No<br>(e) No further hypoglycaemic symptoms	(a) 77<br>(b) Male<br>(c) Type II diabetes, dry eyes, dry mouth, benign prostatic hypertrophy and mild chronic asthma	(a) 7 days<br>(b) 2.8 g<br>(c) 0.4 g QD	(a) NA<br>(b) NA	83
Hydroxychloroquine as a glucose lowering drug. Winter EM. BMJ Case Rep. 2011	HCQ	Osteoarthritis	Hypogly-caemia	(a) Yes<br>(b) Glucose 50% IV<br>(c) No<br>(d) No<br>(e) Follow-up consists of 24 months now, with no further incidents reported	(a) 80<br>(b) Female<br>(c) Patient non-diabetic, mild renal insufficiency, M-protein of unknown significance, hypertension and one event of acute coronary syndrome	(a) The patient appeared to take HCQ since 4 months, with all four reported hypoglycaemic incidents within this time window<br>(b) NA<br>(c) 0.4 g QD	(a) NA<br>(b) NA	93
Acute life-threatening methaemoglobinaemia following ingestion of chloroquine. Rizvi I. BMJ Case Rep. 2012	CQ base	Fever	Life-threatening methae-moglobi-naemia	(a) NA<br>(b) Methylene blue 60 mg IV<br>(c) NA<br>(d) No<br>(e) He was discharged from the hospital next day in stable condition	(a) 25<br>(b) Male<br>(c) Without any history of chronic illness with average built and fair coloured skin	(a) Found unconscious after taking two CQ tablets<br>(b) 0.6 g<br>(c) 0.6 g	(a) NA<br>(b) NA	86
A case of thrombocytopenia associated with the use of hydroxychloroquine following open heart surgery. Demir D. Int J Surg Case Rep. 2014	HCQ	Used erroneously as a pain killer	Thrombocytopenia	(a) Yes<br>(b) Steroid therapy<br>(c) No<br>(d) No<br>(e) After 3 months the patient was healthy	(a) 55<br>(b) Male<br>(c) Coronary artery disease	(a) 7 days<br>(b) NA<br>(c) NA	(a) NA<br>(b) NA	93
Suspected hydroxychloroquine-induced thrombotic	HCQ	Rheumatoid arthritis	Thrombotic thrombocytopaenic purpura	(a) NA<br>(b) Ceftriaxone, vancomycin, piperacillin/tazobactam, acyclovir, fluconazole, midazolam, phenytoin,	(a) 64<br>(b) Female<br>(c) Osteoporosis, hypertension, undiagnosed right lung mass and cerebral	(a) 3 days<br>(b) 0.6 g<br>(c) 0.2 g QD	(a) Naranjo[b]<br>(b) NA (possible)	83

thrombocytopaenic purpura. Fromm LM. J Pharm Prac. 2018				hydrocortisone, IV furosemide, noradrenaline, propofol, fentanyl, 4% albumin and plasma exchange<br>(c) NA<br>(d) Death related to cardiac failure (primary diagnosis) with TTP (secondary diagnosis)<br>(e) NA	palsy secondary to encephalitis, neurological deficit but not seizure history			

Abbreviations: CQ: Chloroquine; g: grams; HCQ: Hydroxychloroquine; IV: intravenous; mg: milligrams; NA: not available/not applicable; QD: once a day.

[a]Quality assessment: a higher score indicates a higher quality. (%) [Yes (1) + Partly (0.5)/Total applicable] x100
[b]Naranjo, C.A.; Busto, U.; Sellers, E.M.; Sandor, P.; Ruiz, I.; Robert, E.A.; Janecek, E.; Domecq, C.; Greenblatt, D.J. A method for estimating the probability of adverse drug reaction. Clin. Pharmacol. Ther. 1981, 30, 239–245.

Table S10. Characteristics of included studies: case reports and case series related to sense organs adverse events

Study ID: title, first author, journal of publication, year	Drug	Indication	Adverse reaction reported	Adverse effect management<br>(a) Discontinuation<br>(b) Management<br>(c) Reintroduction<br>(d) Long-term consequences<br>(e) Follow up	Population characteristics:<br>(a) Age (years)<br>(b) Gender<br>(c) Clinical characteristics	Other:<br>(a) Time of appearance<br>(b) Cumulative dose<br>(c) Regimen	Adverse Drug Reaction Probability Scale:<br>(a) Scale<br>(b) Score (probability category)	Quality assess-ment[a] (%)
Diplopia and loss of accommodation due to chloroquine. Rubin ML. Arthritis Rheum. 1970	CQ	Hypercalcemia associated with sarcoidosis	Occasional diplopia and persistent blurred near vision	(a) Yes<br>(b) NA<br>(c) NA<br>(d) No<br>(e) Ocular function returned to pretreatment limits 1 w after discontinuing CQ	(a) 50<br>(b) Female<br>(c) Splenectomy. No ocular abnormalities	(a) 14 days<br>(b) 7.0 g<br>(c) 0.5 g QD	(a) NA<br>(b) NA	77
Hydroxychloroquine-induced vertigo. Prince DS. JAMA. 1975	HCQ sulphate	Rheumatoid arthritis	Severe positional vertigo	(a) Yes<br>(b) Bedrest and antiemetics<br>(c) Yes<br>(d) Bedrest and antiemetics produced complete resolution of all symptoms in 72 h, but 48 h after reintroducing HCQ sulphate severe vertigo and headache recurred, and the therapy was discontinued<br>(e) All symptoms disappeared within 36 h	(a) 54<br>(b) Female<br>(c) NA	(a) 3 days<br>(b) 1.2 g<br>(c) 0.4 g QD	(a) NA<br>(b) NA	68
Chloroquine causing vestibular toxicity. Malik MK. Indian J Otolaryngol. 1977	HCQ	Malaria	Severe vestibular toxicity	(a) Yes<br>(b) NA<br>(c) NA<br>(d) Bilateral complete canal paresis<br>(e) NA	(a) 35<br>(b) Male<br>(c) NA	(a) 5-6 h<br>(b) 1 g<br>(c) 1 g	(a) NA<br>(b) NA	40
	CQ		Vestibular toxicity	(a) NA<br>(b) NA<br>(c) NA<br>(d) Bilateral complete canal paresis<br>(e) NA	(a) 23<br>(b) Male<br>(c) NA	(a) 3 days<br>(b) 2.5 g<br>(c) 2.5 g spread over 3 days	(a) NA<br>(b) NA	
Ototoxicity of chloroquine phosphate. A case report. Dwivedi GS. J Laryngol Otol. 1978	CQ phosphate	Malaria	Loss of hearing	(a) NA<br>(b) NA<br>(c) No<br>(d) Any improvement at 5.5 months<br>(e) NA	(a) 52<br>(b) Male<br>(c) Without previous complaints of ear troubles and use of ototoxic drugs	(a) 1.5 h<br>(b) 1 g<br>(c) 1 g	(a) NA<br>(b) NA	43
Reversible ageusia as an adverse effect of hydroxychloroquine treatment. Fleury O. J Eur	HCQ	SLE	Complete ageusia	(a) Yes<br>(b) NA<br>(c) No<br>(d) No	(a) 28<br>(b) Female<br>(c) Arthritis, sicca symptoms, photosensitivity and Raynaud's phenomenon during infancy	(a) NA (quickly)<br>(b) NA<br>(c) 0.4 g QD	(a) NA<br>(b) NA	74

Acad Dermatol Venereol. 2009				(e) Ageusia disappeared after removal of HCQ. Five years later CQ was started and well supported without loss of taste				

Abbreviations: CQ: Chloroquine; g: grams; h: hours; HCQ: Hydroxychloroquine; NA: not available/not applicable; SLE: systemic lupus erythematosus; QD: once a day; w: weeks.

[a]Quality assessment: a higher score indicates a higher quality. (%) [Yes (1) + Partly (0.5)/Total applicable] x100

Table S11. Characteristics of included studies: case reports and case series related to hepatic adverse events

Study ID: title, first author, journal of publication, year	Drug	Indication	Adverse reaction reported	Adverse effect management (a) Discontinuation (b) Management (c) Reintroduction (d) Long-term consequences (e) Follow up	Population characteristics: (a) Age (years) (b) Gender (c) Clinical characteristics	Other: (a) Time of appearance (b) Cumulative dose (c) Regimen	Adverse Drug Reaction Probability Scale: (a) Scale (b) Score (probability category)	Quality assess-ment[a] (%)
Severe acute hepatitis related to hydroxychloroquine in a woman with mixed connective tissue disease. Giner Galvañ V. Clin Rheumatol. 2007	HCQ	Arthritis	Severe acute hepatitis	(a) Yes<br>(b) Methylprednisolone 60 mg/day IV and ceftriaxone 1 g/day IV<br>(c) No<br>(d) No<br>(e) Complete normalization of analytical parameters	(a) 26<br>(b) Female<br>(c) Raynaud's phenomena	(a) 8 to 10 h after the first dose<br>(b) 0.2 g<br>(c) 0.2 g QD	(a) Naranjo[b]<br>(b) 7 (probable)	92
The devil's in the dosing: severe drug-induced liver injury in a hydroxychloroquine-naive patient with subacute cutaneous lupus erythematosus and porphyria cutanea tarda. Sunkara B. Lupus. 2018	HCQ	Subacute cutaneous lupus erythe-matosus	Liver injury	(a) Yes<br>(b) The adverse reaction forced the authors to revise their original diagnosis of subacute cutaneous lupus erythematosus starting with the nature of her bullous skin condition. Biopsy of her bullous skin lesions was consistent with porphyria cutanea tarda. Therapeutic phlebotomy and HQC permanent discontinuation<br>(c) No<br>(d) NA<br>(e) Marked improvement in liver enzyme abnormalities returning to baseline	(a) 29<br>(b) Female<br>(c) Previously healthy	(a) 3 days<br>(b) 1.2 g<br>(c) 0.4 g QD	(a) NA<br>(b) NA	91
Hepatotoxic reaction to chloroquine phosphate in a patient with previously unrecognized porphyria cutanea tarda. Liu AC. West J Med. 1995	CQ phosphate	Malaria prophylaxis	Hepatotoxic reaction	(a) Yes<br>(b) NA<br>(c) No<br>(d) No<br>(e) Finally diagnosed with porphyria cutanea tarda, resolution of hepatotoxic reaction	(a) 61<br>(b) Female<br>(c) Unrecognized porphyria cutanea tarda, hysterectomy, Quervain's thyroiditis and menopausal symptoms. No history of hepatitis or blood transfusion	(a) 1 day<br>(b) 0.5 g<br>(c) 0.5 g single dose	(a) NA<br>(b) NA	80
Fulminant hepatic failure secondary to hydroxychloroquine. Makin AJ. Gut. 1994	HCQ	SLE	Fulminant hepatic failure	(a) NA<br>(b) Transplant proposed<br>(c) NA<br>(d) Death<br>(e) NA	(a) 27<br>(b) Female<br>(c) No previous liver disease	(a) 14 days<br>(b) 5.6 g<br>(c) 0.2 g BD	(a) NA<br>(b) NA	81
Bullous rash and brown urine in a systemic lupus erythematosus patient treated with	HCQ	SLE	Bullous rash and acute hepatitis	(a) NA<br>(b) NA<br>(c) NA<br>(d) No	(a) 36<br>(b) Female<br>(c) Unrecognized porphyria cutanea tarda	(a) 7 days<br>(b) 2.8 g<br>(c) 0.4 g QD	(a) NA<br>(b) NA	55

hydroxychloroquine. Kutz DC. Arthritis Rheum. 1995				(e) Finally diagnosed with porphyria cutanea tarda				

Abbreviations: BD: twice a day; CQ: Chloroquine; g: grams; h: hours; HCQ: Hydroxychloroquine; IV: intravenous; mg: milligrams; NA: not available/not applicable; SLE: systemic lupus erythematosus; QD: once a day.

[a]Quality assessment: a higher score indicates a higher quality. (%) [Yes (1) + Partly (0.5)/Total applicable] x100

[b]Naranjo, C.A.; Busto, U.; Sellers, E.M.; Sandor, P.; Ruiz, I.; Robert, E.A.; Janecek, E.; Domecq, C.; Greenblatt, D.J. A method for estimating the probability of adverse drug reaction. Clin. Pharmacol. Ther. 1981, 30, 239–245.

Table S12. Characteristics of included studies: case reports and case series related to other adverse events

**Study ID: title, first author, journal of publication, year**	**Drug**	**Indication**	**Adverse reaction reported**	**Adverse effect management (a) Discontinuation (b) Management (c) Reintroduction (d) Long-term consequences (e) Follow up**	**Population characteristics: (a) Age (years) (b) Gender (c) Clinical characteristics**	**Other: (a) Time of appearance (b) Cumulative dose (c) Regimen**	**Adverse Drug Reaction Probability Scale: (a) Scale (b) Score (probability category)**	**Quality assess-ment[a] (%)**
Porphyria precipitated by hydroxychloroquine treatment of systemic lupus erythematosus. Baler GR. Cutis. 1976	HCQ	SLE	Porphyria variegata precipitation	(a) NA(b) No specific therapy (c) NA (d) NA (e) At 4 years follow up the patient was free of all photosensitivity, uroporphyrins remained negative and porphobilinogen gradually became negative	(a) 48 (b) Female (c) Previous discontinuation of CQ due to eye symptoms and HQC due to persistent diarrhoea	(a) 36 h (b) 0.6 g (c) 0.2 g BD	(a) NA (b) NA	80
Toxicity related to chloroquine treatment of resistant vivax malaria. Davis TM. Ann Pharmacother. 2003	CQ phosphate	Malaria	CQ overdose with severe headache, dizziness on standing, nausea, and blurred vision (intake < 5 times the DDD of 0.5 g of CQ base)	(a) No (b) NA (c) NA (d) Unresolved malaria that required treatment with atovaquone, proguanil and primaquine (e) NA	(a) 36 (b) Male (c) No past history of any other illness and never had malaria	(a) During the treatment with CQ 0.6 g tid for 7 days (b) 12.6 g CQ phosphate (c) 0.6 g TD for 7 days	(a) NA (b) NA	66
Differential diagnosis of high serum creatine kinase levels in systemic lupus erythematosus. Richter JG. Rheumatol Int. 2003	CQ	SLE with arthralgia and renal involvement	Severe myopathy	(a) Yes (b) Calcium and thyroid hormones supplementation (c) No (d) NA (e) Serum creatine kinase levels returned to the normal range	(a) 57 (b) Female (c) Mixed connective tissue disease, Fahr’s syndrome and hypothyroidism after thyroidectomy because of Graves’ disease	(a) When reintroducing CQ (b) NA (c) NA	(a) NA (b) NA	77
Hydroxychloroquine causes severe vacuolar myopathy in a patient with chronic graft-versus-host disease. Bolaños-Meade J. Am J Hematol. 2005	HCQ sulphate	cGVHD	Severe vacuolar myopathy	(a) Yes (b) NA (c) No (d) No (e) Improvement in strength and function	(a) 51 (b) Male (c) Allogeneic bone marrow transplant for mantle cell lymphoma	(a) 14 days (b) 11.2 g (c) 0.4 g BD	(a) NA (b) NA	79

A rare cause of acute respiratory failure and elevated eosinophils in broncho-alveolar lavage fluid. Knudsen L. Respiration. 2009	CQ	Mild rosacea	Acute eosino-philic pneumo-nitis	(a) Yes<br>(b) Clarithromycin, prednisolone 40 mg/day for 7 days, budesonide/formoterol for 4 w<br>(c) No<br>(d) NA<br>(e) In follow-up consultations, the lung function normalized again	(a) 41<br>(b) Female<br>(c) Previously healthy	(a) 13 days<br>(b) 3.25 g<br>(c) 0.25 g QD	(a) NA<br>(b) NA	89
Successful desensitization for hydroxychloroquine anaphylaxis. Donado CD. Rheumatol. 2010	HCQ	SLE	Anaphyla xis	(a) Yes<br>(b) Required attention in the emergency room<br>(c) Yes, after successful HCQ desensitization<br>(d) No<br>(e) At the time of writing the patient continues to tolerate HCQ without incident	(a) 48<br>(b) Male<br>(c) NA	(a) Coinciding with resumption of treatment<br>(b) NA<br>(c) 0.2 g	(a) NA<br>(b) NA	84
A case of urinary incontinence by hydroxychloroquine in a geriatric patient. Carnovale C. J Clin Pharm Ther. 2013	HCQ	Rheumat oid arthritis	Two episodes of urinary incontine nce	(a) Yes<br>(b) No<br>(c) Yes<br>(d) Urinary incontinence recurred<br>(e) The second discontinuation of HCQ led to resolution of incontinence	(a) 71<br>(b) Female<br>(c) Hypercholesterolaemia and myocardial infarction	First episode:<br>(a) NA<br>(b) NA<br>(c) 0.25 g QD<br>Second episode:<br>(a) 2 days<br>(b) 0.5 g<br>(c) 0.25 g QD	(a) Naranjo[b]<br>(b) 6 (probable)	100
Interstitial lung disease induced by hydroxychloroquine. Català R. Med Clin (Barc). 2015	HCQ	Polymorp hic light eruption	Diffuse interstitia l lung disease	(a) Yes<br>(b) Oxygen therapy and methylprednisolone 40 mg tid<br>(c) No<br>(d) No<br>(e) After 3 years the patient is asymptomatic without evidence of any lung disease	(a) 36<br>(b) Female<br>(c) Hypertriglyceridemia and Wolff-Parkinson-White syndrome	(a) 1 day<br>(b) 0.4 g<br>(c) 0.2 g BD	(a) Naranjo[b]<br>(b) 6 (probable)	89
Drug-induced acute eosinophilic pneumonia due to hydroxychloroquine in a chilblain lupus patient. Ishiguro Y. J Dermatol. 2019	HCQ	Chilblain lupus erythema tosus	Acute eosinophi -lic pneumo-nia	(a) Yes<br>(b) Prednisolone 20 mg/day<br>(c) No<br>(d) NA<br>(e) Skin lesions improved and resolution of the lung lesions	(a) 69<br>(b) Female<br>(c) NA	(a) 14 days<br>(b) 2.8 g<br>(c) 0.2 g QD	(a) Allen[c]<br>(b) 4 (possible)	81

Abbreviations: BD: twice a day; cGVHD: chronic graft-versus-host disease; CQ: Chloroquine; DDD: defined daily dose; tid: three times a day; g: grams; h: hours; HCQ: Hydroxychloroquine; mg: milligrams; NA: not available/not applicable; QD: once a day; SLE: systemic lupus erythematosus; TD: three times a day; w: weeks.

[a]Quality assessment: a higher score indicates a higher quality. (%) [Yes (1) + Partly (0.5)/Total applicable] x100

[b]Naranjo, C.A.; Busto, U.; Sellers, E.M.; Sandor, P.; Ruiz, I.; Robert, E.A.; Janecek, E.; Domecq, C.; Greenblatt, D.J. A method for estimating the probability of adverse drug reaction. Clin. Pharmacol. Ther. 1981, 30, 239–245.

[c]Allen JN. Drug-induced eosinophilic lung disease. Clin Chest Med 2004; 25: 77–88.

Table S13. Characteristics of included studies: observational studies, patients affected by malaria or who received prophylactic treatment.

Study ID: title, first author, journal of publication, year	Drug	Sample Size and study groups	Age and Gender	Drug Indication	Aim	Patient Inclusion and Exclusion Criteria	Main study characteristics: (a) Epidemiological approach (b) Data gathering (c) Time horizon (d) Time frame (e) Location	Adverse event characteristics: (a) Measure	Other: (a) Time of appearance (b) Cumulative dose (c) Regimen	Quality assessment[a] (%)
Epidemiology of antimalarial-induced pruritus in Africans. Ajayi AA. Eur J Clin Pharmacol. 1989	CQ	1100 (survey respondents)	(a) NA<br>(b) NA	Malaria	To assess the epidemiological characteristics of antimalarial-induced pruritus in Africans	Inclusion criteria:<br>- Respondents who had had antimalarial chemotherapy in the preceding 4 months	(a) Cross-sectional study<br>(b) NA<br>(c) NA<br>(d) NA<br>(e) Nigeria	(a) Pruritus:<br>74% had pruritus, of them 61% took CQ alone	(a) 6-24 h after CQ. The duration of pruritus was more than 2 days in 42% of patients<br>(b) NA<br>(c) NA	91
Chloroquine and psoriasis. Katugampola G. Int J Dermatol. 1990	CQ	50	NA	Malaria prophylaxis and treatment	Effect of CQ on the course of psoriasis	Inclusion criteria:<br>- Settlers affected by psoriasis	(a) Cohort study<br>(b) Retrospective<br>(c) 3 months<br>(d) NA<br>(e) Sri Lanka	(a) Worsening of psoriasis:<br>6 (12)[c]	(a) 14 days<br>(b) NA<br>(c) NA	29
Acute reactions to chloroquine phosphate at the beginning of antimalaria chemoprophylaxis. Frías Salcedo JA. Rev Sanid. Mil. 1992	CQ phosphate	972	NA	Malaria prophylaxis	Acute ADRs due to malaria prophylaxis with CQ phosphate	Inclusion criteria:<br>- Military who participated in the Mars XVI task force in the Sinaloa, Durango and Chihuahua area	(a) NA<br>(b) NA<br>(c) NA<br>(d) NA<br>(e) NA	(a) Visual and gastrointestinal disturbances or pruritus:<br><2.5%<br>Moderate and transient headache:<br>24 (2.5)[c]	(a) NA<br>(b) NA<br>(c) NA	NA
Malaria therapy in 452 patients, with special reference to the use of quinine. Weinke T. J Infect. 1992	CQ	452<br>Regimens:<br>- CQ: 286<br>- Quinine: 100<br>- Mefloquine: 47<br>- Pyrimethamine + sulphadoxine: 11<br>- Halofantrine: 8	Age (years):<br>32.2 (12-78)[f]<br>Gender (male):<br>72.3%	Malaria	To assess the efficacy and toxic potential of antimalarial therapy regimens	Inclusion criteria:<br>- Patients who had plasmodia in blood films, clinical signs of malaria and clinical documentation available	(a) Cohort study<br>(b) Retrospective<br>(c) NA<br>(d) 1980 to 1990<br>(e) Germany	(a) 301 patients with CQ presented side effects:<br>Pruritus:<br>3.3% (dark-skinned patients 9.7%)<br>Abdominal pain:<br>4.7%<br>Tinnitus:<br>0.7%<br>Vertigo:<br>0.3%<br>Headache:<br>0.3%	(a) 2 days<br>(b) 1.5 g<br>(c) CQ 0.6 g followed by 0.3 g at 6, 24 and 48 h	48

Knowledge, attitude and practice of prednisolone prevention of chloroquine induced pruritus among Nigerian health workers. Ajayi AA. Trop Doct. 1998	CQ base	117 (questionnaire respondents)	<u>Age:</u> 25 to 34: 50%<br><br><u>Gender (male):</u> 58 (49.6)[c]	Malaria	To assess the knowledge, attitude and practice of prednisone prevention on CQ induced pruritus	<u>Inclusion criteria:</u><br>- Nigerian hospital workers and their families historically established as CQ pruritus reactors	(a) Cross-sectional study<br>(b) NA<br>(c) NA<br>(d) NA<br>(e) Nigeria	(a) <u>Pruritus:</u><br>105 (90)[c] had a positive family history of CQ pruritus<br>(	(a) NA<br>(b) 2.1 g<br>(c) 2.1 g of CQ base over a 3 day period	57
Frequency of pruritus in Plasmodium vivax malaria patients treated with chloroquine in Thailand. Bussaratid V. Trop Doct. 2000	CQ	1189	<u>Age (years, mean):</u><br>Pruritus group:<br>26.7<br>Non- pruritus group:<br>NA<br><br><u>Gender (female):</u><br>422 (35.49)[c]	Malaria	To assess the frequency and severity of CQ induced pruritus in an Asian population and the effect on patients compliance	<u>Inclusion criteria:</u><br>- Patients with *Plasmodium vivax* malaria admitted to Bangkok Hospital for Tropical Diseases<br><u>Exclusion criteria:</u><br>- Patients who received any medication in addition to CQ, primaquine or an antihistamine	(a) Cohort study<br>(b) Retrospective<br>(c) Hospital admission<br>(d) 1992 to 1997<br>(e) Thailand	(a) <u>Pruritus:</u><br>23 (1.9%)[c]	(a) 12-96 h after first dose<br>(b) 1.5 g<br>(c) 0.6 g at admission followed by 0.3 g at 6, 24 and 48 h	43
Pattern of chloroquine-induced pruritus in antenatal patients at the University College Hospital, Ibadan. Olayemi O. J Obstet Gynaecol. 2003	CQ	200 (questionnaire respondents)	<u>Age:</u><br>Pruritus group:<br>30.2 (4.22)[b]<br>Non-pruritus group:<br>30.25 (4.96)[b]<br><br><u>Gender (female):</u><br>100%	Malaria	To assess the pattern of CQ induced pruritus in antenatal patients	<u>Inclusion criteria:</u><br>- Consecutive antenatal patients at the University College Hospital of Ibadan treated with CQ	(a) Cross-sectional study<br>(b) NA<br>(c) NA<br>(d) NA<br>(e) Ibadan	(a) <u>Pruritus:</u><br>129 (64.5)[c]<br><u>Generalized pruritus with insomnia:</u><br>54 (41.8)[c]	(a) <6 h: 39 (30.2)[c]<br>6-12 h: 21 (16.3)[c]<br>13-24 h: 39 (30.2)[c]<br>>24 h: 30 (23.3)[c]<br>(b) NA<br>(c) NA	48
Chloroquine induced pruritus - questionnaire based epidemiological study. George AO. Afr J Health Sci. 2004	CQ	63 (questionnaires suitable for evaluation)	<u>Age (years):</u><br>General population:<br>24 (21-49)[e]<br><br><u>Gender (female):</u><br>50%	Malaria treatment and prophylaxis	To assess factors and features associated with CQ induced pruritus	<u>Inclusion criteria:</u><br>- South Western Nigerian Black volunteers<br>- ≥ 15 years	(a) Cross-sectional study<br>(b) NA<br>(c) NA<br>(d) July 2002 to March 2004<br>(e) Nigeria	(a) <u>Pruritus:</u><br>100% of the patients treated with oral CQ	(a) NA<br>(b) NA<br>(c) NA	67
Factors associated with chloroquine-induced pruritus during malaria treatment in Mozambican university students.	CQ	795 (survey respondents)<br><br>542 (treated with CQ)	<u>Age (years):</u><br>23 (18-51)[d]<br><br><u>Gender (female):</u><br>58.7%	Malaria treatment and prophylaxis	To assess the prevalence and the risk factors of CQ induced pruritus	<u>Inclusion criteria:</u><br>- Students enrolled at a private University in Maputo, Mozambique who agreed to sign the informed consent	(a) Cross-sectional study<br>(b) NA<br>(c) NA<br>(d) March 2004<br>(e) Mozambique	(a) <u>Pruritus:</u><br>1) 158 out of 525 (30.1%) of the whole subjects presented pruritus<br>2) Pruritus was more frequent in those patients treated for malaria than prophylactic treatments (31.2 vs. 10.3% $p<0.05$)	(a) NA<br>(b) NA<br>(c) NA	76

Gama H. Gac Sanit. 2009										
Prescription pattern of anti-malarial drugs in a tertiary care hospital. Jeevangi SR. Asian Pac J Trop Med. 2010	CQ	212 prescriptions<br><br><u>Groups:</u><br>*P. vivax* cases (treated with CQ): 128<br>*P. falciparum* cases (treated with other drugs): 84	<u>Age (years):</u><br>18-30: 96 (45.23)[c]<br>31-49: 64 (30.18)[c]<br>50-69: 44 (20.75)[c]<br><br><u>Gender (male):</u><br>136 (64.15)[c]	Malaria	To evaluate the prescribing pattern of antimalarial drugs in a tertiary care hospital	<u>Inclusion criteria:</u><br>- Prescriptions from the newly registered patients	(a) Cross-sectional<br>(b) Prospective<br>(c) NA<br>(d) January 2002 to July 2002<br>(e) India	(a) <u>Anorexia:</u><br>9.37%<br><u>Nausea:</u><br>9.37%<br><u>Vomiting:</u><br>9.37%<br><u>Tinnitus:</u><br>9.37%	(a) NA<br>(b) NA<br>(c) NA	33
Prevalence of ototoxicity in University of Benin Teaching Hospital, Benin city: A 5-year review. Obasikene G. Niger J Clin Pract. 2012	CQ	79	<u>Age (years):</u><br>31-40 (1.89)[b]<br><br><u>Gender (male):</u><br>40 (50.6)[c]	Malaria	To identify implicated drugs in patients diagnosed with ototoxicity	<u>Inclusion criteria:</u><br>- Sudden or progressive hearing loss of less than 3 w duration, patients who did pure tone audiometry following the hearing loss<br><u>Exclusion criteria:</u><br>- Patients with previous history of hearing impairment from any other cause<br>- Incomplete data	(a) Cohort study<br>(b) Retrospective<br>(c) Within the 3 w since the patient noticed audition loss<br>(d) June 2005 to July 2010<br>(e) Benin	(a) <u>Ototoxicity</u>:<br>CQ was the responsible for 6.3% of ototoxicity cases and 84.8% of the patients developed tinnitus	(a) NA (less than 3 w)<br>(b) NA<br>(c) NA	95
Prevalence and risk factors associated to pruritus in Plasmodium vivax patients using chloroquine in the Brazilian Amazon. Ballut PC. Acta Trop. 2013	CQ	510 (patients interviewed)	<u>Age (years):</u><br>< 15: 8.8%<br>16 to 50: 68.5%<br>>50: 22.7%<br><br><u>Gender (male):</u><br>59.7%	Malaria	To estimate the frequency of pruritus and associated risk factors in *P.vivax*-infected patients treated with CQ in a reference centre in the Brazilian Amazon	<u>Inclusion criteria:</u><br>- Patients who were prescribed CQ for the treatment of microscopy-confirmed *P. vivax* infection in the past five days<br><u>Exclusion criteria:</u><br>- Patients referring pruritus prior to CQ initiation	(a) Cross-sectional study<br>(b) NA<br>(c) NA<br>(d) 2008<br>(e) Brazilian Amazon	(a) <u>Pruritus:</u><br>104 (20.4)[c] (CI 95%: 16.9-23.9%)<br><u>Pruritus impaired sleep:</u><br>51 (49)[c]	(a) NA<br>(b) NA<br>(c) 25 mg/kg over 3 days	87
Antimalarial chemoprophylaxis and the risk of neuropsychiatric disorders. Schneider C. Travel Med Infect Dis. 2013	CQ	<u>Groups:</u><br>Cases: 414<br>Controls: 537	<u>Age (years):</u><br>CQ and/or proguanil group:<br><40: 26 (55.31)[c]<br>40-49: 9 (19.15)[c]<br>50-59: 5 (10.64)[c]	Malaria prophylaxis	To assess the risk of developing first-time anxiety, stress related disorders or psychosis, depression, epilepsy or peripheral	<u>Inclusion criteria:</u><br>- Cases: people with an incident diagnosis of a neuropsychiatric disorder during or after anti-malarial drug use.<br>- Controls: selected randomly up to six controls per case for the nested analysis; patients who did not develop an	(a) Case-control study<br>(b) Retrospective<br>(c) From first prescription or first pre-travel consultation to 540 days after drug exposition	(a) <u>Anxiety or stress related disorders combined:</u><br>47 cases took CQ and/or proguanil. OR: 1.04 (CI 95%: 0.74-1.46)<br><u>Depression:</u><br>33 cases took CQ and/or proguanil.<br>OR: 1.06 (CI 95%: 0.71-1.59)<br><u>Epilepsy:</u>	(a) NA<br>(b) NA<br>(c) NA	89

			60-69: 2 (4.25)[c]<br>≥70: 5 (10.64)[c]<br>Controls:<br><40: 306 (56.98)[c]<br>40-49: 104 (19.37)[c]<br>50-59: 71 (13.22)[c]<br>60-69: 45 (8.38)[c]<br>≥70: 11 (2.05)[c]<br><br>Gender (female):<br>CQ and/or proguanil group: 37 (78.72)[c]<br>Controls: 350 (65.18)[c]		neuropathies on patients using antimalarials as compared to unexposed travellers	outcome of interest during follow-up<br><br>Exclusion criteria:<br>- Cases: diagnosis of malaria prior to the start of anti-malarial drug use, patients with a history of cancer, alcoholism, rheumatoid arthritis or with an outcome of interest prior to using anti-malarial drugs	(d) January 2001 to October 2009<br>(e) United Kingdom	7 cases took CQ and/or proguanil. OR: 1.41 (CI 95%: 0.54-3.67)<br>Peripheral neuropathies:<br>3 cases took CQ and/or proguanil. OR: 1.59 (CI 95%: 0.41-6.15)		
Gender and environmental influences on visual acuity in Owerri, Nigeria. Emerole CG. Niger J Physiol Sci. 2014	CQ	3451<br><br>Groups:<br>Tests: 2606<br>Controls: 845	(a) Age (years):<br>The majority of subjects aged 40-49<br><br>(b) Gender (male):<br>Tests: 1079 (41.40)[c]<br>Control: 357 (42.25)[c]	Malaria	To assess the gender and environmental influences on visual function among adults in Owerri, Nigeria	Inclusion criteria:<br>- Cases: eye defects<br>- Controls: individuals who did not present any eye defects on examination protocols<br>Exclusion criteria:<br>- Conditions interfering with accurate ocular refraction such as corneal opacity, visually impairing opaque media, diabetes mellitus, hypertension, pseudophakics and aphakics	(a) Cross-sectional<br>(b) Retrospective<br>(c) NA<br>(d) September 2007 to November 2009<br>(e) Nigeria	(a) Visual acuity:<br>CQ use as first line of malaria treatment was reported as contributory factor for loss of visual acuity	(a) NA<br>(b) NA<br>(c) NA	60
Psychosis following chloroquine ingestion: A 10-year comparative study from a malaria-hyperendemic district of India. Sarathi P. Gen Hosp Psychiatry. 2014	CQ base	102<br><br>Groups:<br>Cases of psychosis following CQ: 51<br>Controls: brief psychotic disorder not	Age:<br>31.29 (10.58)[b] PFC<br>25.76 (4.91)[b] BPD<br><br>Gender (male):<br>27 (52.9)[c] PFC	Malaria	To identify the most frequent type of psychiatric manifestation and symptomatic characteristics of psychosis	Inclusion criteria:<br>Cases:<br>- Diagnosed by consultant psychiatrists<br>- No psychiatric history before starting CQ<br>- Prominent hallucinations or delusions not exclusively during delirium	(a) Case-control study<br>(b) Retrospective<br>(c) 6 months<br>(d) 2002 to 2012<br>(e) India	(a) Brief Psychiatric Rating Scale:<br>Somatic concern:<br>1.81 (1.36)[b] CQ; 1.14 (0.48)[b] no CQ<br>Anxiety:<br>2.33 (1.49)[b] CQ; 2.52 (1.60)[b] no CQ<br>Emotional withdrawal:<br>1.95 (1.36)[b] CQ; 3.43 (1.83)[b] no CQ	(a) 100.08 (96)[b] h<br>(b) 1.4 (0.62)[b] g<br>(c) NA	85

		related with CQ:<br>51	24 (47.1)[c]<br>BPD		following CQ ingestion	- Psychiatric symptoms continuing for at least 1 w<br>- Onset of psychotic disorder within 6 w after starting CQ<br>- To take at least one tablet of CQ (150 mg base)<br>- Symptoms have to be severe enough to cause hospitalization or referral for specialist treatment<br>- Symptoms must be resolved within 8 w of cessation of CQ<br>- Disturbance is not better accounted for by a psychotic disorder that was not substance induced<br>Controls:<br>- Diagnosed with brief psychotic disorder according to DMS-IV-TR<br><u>Exclusion criteria:</u><br>- Family history of any psychotic disorder in first- and second-degree relatives<br>- Prior history of significant head injury<br>- Major medical or neurological disorders<br>- Concomitant prescribed or illicit drug use		<u>Conceptual disorganization:</u><br>3.24 (1.26)[b] CQ; 3.86 (1.42)[b] no CQ<br><u>Guilt feelings:</u><br>1.24 (0.54)[b] CQ; 1.33 (0.79)[b] no CQ<br><u>Tension:</u><br>3.19 (1.29)[b] CQ; 3.67 (1.53)[b] no CQ<br><u>Mannerisms and posturing:</u><br>1.14 (0.65)[b] CQ; 2.14 (1.90)[b] no CQ<br><u>Grandiosity:</u><br>1.71 (1.49)[b] CQ; 1.95 (1.63)[b] no CQ<br><u>Depressive mood:</u><br>1.67 (1.28)[b] CQ; 2.19 (1.69)[b] no CQ<br><u>Hostility:</u><br>3.38 (1.71)[b] CQ; 2.52 (1.57)[b] no CQ<br><u>Suspiciousness:</u><br>2.81 (1.63)[b] CQ; 3.38 (1.50)[b] no CQ<br><u>Hallucinatory behaviour:</u><br>2.86 (1.71)[b] CQ; 2.43 (2.09)[b] no CQ<br><u>Motor retardation:</u><br>1.33 (0.86)[b] CQ; 2.05 (1.69)[b] no CQ<br><u>Uncooperativeness:</u><br>3.67 (0.97)[b] CQ; 3.48 (1.36)[b] no CQ<br><u>Unusual thought content:</u><br>3.48 (1.67)[b] CQ; 2.67 (1.06)[b] no CQ<br><u>Blunted affect:</u><br>1.52 (0.98)[b] CQ; 2.81 (1.75)[b] no CQ<br><u>Excitement:</u><br>3.71 (1.42)[b] CQ; 3.24 (1.79)[b] no CQ<br><u>Disorientation:</u><br>1.95 (0.74)[b] CQ; 1 (0.0)[b] no CQ<br><br>Young Mania Rating Scale:<br><u>Elevated mood:</u>		

								0.34 (1.12)[b] CQ; 0.33 (0.80)[b] no CQ<br>Increased motor activity:<br>2.05 (1.60)[b] CQ; 1.38 (1.53)[b] no CQ<br>Sexual interest:<br>0.52 (1.12)[b] CQ; 0.57 (0.98)[b] no CQ<br>Sleep:<br>2.48 (0.68)[b] CQ; 1.81 (0.98)[b] no CQ<br>Irritability:<br>2.95 (1.63)[b] CQ; 2.71 (1.23)[b] no CQ<br>Rate and amount of speech:<br>1.52 (1.72)[b] CQ; 0.48 (0.75)[b] no CQ<br>Language-thought disorder:<br>1.90 (1.61)[b] CQ; 1.19 (1.44)[b] no CQ<br>Content:<br>1.24 (2.30)[b] CQ; 1.19 (2.46)[b] no CQ<br>Disruptive-aggressive behaviour:<br>2.28 (2.55)[b] CQ; 1.81 (1.44)[b] no CQ<br>Appearance:<br>1.33 (1.02)[b] CQ; 1.71 (1.06)[b] no CQ<br>Insight:<br>3.86 (0.65)[b] CQ; 3.90 (0.44)[b] no CQ<br><br>Hamilton Depression Rating Scale:<br>Depressed mood:<br>0.52 (1.12)[b] CQ; 1.90 (1.92)[b] no CQ<br>Feelings of guilt:<br>0.33 (0.86)[b] CQ; 0.33 (0.73)[b] no CQ<br>Suicide:<br>0.48 (0.93)[b] CQ; 0.09 (0.3)[b] no CQ<br>Insomnia: early in the night:<br>1.95 (0.22)[b] CQ; 1.71 (0.72)[b] no CQ<br>Insomnia, middle of the night:		

								1.24 (0.89)^b CQ; 1.19 (0.93)^b no CQ<br>Insomnia, early hours of the morning:<br>1.24 (0.89)^b CQ; 1.33 (0.97)^b no CQ<br>Work and activities:<br>3.24 (0.94)^b CQ; 3.86 (0.36)^b no CQ<br>Retardation:<br>0.33 (0.86)^b CQ; 1.62 (1.75)^b no CQ<br>Agitation:<br>2.38 (0.92)^b CQ; 1.95 (1.02)^b no CQ<br>Anxiety psychic:<br>2.43 (1.54)^b CQ; 2.19 (1.47)^b no CQ<br>Anxiety somatic:<br>1.52 (1.47)^b CQ; 1 (1.38)^b no CQ<br>Somatic symptoms gastrointestinal:<br>0.66 (0.91)^b CQ; 1.48 (1.03)^b no CQ<br>General somatic symptoms:<br>0.57 (0.93)^b CQ; 0.57 (0.68)^b no CQ<br>Genital symptoms:<br>0.62 (1.02)^b CQ; 1.62 (0.8)^b no CQ<br>Hypochondriasis:<br>0.43 (0.93)^b CQ; 0.24 (0.54)^b no CQ<br>Loss of weight:<br>1.33 (0.91)^b CQ; 1.44 (0.85)^b no CQ<br>Insight:<br>1.90 (0.44)^b CQ; 2 (0.0)^b no CQ		
Assessment of the efficacy and safety of chloroquine monotherapy for the treatment of acute uncomplicated gestational malaria caused by *P. vivax*, Cordoba, Colombia, 2015-2017. Castro-	CQ	47	Age:<br>22 (6.77)^b<br>20.5 (14-42)^f<br><br>Gender (female):<br>100%	Malaria	To determine the efficacy of CQ monotherapy in Colombian pregnant women with acute uncomplicated malaria vivax	Inclusion criteria:<br>- Pregnant women who presented of their own to malaria or prenatal care in whom the diagnosis of *P. vivax* was confirmed by means of blood spot test and and qPCR<br>Exclusion criteria:	(a) Cohort study<br>(b) Prospective<br>(c) Follow-up period of 28 days for therapeutic failure and serious AE assessment and a follow-up	(a) No serious AE were identified. The most non-serious AE were confused in frequency and intensity with symptoms and signs of malaria	(a) NA<br>(b) NA<br>(c) Initial dose of 10 mg/kg and 7.5 mg/kg at 24 and 48 h	90

Cavadía CJ. Rev Colomb Obstet Ginecol. 2020						- Patients with toxoplasmosis, rubella, cytomegalovirus, herpes simplex, HIV, syphilis, other blood disease than anemia and liver or kidney disease	period of 120 days for frequency of recurrence-relapse determination (d) February 2015 to December 2017 (e) Colombia			

Abbreviations: ADRs: adverse drug reactions; AE: adverse events; BPD: brief psychotic disorder not related with CQ; CQ: chloroquine; DMS-IV-TR: Diagnostic and Statistical Manual of Mental Disorders, Fourth Edition, Text Revision; g: grams; h: hours; HCQ: hydroxychloroquine; mg: milligrams; NA: not available/not applicable; OR: odds ratio; PFC: psychosis following CQ; qPCR: quantitative polymerase chain reaction; w: weeks.

[a]Quality assessment: a higher score indicates a higher quality: [Yes (1) + Partly (0.5)/Total applicable] x100.
Values are: [b]mean (SD); [c] n (%); [d]mean (range); [e]median (interquartile range); [f]median (range).

Table S14. Characteristics of included studies: observational studies, patients affected by cutaneous and/or systemic lupus erythematosus and dermatomyositis.

Study ID: title, first author, journal of publication, year	Drug	Sample Size and study groups	Age and Gender	Drug Indication	Aim	Patient Inclusion and Exclusion Criteria	Main study characteristics: (a) Epidemiological approach (b) Data gathering (c) Time horizon (d) Time frame (e) Location	Adverse event characteristics: (a) Measure	Other: (a) Time of appearance (b) Cumulative dose (c) Regimen	Quality assessment[a] (%)
Changing antimalarial agents after inefficacy or intolerance in patients with cutaneous lupus erythematosus: A multicenter observational study. Chasset F. J Am Acad Dermatol. 2018	CQ and HCQ	64	Age (years): AE group at the time of switch: 38 (13-74)[f]<br><br>Gender (female): AE group: 13 (81)[c]	CLE	To evaluate the effect on cutaneous response of a switch from HCQ to CQ, or the reverse, after failure of the first antimalarial agent	Inclusion criteria:<br>- Acute, subacute, chronic, or intermittent CLE (tumid lupus erythematosus) with pathological confirmation based on validated clinical and histopathological criteria<br>- Patients who received HCQ or CQ after failure of the first antimalarial agent for inefficacy or intolerance<br>- Known cutaneous response at least 3 months after the switch<br>- The patients had no change in other therapies that could affect CLE activity for at least 3 months after the switch<br>Exclusion criteria:<br>- The dose of HCQ or CQ was unknown or if another treatment that could affect CLE activity, including topical treatment, was prescribed with the switch	(a) Cohort study<br>(b) Retrospective<br>(c) The overall cutaneous response rate and reasons for failure of the switch were assessed for up to 48 months<br>(d) 1997 to September 2015<br>(e) France	(a) Macular and papular exanthema (without severity criteria):<br>A total of 16 patients had AE responsible for failure of antimalarial treatment. Among them, in only 2 cases was specified that macular and papular exanthema appeared 10 and 15 days after treatment initiation, respectively	(a) 10 and 15 days<br>(b) NA<br>(c) NA	92
Therapeutic efficacy and adverse events of hydroxychloroquine administration in Japanese systemic/cutaneous lupus erythematosus patients. Kishi C. J Dermatol. 2018	HCQ	31	Age (years, range):<br>14-73<br><br>Gender (female):<br>26 (83.87)[c]	SLE and CLE	To assess the clinical efficacy and AE of HCQ	Inclusion criteria:<br>- SLE and CLE patients treated with HCQ	(a) Cohort study<br>(b) Retrospective<br>(c) NA<br>(d) 2015 to 2017<br>(e) Japan	(a) General AE:<br>3 (9.7)[c] patients<br>Erythema:<br>1 (3.22)[c]<br>Urticaria:<br>1 (3.22)[c]<br>Maculopapular erythema:<br>1 (3.22)[c]<br>Abdominal pain:<br>1 (3.22)[c]	(a) Described cases appeared from 1 to 2 w after initiation<br>(b) NA<br>(c) NA	64

								<u>Diarrhea:</u><br>2 (6.45)[c]<br><u>Colour vision abnormality:</u><br>1 (3.22)[c]<br><u>Dizziness:</u><br>1 (3.22)[c]<br><u>Eosinophilia:</u><br>1 (3.22)[c]		
Adverse cutaneous drug reactions with antimalarials in cutaneous lupus and dermatomyositis: A retrospective cohort study. Gonzalez CD. J Am Acad Dermatol. 2019	CQ and HCQ	180	<u>Age (years, mean):</u><br>Cutaneous lupus erythematosus: 48<br>Dermatomyositis: 54<br><br><u>Gender (female):</u><br>Cutaneous lupus erythematosus: 80%<br>Dermatomyositis: 84%	Cutaneous lupus erythematosus and dermatomyositis	To assess the frequency of antimalarial adverse cutaneous drug eruptions of adults patients	<u>Inclusion criteria:</u><br>- Patients who were taking HCQ or CQ and had at least 3 months of follow-up data noting reaction timing and severity	(a) Cohort study<br>(b) Retrospective<br>(c) NA<br>(d) January 2013 to January 2018<br>(e) Utah	(a) <u>Cutaneous drug eruptions:</u><br>Cutaneous lupus erythematosus patients:<br>4 (4)[c]<br>Dermatomyositis patients:<br>4 (5)[c]	(a) Adverse cutaneous drug eruptions occurred 5 to 14 days after initiation of HCQ treatment<br>(b) NA<br>(c) NA	56

Abbreviations: AE: adverse events; CLE: cutaneous lupus erythematosus; CQ: chloroquine; HCQ: hydroxychloroquine; NA: not available/not applicable; SLE: systemic lupus erythematosus.

[a] Quality assessment: a higher score indicates a higher quality: [Yes (1) + Partly (0.5)/Total applicable] x100.
Values are: [b]mean (SD); [c] n (%); [d]mean (range); [e]median (interquartile range); [f]median (range).

Table S15. Characteristics of included studies: observational studies, patients affected by porphyria cutanea tarda.

Study ID: title, first author, journal of publication, year	Drug	Sample Size and study groups	Age and Gender	Drug Indication	Aim	Patient Inclusion and Exclusion Criteria	Main study characteristics: (a) Epidemiological approach (b) Data gathering (c) Time horizon (d) Time frame (e) Location	Adverse event characteristics: (a) Measure	Other: (a) Time of appearance (b) Cumulative dose (c) Regimen	Quality assessment[a] (%)
High dose hydroxychloroquine treatment of porphyria cutanea tarda. Petersen CS. J Am Acad Dermatol. 1992	HCQ	72<br><br>Groups:<br>High-dose HCQ: 65<br>Modified high-dose HCQ: 7	Age (years):<br>Male:<br>50.9 (25-75)[d]<br>Female:<br>46.8 (23-70)[d]<br>Gender (male):<br>42 (64.6)[c]	PCT	Efficacy and safety of a high-dose HCQ regimen	Inclusion criteria:<br>- First episode of PCT	(a) Cohort study<br>(b) Retrospective<br>(c) Male: 37.3 months; female: 25.9 months<br>(d) 1980 to 1990<br>(e) Denmark	(a) - High-dose HCQ group:<br>AST values >40U per liter:<br>62 (95.4)[c] of which 17 (26.2)[c] had AST values >1000 U per liter<br>Fever:<br>27 (41.5)[c]<br>Headache:<br>18 (27.7)[c]<br>Myalgia:<br>19 (29.2)[c]<br>Nausea:<br>14 (21.5)[c]<br>Abdominal pain:<br>13 (20.0)[c]<br>Vomiting:<br>9 (13.8)[c]<br>Arthralgia:<br>4 (6.2)[c]<br>Fatigue:<br>10 (15.4)[c]<br>Hepatomegaly:<br>2 (3.1)[c]<br>Icterus:<br>1 (1.5)[c]<br>- Modified high-dose HCQ group:<br>Only 2 patients experienced AE: one patient had abdominal pain and nausea and one patient had abdominal pain	(a) NA<br>(b) 2.25 g (65 patients), 1.5 g (2 patients), 11.25 g (1 patient), 5.25 g (1 patient), 3.5 g (1 patient), 1 g (1 patient), 0.75 g (1 patient)<br>(c) NA	60
Porphyria cutanea tarda: effects and risk factors for hepatotoxicity from high-dose chloroquine	CQ phosphate	57	Age (years):<br>Median age for clinical manifestation of PCT was 61 (34-81)[f] for	PCT	To find possible predictors of hepatotoxicity and side-effects associated with high-dose CQ	Inclusion criteria:<br>- Patients with a first-time diagnosis of PCT	(a) Cohort study<br>(b) Retrospective<br>(c) During the period 1978 to	(a) Hepatotoxic reaction:<br>A hepatotoxic reaction with high serum aminotransferases occurred in almost all patients<br>Influenza-like symptoms (fever and/or nausea):	(a) NA<br>(b) CQ phosphate, 250 mg daily, was given orally for 7 days. Treatment was stopped	75

treatment. Rossmann-Ringdahl I. Acta Derm Venereol. 2007			females and 59 (29-77)[f] for males<br><br>Gender (male):<br>36 (63.16)[c]		treatment in patients with PCT		1997 and followed-up until 2005<br>(d) NA<br>(e) Sweden	25 (43.85)[C]	prematurely after 2-4 days in 4 patients because of influenza-like symptoms with very high serum amino transferases<br>(c) NA	

Abbreviations: AE: adverse events; CQ: chloroquine; HCQ: hydroxychloroquine; NA: not available/not applicable; PCT: porphyria cutanea tarda.

[a] Quality assessment, a higher score indicates a higher quality: [Yes (1) + Partly (0.5)/Total applicable] x100.
Values are: [b]mean (SD); [c] n (%); [d]mean (range); [e]median (interquartile range); [f]median (range).

Table S16. Characteristics of included studies: observational studies, patients affected by diverse pathologies.

Study ID: title, first author, journal of publication, year	Drug	Sample Size and study groups	Age and Gender	Drug Indication	Aim	Patient Inclusion and Exclusion Criteria	Main study characteristics: (a) Epidemiological approach (b) Data gathering (c) Time horizon (d) Time frame (e) Location	Adverse event characteristics: (a) Measure	Other: (a) Time of appearance (b) Cumulative dose (c) Regimen	Quality assessment[a] (%)
Detection and incidence of muscular adverse drug reactions: a prospective analysis from laboratory signals. Dugué A. Eur J Clin Pharmacol. 2004	HCQ	103	Patients affected with HCQ muscular ADRs:<br>-One 53 years old male<br>-One 47 years old female	NA	To assess the detection and incidence of muscular ADRs in a University Hospital using biochemical laboratory data. To assess the underreporting rate of drug-induced muscular disorders	Inclusion criteria:<br>- Increase of CPK levels ≥ 2 the upper limit of normal range<br>- Cardiology and neurology outpatients<br>Exclusion criteria:<br>- Patients hospitalized in emergency, re-animation, cardiology, day-care services neurology and traumatology departments	(a) Cohort study<br>(b) Prospective<br>(c) NA<br>(d) 1 w per month from November 2001 to October 2002<br>(e) France	(a) Muscular AE:<br>HCQ was involved in 2 (7.1)[c] of muscular ADRs cases (one of them the suspected drugs were HCQ, methotrexate and simvastatin)	(a) NA<br>(b) NA<br>(c) NA	80
Evaluation of the prevalence and economic burden of adverse drug reactions presenting to the medical emergency department of a tertiary referral centre: a prospective study. Patel KJ. BMC Clin Pharmacol. 2007	CQ	6899	Age (years):<br>40 (mean)<br>Gender:<br>NA	NA	To determine the prevalence of patients presenting ADRs to the ED and the causality, avoidability, severity and economic burden of these ADRs	Inclusion criteria:<br>- ≥ 18 years<br>- Patients presenting with ADRs to the ED	(a) Cohort study<br>(b) Prospective<br>(c) ED and hospitalization<br>(d) 1st May 2005 to 15th June 2005<br>(e) India	(a) Gastritis:<br>22 out of 265 ADRs were gastritis secondary to CQ	(a) NA<br>(b) NA<br>(c) NA	64
Risk factors for acute generalized exanthematous pustulosis (AGEP)-results of a multinational case-control study	CQ and HCQ	97 cases and 1009 controls<br><br>7 cases and 2 controls were exposed to CQ and HCQ	Age:<br>Cases: 56 (21)[b]<br>Controls: 48 (24)[b]	NA	To assess the risk for different drugs of causing severe cutaneous adverse reactions	Inclusion criteria:<br>- Cases: patients classified as definite and probable after reviewed by a multinational expert committee of dermatologists blinded for information on risk factors	(a) Case-control study<br>(b) Retrospective<br>(c) NA<br>(d) April 1997 to December 2001	(a) HCQ and CQ group:<br>AGEP: OR 39 (CI 95%: 8-∞)	(a) NA<br>(b) NA<br>(c) NA	77

(EuroSCAR). Sidoroff A. Br J Dermatol. 2007			Gender (male/female ratio):<br>Cases: 0:80<br>Controls: 0:73			- Controls: three control patients were obtained for each AGEP case. Controls were patients admitted to the same hospital also for an acute disease but not suspected of resulting from drug use<br>Exclusion criteria:<br>- Cases: patients excluded or classified as possible cases after reviewed by a multinational expert committee of dermatologists	(e) Austria, France, Israel, Italy, Netherlands and Germany			
The comparative safety of multiple alternative agents in refractory chronic urticaria patients. Seth S. J Allergy Clin Immunol Pract. 2017	HCQ	126<br><br>Groups (25 patients were treated with more than 1 alternative agent):<br>Dapsone: 73<br>Sulfasalazine: 47<br>HCQ: 45<br>Tacrolimus: 36<br>Mycophenolate: 27<br>Omalizumab: 24<br>Cyclosporine: 8	Age:<br>44 (18-69)[d]<br><br>Gender (female):<br>77%	Refractory chronic urticaria	To assess the safety of alternative agents for chronic urticaria	Inclusion criteria:<br>- Diagnosis of chronic urticaria<br>- Treatment with alternative agent<br>- ≥ 18 years<br>Exclusion criteria:<br>- Lack of information<br>- Other indications for treatment	(a) Cohort study<br>(b) Retrospective<br>(c) Treatment duration<br>(d) January 2001 to April 2014<br>(e) Dallas, Texas	(a) HCQ group:<br>Pruritus:<br>One patient suffered pruritus on day 1 and small itchy bumps	(a) One patient suffered pruritus on day 1 and small itchy bumps on day 4. Appearance time was NA in other 6 cases<br>(b) NA<br>(c) NA	75

Abbreviations: ADRs: adverse drug reactions; AE: adverse events; AGEP: acute generalized exanthematous pustulosis; CQ: chloroquine; ED: emergency department; HCQ: hydroxychloroquine; NA: not available/not applicable.

[a] Quality assessment, a higher score indicates a higher quality: [Yes (1) + Partly (0.5)/Total applicable] x100.
Values are: [b]mean (SD); [c]n (%); [d]mean (range); [e]median (interquartile range); [f]median (range).

Table S17. Characteristics of included studies: clinical trials, patients affected by malaria or who received prophylactic treatment.

Study ID: title, first author, journal of publication, year	Drug (CQ/ HCQ)	Sample Size	Aim	Age (years) and Gender	Patient Inclusion and Exclusion Criteria	Main study characteristics: (a) Type of study (b) Time Horizon (c) Time Frame	Regimens	Adverse event characteristics: (a) Measure	Jadad quality score[a]
Prolonged malaria prophylaxis with chloroquine and proguanil (chloroguanide) in a nonimmune resident population of an endemic area with a high prevalence of chloroquine resistance. Gozal D. Antimicrob Agent Chemother. 1991	CQ base	156	To study the efficacy and safety of weekly CQ and daily proguanil (chloroguanide) (CQ-P) in malaria prevention	CQ-P treated group:<br><br>Age: 23.7[b]<br><br>Gender (male): 52.5%	Inclusion criteria:<br>- Non-immune residents of Israeli nationality recruited in the week preceding the beginning of the study and living in the Yaounde area for the duration of the study<br>Exclusion criteria: NA	(a) NA<br>(b) 18-month prospective study<br>(c) August 1988 to January 1990	- 78 CQ-P-treated subjects: every 2 w were given prophylactic antimalarial medication consisting of weekly CQ base (5 mg/kg) and daily proguanil (3.5 mg/kg)<br><br>- 52 subjects received no prophylaxis	(a) Side effects were reported in a total of 26 CQ-P-treated subjects (31%) as follows: anorexia in 13 subjects, nausea in 9 subjects, emesis in 7 subjects, abdominal pain or discomfort in 19 subjects, diarrhoea in 3 subjects, recurrent oral ulcers usually in the soft palate or in the tip of the tongue in 15 subjects, headache in 8 subjects, pruritus in 1 subject, and visual disturbances consisting of difficulty in reading and temporary foggy vision without discernible retinal changes in 1 subject	0
Norfloxacin is inferior to chloroquine for falciparum malaria in Northwestern Zambia: a comparative clinical trial. McClean K. The Journal of Infectious Diseases. 1992	CQ phos phat e	41	To compare norfloxacin with a standard course of CQ	Age: 30[b]<br><br>Gender (male): 22.2%	Inclusion criteria:<br>- Semi-immune adults of both sexes > 18 years and a blood film demonstrating asexual forms of *P. falciparum*<br>- Female patients of childbearing potential were required to have a negative pregnancy test before enrollment and to practice effective birth control measures during the study<br>Exclusion criteria:<br>- Allergy to CQ, norfloxacin, nalidixic acid, or other quinolone derivatives<br>- Pregnancy or lactation<br>- Need for parenteral therapy<br>- Severe complications of malaria and treatment of malaria within the previous	(a) Randomized<br>(b) Twenty eight days<br>(c) May 1990 to February 1991	- 20 patients randomized to treatment with CQ received 1 g orally followed by 0.5 g in 6 h and 0.5 g daily for 2 days<br><br>- 21 patients given norfloxacin received 0.4 g orally every 12 h for 3 days	(a) Pruritus:<br>Patients in the CQ group were symptomatic for an average of 2.9 days after the initiation of therapy. AEs were significantly more frequent in the CQ group, in which 8 of 18 patients developed pruritus ($p < 0.001$)<br>(b) No patient in either group had an AE considered serious enough to warrant any change in therapy	1

					30 days or the ingestion of any antimalarial agent within the 7 days before entry into the study<br>- Patients requiring concurrent treatment with antibiotics known to have some activity against *P.falciparum* (tetracyclines, erythromycin, clindamycin, sulfonamides, dapsone, and trimethoprim)<br>- Patients with combined infections with *P. falciparum* and Borrelia duttoni at the time of enrollment				
Randomized dose-ranging study of the safety and efficacy of WR 238605 (Tafenoquine) in the prevention of relapse of *Plasmodium vivax* malaria in Thailand. Walsh DS. J Infect Dis. 1999	CQ	44	To assess safety, tolerability, and the prevention of *P. vivax* malaria relapse of WR 238605 (tafenoquine)	Group D (CQ only):<br>Age:<br>23 (19–28)[f]<br><br>Gender (ratio male:female):<br>2:7	Inclusion criteria:<br>- Thai or ethnic Burmese patients aged 18–60 years with *P. vivax* infections admitted to hospital wards<br>- Weight within 20% of the standards for the population<br>- Normal G6PDH screen<br>- Ability to take oral medication<br>- Negative serum pregnancy test<br>Exclusion criteria:<br>Mixed infections (*P. vivax and P. falciparum*)<br>- Severe anemia, protracted vomiting, oliguria, hypotension, severe central nervous system symptoms, hematologic or biochemical abnormalities beyond values expected in the setting of malaria infection<br>- Lactation<br>- Concomitant systemic disease<br>- Ingestion of any antimalarial drug in the past 14 days or evidence	(a) Randomized, open label<br>(b) Follow up > 2 months<br>(c) August 1996 to June 1997	Patients were randomized:<br><br>Three groups received a dose of CQ followed by WR 238605:<br>- Group A (n = 15) received 300 mg daily for 7 days<br>- Group B (n = 11) received 500 mg daily for 3 days and repeated 1 w after the initial dose<br>- Group C (n = 9) received 1 dose of 500 mg<br><br>- Group D (n = 9) received CQ only	(a) AEs occurred in a minority of patients in all treatment groups. Abdominal discomfort and diarrhoea: only gastrointestinal disturbances were slightly more frequent in the WR 238605–treated groups than among patients treated with CQ only. There were no serious adverse events	1

					suggestive of drug or alcohol abuse				
Rapid therapeutic response onset of a new pharmaceutical form of chloroquine phosphate 300 mg: Effervescent tablets. Yanze MF. Trop Med Int Health. 2001	CQ	60	To asses efficiency, safety and taste of two pharmaceutical forms of CQ phosphate 300 mg CQTE and CQTU in the treatment of acute uncomplicated malaria due to *P. falciparum*	Age:<br>29 (7) [19-51][g] CQTE<br>32 (6) [22-49][g] CQTU<br><br>Gender (male):<br>17[c] CQTE<br>19[c] CQTU	Inclusion criteria:<br>- Live in the area of their respective Health centre<br>- Verbal consent<br>- Weight > 45 kg<br>- Axillary temperature 37.2 ºC – 4 ºC<br>- Parasite density > 3000 asexual parasites/µl<br>- Negative Wilson-Edeson and Modified Bratton-Marshall test	(a) Randomized, open label<br>(b) 7 days<br>(c) July to September 1999	Patients were assigned to one of two treatments (CQTE or CQTU) according to the following schedule D0: 10 mg/kg 600 mg 2 tablets; D1: 10 mg/kg 600 mg 2 tablets; D3: 5 mg/ kg 300 mg 1 tablet<br><br>- CQTE group: 30 patients<br><br>- CQTU group: 30 patients	(a) Headache:<br>0% CQTE; 6.7% CQTU<br>Diarrhoea:<br>3.3% CQTE; 10% CQTU<br>Abdominal pain:<br>6.7% CQTE; 16.7% CQTU<br>Nausea:<br>6.7% CQTE; 13.3% CQTU<br>Pruritus:<br>3.3% CQTE; 6.7% CQTU<br>Dizziness:<br>3.3% CQTE; 6.7% CQTU<br>Vomiting:<br>3.3% CQTE; 3.3% CQTU	2
A double-blind, randomized study of azithromycin compared to chloroquine for the treatment of *Plasmodium vivax* malaria in India. Dunne MW. Am J Trop Med. Hyg. 2005	CQ	199	To explore the role of a 3-day course of AZM for treatment of vivax malaria	Age:<br>CQ: 30.0 (11.8)[b]<br>AZM: 31.7 (11.6)[b]<br><br>Gender (male):<br>CQ : 77 %<br>AZM: 82 %	Inclusion criteria:<br>- 18 to 65 years, history of fever within the prior 48 h, a Giemsa-stained thin smear of peripheral blood with asexual forms consistent with *P. vivax* and a quantitative count of < 100,000 parasites/?L as well as a rapid test negative for evidence of *P. falciparum*<br><br>Exclusion criteria:<br>- Evidence of impaired consciousness, jaundice, respiratory distress, self-report of hematuria, treatment with any antimalarial drug or antibacterial with known antimalarial activity within 15 days prior to enrollment into the study, significant cardiovascular, liver, or renal functional abnormality, serum glucose level less than the lower limit of normal, history of allergy to or hypersensitivity	(a) Randomized double-blind, double-dummy, non-inferiority trial<br>(b) 28 days<br>(c) July 1998 to October2001	- CQ: 0.6 g/day for 2 days and 0.3 g on day 3<br><br>- AZM: 1 g/day for 3 days	(a) Patients with treatment-related AEs (n):<br>24 CQ; 13 AZM; p=0.06<br>Discontinued for AEs (n):<br>2 CQ (maculopapular rash and severe pruritus); 0 AZM<br><br>Number of AEs (n):<br>35 CQ; 15 AZM; p= 0.002<br>Headache (n):<br>1 CQ; 0 AZM<br>Nausea (n):<br>5 CQ; 0 AZM<br>Vomiting (n):<br>8 CQ; 0 AZM<br>Musculoskeletal (n):<br>0 CQ; 2 AZM<br>Respiratory (n):<br>0 CQ; 2 AZM<br>Rash (n):<br>3 CQ; 0 AZM<br>Pruritus (n):<br>8 CQ; 1 AZM	4

					to CQ, AZ or other macrolides, history of a transfusion of red blood cells within the prior 28 days, to have any situation that would prevent the patient from returning to follow-up visits				
Efficacy, safety, and tolerability of amodiaquine plus sulphadoxine-pyrimethamine used alone or in combination for malaria treatment in pregnancy: a randomised trial. Tagbor H. Lancet. 2006	CQ	900 (+ 220 control group with negative results from the malaria antigen test)	Primary outcome: To assess the parasitological failure by day 28 of treatment<br>Secondary outcomes: To compare the effect of study drugs on maternal haemoglobin, occurrence of adverse events, liver and bone marrow function, and perinatal outcomes	CQ group:<br>Age: 23.1 (5.6)[b]<br><br>Gender (female): 100%	Inclusion criteria:<br>- Pregnant women with a gestational age of 16 w or more with peripheral blood parasitaemia<br>Exclusion criteria:<br>- Women who had a multiple pregnancy, had severe malaria, or were enrolled previously in the current study	(a) Randomized, double-bind<br>(b) 28 days<br>(c) March 2003 to September 2004	Four groups + control group:<br>- 225 patients: CQ 600 mg for 2 days and 300 mg on the third day<br>- 225 patients: amodiaquine 600 mg for 2 days and 300 mg on the third day<br>- 225 patients: sulphadoxine 1500 mg and pyrimethamine 75 mg as 1 dose<br>- 225 patients: amodiaquine 600 mg, sulphadoxine 1500 mg and pyrimethamine 75 mg on day 1, amodiaquine 600 mg on day 2 and amodiaquine 300 mg on day 3<br><br>- 220 patients: control group	(a) CQ group:<br>Day 3 after start of treatment:<br>- Reported side effects: 163 (76)[c]<br>- General weakness: 106 (49)[c]<br>- Dizziness: 97 (45)[c]<br>- Vomiting: 70 (33)[c]<br>- Itching: 88 (41)[c]<br>- Nausea: 50 (23)[c]<br><br>Day 7 after start of treatment:<br>- Reported side effects: 67 (31)[c]<br>- General weakness: 49 (22)[c]<br>- Dizziness: 34 (16)[c]<br>- Vomiting: 31 (14)[c]<br>- Itching: 18 (8)[c]<br>- Nausea: 33 (15)[c]	5
Therapeutic response of multidrug-resistant Plasmodium falciparum and *P. vivax* to chloroquine and sulfadoxine-pyrimethamine in southern Papua, Indonesia. Ratcliff A. Trans R Soc Trop Med Hyg. 2007	CQ	143	To determine the efficacy of protocols recommended in 2004 for uncomplicated falciparum and vivax malaria	CQ group:<br>Age: 14.5 (1.6-60)[f]<br>Gender (male): 55%	Inclusion criteria:<br>- Consecutive patients with slide-confirmed malaria with any parasitaemia and a fever or a history of fever during the preceding 48 h presenting to the outpatient clinic<br>Exclusion criteria:<br>- Pregnant or lactating women<br>- Children under 10 kg<br>- Patients with signs of severity, parasitaemia > 4% or concomitant disease requiring hospital admission	(a) Open label<br>(b) Patients were followed for 42 days in the falciparum arm and 28 days in the vivax arm<br>(c) April to September 2004	- 103 patients received standard treatment of CQ+SP for *P. falciparum* (alone or mixed with *P. vivax*)<br>- 40 patients were treated with CQ for pure *P. vivax* infections were administered	(a) Vomiting:<br>1) 10% of patients (4/40) treated with CQ<br>2) Vomiting after CQ was 7.2-fold (95% CI 1.7–31) higher in children compared with adults: 19% (13/68) and 3% (2/75), respectively (p = 0.003)	1

Biological and haematological safety profile of oral amodiaquine and chloroquine in healthy volunteers with or without Plasmodium falciparum infection in northeast Tanzania. Massaga JJ. Tanzan J Health Res. 2008	CQ	40	To evaluate safety, tolerability and efficacy of AQ in semi-immune indigenous adult healthy volunteers with and without *P. falciparum* infection in Tanzania	CQ groups:<br>Age:<br>29.4 (9.28)[c] parasite -<br>22.1 (3.73)[c] parasite +<br><br>Gender (male):<br>100%	Inclusion criteria:<br>- Aged 15-45 years<br>- Malaria parasite negative or positive asymptomatic<br>- No history of fever<br>- Free from medication including intake of antimalarials for the past 4 weeks<br>- Without history of congenital abnormalities or chronic and severe diseases<br>Exclusion criteria:<br>- Contraindication for the use of test drug especially previous history of sore throat with amodiaquine	(a) Randomized, open-label, single-blind<br>(b) 15 days<br>(c) NA	- 20 patients in CQ group (10 with parasite +) received 25mg base/kg body weight for consecutive three days<br><br>- 20 patients in amodiaquine group (10 with parasite +)	(a) CQ group:<br>Abdominal discomfort:<br>1 (10)[c] parasite -; 5 (50)[c] parasite +<br>Headache:<br>3 (30)[c] parasite -; 2 (20)[c] parasite +<br>Body malaise:<br>2 (20)[c] parasite -; 2 (20)[c] parasite +<br>Fever:<br>2 (20)[c] parasite -; 1 (10)[c] parasite +<br>Biological and haematological charactristics changes at day seven compared to pre-trial:<br>ALT: 1.50 (6.04)[b] parasite -; 2.40 (6.55)[b] parasite +<br>AST: 2 (2.63)[b] parasite -; 1.40 (2.41)[b]<br>Creatinine: 3.50 (12.6)[b] parasite -; 7 (19.39)[b] parasite +<br>Bilirubin: 0.20 (6.30)[b] parasite -; 0.40 (4.22)[b] parasite +<br>Leucocyte count: 0.86 (1.26)[b] parasite -; 0.76 (1.85)[b] parasite +<br>Neutophils count: 0.54 (1.57)[b] parasite -; 0.02 (1.29)[b] parasite +	2
Pyronaridine-artesunate versus chloroquine in patients with acute Plasmodium vivax malaria: a randomized, double-blind, non-inferiority trial. Poravuth Y. PLoS One. 2011	CQ	228	Compare the efficacy and safety of pyronaridine-artesunate with that of CQ for the treatment of P. vivax uncomplicated malaria	CQ groups:<br>Age:<br>26.4(10.9)[b]<br><br>Gender (male):<br>164 (71.9)[c]	Inclusion criteria:<br>-Aged 3 to 60 years<br>-microscopically confirmed P. vivax uncomplicated, acute, mono-infection<br>-Parasite density ≥ 250 parasites/µl (including at least 50% asexual parasites) and fever.<br>-Fever in the previous 24 h<br>-Body weight between 20 and 90 kg (with no clinical evidence of severe malnutrition)<br><br>Exclusion criteria:<br>-Other condition requiring hospitalization<br>-Anemia<br>-Hepatic or renal impairment<br>-Presence or history of clinically significant disorders	(a) Randomized, double-blind, non-inferiority<br>(b) 42 days<br>(c) March 2007 to March 2008	-CQ dose was 620 mg on Days 0 and 1 and 310 mg on Day 2<br>-CQ dose for children was 10 mg/kg on Days 0 and 1 and 5 mg/kg on Day 2	(a) CQ group:<br>Deaths:<br>0%<br>Treatment emergent adverse event of any cause:<br>31.6%<br>Vomiting:<br>1.8%<br>Fatigue:<br>0.4%<br>Bilirrubin increase:<br>0%<br>AST >5 upper limit:<br>0.43%<br>QT prolongation:<br>2.7%<br>Headache:<br>1.3%<br>Anorexia:<br>0.9%<br>Blood creatine phosphokinase increase:<br>3.5%<br>Dizziness:<br>2.2%	5

					-Hypersensitivity to study drugs or excipients<br>-Active hepatitis A IgM, hepatitis B surface antigen, hepatitis C antibody or seropositive for HIV antibody<br>-Used an antimalarial within the previous two weeks (urine test required)<br>-Used an antibacterial with anti-malarial activity within the previous two weeks -Received an investigational drug within the past four weeks<br>-Pregnant or lactating women				
Chloroquine and quinine: a randomized, double-blind comparison of efficacy and side effects in the treatment of Plasmodium falciparum malaria in the Philippines. Watt G. Trans R Soc Trop Med Hyg. 1988	CQ	20<br><br>CQ group:<br>10	Efficacy and side effects of CQ and quinine in the treatment of P. falciparum malaria in Philippines	CQ groups:<br>Age:<br>30(11)[b]<br><br>Gender (male):<br>100%	Inclusion criteria:<br>-Male<br>-≥ 16 years<br>->1000 asexual P. falciparum parasites/µl of blood<br>-Informed consent<br>Exclusion criteria:<br>-Seriously ill<br>-Acquired malaria outside the Philippines<br>-History of vomiting<br>-Treatment with antimalarials during the 3 weeks before hospital admission	(a) Randomized, double-blind<br>(b) NA<br>(c) NA	-CQ dose: 25 mg/kg over 3 days	(a) CQ group:<br>Diarrhea:<br>20%<br>Vomiting:<br>10%<br>Pruritus:<br>10%	4

Abbreviations: AEs: adverse events; ALT: alanine aminotransferase; AST: aspartate aminotransferase; CQ: chloroquine; CQ-P: chloroquine and proguanil; CQTE: chloroquine effervescent tablets; CQTU: chloroquine uncoated tablets; g: grams; h: hours; kg: kilograms; mg: milligram; NA: not available/not applicable; w: weeks.

[a]Jadad, AR et al. Assessing the quality of reports of randomized clinical trials: is blinding necessary? Control Clin Trials. 1996; 17(1):1–12.
Values are: [b]mean (SD); [c] n (%); [d]mean (range); [e]median (interquartile range); [f]median (range). [g]mean (SD) [range]; creatinine in mmol/L; bilirubin in mmol/L; leucocyte count x 10^9; neutrophils count x 10^9

Table S18. Characteristics of included studies: clinical trials, patients affected by rheumatoid arthritis.

Study ID: title, first author, journal of publication, year	Drug (CQ/ HCQ)	Sample Size	Aim	Age (years) and Gender	Patient Inclusion and Exclusion Criteria	Main study characteristics: (a) Type of study (b) Time Horizon (c) Time Frame	Regimens	Adverse event characteristics: (a) Incidence (	Jadad quality score[a]
A double-blind comparative study of hydroxychloroquine and dapsone, alone and in combination, in rheumatoid arthritis. Haar D. Scand J Rheumatol. 1993	HCQ	80	To compare the efficacy and toxicity of HCQ, dapsone and a combination of both drugs in treatment of rheumatoid arthritis	HCQ-placebo group: Age: 58.3 (31-86)[f]<br><br>Gender (ratio male:female): 7:21	Inclusion criteria:<br>- Patients with probable, definite or classical rheumatoid arthritis whose active disease was not adequately controlled by nonsteroidal anti-inflammatory drugs<br>- Patients were included if they fulfilled at least 2 of the following criteria: tenderness of 3 or more joints, swelling of 1 or more joints, erythrocyte sedimentation rate more than 28 mm/h and serum haptoglobin concentration more than 2.8 g/l<br>Exclusion criteria:<br>- Patients with a history of untoward effects to sulfones or chloroquines, previous nonresponse to dapsone or chloroquines, antimalarial induced retinopathy and significant renal, hepatic or hematologic disease<br>- Patients treated with disease-modifying drugs during the preceding 2 w	(a) Randomized, double-bind<br>(b) 24 w<br>(c) NA	Three groups:<br>- 28 patients: HCQ 250 mg/day plus dapsone placebo<br>- 27 patients: dapsone 100 mg/day plus HCQ placebo<br>- 25 patients: HCQ 250 mg/day plus dapsone 100 mg/day	(a) HCQ-placebo group:<br>Three patients discontinued the drug due to AE, but only 1 of them stopped the treatment at 2 w (dyspepsia)	5
Prednisone treatment of elderly-onset rheumatoid arthritis. Disease activity and bone mass in comparison with chloroquine treatment. van Schaardenburg D. Arthritis & Rheumatology. 1995	CQ	56	To compare disease activity and bone mass during long-term treatment with prednisone versus CQ in this patient population	Age (years): 70[b]<br><br>Gender (male): 57%	Inclusion criteria:<br>- Rheumatoid arthritis<br>- ≥ 60 years old<br>- Active disease that was unresponsive to 3 months of nonsteroidal antiinflammatory therapy<br>Exclusion criteria:	(a) Randomized<br>(b) 2 years<br>(c) NA	Patients were randomized to receive prednisone (15 mg/day for 1 month, with the dosage tapered as low as possible thereafter) (n = 28) or CQ (n = 28)<br><br>The CQ dosage was 0.1 g/day after a loading phase of 0.1 g 3 times daily during the first	(a) In the CQ group, 11 patients (39%) prematurely discontinued the study treatment, 5 patients due to AEs. The AEs that led to CQ discontinuation were nausea and/or rash. The first 3 patients who were started on the CQ regimen could not tolerate the 0.3 g/day loading dose. Thus, for subsequent patients, the loading phase of CQ was changed. Of all patients	2

					- Use of a DMARD or prednisone during the preceding 3 months<br>- Use of thiazide diuretics, inadequately controlled hypertension<br>- Active peptic ulcer disease, diabetes mellitus, hepatic disease, renal disease<br>- Ophthalmologic contraindications to CQ treatment<br>- Osteoporosis with vertebral fracture of the thoracic and lumbar spine and other serious diseases		month and 0.1 g twice daily during the second month	randomized to receive CQ, 10 (36%) could not tolerate the full loading dose	

Abbreviations: AEs: adverse events; CQ: chloroquine; g: grams; h: hours; HCQ: hydroxychloroquine; kg: kilograms; mg: milligram; NA: not available/not applicable; w: weeks.

[a]Jadad, AR et al. Assessing the quality of reports of randomized clinical trials: is blinding necessary? Control Clin Trials. 1996; 17(1):1–12.
Values are: [b]mean (SD); [c] values are n (%); [d] values are mean (range); [e] values are median (interquartile range); [f]median (range).

Table S19. Characteristics of included studies: clinical trials, patients affected by other pathologies.

Study ID: title, first author, journal of publication, year	Drug (CQ/ HCQ)	Sample Size	Aim	Age (years) and Gender	Patient Inclusion and Exclusion Criteria	Main study characteristics: (a) Type of study (b) Time Horizon (c) Time Frame	Regimens	Adverse event characteristics: (a) Incidence	Jadad quality score[a]
Randomized trial of prolonged chloroquine therapy in advanced pulmonary sarcoidosis. Baltzan M. Am J Respir Crit Care Med. 1999	CQ	23	To investigate the benefits of prolonged CQ therapy in symptomatic patients with biopsy-proven pulmonary sarcoidosis	Age: 42.5 (29-67)[d]<br><br>Gender (female): 10[c]	Inclusion criteria:<br>- Presence of pathologically documented sarcoidosis<br>- Evidence of symptomatic pulmonary disease for at least 2 years, radiographic disease of stage 2 or 3<br>- Evidence of physiologic abnormalities on pulmonary function testing<br>Exclusion criteria:<br>- Subjects with homozygous G6PDH	(a) Randomized, open label<br>(b) NA<br>(c) NA	All 23 patients were initially treated for 6 months with CQ 750 mg/d, tapering every 2 months to 250 mg/day. Eighteen patients were then randomized to either a maintenance group (CQ 250 mg/day) or to an observation group (no CQ)	(a) Three patients abandoned CQ due to side effects. The reasons for abandoning CQ included abdominal pain, generalized maculopapular rash, or exacerbation of a preexisting anxiety disorder	1
Hydroxychloroquine treatment of patients with human immunodeficiency virus type 1. Sperber K. Clin Ther. 1995	HCQ or placebo	38<br><br>Groups:<br>HCQ: 19<br><br>Placebo: 19	To evaluate HCQ activity in suppressing HIV-1 replication and improving immune function in infected patients	Age:<br>HCQ: 39.1 (6.6)[b]<br><br>Placebo: 40.6 (12.5)[b]<br><br>Gender (male):<br>HCQ: 15<br><br>Placebo: 18	Inclusion criteria;<br>-CD4+ counts between 200 and 500 cells/mm^3<br>-Asymptomatic<br>-Not receiving any antiretroviral agents<br>-All women of childbearing potential had to have negative pregnancy test results 2 weeks before the start of the study and use birth control methods.<br>-Have hemoglobin levels of ≥ 8.5 g/dL<br>-Neutrophil counts of ≥ 1000 cells/mm^3<br>-Platelet counts of ≥ 75,000 cells/mm^3<br>-AST, ALT and ALP < 3 times the upper limit of normal<br>-Amylase levels < 1.3 times the upper limit of normal<br><br>Exclusion criteria:<br>-Younger than age 18<br>-Women who were breast-feeding	(a) Randomized, double-blind, placebo-controlled<br>(b) 8 weeks<br>(c) NA	Treatment group:<br><br>HCQ 0.8 g QD for 8 weeks	(a) Adverse reactions to the study drug: 0%<br><br>Ophthalmologic examinations (Amsler grid, color discrimination, and visual acuity) changes before and after the study: 0%	3

					-G6PD deficiency<br>-Had AIDS-defining infections<br>-Malignancies (Kaposi's sarcoma)<br>-Were actively abusing alcohol or drugs<br>-Stage 2 AIDS dementia complex<br>-Noncompliance in taking the study medication				
Impact of hydroxychloroquine therapy on chronic urticaria: chronic autoimmune urticaria study and evaluation. Reeves GE. Intern Med J. 2004	HCQ or placebo	18<br><br>Groups:<br>HCQ: 9<br><br>Placebo: 9	To evaluate the efficacy of HCQ in patients with chronic idiopathic urticaria	Age (years): 38.2<br><br>Gender: Male : female ratio: 5:1	Inclusion criteria:<br>-Had history of urticaria occurring two or more times per week over a continuous period of more than 6 weeks.<br>-> 18 years<br>-Able to give informed consent<br><br>Exlcusion criteria:<br>-Other underlying aetiologies (thyroid disease, vasculitis, lupus and related disorders, food intolerance, specific allergies)<br>-Patients already receiving HCQ or any other antimalarial agent<br>-History of previous toxicity from 4-aminoquinoline compounds	(a) Randomized, blinded<br>(b) 12 weeks<br>(c) January and December 2001	NA	(a) Significant toxicity form HCQ during the course of the trial:<br>0%	2
A randomized, double-blind, window of opportunity trial evaluating the effects of chloroquine in breast cancer patients. Arnaout A. Breast Cancer Res Treat. 2019	CQ or placebo	70<br><br>Groups:<br>CQ: 46<br><br>Placebo: 24	Effect of single-agent CQ on breast tumour cellular proliferation	Age (years):<br>CQ: 57.4 (9.7)[b]<br><br>Placebo: 55.7 (8.4)[b]<br><br>Gender (female): 100%	Inclusion criteria:<br>-Biopsy proven operable invasive cancer clinically and/or radiologically (sup o igual) 1.5 cm in size<br>-Surgery planned for 2-6 w after initial consultation<br>-ECOG performance status 0-2<br><br>Exclusion criteria:<br>-Recurrent or metastatic breast cancer	(a) Randomized, doble-blind, placebo-controlled window of opportunity trial<br>(b) NA<br>(c) September 2015 to December 2016	Treatment group:<br><br>CQ: 0.5 g QD for 2 to 6 weeks prior to breast surgery	(a) All reported adverse events were classified as grade 1<br><br>n<br>CQ completed: 4 (11.1)[c]<br>CQ withdrawn: 7 (70)[c]<br>Total CQ: 11 (23.9)[c]<br>Placebo: 3 (12.5)[c]<br><br>Diarrhea:<br>CQ completed: 5 (13.9)[c]<br>CQ withdrawn: 3 (30)[c]<br>Total CQ: 8 (17.4)[c]	5

					-Currently on or have been exposed to CQ or HCQ within the past 3 months<br>-Pregnant<br>-Actively nursing<br>-History of auditory, retinal or ocular pathology, psoriasis, epilepsy, seizures or G6PD deficiency.<br>-Baseline ECG withQT prolongation based on QT_C interval > 450 ms<br>-Baseline abnormal hepatic function (serum AST or ALT > 3 x upper limit of normal)<br>-Creatinine > 100 umol/L			Placebo: 1 (4.2)[c]<br><br><u>Dizziness:</u><br>CQ completed: 1 (2.8)[c]<br>CQ withdrawn: 3 (30)[c]<br>Total CQ: 4 (8.7)[c]<br>Placebo: 3 (12.5)[c]<br><br><u>Fatigue:</u><br>CQ completed: 1 (2.8)[c]<br>CQ withdrawn: 0 (0)[c]<br>Total CQ: 1 (2.2)[c]<br>Placebo: 1 (4.2)[c]<br><br><u>Visual symptoms:</u><br>CQ completed: 2 (5.6)[c]<br>CQ withdrawn: 2 (20)[c]<br>Total CQ: 4 (8.7)[c]<br>Placebo: 0 (0)[c]<br><br><u>Auditory symptoms:</u><br>CQ completed: 1 (2.8)[c]<br>CQ withdrawn: 0 (0)[c]<br>Total CQ: 1 (2.2)[c]<br>Placebo: 0 (0)[c]<br><br><u>Muscle weakness:</u><br>CQ completed: 1 (2.8)[c]<br>CQ withdrawn: 3 (30)[c]<br>Total CQ: 4 (8.7)[c]<br>Placebo: 0 (0)[c]<br><br><u>Dry mouth:</u><br>CQ completed: 1 (2.8)[c]<br>CQ withdrawn: 1 (10)[c]<br>Total CQ: 2 (4.3)[c]<br>Placebo: 0 (0)[c]<br><br><u>Documented visual changes:</u><br>CQ completed: 0 (0)[c]<br>CQ withdrawn: 0 (0)[c]<br>Total CQ: 0 (0)[c]<br>Placebo: 0 (0)[c]<br><br>All the symptoms are recognized side effects of CQ	

								All the above symptoms subsided once the medication ceased	
On chikungunya acute infection and chloroquine treatment. De Lamballerie X. Vector Borne Zoonotic Dis. 2008	CQ or place bo	54<br><br>Groups :<br>CQ: 27<br><br>Placebo : 27	Efficacy and safety of CQ as a therapeutic treatment of chikungunya disease	NA	Inclusion criteria:<br>-Adult patients (18-65 years old, men and women, without contraindications) who volunteered to take part in the study<br>-Residing at the Reunion Island<br>-≥ 60 kg<br>-Typical presentation of acute chikungunya disease (defined by acute febrile arthralgia)<br>-Diagnosed within < 48 h<br><br>Exclusion criteria:<br>-Pregnancy<br>-Contraindications to CQ<br>-Renal insufficiency<br>-Retinopathy<br>-Celiac disease	(a) Randomized, double-blind, placebo-controlled<br>(b) 200 days<br>(c) Was started on May 20, 2006	Treatment group:<br><br>CQ: 0.6 g (one dose) at day 1, 0.6 g (0.3 g twice daily) at days 2 and 3, and 0.3 g at days 4 and 5 (total dose: 2.4 g; duration of treatment: 5 days)	(a) Mild adverse reactions (mainly nausea and pruritus):<br>CQ: 7 (25.9)[c]<br>Placebo: 0 (0)[c]<br>$p < 0.01$<br><br>Interruption of the treatment:<br>CQ: 0 (0)[c]<br>Placebo: 0 (0)[c]	2
A randomized controlled trial of chloroquine for the treatment of dengue in Vietnamese adults. Tricou V. PLoS Negl Trop Dis. 2010	CQ or place bo	307<br><br>Groups :<br>CQ: 153<br><br>Placebo : 154	To evaluate CQ as potential antiviral therapy in a randomized, double-blind placebo-controlled trial of adolescents and adults with dengue	Age (years):<br>CQ: 22 (18-27)[e]<br><br>Placebo: 22 (19-28)[e]<br><br>Gender (male):<br>CQ: 104 (68)[c]<br><br>Placebo: 106 (68.8)[c]	Inclusion criteria:<br>- ≥15 years<br>-Had a self-reported illness history of 72 h or less and were suspected of having dengue<br><br>Exclusion criteria:<br>-Pregnant<br>-Receiving therapy for other chronic disorders<br>-History of hypersensitivity to CQ<br>-Written consent from either the patient or a parent was not obtained	(a) Randomized, double-blind, placebo-controlled, parallel-group<br>(b) 10-14 days hospital discharge<br>(c) May 2007 to July 2008	Treatment group:<br><br>CQ: 0.6 g base on study enrolment, then 0.6 g on day 2 and 0.3 g on day 3	(a)Intention to treat population:<br>Patient with adverse reaction:<br>CQ: 18 (11.8)[c]<br>Placebo: 6 (3.9)[c]<br>$p = 0.01$<br>Patient with vomiting:<br>CQ: 15 (9.8)[c]<br>Placebo: 6 (3.9)[c]<br>$p = 0.04$<br>Patient requiring IV fluid:<br>CQ: 21 (13.7)[c]<br>Placebo: 11 (7.1)[c]<br>$p = 0.06$<br>Per protocol population:<br>Patient with adverse reaction:<br>CQ: 17 (13.6)[c]<br>Placebo: 5 (4.1)[c]<br>$p = 0.01$<br>Patient with vomiting:<br>CQ: 14 (11.2)[c]<br>Placebo: 5 (4.1)[c]<br>$p = 0.05$<br>Patient requiring IV fluid:	5

								CQ: 19 (15.2)[c]<br>Placebo: 10 (8.2)[c]<br>p = 0.11	
Chloroquine use improves dengue-related symptoms. Borges MC. Mem Inst Oswaldo Cruz. 2013	CQ or placebo	37<br><br>Groups:<br>CQ: 19<br><br>Control: 18	Effect of CQ in patients with dengue	Age (years):<br>CQ: 32.72 (11.5)[b]<br><br>Gender(male):<br>CQ: 8 (42)[c]	Inclusion criteria:<br>-Dengue-related symptoms, such as fever and at least two other symptoms, such as headache, retro-orbital pain, muscle and bone or joint pain, nausea, vomiting and rash, for < 72h<br><br>Exclusion criteria:<br>-Pregnant<br>-< 18 years old<br>-Cardiac or neurologic disease	(a) Randomized, double-blind<br>(b) NA<br>(c) February to May 2008	CQ: 0.5 g CQ (0.3 g base) BD for 3 days	(a) Blurred vision:<br>CQ: 1(5.2)c<br><br>Loss of consciousness: 1<br>CQ: 1(5.2)c<br><br>The latter patient was severely dehydrated and seizure was ruled out based on clinical presentation and follow-up. Both patients were not confirmed to be dengue patients.	4
Chloroquine in the treatment of infectious mononucleosis. Cowley RG. Ann Intern Med. 1962	CQ	40<br><br>CQ group: 20	Determine the effect of CQ on the clinical and laboratory course of mononucleosis	Age (years):<br>20.5(17-25)[d]<br>Gender (male):<br>100%	Inclusion criteria:<br>-Suspected of having infectious mononucleosis<br>- Absolute lymphocytosis with with atypical lymphocytes, significant lymphadenopathy, fever, pharyngitis, and a changing heterophile antibody titer reaching a peak of 1:224 or greater.	(a) Double-blind<br>(b) NA<br>(c) November 1960 to May 1962	CQ dose: 1 g immediately, 0.5 g in 8 hours, 0.5 g QD for 9 days, and 0.25 g QD for 8 additional days	(a) No significant side effects of CQ were noted<br>60% of patients treated with CQ suffered gastrointestinal complaints (anorexia, nausea, vomiting)	3
Treatment of infectious mononucleosis. Schumacher HR. Ann Intern Med. 1963	CQ	100<br><br>CQ group: 5	Relate the results of a double blind study concerning the treatment of infectious mononucleosis	Age (years):<br>mean: 19.6<br>Gender (male):<br>100%	Inclusion criteria:<br>-Adequate clinical picture<br>-Significant peripheral blood changes<br>-Characteristic antibody heterophile antibody reaction	(a)Double-blind<br>(b) NA<br>(c) March 1961 to May 1962	CQ dose: 1g of CQ shortly after arriving at the hospital. Then, 0.5 g every 6 hours for 24 hours and, finally, 0.25g every 6 hours for 5 days	(a) No complications were described in the CQ group	3

Abbreviations CQ: chloroquine; G6PDH glucose-6-phosphate dehydrogenase; mg: milligrams; NA: not available/not applicable; AIDS: acquired immunodeficiency syndrome; AST: aspartate aminotransferase; ALT:alanine aminoransferase; ALP: alkakline phosphatase; w: week; ECG: electrocardiogram; h: hour; DENV: dengue virus;

[a]Jadad, AR et al. Assessing the quality of reports of randomized clinical trials: is blinding necessary? Control Clin Trials. 1996; 17(1):1–12.

Values are: [b]mean (SD); [c]values are n (%); [d]values are mean (range); [e]values are median (interquartile range); [f]median (range).

Table S20. Characteristics of included studies: clinical trials and observational studies, patients affected by malaria or who received prophylactic treatment with CQ or HCQ in combination with other drugs.

Study ID: title, first author, journal of publication, year	Drug (CQ/ HCQ)	Sample Size	Aim	Age (years) and Gender	Patient Inclusion and Exclusion Criteria	Main study characteristics: (a) Type of study (b) Time Horizon (c) Time Frame	Regimens	Adverse event characteristics: (a) Measure	Jadad quality score[a]
The effects of prednisolone and niacin on chloroquine-induced pruritus in malaria. Ajayi AA. Eur J Clin Pharmacol. 1991	CQ phos phat e	Prednisolone: 10<br>Niacin: 8<br>Placebo: 10	To evaluate the safety, the prophylactic and palliative antipruritic effects of prednisolone and niacin in historically established CQ pruritus reactors, with malaria fever	Age:<br>Prednisolone: 27 (9)[b]<br>Niacin: 22 (5)[b]<br>Placebo: 27 (12)[b]<br><br>Gender (male/female):<br>Prednisolone: 5/5<br>Niacin: 2/6<br>Placebo: 3/7	Inclusion criteria:<br>- Adult febrile patients with clinical malaria, supported by presence of asexual stage in the blood, who are historically established (reacted to CQ with pruritus within 6 months) pruritus hyper-reactors to CQ<br><br>Exclusion criteria:<br>- Failure to consent, pruritus non-reactors, children and patients with complicated malaria, absence of plasmodium parasitemia, patients unable to take CQ orally	(a) Randomized, double blind, placebo controlled, parallel group comparison<br>(b) 72 h<br>(c) NA	CQ: 0.6 g base of CQ given at time 0, 0.3 g base at 6 h up to a total of 2.1 g of CQ base.<br><br>Prednisolone: 5 mg.	(a) Pruritus:<br>100 %	3
Chloroquine-induced pruritus in malaria fever: contribution of malaria parasitemia and the effects of prednisolone, niacin, and their combination, compared with antihistamine. Adebayo RA. Br J Clin Pharmacol. 1997	CQ	Promethazine: 7<br>Niacin: 6<br>Prednisolone: 9<br>Prednisolone + niacin: 6	To examine and compare the antipruritic effects of promethazine, niacin, prednisolone and their combination on pruritus induced by CQ in historical itching patients with parasitologically proven malaria fever. To evaluate the role of the antecedent malaria parasite density in	Age:<br>Promethazine: 28 (9)[b]<br>Niacin: 29 (8)[b]<br>Prednisolone: 29 (8)[b]<br>Prednisolone + niacin: 23 (8)[b]<br><br>Gender (male/female):<br>Promethazine: 5/2<br>Niacin: 3/3	Inclusion criteria:<br>- Adult febrile patients with clinical malaria, supported by presence of asexual stage in the blood, who are historically established (reacted to CQ with pruritus within 6 months) pruritus hyper-reactors to CQ<br><br>Exclusion criteria:<br>- Failure to consent, pruritus non-reactors, children and patients with complicated malaria, absence of plasmodium	(a) Randomized, double-blind, parallel group study<br>(b) 72 h<br>(c) NA	CQ: 2.1 g of CQ base total dose<br><br>Prednisolone: 10 mg.	(a) Pruritus:<br>100 %	3

			the severity of CQ pruritus intensity	Prednisolone:<br>4/5<br>Prednisolone + niacin:<br>2/4	parasitemia and patients unable to take CQ orally				
A multicenter study of azithromycin, alone and in combination with chloroquine, for the treatment of acute uncomplicated *Plasmodium falciparum* malaria in India. Dunne MW. J Infect Dis. 2005	CQ base	Double-blind monotherapy:<br>AZM: 16<br>CQ :16	To explore the use of AZM monotherapy or in combination therapy with 4-aminoquinolines in the treatment of acute uncomplicated malaria due to *P. falciparum*	Age (mean):<br>AZM: 30.2<br>CQ: 31.8<br><br>Gender (male):<br>AZM: 88 %<br>CQ: 94 %	Inclusion criteria:<br>- Falciparum malaria, informed consent, 18-65 years, < 100,000 parasites/µl on baseline blood smear and, during the preceding 48 h, fever > 38 ºC, women with negative hCGβ test and willing to use adequate contraception both during and for 3 months after the study<br><br>Exclusion criteria:<br>- Impaired consciousness, jaundice, ± respiratory distress, history of hematuria, serum glucose level less than the lower limit of normal, pregnant or lactating, treated with any antimalarial drug during the preceding 15 days, received a transfusion of red blood cells during the preceding 28 days ± significant abnormality of cardiovascular, liver or renal function	(a) Randomized, placebo-controlled, double-blind<br>(b) 28 days<br>(c) July 1998 to October 2001	AZM: 1g for 3 days<br><br>CQ: 0.6 g for 2 days and 0.3 g on day 3	(a) Patients with treatment-related AEs:<br>9 (56)[c] CQ; 8 (50)[c] AZM<br>Patients who discontinued because of AEs:<br>0 CQ; 0 AZM<br>Total of AEs (n):<br>10 CQ; 8 AZM<br>Diarrhoea:<br>1 (6)[c] CQ; 2 (13)[c] AZM<br>Nausea:<br>0 CQ; 0 AZM<br>Vomiting:<br>0 CQ; 0 AZM<br>Dizziness:<br>3 (19)[c] CQ; 1 (6)[c] AZM<br>Pharyngitis:<br>1 (6)[c] CQ; 0 AZM<br>Pruritus:<br>3 (19)[c] CQ; 1 (6)[c] AZM<br>Other:<br>2 (19)[c] CQ; 4 (25)[c] AZM	4
		Open-label combination:<br>AZM plus CQ: 64		Age (mean):<br>AZM plus CQ: 31.7<br><br>Gender (male):<br>AZM plus CQ: 83 %		(a) Randomized, open-label combination therapy<br>(b) 28 days<br>(c) July 1998 to October 2001		(a) Patients with treatment-related AEs:<br>13 (20)[c] AZM plus CQ<br>Patients who discontinued because of AEs:<br>0 AZM plus CQ<br>Total of AEs (n):<br>18 AZM plus CQ<br>Diarrhoea:<br>2 (3)[c] AZM plus CQ<br>Nausea:<br>4 (6)[c] AZM plus CQ<br>Vomiting:<br>5 (8)[c] AZM plus CQ<br>Dizziness:<br>0 AZM plus CQ<br>Pharyngitis:<br>0 AZM plus CQ	2

								Pruritus:<br>1 (2)[c] AZM plus CQ<br>Other:<br>6 (10)[c] AZM plus CQ	
Efficacy and safety of a combination of azithromycin and chloroquine for the treatment of uncomplicated *Plasmodium falciparum* malaria in two multi-country randomized clinical trials in African adults. Sagara I. Malar J. 2014	CQ base	Double-blind:<br>AZM plus CQ : 114<br><br>MQ: 115	To confirm the hypothesis that AZM plus CQ is non-inferior to MQ for the treatment of symptomatic, uncomplicated malaria due to *P. falciparum*<br><br>To compare AZM plus CQ versus MQ to treat uncomplicated *P. falciparum* malaria	Age:<br>AZM plus CQ: 29.4 (9.6)[b]<br>MQ: 30.2 (11)[b]<br><br>Gender (male):<br>AZM plus CQ: 66<br>MQ: 61	Inclusion criteria:<br>≥ 18 years, symptomatic uncomplicated malaria (blood smears positive for *P. falciparum* asexual parasitemia between 1,000 and 100,000 parasites /µL) and fever, documented or by history, within the prior 24 h, serum glucose ≥ 60 mg/dl and a rapid diagnostic test positive for *P. falciparum*, women of childbearing potential: negative urine hCG test, adequate contraception during the study<br><br>Exclusion criteria:<br>- Severe or complicated malaria, non-falciparum species, pregnancy or breast-feeding, allergy or hypersensitivity to AZM, CQ, MQ or related, history of epilepsy or psoriasis, treatment with any antimalarial drug or with any antibacterial with known antimalarial activity within 2 w before enrolment, abnormal renal or liver function, any major psychiatric disorder, inability to swallow oral medication, treatment with other investigational drugs 30 days prior to enrolment,	(a) Double-blind phase II, multi-center, randomized, comparative, non-inferiority<br>(b) 42 days<br>(c) June 2004 to May 2006	AZM plus CQ 1 g:<br>AZM 1 g + CQ 0.6 g/24 h for 3 days<br><br>AZM plus CQ 500 mg:<br>AZM 0.5 g + CQ 0.6 g/24 h for 3 days → Stopped early<br><br>MQ 1.25 g (salt equivalent to 28 mg free base):<br>0.75 g followed by 0.5 g on day 0	(a) Any AE:<br>89 (78.1)[c] AZM plus CQ; 71 (61.7)[c] MQ<br>Pruritus:<br>58 (50.9)[c] AZM plus CQ; 11 (9.6)[c] MQ<br>Dizziness:<br>11 (9.6)[c] AZM plus CQ; 26 (22.6)[c] MQ<br>Vomiting:<br>18 (15.8)[c] AZM plus CQ; 12 (10.4)[c] MQ<br>Headache:<br>15 (13.2)[c] AZM plus CQ; 11 (9.6)[c] MQ<br>Abdominal pain:<br>8 (7.0)[c] AZM plus CQ; 13 (11.3)[c] MQ<br>Nausea:<br>9 (7.9)[c] AZM plus CQ; 13 (11.3)[c] MQ<br>Asthenia:<br>6 (5.3)[c] AZM plus CQ; 11 (9.6)[c] MQ<br>Palpitations:<br>3 (2.6)[c] AZM plus CQ; 7 (6.1)[c] MQ<br>Diarrhoea:<br>6 (5.3)[c] AZM plus CQ; 5 (4.3)[c] MQ<br>Fatigue:<br>0 AZM plus CQ; 0 MQ<br>Pain:<br>2 (1.8)[c] AZM plus CQ; 2 (1.7)[c] MQ	4
		Open-label:<br>AZM plus CQ: 113<br><br>MQ: 116		Age:<br>AZM plus CQ: 30.2 (11.0)[b]<br>MQ: 31.2 (12.4)[b]<br><br>Gender (male):<br>AZM plus CQ: 65		(a) Open-label phase III, multi-center, randomized, comparative, non-inferiority<br>(b) 42 days	AZM plus CQ 1 g:<br>AZM 1 g + CQ 0.6 g /24 h for 3 days<br><br>MQ 1.25 g (salt equivalent to 28 mg free base):<br>0.75 g followed by 0.5 g on day 0	(a) Any AE:<br>80 (70.8)[c] AZM plus CQ; 72 (62.1)[c] MQ<br>Pruritus:<br>32 (28.3)[c] AZM plus CQ; 1 (0.9)[c] MQ<br>Dizziness:<br>18 (15.9)[c] AZM plus CQ; 19 (16.4)[c] MQ	3

				MQ: 63	alcohol and/or any other drug abuse, medication or other medical conditions that might have interfered with the evaluation of the study drug, inability to comprehend and/or unwillingness to follow the study protocol, prior participation in this study, patients who not lived continuously in a malaria-endemic area for at least the previous year	(c) November 2006 to September 2007		Vomiting:<br>4 (3.5)[c] AZM plus CQ; 20 (17.2)[c] MQ<br>Headache:<br>20 (17.7)[c] AZM plus CQ; 25 (21.6)[c] MQ<br>Abdominal pain:<br>13 (11.5)[c] AZM plus CQ; 9 (7.8)[c] MQ<br>Nausea:<br>10 (8.8)[c] AZM plus CQ; 12 (10.3)[c] MQ<br>Asthenia:<br>9 (8.0)[c] AZM plus CQ; 3 (2.6)[c] MQ<br>Palpitations:<br>0 AZM plus CQ; 0 MQ<br>Diarrhoea:<br>11 (9.7)[c] AZM plus CQ; 4 (3.4)[c] MQ<br>Fatigue:<br>4 (3.5)[c] AZM plus CQ; 6 (5.2)[c] MQ<br>Pain:<br>6 (5.3)[c] AZM plus CQ; 1 (0.9)[c] MQ	
Efficacy and safety of azithromycin-chloroquine versus sulfadoxine-pyrimethamine for intermittent preventive treatment of *Plasmodium falciparum* malaria infection in pregnant women in Africa: an open-label, randomized Trial. Kimani J. PLoS One. 2016	CQ	Allocated to IPTp with AZM plus CQ: 1,446<br><br>Allocated to IPTp with SP: 1,445	To evaluate the efficacy and safety of IPTp with AZM plus CQ versus IPTp-SP in pregnant women in East and Southern African countries, where SP is the current standard of care and antifolate resistance in *P. falciparum* was evident	Age:<br>23.3 (4.5) [16–35][g]<br><br>Gender (female):<br>100%	Inclusion criteria:<br>- Pregnant women of all gravidities, to carry a single fetus of 14 to 26 w of gestation, 16 to 35 years of age, willing and able to comply with all study procedures and to attend all scheduled follow-up visits<br><br>Exclusion criteria:<br>- Symptoms of malaria, severe anemia (hemoglobin < 8 g/dl), any condition requiring hospitalization, obstetric complications increasing the risk of sub-optimal pregnancy outcome, evidence of severe concomitant infection, to take antimalarial drugs within the past 4 w	(a) Randomized, open-label, multi-center, phase III<br>(b) Day 28-42 post-delivery<br>(c) October 2010 to November 2013	For the AZM plus CQ combination, a fixed-dose tablet formulation of AZM plus CQ 250/155 mg was used; each IPTp treatment course consisted of a 3-day course of AZM plus CQ 1,000/620 mg per day administered orally once daily on days 0, 1, and 2<br><br>For the SP regimen, a fixed-dose tablets of sulfadoxine 0.5 g plus pyrimethamine 25 mg was used; each treatment course consisted of a single dose of sulfadoxine 1.5 g plus pyrimethamine 75 mg administered orally once on day 0	(a) Participants with treatment-related AEs in mothers:<br>996 (68.9)[c] AZM plus CQ; 286 (19.8)[c] SP<br><br>Participants discontinued treatment due to AEs (mothers):<br>41 (2.8)[c] AZM plus CQ; 5 (0.3)[c] SP<br><br>Participants discontinued temporarily the treatment due to AEs (mothers):<br>11 (0.8)[c] AZM plus CQ; 1 (0.1)[c] SP<br><br>Most common AEs in mothers (≥ 5% of study participants in either treatment group):<br>Blood and lymphatic system disorders:<br>207 (14.3)[c] AZM plus CQ; 192 (13.3)[c] SP<br>Eye disorders:<br>146 (10.1)[c] AZCQ; 2 (0.1)[c] SP<br>Gastrointestinal disorders:	4

								856 (59.2)[c] AZM plus CQ; 269 (18.6)[c] SP<br>General disorders and administration site conditions:<br>344 (23.8)[c] AZM plus CQ; 120 (8.3)[c] SP<br>Infections and infestations:<br>435 (30.1)[c] AZM plus CQ; 498 (34.5)[c] SP<br>Investigations:<br>160 (11.1)[c] AZM plus CQ; 169 (11.7)[c] SP<br>Nervous system disorders:<br>660 (45.6)[c] AZM plus CQ; 271 (18.8)[c] SP<br>Pregnancy, puerperium and perinatal conditions:<br>78 (5.4)[c] AZM plus CQ; 75 (5.2)[c] SP<br><br>All event incidences occurred between the first dose of study drug and up to 35 days after the last dose of study drug<br><br>No deaths were considered related to study drug	
Parasitological clearance rates and drug concentrations of a fixed dose combination of azithromycin-chloroquine in asymptomatic pregnant women with *Plasmodium falciparum* parasitemia: an open-label, non-comparative study in sub-saharan africa. Phiri K. PloS One. 2016	CQ	168	To determine if AZM plus CQ could clear the malaria parasites from the blood during pregnancy with a similar efficacy as that demonstrated in previous adult treatment studies	Age:<br>18.8 (2.3)[b]<br>range: 16-34<br><br>Gender (female):<br>100 %	Inclusion criteria:<br>- Primi-or second gravida women, ≥16 to ≤ 35 years of age, second or third trimesters of pregnancy and asymptomatic peripheral *P. falciparum* parasitemia (counts of 80-100,000 cells / µl)<br><br>Exclusion criteria:<br>- Multiple gestations, any chronic illness that might have adversely affected fetal growth or viability, evidence of current obstetric complications, clinical signs and symptoms of malaria,	(a) Open-label, non-comparative<br>(b) 42 days<br>(c) March 2011 to October 2013	AZM plus CQ: 4 fixed dose combination tablets of AZM 0.25 g/CQ 0.155 g for 3 days	(a) All-causality treatment-emergent adverse events occurring in ≥5 mothers:<br>Vomiting:<br>35 (20.8)[c]<br>Dizziness:<br>33 (19.6)[c]<br>Pruritus:<br>13 (7.7)[c]<br>Infection parasitic:<br>12 (7.1)[c]<br>Headache:<br>10 (6.0)[c]<br>Generalized pruritus:<br>9 (5.4)[c]<br>Fatigue:<br>7 (4.2)[c]<br>Upper respiratory infection:<br>7 (4.2)[c]<br>Nausea:	2

					history of fever within prior 24h, baseline hemoglobin < 8 g/dl, use of antimalarial drugs in the previous 4 w, inability to tolerate oral treatment in tablet form, known allergy to AZM, CQ, SP or to any macrolides or sulfonamides, any medication that may have interfered with the evaluation of the study drug or that was contra-indicated during pregnancy, current history of smoking or alcohol/drug abuse, severe acute or chronic and medical or psychiatric condition or laboratory abnormality			6 (3.6)[c]	
Treatment of adults with acute uncomplicated malaria with azithromycin and chloroquine in India, Colombia, and Suriname. Kshirsagar NA. Res Rep Trop Med. 2017	CQ	<u>A0661120 study in India</u><br><br>AZM plus CQ 1 g: 83<br>AZM plus CQ 500 mg:<br>67<br>SPCQ: 80	To explore the use of AZM plus CQ for the treatment of malaria	<u>Age:</u><br>AZM plus CQ 1 g: 31.7 (12.9) [18-75][g]<br>AZM plus CQ 500 mg:<br>29.5 (9.6) [18-58][g]<br>SPCQ: 31.7 (11.1) [18-60][g]<br><br><u>Gender (male):</u><br>AZM plus CQ 1 g: 68<br>AZM plus CQ 500 mg: 61<br>SPCQ: 70	<u>Inclusion criteria:</u><br>≥ 18 years, uncomplicated symptomatic malaria as demonstrated by blood smears positive for *P. falciparum* asexual parasitemia between 1,000 and 100,000 parasites/µL and either documented fever or history of fever within the prior 24 h, to have a serum glucose ≥ 60 mg/dL, to have a rapid diagnostic test positive for *P.falciparum*	(a) Randomized<br>(b) 28 days<br>(c) September 2003 to January 2005	AZM plus CQ 1 g:<br>1g AZM plus 0.6 g base CQ /daily for 3 days<br><br>AZM plus CQ 0.5 g:<br>0.5 g AZM + 0.6 g base CQ for 3 days<br><br>SPCQ:<br>SP 1.5 g/0.075 g + 0.6 g CQ on days 0 and 1 plus 0.3 g CQ on day 2<br><br>AZM doses were blinded while SP and CQ doses were administered open label	(a) Discontinuation due to AEs:<br>0 AZM plus CQ 2 g<br>7 (6.3)[c] AZM plus CQ 1 g<br>0 AZM plus CQ 500 mg<br><br>Treatment-related AEs in > 2% of patients:<br><u>Any AE:</u><br>48 (44)[c] AZM plus CQ 2 g<br>52 (26)[c] AZM plus CQ 1 g<br>8 (10)[c] AZM plus CQ 500 mg<br>4 (5)[c] SPCQ<br>14 (12)[c] AP<br><u>Pruritus:</u><br>4 (4)[c] AZM plus CQ 2 g<br>30 (15)[c] AZM plus CQ 1 g<br>5 (6)[c] AZM plus CQ 500 mg<br>0 SPCQ	3

		A0661126 study in Colombia and Suriname<br><br>AZM plus CQ 1 g:<br>114<br>AZM plus CQ 500 mg: 14<br>AP: 116		Age:<br>AZM plus CQ 1 g: 34.6 (13.9) [18-68][g]<br>AZM plus CQ 500 mg:<br>41.4 (12.3) [24-68][g]<br>AP: 33.6 (18.1) [18-74][g]<br><br>Gender (male):<br>AZM plus CQ 1 g: 66<br>AZM plus CQ 500 mg: 9<br>AP: 64	Exclusion criteria:<br>- Clinical or laboratory evidence of severe or complicated malaria, pregnancy or breast feeding, history of allergy or hypersensitivity to AZM, any macrolide, or the relevant study drugs, history of epilepsy or psoriasis, treatment with any antimalarial drug or with any antibacterial with known antimalarial activity within 2 w prior to enrollment, impaired renal function, ALT and/or AST > 3x upper limit of normal, inability to swallow oral medication, treatment with other investigational drugs within 30 days prior to enrollment in the study, alcohol and/or any other drug abuse, requirement to use medication during the study that might interfere with the evaluation of the study drug, specific systemic diseases or other medical conditions that would interfere with the evaluation of the therapeutic response or safety of the drug, inability to comprehend and/or unwillingness to follow the study protocol, prior participation in these studies, patients in India: to have known or suspected folate deficiency or known history of G6PD	(a) Randomized<br>(b) 28 days<br>(c) July 2004 to July 2005	AZM plus CQ 1 g:<br>1g AZM + 0.6 g base CQ /daily for 3 days<br><br>AZM plus CQ 0.5 g:<br>0.5 g AZM + 0.6 g base CQ for 3 days<br><br>AP:<br>AP 0.25 g/ 0.1 g given as four capsules/day for 3 days<br><br>Study drug was administered as blinded therapy	2 (2)[c] AP<br>Diarrhoea:<br>13 (12)[c] AZM plus CQ 2 g<br>7 (4)[c] AZM plus CQ 1 g<br>0 AZM plus CQ 500 mg<br>0 SPCQ<br>4 (3)[c] AP<br>Paresthesia:<br>0 AZM plus CQ 2 g<br>6 (3)[c] AZM plus CQ 1 g<br>0 AZM plus CQ 500 mg<br>0 SPCQ<br>0 AP<br>Abdominal pain:<br>0 AZM plus CQ 2 g<br>5 (3)[c] AZM plus CQ 1 g<br>0 AZM plus CQ 500 mg<br>0 SPCQ<br>4 (3)[c] AP<br>Headache:<br>0 AZM plus CQ 2 g<br>3 (2)[c] AZM plus CQ 1 g<br>0 AZM plus CQ 500 mg<br>0 SPCQ<br>1 (1)[c] AP<br>Gastritis:<br>4 (4)[c] AZM plus CQ 2 g<br>4 (2)[c] AZM plus CQ 1 g<br>1 (1)[c] AZM plus CQ 500 mg<br>0 SPCQ<br>0 AP<br>Vomiting:<br>20 (18)[c] AZM plus CQ 2 g<br>7 (4)[c] AZM plus CQ 1 g<br>1 (1)[c] AZM plus CQ 500 mg<br>3 (4)[c] SPCQ<br>1 (1)[c] AP<br>Nausea:<br>33 (30)[c] AZM plus CQ 2 g<br>0 AZM plus CQ 1 g<br>0 AZM plus CQ 500 mg<br>0 SPCQ<br>0 AP<br>Dehydration:<br>4 (4)[c] AZM plus CQ 2 g<br>0 AZM plus CQ 1 g	5
		A0661154 study in Goa and Tumaco<br><br>AZM plus CQ 2 g: 110		Age:<br>30.8 (13) [18-77][g]<br><br>Gender (male):<br>85		(a) Non-comparative<br>(b) 28 days<br>(c) March 2006 to February 2008	2 g AZM and 0.6 g CQ/ day for 3 days		83[h] (STROBE)

					deficiency or other hematologic abnormalities, women of childbearing potential were required to have a negative urine gonadotropin test and to use adequate contraception during study			0 AZM plus CQ 500 mg<br>0 SPCQ<br>0 AP	
An evaluation of co-use of chloroquine or hydroxychloroquine plus azithromycin on cardiac outcomes: A pharmacoepidemiological study to inform use during the COVID19 pandemic. Vouri Sm. Res Social Adm Pharm. 2020	CQ<br>HCQ	69,743 episodes CQ or HCQ +AZM<br><br>72,163 episodes CQ or HCQ + amoxicillin	Risk of cardiac adverse events between combinations of CQ and AZM and CQ and amoxicillin.<br><br>Primary endpoint: sudden cardiac arrest or ventricular arrhythmias (SVC/VA)<br>Secondary endpoint: cardiac symptoms (syncope, tachycardia, or palpitations)	Age (years):<br>18–30: 7,732<br>31–40: 17,101<br>41–50: 31,686<br>51–65: 66,754<br>>65: 18,363<br><br>Gender (male):<br>AZM: 7,921 (11.4) [c]<br>Amoxicillin: 9,467(13.1) [c]	Inclusion criteria:<br>- ≥18 years old<br>- autoimmune disease (on CQ or HQC)<br>- initiation AZM or amoxicillin for ≥5 days<br><br>Exclusion criteria:<br>- HIV<br>- cancer<br>- organ transplant<br>- valvular disorders<br>- cardiomyopathy<br>- pregnancy,<br>- malaria<br>- with any AZM or amoxicillin prescription filled during the six months before the index date	(a)Observational, longitudinal, cohort, retrospective.<br>(b) From amoxicillin or AZM initiation date during CQ use for up to 5 days or until fill of another known QT-prolonging drug.<br>(c) IBM MarketScan Commercial Claims and Medicare Supplemental Databases (it contains detailed information on patient healthcare utilization including medical inpatient and out- patient encounters and pharmacy dispensing	Treatment group (n= 69,743): CQ or HCQ + AZM (no doses mentioned)<br><br>Control group (n=72,163): CQ or HCQ + amoxicillin (no doses mentioned)	(a)<br>a.1 SVC/VA<br><br>AZM group:<br>-1/72529<br>-10 events (95%CI 1-74) /10.000 person-years<br>-HR 1.01 (95%CI 0.06-16.14)<br><br>Amoxicillin group:<br>- 1/75396<br>- 10 events (95%CI 1-74) /10.000 person-years<br>- HR reference<br><br>AZM group adjusted*:<br>- 11 (95%CI 1–77)/10.000 person-years<br>- HR 0.95 (95%CI 0.06–15.17)<br><br>Amoxicillin group adjusted*:<br>- 11 (95%CI 2–76)/10.000 person-years<br>- HR Reference<br><br>a.2 Cardiac symptoms<br><br>AZM group:<br>- 29/69473<br>- 317 (95%CI 221-457)/10.000 person-years<br>HR 1.28 (95%CI 0.74-2.22)<br><br>Amoxicillin group:	86.4 [h]<br>(STROBE)

						claims) from 2005 to 2018		- 23/72163<br>- 249 (95%CI 166-376)/10.000 person-years<br>- HR reference<br><br><u>AZM group adjusted*:</u><br>- 276 (95%CI 185–410) /10.000 person-years<br>- HR 1.10 (95%CI 0.62–1.95)<br><br><u>Amoxicillin group adjusted*:</u><br>- 254 (95%CI 168–383) /10.000 person-years<br>- HR Reference<br><br>*Covariates included cardiac and metabolic conditions, autoimmune disorders, psychiatric conditions, respiratory conditions, infections, variety of other chronic conditions, hospital utilization, smoking, duration of chloroquine/hydroxychloroquine, and using of QTc prolongation drugs (known, possible, or conditional risk).	
Safety signals for QT prolongation or Torsades de Pointes associated with azithromycin with or without chloroquine or hydroxychloroquine. A Sarayani. Res Social Adm Pharm. 2020	HCQ<br>CQ	13.3 million data from the U.S. Food and Drug Administration's Adverse Event Reporting System (FAERS)	To assess the disproportionality in reporting of QT segment prolongation and Torsade de Pointes (TdP) for HCQ and CQ when used alone and in combination	<u>Age (years):</u><br>not reported<br><br><u>Gender (male):</u><br>not reported	<u>Inclusion criteria:</u><br>FAERS report based on the following observed drug mentions:<br>- HCQ/CQ alone<br>- AZM alone<br>- HCQ/CQ + AZM<br>- HCQ/ CQ + amoxicillin<br>- Amoxicillin alone (as control)<br><br><u>Exclusion criteria:</u><br>NA	(a) Observational, cross-sectional<br>(b) NA<br>(c) From the years 1969 through Q3/2019		a)<br>a.1 number of reports, and number of events observed<br>- HCQ/CQ = 78848 reports<br>Death 3412<br>TdP/QT prolongation 344<br>Accidents/Injuries 5488<br>Depression 1341<br><br>- AZM plus HCQ/CQ = 600 reports<br>Death 37<br>TdP/QT prolongation 7<br>Accidents/Injuries 47<br>Depression 24<br><br>- Amoxicillin + HCQ/CQ = 863 reports<br>Death 45<br>TdP/QT prolongation 0	80 [h]<br>(STROBE)

								Accidents/Injuries 66<br>Depression 30<br><br>- AZM: 53378 reports<br>Death 4097<br>TdP/QT prolongation 667<br>Accidents/Injuries 2530<br>Depression 1210<br><br>- Amoxicillin: 103661 reports<br>Death 8809<br>TdP/QT prolongation 521<br>Accidents/Injuries 4340<br>Depression 1806<br><br>a.2 Proportional Reporting Ratios (95% confidence intervals)<br><br>- HCQ/CQ<br>Death 0.46 (0.44-0.47)<br>TdP/QT prolongation 1.43 (1.29-1.59)<br>Accidents/Injuries 1.73 (1.68-1.77)<br>Depression 0.97 (0.92-1.02)<br><br>- AZM plus HCQ/CQ<br>Death 0.66 (0.48-0.90)<br>TdP/QT prolongation 3.77 (1.80-7.87)<br>Accidents/Injuries 1.94 1.47 2.55<br>Depression 2.28 1.54 3.38<br><br>- Amoxicillin plus HCQ/CQ<br>Death 0.56 (0.42-0.74)<br>TdP/QT prolongation NA (NA-NA)<br>Accidents/Injuries 1.89 (1.50-2.38)<br>Depression 1.98 (1.40-2.82)<br><br>- AZM:<br>Death 0.81 (0.79-0.83)<br>TdP/QT prolongation* 4.10 (3.80*- 4.42)<br>Accidents/Injuries 1.18 (1.13-1.22)<br>Depression 1.29 (1.22-1.37)<br><br>- Amoxicillin:	

								Death 0.9 (0.88-0.92)<br>TdP/QT prolongation 1.65 (1.51-1.79)<br>Accidents/Injuries 1.04 (1.01-1.07)<br>Depression 0.99 (0.94-1.04)<br><br>* For pharmacovigilance safety signal detection, the lower limit of the 95% CI is often interpreted as significant if ≥ 2<br><br>- No significant safety signals were observed for HCQ/CQ when used alone. However, when AZM was used alone, it was associated with a significant safety signal related to TdP/QT prolongation. With HCQ/CQ plus AZM, results did not reach the threshold to indicate a significant safety signal.	

Abbreviations: AEs: adverse events; ALT: alanine aminotransferase; AP: atovaquone and proguanil; AST: asparate aminotransferase; AZM: azithromycin; CK: creatine phosphokinase; CKMB: creatine kinase-MB; CQ: chloroquine; g: grams; G6PD: glucose-6-phosphate dehydrogenase; hCG: human chorionic gonadotropin; HCQ: hydroxychloroquine; IPTp: intermittent preventive treatment during pregnancy; mg: milligrams; mg/dl: milligrams/deciliter; MQ: chloroquine and mefloquine hydrochloride; ms: milliseconds; NA: not available/not applicable; QTcF: QTc interval corrected by the Fridericia method; SP: sulfadoxine-pyrimethamine; SPCQ: sulfadoxine-pyrimethamine-chloroquine ; µL: microliter.

[a]Jadad, AR et al. Assessing the quality of reports of randomized clinical trials: is blinding necessary? Control Clin Trials. 1996; 17(1):1–12.
Values are: [b]mean (SD); [c] n (%); [d]mean (range); [e]median (interquartile range); [f]median (range); [g]mean (SD) {range}.
[h]Quality assessment, a higher score indicates a higher quality: [Yes (1) + Partly (0.5)/Total applicable] x100.

Table S21. Characteristics of included studies: case reports and case series in patients with COVID-19

Study ID: title, first author, journal of publication, year	Drug	Indication	Adverse reaction reported	Adverse effect management<br>(a) Discontinuation<br>(b) Management<br>(c) Reintroduction<br>(d) Long-term consequences<br>(e) Follow up	Population characteristics:<br>(a) Age (years)<br>(b) Gender<br>(c) Clinical characteristics	Other:<br>(a) Time of appearance<br>(b) Cumulative dose<br>(c) Regimen	Adverse Drug Reaction Probability Scale:<br>(a) Scale<br>(b) Score (probability category)	Quality assess-ment[a] (%)
Case report: right bundle brunch block and QTc prolongation in a patient with novel coronavirus disease (COVID-19) treated with hydroxychloroquine. Asli R. Am J Trop Med Hyg. 2020	HCQ	COVID-19	Right bundle brunch block and critically prolonge d QTc	(a) Yes<br>(b) Blood investigations showed slightly low corrected Ca^{2+} which was corrected with calcium replacement<br>(c) No<br>(d) No<br>(e) ECG performed 24 h after the last dose of HCQ showed normalization of the QTc	(a) 60<br>(b) Female<br>(c) Hypertension, hyperlipidemia and being overweight but without any cardiac history	(a) 3 days<br>(b) 1.4 g<br>(c) 0.4 g start dose followed by 0.2 g BD	(a) NA<br>(b) NA	87
COVID-19 infection and treatment with hydroxychloroquine cause severe haemolysis crisis in a patient with glucose-6-phosphate dehydrogenase deficiency. Beauverd Y. Eur J Haematol. 2020	HCQ	COVID-19	Worsenin g of haemolysi s	(a) Yes<br>(b) Several blood transfusions<br>(c) NA<br>(d) NA<br>(e) NA	(a) 68<br>(b) Male<br>(c) Type 2 diabetes mellitus, high blood pressure, chronic renal insufficiency and previous history of stroke	(a) 1 day<br>(b) 0.6 g<br>(c) 0.6 g single dose	(a) NA<br>(b) The authors suggested that severe COVID-19 infection triggered haemolysis in a patient with G6PD deficiency. Moreover, haemolysis was possibly worsened by HCQ administration	58
G6PD deficiency-associated hemolysis and methemoglobinemia in a COVID-19 patient treated with chloroquine. Kuipers MT. Am J Hematol. 2020	CQ	COVID-19	G6PD deficiency - associate d hemolysis and methemo globinemi a	(a) Yes<br>(b) Three units of packed red blood cells and ascorbic acid 1 g IV 4 times a day for 2 days<br>(c) NA<br>(d) No<br>(e) The patient's methaemoglobin normalized within 6 days and laboratory testing for G6PD deficiency confirmed very low G6PD activity in the patient's red blood cells. Genetic analysis demonstrated variant G6PD A-	(a) 56<br>(b) Male<br>(c) Diabetes mellitus type 2	(a) Less than 5 days<br>(b) NA<br>(c) 0.6 g followed by 0.3 g BD (for 5 days)	(a) NA<br>(b) NA	67
A case of exacerbation of psoriasis after oseltamivir and hydroxychloroquine in a patient with COVID-19: Will cases of psoriasis increase	HCQ	COVID-19	Psoriasis exacerbat ion	(a) NA<br>(b) NA<br>(c) NA<br>(d) NA<br>(e) NA	(a) 71<br>(b) Female<br>(c) NA	(a) 4 days<br>(b) NA<br>(c) 2 × 0.4 g on the first day then 2 × 0.2 g	(a) NA<br>(b) NA	46

after COVID-19 pandemic? Kutlu Ö. Dermatol Ther. 2020								
An algorithm for managing QT prolongation in coronavirus disease 2019 (COVID 2019) patients treated with either chloroquine or hydroxychloroquine in conjunction with azithromycin: possible benefits of intravenous lidocaine. Mitra RL. Heart Rhythm Case Reports. 2020	HCQ	COVID-19	QT interval prolongation in a patient on azithromycin	(a) Yes<br>(b) Lidocaine 100 mg IV<br>(c) Yes<br>(d) Shortening of the QTc. With lidocaine IV infusion, the patient was able to complete the course of HCQ without an arrhythmic event<br>(e) Death owing to progressive metabolic acidosis and multiorgan system failure	(a) 66<br>(b) Female<br>(c) Rheumatoid arthritis, pulmonary fibrosis and asthma	(a) After the first dose of HCQ<br>(b) NA<br>(c) One dose	(a) NA<br>(b) NA	73
Acute generalized exanthematous pustulosis with erythema multiforme-like lesions in a COVID-19 woman. Robustelli Test E. Eur Acad Dermatol Venereol. 2020	HCQ	COVID-19	AGEP with erythema multiforme-like lesions	(a) No<br>(b) Oral prednisone 0.3 mg/kg daily and gradually taper prednisone within 30 days<br>(c) No<br>(d) No<br>(e) Slow but progressive resolution	(a) 70<br>(b) Female<br>(c) No personal/family history of psoriasis	(a) 13 days<br>(b) 4 g<br>(c) 0.2 g BD for 10 days	(a) NA<br>(b) Although the authors cannot rule out that lopinavir/ritonavir could be implicated into the rash, they suggest that HCQ was the probable triggering drug	76
Chloroquine-induced torsade de pointes in a patient with coronavirus disease 2019. Szekely Y. Heart Rhythm. 2020	CQ diphosphate	COVID-19	Major QT prolongation and recurrent torsade de pointes	(a) Yes<br>(b) Other known or suspected QT-prolonging medications were discontinued as well as nonessential medications were withheld, including memantine and letrozole. Bisoprolol was also discontinued owing to bradycardia. The patient was placed under continuous ECG monitor and was given additional oral potassium supplements. The patient was treated with magnesium sulphate 2 g IV, potassium 3 g IV, lidocaine 100 mg IV and isoproterenol IV (18 h infusion)<br>(c) NA<br>(d) No<br>(e) Immediate resolution of all ventricular arrhythmias with no apparent clinical change. Subsequent ECGs showed gradual normalization of her QT interval	(a) 84<br>(b) Female<br>(c) Metastatic breast cancer, a remote history of pulmonary embolism, arterial hypertension and evidence of left ventricular hypertrophy but is otherwise free of heart disease. Her medications include bisoprolol, letrozole, memantine and apixaban	(a) 5 days<br>(b) 5 g<br>(c) 0.5 g BD for 5 days	(a) NA<br>(b) Memantine is a drug rated as a medication with a “possible risk of torsade de pointes” and likely contributed to the proarrhythmic effects of CQ. Yet, the QTc interval increased after CQ was initiated, clearly pointing to CQ as the culprit drug	80

Hydroxychloroquine-induced creatine kinase elevation. Bayrakçı S. Flora. 2020	HCQ	Dry cough, high fever, diarrhea and general condition disorder compatible with COVID-19	Creatine kinase, creatine kinase MB and creatinine elevation	(a) Yes<br>(b) Blood investigations<br>(c) NA<br>(d) No<br>(e) Creatine kinase levels decreased 72 h after cessation. During the treatment, liver functions were normal and kidney functions returned to normal	(a) 80<br>(b) Male<br>(c) Dementia, hypertension, and previous cerebrovascular accident	(a) After the second dose of HCQ<br>(b) 0.8 g<br>(c) 0.4 g BD on the first day then 0.2 g BD	(a) Naranjo[b]<br>(b) 3 (possible)	78
Wide complex tachycardia in a patient with COVID-19 treated with chloroquine/azithromycin. Gracia-Ramos AE. Oxf Med Case Reports. 2021	CQ plus AZM	COVID-19	WCT	(a) NA<br>(b) Amiodarone 0.3 g IV<br>(c) NA<br>(d) The rhythm following cardioversion 5 min after starting amiodarone infusion was visualized with a DII rhythm strip, which showed an irregular rhythm, QRS 110 ms at 100 beats/min on average with biphasic T waves with ST-segment depression, without organized atrial activity, suggesting atrial fibrillation. There was no evidence of prolonged QT. A measurement of troponin I was obtained during the arrhythmia episode, which was 0.2 ng/dl (NR 0.0– 0.6 ng/dl). Laboratory tests obtained on the same day (prior to the arrhythmia episode) were not relevant to explain the event. A Cardiology Service consultation was requested; unfortunately, the patient presented a cardiac arrest (50 min after pharmacological cardioversion) without response to cardiopulmonary resuscitation and died<br>(e) Death after cardiac arrest	(a) 43<br>(b) Male<br>(c) 24-year history of hypertension and chronic renal disease due to non-specific glomerulonephritis, receiving a kidney transplant from a cadaveric donor 6 months ago and surgical parathyroidectomy due to tertiary hyperparathyroidism. His medications were nifedipine 30 mg twice daily, metoprolol 100 mg twice daily, mycophenolic acid 500 mg twice daily, tacrolimus 1 mg twice daily, prednisone 25 mg/day, calcium carbonate 4 g/day and calcitriol 0.25 mcg/day	(a) 4 days<br>(b) NA<br>(c) CQ: 0.5 g BD on the first day then 0.5 g QD on days 2-5.<br><br>AZM: 0.5 g QD on the first day then 0.25 g QD on days 2–5	(a) NA<br>(b) Although the authors were unable to get a 12 lead ECG, they considered that the WCT was very likely a monomorphic VT based on the analysis of the DII rhythm strip. They hypothesize that such condition was possibly related to the combined CQ/AZM therapy along with the previously described associated medical conditions. It is possible that the simultaneous use of AZM and tacrolimus (another medication with known capacity to increase QTc) had had a synergistic cardiotoxic effect. Another factor to consider is a possible myocarditis. Therefore, the authors speculate that the presence of SARS-CoV-2 in the cardiomyocyte could result in alteration of signaling pathways that, together with proarrhythmogenic drugs, generated the	88

							arrythmia in this patient	
Torsades de pointes in SARS-CoV-2 (COVID-19) pneumonia: medicine reconciliation and careful monitoring of QTc interval may help prevent cardiac complications. Aslam W. BMJ Case Rep. 2021	HCQ	COVID-19	QTc prolongation and torsade de pointes	(a) Yes<br>(b) NA<br>(c) NA<br>(d) NA<br>(e) QTc improved after discontinuation of HCQ and other QT-prolonging medications. The patient's intensive care unit course was complicated by prolonged respiratory failure, methicillin-resistant Staphylococcus aureus pneumonia, metabolic encephalopathy, right lower extremity deep venous thrombus and upper gastrointestinal bleed. The patient did not have any recurrence of torsade de pointes or any other ventricular arrhythmia. Unfortunately, the patient was transitioned to comfort care on day 18 of the intensive care unit stay after a failed extubation	(a) 73<br>(b) Female<br>(c) Diabetes mellitus type II, hyperlipidaemia, dementia, depression and gastro-oesophageal reflux disease.<br>Her medications included citalopram 20 mg daily, donepezil 10 mg daily and gabapentin 300 mg three times per day	(a) 3 days<br>(b) 1.6 g<br>(c) 0.8 g for 1 day followed by 0.4 g for 4 days	(a) NA<br>(b) The authors reported the patient had QTc prolongation and torsade de pointes provoked by multiple QT-prolonging medications including citalopram, donepezil, propofol and HCQ	74
Psychiatric side effects of chloroquine in COVID-19 patients: two case reports. Benjelloun R. Pan Afr Med J. 2020	CQ plus AZM	COVID-19	Psychotic symptoms	(a) Yes<br>(b) Cerebral CT scan, laboratory tests and serologies were performed. The COVID-19 medication protocol and vortioxetine were interrupted, and amilsulpride 100 mg per day was initiated.<br>(c) NA<br>(d) NA<br>(e) Psychotic symptoms disappeared totally after 48 h. Amilsulpride was tapered off and then stopped within a week. The patient remained asymptomatic	(a) 46<br>(b) Male<br>(c) Mild depressive episode treated with vortioxetine since November 2019	(a) 9 days<br>(b) NA<br>(c) NA	(a) Naranjo[b]<br>(b) 7 (probable)	72
	CQ plus AZM	COVID-19	Acute and intense anxiety	(a) Yes<br>(b) Lorazepam 2.5 mg spread over 24 h showed moderate improvement of anxiety symptoms. Five days after the AZM-CQ initiation, and two days after the onset of anxiety symptoms, the patient interrupted AZM-CQ association on her own initiative.<br>(c) No<br>(d) No<br>(e) Anxiety symptoms disappeared rapidly. The physician indicated to switch to HCQ	(a) 35<br>(b) Female<br>(c) No personal or family history of mental illness	(a) 3 days<br>(b) NA<br>(c) NA	(a) Naranjo[b]<br>(b) 7 (probable)	

				associated with AZM. The patient went under this treatment for six days, and remained asymptomatic. After the patient left hospital, the physician followed her up by phone. The patient did not express any distress, anxiety or other psychiatric symptom				
Hydroxychloroquine-related rash in COVID-19 infected patient. Kurd R. Isr Med Assoc J. 2020	HCQ sulfate	COVID-19	Rash	(a) NA<br>(b) NA<br>(c) NA<br>(d) NA<br>(e) NA	(a) 77<br>(b) Male<br>(c) Ischemic heart disease	(a) 12 days<br>(b) NA<br>(c) NA	(a) NA<br>(b) The rash was attributed to HCQ treatment as no other offending agent was acknowledged	44
Chloroquine-Induced First-Episode Psychosis in a Patient Self-medicated for COVID-19. Ambar M. Biol Psychiatry. 2021	CQ	COVID-19	Psychosis	(a) Yes, at 7th day of CQ treatment<br>(b) The patient was hospitalized in a psychiatric department. Sedation with loxapine 100 mg and clonazepam 1 mg was administered with a mechanical contention<br>(c) No<br>(d) NA<br>(e) NA	(a) 57<br>(b) Female<br>(c) Subsyndromal anxiety treated with escitalopram 10 mg/day for several years, and no other personal or family medical history	(a) 2 days<br>(b) NA<br>(c) Self-medicated with CQ for 7 days to prevent it	(a) Naranjo[b]<br>(b) 7 (probable)	64
Acute generalized exanthematous pustulosis induced by empiric hydroxychloroquine for presumed COVID-19. Enos T. Dermatol Ther. 2020	HCQ	COVID-19	AGEP	(a) Yes<br>(b) Six-day oral methylprednisolone taper<br>(c) No<br>(d) Twelve days after she began HCQ, the patient presented at an outside hospital, where she was given intravenous methylprednisolone 125 mg. The patient was admitted and treated conservatively with topical triamcinolone 0.1% ointment but experienced significant pruritus and additional pustule formation, necessitating one administration of IV methylprednisolone 500 mg<br>(e) The rash resolved with the prednisone taper after 38 days	(a) 29<br>(b) Female<br>(c) Protein S deficiency and Stevens-Johnson syndrome due to cefaclor	(a) 4 days<br>(b) 1.6 g<br>(c) 0.2 g BD	(a) NA<br>(b) NA	83
Acute generalized exanthematous pustulosis induced by hydroxychloroquine prescribed for COVID-19. Delaleu J. J Allergy Clin Immunol Pract. 2020	HCQ	COVID-19	AGEP	(a) Yes<br>(b) NA<br>(c) NA<br>(d) The patient eventually died from massive pulmonary embolism 10 days after the AGEP diagnosis<br>(e) NA	(a) 76<br>(b) Male<br>(c) Diabetes mellitus	(a) 9 days<br>(b) 3.6 g<br>(c) 0.2 g TD	(a) RegiSCAR<br>(b) 11 (definite case)	88

Hydroxychloroquine-induced erythema multiforme in a patient with COVID-19. Monte J. Med Clin (Barc). 2020	HCQ	COVID-19	Erythema multiform e	(a) Yes<br>(b) NA<br>(c) No<br>(d) NA<br>(e) NA	(a) 55<br>(b) Female<br>(c) NA	(a) 12 days<br>(b) NA<br>(c) NA	(a) NA<br>(b) NA	43
A case report of serious haemolysis in a glucose-6-phosphate dehydrogenase-deficient COVID-19 patient receiving hydroxychloroquine. Maillart E. Infect Dis (Lond). 2020	HCQ	COVID-19	Haemoly sis in a G6DP-deficient patient	(a) Yes<br>(b) Multiple blood transfusions<br>(c) NA<br>(d) NA<br>(e) HCQ level was still at 93 mg/L 30 days after its administration	(a) 65<br>(b) Male<br>(c) Hypertension and type 2 diabetes. There was no history of drug-induced haemolysis	(a) 1 day<br>(b) NA<br>(c) HCQ and AZM were started on the admission day	(a) NA<br>(b) NA	85
Hemolytic anemia in a glucose-6-phosphate dehydrogenase-deficient patient receiving hydroxychloroquine for COVID-19: A case report. Aguilar J. Perm J. 2020	HCQ	COVID-19	Haemoliti c anemia in a G6DP-deficient patient	(a) NA<br>(b) Red blood cell transfusions<br>(c) NA<br>(d) Outpatient hemodialysis scheduled<br>(e) NA	(a) 51<br>(b) Male<br>(c) Type-2 diabetes, hypertension, and morbid obesity	(a) 6 days<br>(b) 2 g<br>(c) 0.4 g BD on day 1, and 0.4 g QD on days 2-4	(a) NA<br>(b) NA	88
COVID-19 & Hydroxychloroquine side-effects: Glucose 6-phosphate dehydrogenase deficiency (G6PD) and acute haemolytic anaemia. Chaney Sl. QJM 2020	HCQ	COVID-19	Haemolys is a G6DP-deficient patient	(a) NA<br>(b) Two units of RCC<br>(c) NA<br>(d) Test<br>(e) NA	(a) 57<br>(b) Male<br>(c) Non-insulin dependent diabetes, hypertension, hypercholesterolaemia, gastro-oesophageal reflux disease and glaucoma. He had mild anaemia on admission, but no known haematology history	(a) 7 days<br>(b) NA<br>(c) NA	(a) NA<br>(b) NA	57
Suspected hydroxychloroquine-induced sinus bradycardia and QTc prolongation in a patient with COVID-19. Kang Y. Int Heart J. 2020	HCQ	COVID-19	Suspecte d-HCQ induced sinus bradycard ia and QTc interval prolongat ion	(a) Yes<br>(b) Isoproterenol intravenously titrated to a maximal dose of 10 mcg/minute, epinephrine at a maximal dose of 1 mcg/kg/minute. She was incubated and rescued by defibrillation.<br>(c) NA<br>(d) A temporary pacemaker was implanted<br>(e) Such an abnormality disappeared days after HCQ withdrawal	(a) 84<br>(b) Female<br>(c) Hypertension for 10 years, Alzheimer's disease for five years, and stage 4 chronic kidney disease diagnosed two months ago	(a) 5 days<br>(b) 2 g<br>(c) 0.2 g BD	(a) NA<br>(b) NA	63
Case report: hepatotoxicity associated with the use of hydroxychloroquine in a	HCQ	COVID-19	Hepatoto xicity	(a) Yes<br>(b) Discontinuation<br>(c) No<br>(d) NA	(a) 29<br>(b) Female<br>(c) NA	(a) 2 days<br>(b) 0.8g<br>(c) 0.2 g BD	(a) NA<br>(b) NA	90

patient with COVID-19. Falcão MB. Am J Trop Med Hyg. 2020				(e) NA				
Cutaneous side effects of hydroxychloroquine in health care workers in a COVID referral hospital - implications for clinical practice. Sardana K. J Dermatolog Treat. 2020	HCQ	COVID-19 prophylaxis	Urticaria with maculopapular rash, Palmoplantar itching	(a) Yes<br>(b) Discontinuation, antihistamine plus methylprednisolone.<br>(c) No<br>(d) NA<br>(e) NA	(a)26<br>(b)Female<br>(c)NA	(a) 2<br>(b) 0.8g<br>(c) 0.4g BD on day one followed by 0.4g weekly for 7 weeks.	(a) Naranjo<br>(b) 5	90
		COVID-19 prophylaxis	Urticaria	(a) Yes<br>(b) Discontinuation, antihistamine.<br>(c) No<br>(d) NA<br>(e) NA	(a)37<br>(b)Female<br>(c)NA	(a)7<br>(b) 0.8g<br>(c) 0.4g BD on day one followed by 0.4g weekly for 7 weeks.		
		COVID-19 prophylaxis	Palmplantar itching	(a) Yes<br>(b) Discontinuation, antihistamine.<br>(c) No<br>(d) NA<br>(e) NA	(a)22<br>(b)Female<br>(c)NA	(a) 7<br>(b) 0.8g<br>(c) 0.4g BD on day one followed by 0.4g weekly for 7 weeks.		
First case of DRESS syndrome caused by hydroxychloroquine with a positive patch test.Castro Jiménez A. Contact Dermatitis. 2021	HCQ plus AZM plus LPVr	Bilateral pneumonia, suspected COVID-19	DRESS	(a) NA<br>(b) NA<br>(c) NA<br>(d) NA<br>(e)NA	(a) 37<br>(b) Female<br>(c) NA	(a) NA<br>(b) NA<br>(c) HCQ 0.2g QD, LPVr 200/50mg BD and AZM 250mg QD for 5 days.	(a) NA<br>(b) NA	90
Generalized pustular figurate erythema first report in two COVID-19 patients on hydroxychloroquine. Abadías-Granado I. J Eur Acad Dermatol Venereol. 2021	HCQ	COVID-19	Purpuric erythematous rash with non-follicular pustules, on the trunk and limbs, with intense involvement of armpits and scalp	(a) No<br>(b) He received 0.05% betamethasone dipropionate cream twice a day, loratadine (10 mg/days) and methylprednisolone (40 mg/days), with slow resolution over 4 weeks.<br>(c) No<br>(d) No<br>(e) Yes	(a) 64<br>(b) Male<br>(c) Diffuse large B-cell lymphoma	(a) Two to three weeks after starting these medications<br>(b) 4.4 g<br>(c) 400 mg BD first day followed by 200 mg BD for 10 days	(a) NA<br>(b) NA	76

			Purpuric erythematous rash with non-follicular pustules and targetoid lesions on the back	(a) No<br>(b) She received 0.05% betamethasone dipropionate cream twice a day, loratadine (10 mg/days) and methylprednisolone (40 mg/days), with slow resolution over 4 weeks.<br>(c) No<br>(d) No<br>(e) Yes	(a) 60<br>(b) Female<br>(c) Rheumatoid arthritis	(a) Two to three weeks after starting these medications<br>(b) 4.4 g<br>(c) 400 mg BD first day followed by 200 mg<br>BD for 10 days	(a) NA<br>(b) NA	76
Myasthenic Syndrome Caused by Hydroxychloroquine Used for COVID-19 Prophylaxis. Koc G. J Clin Neuromuscul Dis. 2020	HCQ	COVID-19 Prophylaxis	Ocular Myasthenic syndrome	(a) Yes<br>(b) Pyridostigmine treatment with 240 mg/daily dose was begun as symptomatic treatment, and the patient's complaints were dramatically decreased<br>(c) Yes<br>(d) No<br>(e) After 3 weeks, patient's complaints were disappeared; also, the control SFEMG and neurological examination were normal. Symptomatic treatment was ceased	(a) 47<br>(b) Male<br>(c) NA	(a) 1 day<br>(b) 200 mg<br>(c) 200 mg	(a) NA<br>(b) NA	63
Cardiovascular Considerations of Experimental Hydroxychloroquine Therapy on Patients Diagnosed With COVID-19: A Case Series Review. Patel J. Cureus. 2020	HCQ plus AZM	COVID-19	QTc prologantion (from 446 ms to 650 ms)	(a) Yes (as well as ondansetrón and AZM<br>(b) Discontinuation of drugs, and her electrolytes were optimized, specifically with Mg of >2.0 mEq/L and K of >4.0 mEq/L.<br>(c) No<br>(d) No<br>(e) On day nine, her QTc was noted to be 458 ms on. She made an uneventful recovery and was discharged on hospital day 10.	(a) 74<br>(b) Female<br>(c) Coronary artery disease, chronic obstructive pulmonary disease, and autoimmune hepatitis	(a) 3 day<br>(b) 2.4 g<br>(c) HCQ 400 mg BD on day one followed by 200 mg BD on days two to five	(a) NA<br>(b) NA	72
	HCQ plus AZM plus steroids		nausea, vomiting, diarrhea and sinus bradycardia	(a) Yes<br>(b) Intravenous fluids.<br>(c) No<br>(d) No<br>(e) NA	(a) 40<br>(b) Female<br>(c) No significant medical history	(a) 48 h<br>(b) 2.4 g<br>(c) HCQ 400 mg BD on day one followed by 200 mg BD on days two to five	(a) NA<br>(b) NA	72
Hydroxychloroquine-Associated Thrombotic Thrombocytopenic Purpura.	HCQ	Suspected COVID-19 with negative	Thrombotic thromboc	(a) Yes<br>(b) Plasmapheresis with 1.5 volumes was started. Methylprednisolone (1	(a) 65<br>(b) Male<br>(c) chronic obstructive pulmonary disease	(a) 3 days<br>(b) NA<br>(c) NA	(a) Naranjo scale<br>(b) 4, possible	67

Arıkan F. Turk J Haematol. 2020		SARS-CoV-2 PCR test	ytopenic purpura	mg/kg/day) and folic acid supplementation was commenced.<br>(c) No<br>(d) No<br>(e) NA				

Abbreviations: AGEP: Acute generalized exanthematous pustulosis; AZM: azithromycin; BD: twice a day; COVID-19: coronavirus disease 2019; CQ: Chloroquine; DEX: Dexamethasone; DRESS: Drug rash with eosinophilia and systemic symptoms; ECGs: electrocardiograph; g: grams; G6PD: glucose-6-phospate dehydrogenase; h: hours; HCQ: Hydroxychloroquine; IV: intravenous; kg: kilograms; LPVr: lopinavir/ritonavir; mcg: micrograms; mg: milligrams; min: minutes; ms: millisecond; NA: not available/not applicable; ng/dl: nanograms per deciliter; NR: normal range; QD: once a day; QTc: corrected QT interval; TD: three times a day; VT: ventricular tachycardia; WCT: wide complex tachycardia.

[a]Quality assessment: a higher score indicates a higher quality. (%) [Yes (1) + Partly (0.5)/Total applicable] x100

[b]Naranjo, C.A.; Busto, U.; Sellers, E.M.; Sandor, P.; Ruiz, I.; Robert, E.A.; Janecek, E.; Domecq, C.; Greenblatt, D.J. A method for estimating the probability of adverse drug reaction. Clin. Pharmacol. Ther. 1981, 30, 239–245.

Table S22. Characteristics of included studies: observational studies and clinical trials (COVID-19 treatment and prophylaxis)

Study ID: title, first author, journal of publication, year.	Drug (CQ/ HCQ) plus other drugs to treat COVID-19	Sample Size	Aim	Age and Gender	Patient Inclusion and Exclusion Criteria	Main study characteristics: (a) Type of study (b) Epidemiological approach (c) Data gathering (d) Time Horizon (e) Time Frame	Regimens	Adverse event characteristics: (a) Measure	Quality score (%)[a]
Assessment of QT Intervals in a case series of patients with coronavirus disease 2019 (COVID-19) infection treated with hydroxychloroquine alone or in combination with azithromycin in an intensive care unit. Bessière F. JAMA Cardiol. 2020	HCQ or HCQ + AZM	40<br><br>Groups:<br>HCQ: 22<br><br>HCQ + AZ: 18	To assess the safety of HCQ with or without AZM regarding QT interval in ICU patients with COVID-19	Age (years):<br>68 (58-74)[e]<br><br>Gender (male):<br>32 (80)[c]	Inclusion criteria:<br>- Admitted to the ICU<br>- PCR confirmed COVID-19 illness<br>- Received HCQ ± AZM<br><br>Exclusion criteria:<br>-Treatment contraindication, including QTc >460 ms	(a) Observational<br>(b) Longitudinal<br>(c) Retrospective<br>(d) ICU hospitalization<br>(e) 15th to 29th March 2020	Treatment group:<br>HCQ 200 mg BD for 10 days ± AZM 250 mg QD for 5 days<br><br>Control group:<br>NA	a) QTc prolonged ≥500 ms:<br>HCQ: 1 (5)[c]<br>HCQ + AZM: 6 (33)[c]<br>p = 0.03<br>QTc change ≥60 ms:<br>HCQ ± AZM: 10 (25)[c]<br>TdP:<br>Not observed	53.3 (Strobe)
Conduction abnormalities in hydroxychloroquine add on therapy to lopinavir/ritonavir in COVID-19. Chong VH. J Med Virol. 2020	HCQ + LPVr	11	To add on experience with use of HCQ as add on therapy for COVID-19 disease	Age (years):<br>51.5 (27-67)[d]<br><br>Gender (male):<br>63.6%	Inclusion criteria:<br>- RT-PCR confirmed COVID/19<br>- Moderate or severe case which means any of the following:<br>- Chest radiograph changes<br>- > 60 years old<br>- Comorbidities (diabetes, immunocompromised, pulmonary or cardiovascular disease)<br>- Persistent fever and/or diarrhoea<br>- Lymphopenia	(a) Observational<br>(b) Longitudinal<br>(c) Retrospective<br>(d) NA<br>(e) NA	Treatment group:<br>LPVr 400/100 mg BD at least 14 days<br><br>HCQ 400 mg BD day 1 (loading dose) and 200 mg BD for 4 days<br><br>Control group:<br>NA	(a) Discontinuation due to ECG modifications:<br>4 (36.4)[c]<br>QTc prolonged ≥500ms:<br>2 (18.2)[c]<br>(a third patient had a QTc prolongued ≥500 ms but was also prolonged at baseline)<br>Discontinuation due to non-cardiac AEs:<br>1(9)[c]<br>Other (n):<br>Prolonged QTc<500 ms (2), right bundle brunch block (1), bradycardia (1) and thrombocytopenia (1)	64.7 (Strobe)

					- Neutrophil to lymphocyte ratio greater than or equal to 3.1<br>- National Early Warning Score (≥5)<br><br>Exclusion criteria:<br>NA				
QT Interval prolongation and torsade de pointes in patients with COVID-19 treated with hydroxychloroquine/azithromycin. Chorin E. Heart Rhythm. 2020	HCQ + AZM	251	To assess the change in QTc interval and arrhythmic events in patients with COVID-19 treated with HCQ and AZM	Age (years):<br>64 (13)[b]<br><br>Gender (male):<br>188 (75)[c]	Inclusion criteria:<br>- Combination of HCQ and AZM<br>- A baseline ECG and at least one ECG performed after drug administration<br><br>Exclusion criteria:<br>- Patients without baseline ECG	(a) Observational<br>(b) Longitudinal<br>(c) Retrospective<br>(d) From the initiation of treatment to a maximal follow up of 8 days<br>(e) The closing date of follow-up was 15th April 2020 (no mention of when was started)	Treatment group:<br>HCQ 400 mg BD for 1 day (loading dose) followed by 200 mg BD for 4 days. AZM orally 500 mg QD for 5 days<br><br>Control group:<br>NA	(a) ΔQTc (ms), baseline to maximum:<br>34 (35)[b]<br>ΔJTc (ms), baseline to maximum:<br>33 (36)[b]<br>QTc prolonged ≥500 ms (patients with QRS <120):<br>28 out of 222 (13)[c]<br>JTc prolonged ≥410 ms (patients with QRS >120):<br>4 out of 29 (14)[c]<br>Extreme QTc prolongation (QTc prolonged ≥500 ms, QTc change ≥60 ms or JTc change ≥410 ms):<br>58 (23)[c]<br>TdP:<br>1 (0.4)[c]	69 (Strobe)
Arrhythmic profile and 24-hour QT interval variability in COVID-19 patients treated with hydroxychloroquine and azithromycin. Cipriani A. Int J Cardiology. 2020	HCQ + AZM	56<br><br>Groups:<br>Treatment group: 22<br><br>Control group: 34	To assess ECG changes, arrhythmic profile and 24h QT interval variations in non-critically ill patients affected by COVID-19	Age (years):<br>Cases: 64 (56-70)[e]<br><br>Gender (male):<br>Cases: 18 (82)[c]	Inclusion criteria:<br>- Diagnosis of COVID-19<br>- Oral treatment with HCQ (200 mg BD) and AZM (500 mg QD) for at least 3 days<br><br>Exclusion criteria:<br>- Age > 80 years old<br>- Known coronary artery disease or inherited arrhythmic conditions (including channellopathies)<br>- Familial sudden cardiac death<br>- Drug allergy<br>- Severe electrolyte imbalance (defined as a serum potassium levels of <3.5 mEq/l or >5.0 mEq/l; serum sodium levels <130 mEq/l or >150 mEq/l; serum calcium levels of <8.0 mg/dl or >10.0 mg/dl)	(a) Observational (case-control)<br>(b) Longitudinal<br>(c) Prospective<br>(d) At the beginning of therapy and at least 3 days after<br>(e) March 2020	Treatment group:<br>HCQ (200 mg BD) and AZM (500 mg QD) for at least 3 days<br><br>Control group:<br>No treatment, healthy individuals matched for age and sex	(a) Cases:<br>Syncope, fatal arrhythmias and sudden cardiac death:<br>Not observed<br>ΔQTc (ms, mean):<br>24<br>QTc prolonged ≥500 ms:<br>1 (4.54)[c]<br>QTc prolonged ≥480 ms:<br>4 (18)[c]<br><br>Cases vs controls:<br>Non-sustained ventricular tachycardia:<br>Cases: 1 (4.54)[c]<br>Controls: Not observed<br>p = 0.4<br>"R on the T" premature ventricular beats:<br>Cases: Not observed<br>Controls: Not observed<br>p = NA<br>≥ 1 Premature atrial beats:<br>Cases: 18 (82)[c]<br>Controls: 15 (46)[c]<br>p <0.01	79.5 (Strobe)

					- Concomitant treatment with antiarrhythmic drugs or all other QT interval–prolonging medications			≥ 1 Premature ventricular beats:<br>Cases: 15 (68)[c]<br>Controls: 12 (36)[c]<br>p = 0.02<br>≥ 1 Supraventricular tachycardia:<br>Cases: 5 (23)[c]<br>Controls: 3 (9)[c]<br>p = 0.24	
Tocilizumab for treatment of severe COVID-19 patients: preliminary results from SMAtteo COvid19 REgistry (SMACORE). Colaneri M. Microorganisms. 2020	HCQ + AZM + LWH + MPDN ± TCZ	112<br><br>Groups:<br>TCZ + SOC: 21	To assess the role of TCZ therapy in terms of ICU admission and mortality rate of critically ill patients with severe COVID-19 pneumonia	Age (years): 63.55 (16.95)[e]<br><br>Gender (male): 82 (73.2)[c]	Inclusion criteria:<br>- Patients with confirmed diagnosis of COVID-19 disease<br>- SMACORE cohort: patients who were administered TCZ and patients who were treated according to SOC<br><br>Exclusion criteria:<br>NA	(a) Observational<br>(b) Longitudinal<br>(c) Retrospective (analysis of two groups of the SMACORE cohort)<br>(d) Not mentioned<br>(e) 14th to 27th March 2020	Treatment group:<br>TCZ: 8 mg/kg (up to a maximum 800 mg per dose) SOC: a combination of HCQ (200 mg BD), AZM (500 mg QD), prophylactic dose of LWH and MDPN (a tapered dose of 1 mg/kg up to a maximum of 80 mg) for 10 days<br><br>Control group:<br>SOC: a combination of HCQ (200 mg BD), AZM (500 mg QD), prophylactic dose of LWH and MPDN (a tapered dose of 1 mg/kg up to a maximum of 80 mg) for 10 days	(a) Any type of AEs:<br>No AEs were detected following TCZ administration. No other side effects were mentioned	76 (Strobe)
COVID-19 in solid organ transplant recipients: A single-center case series from Spain. Fernández-Ruiz M. Am J Transplant. 2020	HCQ ± LPVr ± IVIG ± TCZ ± INF-β	18<br><br>Groups:<br>HCQ: 4<br><br>LPVr: 1<br><br>HCQ + LPVr: 6<br><br>HCQ +IVIG: 1<br><br>HCQ +TCZ: 1	To report preliminary experience with 18 solid organ transplant recipients diagnosed with COVID-19	Age (years): 71.0 (12.8)[b]<br><br>Gender (male): 14 (77.8)[c]	Inclusion criteria:<br>- Diagnosed with COVID-19<br>- ≥ 18 years old<br>- Solid organ transplantation recipients<br><br>Exclusion criteria:<br>NA	(a) Observational<br>(b) Longitudinal<br>(c) Retrospective<br>(d) Enrolled at the time of diagnosis of COVID-19 and followed up to 4th April 2020<br>(e) 5th to 23th March 2020	Treatment groups:<br>LPVr: 200/100 mg BD for 14 days<br><br>HCQ: 400 mg BD (loading dose) followed by 200 mg BD for 5-10 days<br><br>INF-β: 250 µg every 48 h<br><br>TCZ: 600 mg single dose<br><br>IVIG: not reported	a) Discontinuation due to ECG modifications:<br>Not observed<br>Discontinuation due to non-cardiac AEs:<br>LPVr + HCQ: 1 (16.7)[c] (severe gastrointestinal symptoms)<br>HCQ: not observed<br>HCQ + IVIG: not observed<br>HCQ +TCZ: not observed<br>LPVr + HCQ + INF-β: not observed<br>HCQ + INF-β: not observed<br>Not treated: not observed<br>QTc prolonged ≥500 ms:<br>HCQ: 1 (25)[c] (not mentioned if it was corrected)	64.3 (Strobe)

		HCQ + LPVr + INF-β: 2<br><br>HCQ + INF-β: 1<br><br>Not treated: 2					<u>Control group:</u><br>NA		
Clinical and microbiological effect of a combination of hydroxychloroquine and azithromycin in 80 COVID-19 patients with at least a six-day follow up: A pilot observational study.<br>Gautret P.<br>Travel Med Infect Dis. 2020	HCQ + AZ	80	Three main endpoints: clinical outcomes, contagiousness assessed by PCR and cultures and length of stay in infectious disease unit	<u>Age (years):</u><br>52 (18-88)[d]<br><br><u>Gender (male):</u><br>43 (53.8)[c]	<u>Inclusion criteria:</u><br>- PCR SARS-CoV-2 RNA from a nasopharyngeal sample<br>- Treatment with HCQ and AZM for at least 3 days<br>- Followed-up for at least 6 days<br><br><u>Exclusion criteria:</u><br>-The treatment was not started when the QTc (Bazett's formula) was >500 ms	(a) Observational<br>(b) Longitudinal<br>(c) Retrospective<br>(d) Followed-up for at least 6 days (not clear if it is since starting the treatment)<br>(e) 3th to 21th March 2020	<u>Treatment group:</u><br>HCQ 200 mg TDS for 10 days plus AZ 500 mg on day 1 followed by 250 mg QD for 4 days<br><br><u>Control group:</u><br>NA	a) Reported as possible AEs:<br><u>Nausea or vomiting:</u><br>2 (2.5)[c]<br><u>Diarrhoea:</u><br>4 (5.0)[c]<br><u>Blurred vision:</u><br>1 (1.2)[c]	56.8 (Strobe)
Clinical efficacy of hydroxychloroquine in patients with COVID-19 pneumonia who require oxygen: observational comparative study using routine care data.<br>Mahévas M.<br>BMJ. 2020	HCQ ± AZM	181<br><br><u>Groups:</u><br>HCQ within 48h of admission (treatment group): 84<br><br>Not received HCQ (control): 89	To assess the effectiveness of HCQ in patients admitted to hospital with COVID-19 pneumonia that required oxygen	<u>Age (years):</u><br>60 (52-68)[e]<br><br><u>Gender (male):</u><br>125 (72)[c]	<u>Inclusion criteria:</u><br>- Aged from 18 to 80 years old<br>- PCR confirmed SARS-CoV-2 infection<br>- Oxygen requirement by mask or nasal prongs<br><br><u>Exclusion criteria:</u><br>- Contraindication to HCQ 600 mg/day (including dialysis patients)<br>- HCQ started before admission<br>- Treatment with another experimental drug (TCZ, LPVr or REM)<br>- Organ failure that required admission to the ICU or a CCU	(a) Observational<br>(b) Longitudinal<br>(c) NA<br>(d) From hospital admission to death, loss of follow-up or end of follow-up (24th April)<br>(e) 12th to 31st March 2020	<u>Treatment group:</u><br>HCQ 600 mg/day within 48 h of hospitalization<br><br><u>Control group:</u><br>SOC	(a)*<u>Discontinuation due to ECG modifications:</u><br>HCQ ± AZM: 8 (9.5)[c]<br>SOC: NA<br>*<u>QTc change ≥60 ms:</u><br>HCQ ± AZM: 7 (8.3)[c]<br>SOC: NA<br>*<u>QTc prolonged ≥500 ms:</u><br>HCQ ± AZM: 1 (1.2)[c]<br>SOC: NA<br>*<u>Other (first degree AVB):</u><br>HCQ ± AZM: 1 (1.2)[c]<br>SOC: NA<br><br>*None of the affected cases took AZM	92.9 (Strobe)

		AZM was administered to 18% of the participants in the treatment group vs 29% in the control group			- ARDS at admission<br>- Discharge from the ICU to standard care<br>- Limitation or stop of active treatments at admission<br>- Opposition to data collection				
Risk of QT interval prolongation associated with use of hydroxychloroquine with or without concomitant azithromycin among hospitalized patients testing positive for coronavirus disease 2019 (COVID-2019). Mercuro NJ. JAMA Cardiol. 2020	HCQ or HCQ + AZM	90<br><br>Groups:<br>HCQ: 37<br><br>HCQ + AZM: 53	To characterize the risk and degree of QT prolongation in patients with COVID-19 in association with their use of HCQ with or without concomitant AZM	Age (years):<br>60.1 (16.7)[b]<br><br>Gender (male):<br>46 (51.11)[c]	Inclusion criteria:<br>- Hospitalized patients who received at least 1 day of HCQ and had at least 1 positive COVID-19 nasopharyngeal PCR<br><br>Exclusion criteria:<br>NA	(a) Observational<br>(b) Longitudinal<br>(c) Retrospective<br>(d) NA<br>(e) 1st March to 7th April 2020	Treatment group:<br>HCQ 400 mg BD for 1 day (loading dose) followed by 400 mg QD for 4 days ± AZM<br><br>Control group:<br>NA	a) ΔQTc (ms):<br>HCQ + AZM: 23 (10-40)[e]<br>HCQ: 5.5 (-15.5-34.25)[e]<br>QTc prolongued ≥500 ms:<br>HCQ + AZM: 11 (21)[c]<br>HCQ: 7 (19)[c]<br>QTc change ≥60 ms:<br>HCQ + AZM: 7 (13)[c]<br>HCQ: 3 (8)[c]<br>Discontinuation due to QT prolongation:<br>HCQ ± AZM: 10 (11)[c]<br>Other (possible association):<br>HCQ ± AZM: intractable nausea, premature ventricular contractions and right bundle branch block and hypoglycaemia<br><br>-One patient who had HCQ and AZM discontinued due to QTc prolongation (499 ms), developed TdP 3 days later and subsequently developed other ventricular arrhythmias	75.8 (Strobe)
Early treatment of COVID-19 patients with hydroxychloroquine and azithromycin: A retrospective analysis of 1061 cases in	HCQ + AZM	1,061	Outcomes were: death, clinical worsening (aggressive clinical course requiring oxygen, transfer to ICU, and >10 days hospitalization	Age (years):<br>43.6 (15.6)[b]<br><br>Gender (male): 492 (46.4)[c]	Inclusion criteria:<br>- PCR documented SARS-CoV-2 RNA from a nasopharyngeal sample<br>- HCQ + AZM early treatment, whether or not they had symptoms<br>- At least 3 days of treatment and 9 days of follow-up<br><br>Exclusion criteria:	(a) Observational<br>(b) Longitudinal<br>(c) Retrospective<br>(d) From PCR-documented SARS-CoV-2 RNA in day-care hospital (inpatients) or at infectious disease units (inpatients)	Treatment group:<br>HCQ 200 mg TDS for 10 days + AZM 500 mg on day 1 (loading dose) followed by 250 mg QD for 4 days<br><br>Control group:<br>NA	(a) QTc prolonged ≥500 ms:<br>Not observed<br>QTc change ≥60 ms:<br>9 (0.8)[c]<br>TdP:<br>Not observed<br>Arrhythmogenic deaths:<br>Not observed<br>Patient's without any AEs:<br>1,036 (97.6)[c]<br>Mild AEs possibly related to treatment:	86.4 (Strobe)

Marseille, France. Million M. Travel Med Infect Dis. 2020			) and viral shedding persistence (>10 days)		- <14 years<br>- Pregnant women<br>- G6PD deficiency (based on patient's declaration)	and a minimum of 9 days<br>(e) 3th to 31st March 2020. A new evaluation of data to update fatal cases and case fatality rates was done on 18th April 2020		25 (2.3)[c]<br>Discontinuation due to non-cardiac AEs:<br>3 (0.3)[c]<br>Diarrhoea:<br>12 (1.13)[c]<br>Other (n):<br>Abdominal pain (3), headache (3), nausea (2), insomnia (2), transient blurred vision (2), vomiting (1), urticaria (1), erythematous and bullous rash (1)	
No evidence of rapid antiviral clearance or clinical benefit with the combination of hydroxychloroquine and azithromycin in patients with severe COVID-19 infection. Molina JM. Med Mal Infect. 2020	HCQ + AZM	11	To assess the virologic and clinical outcomes of hospitalised patients who received HCQ plus AZM	Age (years):<br>58.7 (20-77)[d]<br><br>Gender (male):<br>7 (63.63)[c]	Inclusion criteria:<br>- Hospitalized patients<br><br>Exclusion criteria:<br>NA	(a) Observational<br>(b) Longitudinal<br>(c) Prospective<br>(d) NA<br>(e) NA	Treatment group:<br>HCQ 600 mg/day for 10 days plus AZM 500 mg on day 1 followed by 250 mg QD for 4 days<br><br>Control group:<br>NA	a) Discontinuation due to QT prolongation:<br>1 (9.09)[c]	NA
Association of treatment with hydroxychloroquine or azithromycin with in-hospital mortality in patients with COVID-19 in New York State. Rosenberg ES. JAMA. 2020	HCQ or HCQ + AZM or AZM	1,438<br><br>Groups:<br>HCQ: 271<br><br>HCQ + AZM: 735<br><br>AZM: 211<br><br>Neither drug: 221	To understand prescribing patterns of HCQ and AZM in hospitalized patients with COVID-19 and the association of these drugs with mortality and possible adverse events	Age (years, median):<br>63<br><br>Gender (male): 858 (59.7)[c]	Inclusion criteria:<br>- Laboratory confirmed COVID-19 admitted to hospitals in the New York City metropolitan region during the sampling period<br><br>Exclusion criteria:<br>NA	(a) Observational<br>(b) Longitudinal<br>(c) Retrospective<br>(d) Admission period<br>(e) 15th to 28th March 2020. Final follow-up was 24th April 2020	Treatment groups:<br>Diverse regimens of HCQ and AZM were reported<br><br>Control group:<br>NA	(a) Abnormal ECG (arrhythmia or prolonged QT fraction):<br>HCQ: 74 (27.3)[c]<br>HCQ+AZM: 199 (27.1)[c]<br>AZM: 34 (16.1)[c]<br>Neither: 31 (14.0)[c]<br>$p < 0.001$<br>QT prolongation:<br>HCQ: 39 (14.4)[c]<br>HCQ+AZM: 81 (11.0)[c]<br>AZM: 15 (7.1)[c]<br>Neither: 13 (5.9)[c]<br>$p = 0.006$<br>Arrhythmia:<br>HCQ: 44 (16.2)[c]<br>HCQ+AZM: 150 (20.4)[c]<br>AZM: 23 (10.9)[c]<br>Neither: 23 (10.4)[c]<br>$p < 0.001$	93.2 (Strobe)

								Cardiac arrest:<br>HCQ: 37 (13.7)[c]<br>HCQ+AZM: 114 (15.5)[c]<br>AZM: 13 (6.2)[c]<br>Neither: 15 (6.8)[c]<br>p <0.001<br>Diarrhoea:<br>HCQ: 22 (17.0)[c]<br>HCQ+AZM: 85 (11.6)[c]<br>AZ: 16 (8.5)[c]<br>Neither: 16 (7.2)[c]<br>p = 0.003<br>Hypoglycaemia:<br>HCQ: 9 (3.3)[c]<br>HCQ+AZM: 25 (3.4)[c]<br>AZM: 1 (0.5)[c]<br>Neither: 6 (2.7)[c]<br>p = 0.15	
Effect of chloroquine, hydroxychloroquine and azithromycin on the corrected QT interval in patients with SARS-CoV-2 infection. Saleh M. Circ Arrhythm Electrophysiol. 2020	CQ or HCQ ± AZM	201<br><br>Groups:<br>CQ ± AZM: 10<br><br>HCQ ± AZM: 191<br><br>(A total of 119 patients received CQ or HCQ + AZM)	To assess the arrhythmic safety of CQ or HCQ ± AZ	Age (years):<br>58.5 (9.1)[b]<br><br>Gender (male):<br>115 (57.2)[c]	Inclusion criteria:<br>- Hospitalized<br>- >18 years<br>- PCR confirmed COVID-19 illness<br>- Treated with CQ or HCQ ± AZM<br>- The decision to treat with CQ or HCQ ± AZM was based on physician decision and healthcare system guidelines (COVID-19 PCR confirmed or suspected with test pending, ARDS or severe illness according to SIRS criteria or possibly to progressing towards ARDS or severe illness in the next 6 h)<br>Exclusion criteria:<br>- Patients not meeting criteria for treating<br>- Patients chronically treated with HCQ<br>- Documented hypersensitivity to any of the agents<br>- Patients who refused therapies	(a) Observational<br>(b) Longitudinal<br>(c) Prospective<br>(d) NA<br>(e) 1st to 23th March 2020	CQ:<br>500 mg BD for 1 day (loading dose) followed by 500 mg QD for 4 days<br><br>HCQ:<br>400 mg BD for 1 day (loading dose) followed by 200 mg BD for 4 days<br><br>AZM (oral or IV):<br>500 mg QD for 5 days<br><br>Control group:<br>NA	a) ΔQTc (ms), baseline to maximum:<br>CQ or HCQ: 14.4 (25.0)[b]<br>CQ or HCQ + AZM: 30.4 (40.2)[b]<br>p <0.001<br>QTc prolonged ≥500 ms:<br>CQ or HCQ: 7 (8.5)[c]<br>CQ or HCQ + AZM: 11 (9.2)[c]<br>p = 1.00<br>Discontinuation due to QT prolongation:<br>CQ or HCQ: 2 (2.4)[c]<br>CQ or HCQ + AZM: 5 (4.2)[c]<br>TdP:<br>Not observed<br>Arrhythmogenic deaths:<br>Not observed	86 (Strobe)
Chloroquine-induced QTc	CQ	95	To assess the degree of CQ-	Age (years):<br>65 (18–91)[f]	Inclusion criteria:<br>- ≥18 years old	(a) Observational<br>(b) Longitudinal	Treatment group:	a) ΔQTc (ms, mean):<br>Computer interpreted:	70.5 (Strobe)

prolongation in COVID-19 patients. Van den Broek MPH. Neth Heart J. 2020			induced QTc prolongation in hospitalised COVID-19 patients	Gender (male):<br>63 (66)[c]	- Hospitalised and suspected for having COVID-19 disease<br>- CQ treatment<br>- A baseline ECG and an ECG during CQ therapy<br><br>Exclusion criteria:<br>- Baseline QTc interval duration of >500 ms (CQ not prescribed in accordance with hospital policy)	(c) Retrospective<br>(d) 24 h before the initiation of CQ treatment and 24-48h after the initiation of therapy<br>(e) 8th to 27th March 2020	CQ 600 mg (loading dose) followed by 300 mg BD (starting 12 h after the loading dose) for 5 days<br><br>Control group:<br>NA	35 (95% CI 28-43)<br>Manually interpreted:<br>34 (95% CI 25-43)<br>QTc prolongued ≥500 ms:<br>22 (23)[c]<br>TdP:<br>Not observed<br>Clinically relevant ventricular arrhythmias:<br>Not observed	
Effect of high vs low doses of chloroquine diphosphate as adjunctive therapy for patients hospitalized with severe acute respiratory syndrome Coronavirus 2 (SARS-CoV-2) infection: a randomized clinical Trial. Borba MGS. JAMA Netw Open. 2020	CQ diphosphate + AZM + ceftriaxone ± oseltamivir	81<br><br>Groups:<br>Low-dose CQ: 40<br><br>High-dose CQ: 41	To assess the safety and efficacy of CQ in the treatment of hospitalized patients with severe respiratory syndrome secondary to SARS-CoV-2 infection	Age (years):<br>51.1 (13.19)[b]<br><br>Gender (male):<br>60 (75.3)[b]	Inclusion criteria:<br>- Hospitalized with clinical suspicion of COVID-19 disease<br>- ≥18 years old<br>- Respiratory rate > 24 breathes per minute and/or heart rate > 125 bpm (in the absence of fever) and/or peripheral oxygen saturation < 90% in ambient air and/or shock (e.g. arterial pressure < 65 mmHg, with the need for vasopressor medicines, oliguria or a lower level of consciousness)<br><br>Exclusion criteria:<br>- <18 years old	(a) Randomized double blind - parallel group trial<br>(b) Longitudinal<br>(c) Prospective<br>(d) 28 days<br>(e) 23th March to 5th April 2020	Low-dose CQ:<br>450 mg BD for 1 day (loading dose) followed by 450 mg QD for 4 days plus AZM 500 mg QD for 5 days plus ceftriaxone 1 g BD for 7 days ± oseltamivir 75 mg BD for 5 days<br><br>High-dose CQ:<br>600 mg BD for 10 days plus AZM 500 mg QD for 5 days plus ceftriaxone 1 g BD for 7 days ± oseltamivir 75 mg BD for 5 days	a) QTc prolonged ≥500 ms:<br>Clinical suspicion of COVID-19:<br>Low-dose CQ: 4/36 (11.1)[c]<br>High-dose CQ: 7/37 (18.9)[c]<br><br>COVID-19 confirmed:<br>Low-dose CQ: 1/27 (3.6)[c]<br>High-dose CQ: 7/29 (24.1)[c]<br><br>Ventricular tachycardia:<br>Clinical suspicion of COVID-19:<br>Low-dose CQ: Not observed<br>High-dose CQ: 2/37 (5.4)[c]<br><br>COVID-19 confirmed:<br>Low-dose CQ: Not observed<br>High-dose CQ: 2/31 (6.5)[c]<br><br>Haemoglobin decrease:<br>Clinical suspicion of COVID-19:<br>Low-dose CQ: 4/18 (22.2)[c]<br>High-dose CQ: 7/24 (19.2)[c]<br><br>COVID-19 confirmed:<br>Low-dose CQ: 3/11 (27.3)[c]<br>High-dose CQ: 4/18 (22.2)[c]<br><br>Creatinine increase:<br>Clinical suspicion of COVID-19:<br>Low-dose CQ: 7/15 (46.7)[c]<br>High-dose CQ: 9/23 (39.1)[c]<br><br>COVID-19 confirmed:<br>Low-dose CQ: 5/9 (55.6)[c]<br>High-dose CQ: 8/18 (44.4)[c]<br><br>Creatine Kinase increase:	5 (Jadad)

								Clinical suspicion of COVID-19:<br>Low-dose CQ: 6/19 (31.6)[c]<br>High-dose CQ: 7/14 (50)[c]<br><br>COVID-19 confirmed:<br>Low-dose CQ: 3/15 (20.0)[c]<br>High-dose CQ: 6/9 (66.7)[c]<br><br>Creatine Kinase MB increase:<br>Clinical suspicion of COVID-19:<br>Low-dose CQ: 3/13 (23.1)[c]<br>High-dose CQ: 7/13 (53.8)[c]<br><br>COVID-19 confirmed:<br>Low-dose CQ: 3/13 (23.1)[c]<br>High-dose CQ: 4/9 (44.4)[c]	
Efficacy of hydroxychloroquine in patients with COVID-19: results of a randomized clinical trial. Chen Z. MedRxiv. 2020	HCQ	62<br><br>Groups:<br>HCQ: 31<br><br>Control: 31	Time to clinical recovery, clinical characteristics and radiological results assessed at baseline and 5 days after treatment	Age (years):<br>44.7 (15.3)[b]<br><br>Gender (male):<br>29 (46.8)[c]	Inclusion criteria:<br>- ≥ 18 years old<br>- RT-PCR positive of SARS-CoV-2<br>- Chest CT with pneumonia<br>- SaO2/SPO2 ratio > 93% or PaO2/FIO2 ratio > 300 mmHg under the condition in the hospital room (mild illness)<br>- Willing to receive a random assignment to any designated treatment group<br><br>Exclusion criteria:<br>- Not participating in another study at the same time<br>- Severe and critical illness patients or participating in the trial does not meet the patient's maximum benefit or does not meet any criteria for safe follow-up in the protocol after a physician's evaluation<br>- Retinopathy and other retinal diseases<br>- Conduction block and other arrhythmias<br>- Severe liver disease (e.g. Child-Pugh score C or AST> twice the upper limit)<br>- Pregnant or breastfeeding	(a) Randomized double blind - parallel group trial (neither the research performers nor the patients were aware of the treatment assignments)<br>(b) Longitudinal<br>(c) Prospective<br>(d) Assessed at baseline and 5 days after treatment (or until severe reactions appeared)<br>(e) 4th to 28th February 2020	Treatment group:<br>HCQ 200 mg BD between days 1 and 5 plus standard treatment (oxygen therapy, antiviral agents, antibacterial agents and immunoglobulin, with or without corticosteroids)<br><br>Control group:<br>Standard treatment (oxygen therapy, antiviral agents, antibacterial agents and immunoglobulin, with or without corticosteroids)	a) Rash:<br>HCQ: 1 (3.2)[c]<br>Control: Not observed<br>Headache:<br>HCQ: 1 (3.2)[c]<br>Control: Not observed<br>Severe side effects:<br>HCQ: Not observed<br>Control: Not observed	4 (Jadad)

					- Severe renal failure (eGFR 30 ml/min/1.73m^2 or receiving renal replacement therapy)<br>- Possibility of being transferred to another hospital within 72 h<br>- Received any trial treatment for COVID-19 within 30 days before this research				
Treating COVID-19 with chloroquine. Huang M. J Mol Cell Biol. 2020	CQ or LPVr	22<br><br><u>Groups:</u><br>CQ: 10<br><br>LPVr: 12	Efficacy and safety of CQ in hospitalized patients with COVID-19	<u>Age (years):</u><br>All patients: 44.0 (36.5-57.5)[e]<br>CQ: 41.5 (33.8-50.0)[e]<br>LPVr: 53.0 (41.8-63.5)[e]<br><u>Gender (female):</u><br>All patients: 9 (40.9)[c]<br>CQ: 3 (70.0)[c]<br>LPVr: 6 (50.0)[c]	<u>Inclusion criteria:</u><br>- ≥18 years old<br>- Patients diagnosed with COVID-19 according to WHO interim guidance<br><br><u>Exclusion criteria:</u><br>- Pregnant woman<br>- Documented allergic history to CQ<br>- Documented history of haematological system diseases<br>- Documented history of chronic liver and kidney diseases<br>- Documented history of cardiac arrhythmia or chronic heart diseases<br>- Documented history of retina or hearing dysfunction<br>- Documented history of mental illnesses<br>- Use of digitalis due to the previous disease	(a) Randomized<br>(b) Longitudinal<br>(c) Prospective<br>(d) Followed-up for 14 days (not clear if it is since starting the treatment)<br>(e) 27th January to 15th February 2020	<u>CQ:</u><br>500 mg BD for 10 days<br><br><u>LPVr:</u><br>400/100 mg BD for 10 days	a) <u>Total AEs:</u><br>CQ: 9 (90)[c]<br>LPVr: 10 (83.33)[c]<br>p = 0.99<br><u>Shortness of breath:</u><br>CQ : 1 (10)[c]<br>LPVr : 4 (33.33)[c]<br>p = 0.32<br><u>Diarrhoea:</u><br>CQ: 5 (50)[c]<br>LPVr: 8 (66.67)[c]<br>p = 0.67<br><u>Vomiting:</u><br>CQ: 5 (50)[c]<br>LPVr: 1 (8.33)[c]<br>p = 0.06<br><u>Abdominal pain:</u><br>CQ: 1 (10)[c]<br>LPVr: 2 (16.67)[c]<br>p = 0.99<br><u>Nausea:</u><br>CQ: 4 (40)[c]<br>LPVr: 5 (41.57)[c]<br>p = 0.99<br><u>Dizziness:</u><br>CQ: Not observed<br>LPVr: 2 (16.67)[c]<br>p = 0.48<br><u>Headache:</u><br>CQ: Not observed<br>LPVr: 1 (8.33)[c]<br>p = 0.99<br><u>Psychosis:</u><br>CQ: Not observed<br>LPVr: 1 (8.33)[c]<br>p = 0.99<br><u>Rash or itchy:</u><br>CQ: 1 (10)[c]	2 (Jadad)

								LPVr: Not observed<br>p = 0.45<br>Respiratory cough:<br>CQ: 4 (40)[c]<br>LPVr: 6 (50)[c]<br>p = 0.69	
Hydroxychloroquine in patients with mainly mild to moderate coronavirus disease 2019: open label, randomised controlled trial. Tang W. BMJ. 2020	HCQ	150<br><br>Groups:<br>HCQ + SOC: 70<br><br>SOC: 80	Efficacy and safety of HCQ plus SOC compared with SOC alone in adults with COVID- 19	Age (years):<br>48 (14.1)[b]<br><br>Gender (male): 82 (55)[c]	Inclusion criteria:<br>- ≥ 18 years old<br>- Ongoing SARS-CoV-2 infection confirmed in upper or lower respiratory tract specimens with RT-PCR<br>- Willingness to participate and consent<br>- Not included in other clinical trials during the study period<br><br>Exclusion criteria:<br>- Below 18 years<br>- Severe conditions (malignancies, heart, liver or kidney disease)<br>- Poorly controlled metabolic diseases<br>- Unsuitability for oral administration<br>- Pregnancy or lactation<br>- Allergy to HCQ<br>- Inability to cooperate due to cognitive impairments or poor mental status<br>- Severe hepatic impairment (e.g. Child-Pugh grade C, ALT more than 5 fold the upper limit)<br>- Severe renal impairment (eGFR ≤ 30 ml/min/1.73m^2) or receipt of continuous renal replacement therapy, haemodialysis or peritoneal dialysis	(a) Randomized open label controlled trial<br>(b) Longitudinal<br>(c) Prospective<br>(d) From treatment (within 24 h after randomization), until post-treatment follow-up at scheduled visits on days 4, 7, 10, 14, 21, and 28<br>(e) 11th to 29th February 2020	HCQ + SOC:<br>HCQ loading dose of 1.2 g daily for 3 days followed by 800 mg daily (total treatment duration: 2 w for mild/moderate disease and 3 w for severe disease) plus SOC<br><br>SOC:<br>Any of the following treatments: antiviral agents (arbidol, virazole, LPVr, oseltamivir, entecavir), antibiotics, systemic glucocorticoids	(a) Any type of AEs:<br>HCQ+SOC: 21 (30)[c]<br>SOC: 7 (9)[c]<br>AEs reported as serious:<br>HCQ+SOC: 2 (3)[c]<br>SOC: Not observed<br>AEs reported as non-serious:<br>HCQ+SOC: 19 (27)[c]<br>SOC: 7 (9)[c]<br>Diarrhea:<br>HCQ+SOC: 7 (10)[c]<br>SOC: Not observed<br>Other (n):<br>HCQ+SOC: disease progression (1), upper respiratory tract infection (1), vomiting (2), nausea (1), abdominal discomfort (1), thirst (1), sinus bradycardia (1), hypertension (1) orthostatic hypotension (1), hypertriglyceridemia (1), decrease appetite (1), fatigue (1), dyspnoea (1), flush (1), coagulation dysfunction (1), blurred vision (1),<br>decreased white blood cells (1), increased ALT (1), increased serum amylase (1), decreased neutrophil count (1)<br>SOC: abdominal bloating (1), fever (1), liver abnormality (1), hepatic steatosis (1), otitis externa (1), increased ALT (1), increased serum amyloid A (1)	3 (Jadad)
Safety of Hydroxychloroquine Among Outpatient Clinical Trial	HCQ	2795 individuals were enrolled into the 3 trials:	Investigate HCQ as prophylaxis and treatment for COVID-19	Age (years):<br>40 (34–49)[e]<br><br>Gender (female):<br>51 %	Inclusion criteria:<br>Participants were enrolled in the 3 trials via internet-based surveys throughout the United States and selected Canadian provinces	(a) Three randomized, double-blind, placebo-controlled trials<br>(b) Longitudinal	HCQ dosing for both the PEP and PET trials was 0.8 g load dosing, followed by 0.6 g 6–8 h	(a) Overall 2544 (91%) participants reported side effect data, and 748 (29%) reported at least 1 medication side effect.	4 (Jadad)

Participants for COVID-19. Lofgren SM. Open Forum Infect Dis. 2020		Once daily (PEP+PET)1.312<br><br>Postexposure prophylaxis (PEP) trial: n = 821<br><br>Preemptive early treatment (PET) trial: n = 491<br><br>Weekly or twice weekly: 1.483<br><br>Pre-exposure prophylaxis (PREP) trial: n = 1.483	disease versus placebo.<br>The first 2 trials evaluated postexposure prophylaxis and preemptive early treatment. The third trial assessed pre-exposure prophylaxis for COVID-19		Exclusion criteria:<br>Participants were excluded if they were <18 years old, had an allergy to HCQ, retinal eye disease, known glucose-6 phosphate dehydrogenase (G6PD) deficiency, known chronic kidney disease that was stage 4 or 5 or receiving dialysis, known porphyria, weight <40 kg, known QT prolongation, or receiving chemotherapy. Current use of AZM, or cardiac arrhythmia medicines was also an exclusion criterion.	(c) Prospective<br>(d) PEP and PET trials enrollment began on March 17, 2020, and concluded on May 6. PREP trial enrollment began April 6 and ended May 26, 2020<br>(e) Follow-up of PEP and PET trials was completed on May 20, 2020.<br>PREP trial follow-up concluded on July 13, 2020	later, and then 0.6 g daily for 5 days in total.<br><br>HCQ dosing for PREP trial was dosed at 0.4 g orally once, followed by 0.4 g 6 to 8 h later, and thereafter 0.4 g weekly or twice weekly for the duration of follow-up, up to 12 weeks.<br><br>The placebo was dosed similarly	Side effects reported: HCQ once-daily: 40% HCQ twice-weekly: 36% HCQ once-weekly: 31%<br><br>Upset stomach or nausea:<br>HCQ once-daily: 146(25.3)[c]<br>HCQ twice-weekly: 90(19.4)[c]<br>HCQ once-weekly: 83(17.5)[c]<br><br>Diarrhea, vomiting, or abdominal pain:<br>HCQ once-daily: 131(22.7)[c]<br>HCQ twice-weekly: 79(17.1)[c]<br>HCQ once-weekly: 61(12.9)[c]<br><br>Palpitations:<br>HCQ once-daily: NA<br>HCQ twice-weekly: 6(1.3)[c]<br>HCQ once-weekly: 4(0.8)[c]<br><br>Irritability, dizziness, vertigo:<br>HCQ once-daily: 39(6.8)[c]<br>HCQ twice-weekly: 24(5.2)[c]<br>HCQ once-weekly: 27(5.7)[c]<br><br>Tinnitus:<br>HCQ once-daily: 16(2.8)[c]<br>HCQ twice-weekly: 7(1.5)[c]<br>HCQ once-weekly: 10(2.1)[c]<br><br>Headache:<br>HCQ once-daily: 15(2.6)[c]<br>HCQ twice-weekly: NA<br>HCQ once-weekly: NA<br><br>Fatigue:<br>HCQ once-daily: 1(0.2)[c]<br>HCQ twice-weekly: 5(1.1)[c]<br>HCQ once-weekly: 1(0.2)[c]<br><br>Visual changes:<br>HCQ once-daily: 7(1.2)[c]	

								HCQ twice-weekly: 4(0.9)[c]<br>HCQ once-weekly: 7(1.5)[c]<br><br>Skin reaction:<br>HCQ once-daily: 10(1.7)[c]<br>HCQ twice-weekly: 23(5.0)[c]<br>HCQ once-weekly: 13(2.7)[c]<br><br>Taste change or dry mouth:<br>HCQ once-daily: 3(0.5)[c]<br>HCQ twice-weekly: NA<br>HCQ once-weekly: NA<br><br>Allergic reaction:<br>HCQ once-daily: 1(1.0)[c]<br>HCQ twice-weekly: 4(0.9)[c]<br>HCQ once-weekly: 2(0.4)[c]<br><br>Hot flashes, night sweats or palpitations:<br>HCQ once-daily: 2(0.3)[c]<br>HCQ twice-weekly: NA<br>HCQ once-weekly: NA<br><br>Panic:<br>HCQ once-daily: 0(0.0)[c]<br>HCQ twice-weekly: NA<br>HCQ once-weekly: NA<br><br>Sleep disturbance:<br>HCQ once-daily: NA<br>HCQ twice-weekly: 7(1.5)[c]<br>HCQ once-weekly: 10(2.1)[c]<br><br>Myalgia:<br>HCQ once-daily: NA<br>HCQ twice-weekly: 2(0.4)[c]<br>HCQ once-weekly: 7(1.5)[c]<br><br>Arrhythmias:<br>HCQ once-daily: NA<br>HCQ twice-weekly: 1(0.2)[c]	

								HCQ once-weekly: 0(0.0)[c]<br><br><u>Sudden deaths:</u><br>HCQ once-daily: 0(0.0)[c]<br>HCQ twice-weekly: 0(0.0)[c]<br>HCQ once-weekly: 0(0.0)[c]	
Hydroxychloroquine for Early Treatment of Adults with Mild Covid-19: A Randomized-Controlled Trial. Mitjà O. Clin Infect Dis. 2020	HCQ	293<br><br><u>Groups:</u><br>HCQ: 136<br><br>SOC: 157	To determine whether early treatment with HCQ would be efficacious for outpatients with COVID-19	<u>Age (years):</u><br>41.6 (12.6)b<br><br><u>Gender (female):</u><br>68.6%	<u>Inclusion criteria:</u><br>Adult ≥18 years, mild symptoms of COVID-19 (ie, fever, acute cough, shortness of breath, sudden olfactory or gustatory loss, or influenza-like illness) for fewer than 5 days before enrollment, non hospitalized and a positive PCR test for SARSCoV-2 in the baseline<br><br><u>Exclusion criteria:</u><br>Moderate to severe COVID-19 disease, any condition that might preclude following the study procedures safely, known allergy or hypersensitivity to study drugs, known retinal and severe liver or renal diseases, history of cardiac arrhythmia, known electrocardiographic QT interval prolongation or other diseases that could be exacerbated by study drugs (eg, psoriasis), active treatment with medications that are contraindicated with study drugs, or were living with human immunodeficiency virus (HIV). Females who were pregnant (verbally declared or positive pregnancy test) or breastfeeding were also excluded	(a) Multicenter open-label, randomized, controlled trial<br>(b) Longitudinal<br>(c) Prospective<br>(d) Between 17 March and 26 May 2020<br>(e) Participants were assessed on day 1 (baseline, HCQ was started) and days 3, 7, 14, and 28	<u>HCQ:</u><br>0.8 g on day 1 followed by 0.4 g once daily for 6 days<br><br><u>SOC:</u><br>NA	**(a)** <u>Any AE:</u><br>HCQ: 121(72)c<br>SOC: 16 (8.7)c<br><u>Cardiac disorders:</u><br>HCQ: 0 (0)c<br>SOC: 0 (0)c<br><u>Ear and labyrinth disorders</u>:<br>HCQ: 5 (3)c<br>SOC: 0 (0)c<br><u>Eye disorders:</u><br>HCQ: 5 (3) c<br>SOC: 0 (0)c<br><u>Gastrointestinal disorders:</u><br>HCQ: 148 (88.1)c<br>SOC: 7 (3.8)c<br><u>General disorders:</u><br>HCQ: 30 (17.9)c<br>SOC: 1 (0.5)c<br><u>Infections and infestations:</u><br>HCQ: 9 (5.4)c<br>SOC: 12 (6.6)c<br><u>Injury, poisoning, and procedural complications:</u><br>HCQ: 1 (0.6)c<br>SOC: 0 (0)c<br><u>Metabolism and nutrition disorders:</u><br>HCQ: 2 (1.2)c<br>SOC: 1 (0.5)c<br><u>Musculoskeletal and connective tissue disorders:</u><br>HCQ: 1 (0.6)c<br>SOC: 0 (0)c<br><u>Nervous system disorders:</u><br>HCQ: 63 (37.5)c<br>SOC: 3 (1.6)c	3 (Jadad)

								Psychiatric disorders:<br>HCQ: 2 (1.2)c<br>SOC: 0 (0)c<br>Renal and urinary disorders:<br>HCQ: 1 (0.6)c<br>SOC: 0 (0)c<br>Reproductive system and breast disorders:<br>HCQ: 1 (0.6)c<br>SOC: 0 (0)c<br>Respiratory, thoracic, and mediastinal disorders:<br>HCQ: 2 (1.2)c<br>SOC: 0 (0)c<br>Skin and subcutaneous tissue disorders:<br>HCQ: 11 (6.5)c<br>SOC: 0(0)c<br>Vascular disorders:<br>HCQ: 1 (0.6)c<br>SOC: 0 (0)c<br>Deaths:<br>HCQ: 0 (0)c<br>SOC: 0 (0)c	
Hydroxychloroquine versus lopinavir/ritonavir in severe COVID-19 patients : Results from a real-life patient cohort. Karolyi M. Wien Klin Wochenschr. 2021.	HCQ or LPVr	156<br><br>Groups:<br>HCQ: 20<br><br>LPVr: 47<br><br>No treatment: 89	To describe the efficacy and safety of antiviral treatment with HCQ or LPV/RTV in a real-life cohort of patients with COVID19	Age (years):<br>72 (55.25–81)e<br><br>Gender (female):<br>64 (41)c	Inclusion criteria:<br>≥18 years old with molecular proven SARS-CoV-2 infections, need for hospitalization plus respiratory insufficiency ($SpO2 \leq 93\%$ at room air or need for oxygen insufflation) or bilateral consolidations as demonstrated on chest X-ray and at least two comorbidities associated with poor COVID19 prognosis (e.g. age >60 years, diabetes and hypertension)<br><br>Exclusion criteria:<br>Patients who did not fulfil the inclusion criteria, contraindications for and known allergies to both drugs, low life expectancy,	(a) Observational<br>(b) Longitudinal<br>(c) Retrospective<br>(d) From 1st March to 26th April 2020<br>(e) NA	HCQ:<br>Loading dose of 0.4 g twice daily on the first day, followed by 0.2 g twice daily<br><br>LPVr:<br>0.4g/0.1g was administered twice daily	**(a)** Stopped treatment due to side effects<br>HCQ: 0 (0)c<br>LPVr: 6 (15.4)c<br>Nausea<br>HCQ: 1 (5)c<br>LPVr: 12 (25.2)c<br>Diarrhea<br>HCQ: 0(0)c<br>LPVr: 7 (14.9)c<br>Elevated liver enzymes<br>HCQ: 2 (10)c<br>LPVr: 7 (14.9)c	69 (Strobe)

					palliative setting, inability to understand and/or agree to the off-label use				
Association of Hydroxychloroquine With QTc Interval in Patients With COVID-19. Mazzanti A. Circulation. 2020	HCQ or HCQ+AZM or HCQ+LPV/r or HCQ+AZM+LPV/r	150<br><br>Groups:<br>HCQ alone<br><br>HCQ in combination:<br>In 67% of cases, HCQ was associated with AZM (26%), LPV/r (35%), or AZM+LPV/r(6%)	To determine whether the short-term use of HCQ alone, or in combination with at least 1 other hERG-blocking COVID-19 drug, is associated with an excessive QT prolongation, defined as QTc interval ≥500 ms	Age (years):<br>69 (57-81)e<br><br>Gender (male):<br>63 %	Inclusion criteria:<br>Diagnosis of COVID-19 confirmed by polymerase chain reaction, who were admitted to hospital<br><br>Exclusion criteria:<br>NA	(a) Observational<br>(b) Longitudinal<br>(c) Prospective<br>(d) Between March 7, 2020, and April 30, 2020<br>(e) NA	HCQ alone/in combination:<br>A daily dosage of 0.4 g (97% of patient), or 0.6 g (3%) for a median of 9 days (IQR 5–11 days). In 67% of cases, HCQ was associated with AZ (26%), LPV/r (35%), or AZ+LPV/r (6%).	(a)QTc interval:<br>433 ms (414–447 ms)e without differences between HCQ monotherapy and combination therapies (P=0.742)<br><br>In 79 of the 150 patients (53%) with an ECG off-therapy also available the median QTc interval was 414 ms (IQR 397–436 ms) at baseline and 435 ms (IQR 416–451 ms) during treatment, with a median increase of 18 ms (IQR 2–34 ms; P<0.001)<br><br>Heart rate:<br>Did not change<br>PR interval:<br>Prolonged by a median of 6 ms (IQR −5 to 20 ms), from 163 ms (IQR 150–180 ms) to 165 ms (IQR 153–186 ms; P=0.006)<br>First-degree atrioventricular block:<br>4 patients<br>QRS interval:<br>Lengthened by a median of 4 ms (IQR −1 to 8 ms), from 92 ms (IQR 85–102 ms) to 96 ms (IQR 88–105 ms; P=0.053)<br><br>No life-threatening arrhythmic events were documented.	64 (Strobe)
Effect of hydroxychloroquine and Azithromycin on QT Interval Prolongation and Other Cardiac Arrhythmias in COVID-19 Confirmed Patients. Eftekhar SP.	HCQ or HCQ + AZM	172<br><br>Groups:<br>HCQ alone: 29<br><br>HCQ + AZM: 143	To assess QT interval prolongation and its risk factors in patients who received HCQ with or without AZM	Age (years):<br>59.2 (15.4)b<br><br>Gender (female):<br>About half of the patients were female	Inclusion criteria:<br>At least one positive real time reverse transcriptase polymerase chain reaction test (RT-PCR) of nasopharyngeal samples and age over 18 years old<br><br>Exclusion criteria:<br>< 18 years old, have severe metabolic disease, who refused to receive oral drugs (such as breastfeeding and pregnant mothers), and	(a) Observational<br>(b) Transversal<br>(c) NA<br>(d) Between March 5, 2020, and April 3, 2020<br>(e) NA	HCQ:<br>All patients received oral HCQ sulfate 0.6 g daily (0.2 g three times daily for 10 days).<br><br>HCQ + AZ:<br>HCQ 0.6 g daily (0.2 g three times daily for 10 days).	**(a)**<br>Posttreatment QTc, mean ± SD (ms):<br>HCQ: 462:5 ± 43.1b<br>HCQ+AZM: 464:3 ± 59.1b<br>p 0.488<br>ΔQTc, mean ± SD (ms):<br>HCQ: 2.6 ± 29.1b<br>HCQ+AZM: 17.2 ± 53.4b<br>p 0.463<br>Prolonged QTc: n(%)<br>HCQ: 3 (10.3)c<br>HCQ+AZM: 35 (24.5)c	71 (Strobe)

Cardiovasc Ther. 2021					patients dependent on continuous renal replacement therapy like hemodialysis		AZM 0.5 g on day one and 0.25 g for the next 4 days	p 0.094<br>Heart rate Normal: n(%)<br>HCQ: 28 (96.5)c<br>HCQ+AZM: 135 (94.4)c<br>p 0.636<br>Heart rate Tachycardia:<br>HCQ: 0 (0)c<br>HCQ+AZM: 7 (4.9)c<br>p 0.224<br>Heart rate Bradycardia:<br>HCQ: 1 (3.5)c<br>HCQ+AZM: 1 (0.7)c<br>p 0.208<br>Atrial fibrillation:<br>HCQ: 1 (3.4)c<br>HCQ+AZM: 2 (1.4)c<br>p 0.208<br>Ventricular tachycardia:<br>HCQ: 0 (0)c<br>HCQ+AZM: 2 (1.4)c<br>p 0.522<br>TdP:<br>HCQ: 1 (3.4)c<br>HCQ+AZM: 1 (0.7)c<br>p 0.208<br>ST segment elevation:<br>HCQ: 0 (0)c<br>HCQ+AZM: 4 (28)c<br>p 0.362<br>Right bundle branch block:<br>HCQ: 1 (3.4)c<br>HCQ+AZM: 2 (1.4)c<br>p 0.442<br>Left bundle branch block:<br>HCQ: 0 (0)c<br>HCQ+AZM: 1 (0.7)c<br>p 0.652<br><br>Due to QTc prolongation, 14 patients did not continue HCQ+AZM after 4 days.	

								Transient nausea and dizziness:<br>15 patients developed may be due to the side effects of HCQ	
Effect of triple antimicrobial therapy on electrocardiography parameters in patients with mild-to-moderate coronavirus disease 2019. Uğurlu Ilgın B Anatol J Cardiol. 2021	HCQ+OSMV+AZM or HCQ+OSMV+LEV	91<br><br>Groups:<br>HCQ+OSMV+AZM: 43<br><br>HCQ+OSMV+LEV: 48	To explore the changes in ECG parameters after treatment with triple combination therapy in patients with mild-to-moderate symptomatic COVID-19.	Age (years):<br>41.1±15.4 (16-74)g<br>Gender (female):<br>45 (49.5)c	Inclusion criteria:<br>Only patients with mild-to-moderate COVID-19 symptoms admitted to hospital<br><br>Exclusion criteria:<br>Critically ill patients who needed intensive care unit follow-up because of sepsis or acute respiratory distress syndrome and patients with cardiovascular manifestations such as myocardial infarction, myocardial injury, and myocarditis were excluded from the study	(a) Observational<br>(b) Longitudinal<br>(c) Retrospective<br>(d) From April 1, 2020, to April 30, 2020<br>(e) NA	HCQ: loading dose 0.8 g/day, maintenance dose 0.4 g/day<br><br>OSMV: 75 mg twice a day<br><br>AZM: loading dose 0.5 g/day, maintenance dose 0.25 g/day<br><br>LEV: 0.5 g/day	(a) Baseline heart rate, bpm:<br>HCQ+OSMV+AZM: 79±16b<br>HCQ+OSMV+LEV: 83±13b<br>p 0.147<br>Baseline QTc, ms:<br>HCQ+OSMV+AZM: 446±30b<br>HCQ+OSMV+LEV: 434±40b<br>p 0.139<br>Baseline QTD, ms: HCQ+OSMV+AZM: 20 (15-28)e<br>HCQ+OSMV+LEV: 20 (14-34)e<br>p 0.593<br>Baseline QRS duration, ms:<br>HCQ+OSMV+AZM: 107±19b<br>HCQ+OSMV+LEV: 105±20b<br>p 0.673<br>Baseline Tp-e, ms:<br>HCQ+OSMV+AZM: 102±16b<br>HCQ+OSMV+LEV: 86±18b<br>p<0.001<br>Baseline Tp-e Dispersion, ms:<br>HCQ+OSMV+AZM: 12 (8-18)e<br>HCQ+OSMV+LEV: 9 (6-15)e<br>p 0.095<br>Baseline Tp-e/QTc ratio:<br>HCQ+OSMV+AZM: 0.23±0.04b<br>HCQ+OSMV+LEV: 0.20±0.04b<br>p <0.001<br>Baseline PR interval, ms:<br>HCQ+OSMV+AZM: 150 (134-164)e<br>HCQ+OSMV+LEV: 148 (129-176)e<br>p 0.707<br>Baseline P wave duration ms:<br>HCQ+OSMV+AZM: 113±17b<br>HCQ+OSMV+LEV: 106±19b<br>p 0.105<br>Baseline P wave dispersion, ms:<br>HCQ+OSMV+AZM: 9 (5-15)e	65 (Strobe)

								HCQ+OSMV+LEV: 6 (4-11)e<br>p 0.101<br>PT Heart rate, bpm:<br>HCQ+OSMV+AZM: 72 (66-78)e<br>HCQ+OSMV+LEV: 76 (69-86)e<br>p 0.045<br>PT QTc, ms: HCQ+OSMV+AZM: 456±28b<br>HCQ+OSMV+LEV: 455±38b<br>p 0.960<br>PT QTD, ms: HCQ+OSMV+AZM: 15 (13-21)e<br>HCQ+OSMV+LEV: 20 (13-27)e<br>p 0.099<br>PT QRS duration, ms:<br>HCQ+OSMV+AZM: 112 (95-120)e<br>HCQ+OSMV+LEV: 106 (98-126)e<br>p 0.683<br>PT TP-e, ms: HCQ+OSMV+AZM: 104±14b<br>HCQ+OSMV+LEV: 95±15b<br>p 0.007<br>PT Tp-e dispersion, ms:<br>HCQ+OSMV+AZM: 10 (6-15)e<br>HCQ+OSMV+LEV: 10 (6-14)e<br>p 0.758<br>PT Tp-e/QTc ratio:<br>HCQ+OSMV+AZM:0.23±0.03b<br>HCQ+OSMV+LEV: 0.21±0.03b<br>p 0.005<br>PT PR interval, ms:<br>HCQ+OSMV+AZM: 160±24b<br>HCQ+OSMV+LEV: 163±35b<br>p 0.582<br>PT P wave duration, ms:<br>HCQ+OSMV+AZM: 115±17b<br>HCQ+OSMV+LEV: 115±121b<br>p 0.896<br>PT P wave dispersion, ms:<br>HCQ+OSMV+AZM: 9 (6-13)e<br>HCQ+OSMV+LEV: 8 (6-13)e<br>p 0.583<br>Delta QTc:	

								HCQ+OSMV+AZM: 12 (4-18)<br>HCQ+OSMV+LEV: 20.5 (10.5-35)<br>p 0.008<br>PT CRP, mg/L:<br>HCQ+OSMV+AZM: 2.01 (0.57–5.12)e<br>HCQ+OSMV+LEV: 4.38 (1.34–19.79)e<br>p 0.014	
Safety and efficacy of favipiravir versus hydroxychloroquine in management of COVID-19: A randomised controlled trial. Dabbous HM. Sci Rep. 2021	HCQ+OSMV or FVP	100<br><br>Groups:<br>HCQ: 50<br><br>FVP: 50	To evaluate the SARS-CoV-2 viral clearance on Days 3, 7 and 14. While the secondary objectives were the evaluation of the clinical outcomes on Days 3, 7, and 14, and the safety assessment of FVP	Age (years):<br>HCQ: 36.4 (11.5)b<br>FVP: 36.3 (12.5)b<br><br>Gender (male):<br>25 (50.0)c	Inclusion criteria:<br>Adults between 18 and 80 years with confirmed COVID-19 documented by diagnostic laboratory tests (e.g. nasopharyngeal swab) at the time of illness and having mild to moderate symptoms according to the national protocol classification<br><br>Exclusion criteria:<br>Patients in whom the disease was severe, as indicated by the presence of dyspnea, respiratory rate≥30/min, blood oxygen saturation≤93%, partial pressure of arterial oxygen to fraction of inspired oxygen ratio<300, and/or lung infiltrates>50% within 24–48 h or a life-threatening symptoms of the disease defined as respiratory failure, septic shock, and/or multiple organ dysfunction or failure, were excluded. Moreover, pregnant or lactating females or those who participated in any investigational clinical study, other than observational, within the previous 30 days were also excluded.	(a) Randomized, controlled<br>(b) Longitudinal<br>(c) Prospective<br>(d) from 18th April till 18th May<br>(e) Patients were followed up for a period of up to 30 days following the end of treatment for any newly developed symptoms, signs or adverse events	HCQ:<br>0.4 g 12 hourly on Day 1, followed by 0.2 g 12 hourly daily on Day 2 to 10<br><br>OSMV:<br>75 mg 12 hourly for 10 days<br><br>FVP:<br>3200 mg (1600 mg 12 hourly) loading dose on Day 1 followed by 1200 mg maintenance dose (600 mg 12 hourly daily) on Days 2 to 10.	(a) Elevated liver transaminases:<br>HCQ: 0 (0)c<br>FVP: 4 (8)c<br><br>There was one case of mortality among the COVID-19 patients in the HCQ-based arm due to an acute heart failure resulting from myocarditis on Day 8.	2 (Jadad)
Safety of hydroxychloroquine in healthcare workers for	HCQ+-AZM or ivermectin; or CQ alone	1303<br><br>Groups:<br>HCQ: 98.4%	To assess short-term AE of HCQ in healthcare workers	Age (years):<br>mean of 35<br><br>Gender (male):	Inclusion criteria:<br>Healthcare workers taking HCQ as prophylaxis<br><br>Exclusion criteria:	(a) Observational<br>(b) Transversal<br>(c) NA	HCQ:<br>0.8 g on day one in 66.1% (n=861)	(a) Any one or more adverse effects:<br>259 (19.9)c<br>Nausea:<br>114 (8.7)c	72 (Strobe)

COVID-19 prophylaxis. Faruqui AR. Indian J Med Res. 2021		HCQ+AZM: 0.8%<br><br>HCQ +ivermectin: 0.2%<br><br>CQ: 0.5%		56.2 %	NA	(d) Between April 2, and May 13, 2020,<br>(e) Online forms to collect details of Healthcare workers, comorbidities, prophylactic drugs used and AEs after the first dose of HCQ	0.4 g on day one in 28.1 % (n=366)<br><br>0.2 g on day one in 4.6 % (n=60)	Vomiting:<br>18 (1.4)c<br>Abdominal pain:<br>91 (7.0)c<br>Hypoglycaemia:<br>14 (1.1)c<br>Hypersensitivity:<br>12 (0.9)c<br>Photosensitivity:<br>7 (0.5)c<br>Cardiovascular effects:<br>9 (0.7)c<br>Hospitalization:<br>0 (0)c	
Effect of Early Treatment With Hydroxychloroquine or Lopinavir and Ritonavir on Risk of Hospitalization Among Patients With COVID-19: The TOGETHER Randomized Clinical Trial. Reis G. JAMA Netw Open. 2021	HCQ or LPV/r	685<br><br>Groups:<br>HCQ: 214<br><br>LPV/r: 244<br><br>Placebo: 227	To determine whether hydroxychloroquine or lopinavir-ritonavir reduces hospitalization among high-risk patients with early symptomatic COVID-19 in an outpatient setting	Age (years):<br>53 (18-94)f<br><br>Gender (female):<br>55%	Inclusion criteria:<br>18 years or older; reported less than 8 days since onset of flu like symptoms or chest computerized tomography scan consistent with COVID-19. Eligibility for participation also required at least one additional criterion for high risk: aged 50 years or older; presence of pulmonary disease, specifically moderate or severe persistent asthma, chronic obstructive pulmonary disease, pulmonary hypertension, or emphysema; diabetes requiring oral medication or insulin; hypertension requiring treatment; known cardiovascular diseases (congestive heart failure of any etiology, documented coronary artery disease, clinically manifest miscellaneous heart disease); symptomatic lung disease on chronic treatment; history of transplantation; obesity (body mass index 30 [calculated as weight in	(a) Randomized<br>(b) Longitudinal<br>(c) Prospective<br>(d) Between June 2 and September 30, 2020<br>(e) Adequate follow-up to observe the outcome of hospitalization	HCQ:<br>0.8 g loading dose, then 0.4 g daily for 9 days<br><br>LPV/r:<br>Loading dose of 0.8g and 0.2g, respectively, every 12 h followed by 0.4 g and 0.1 g, respectively, every 12 h for the next 9 days<br><br>Placebo	(a) Any AE:<br>HCQ: 46 (22.2)c<br>LPV/r: 92 (39.7)c<br>Placebo: 46 (20.9)c<br>Serious AE:<br>HCQ: 11 (5.3)c<br>LPV/r: 20 (8.6)c<br>Placebo: 12 (5.5)c<br>AE leading to withdrawal of drug:<br>HCQ: 1(0.5)c<br>LPV/r: 9(3.9)c<br>Placebo: 3(1.4)c<br>AE leading to study termination:<br>HCQ: 1(0.5)c<br>LPV/r: 0(0)c<br>Placebo: 0(0)c	2 (Jadad)

					kilograms divided by height in meters squared]); immunocompromised status due to disease (eg, those living with HIV with a CD4 T-cell count of <200 cells/mm3 , confirmed malignant neoplasm); immunocompromised status due to medication (eg, people taking 10 mg or more of prednisone equivalents a day); and patients with cancer.<br><br><u>Exclusion criteria:</u><br>The use of any of study drugs in 30 days prior to screening; clinical evidence of progression of COVID-19 (ie, use of oxygen supplementation; arterial oxygen saturation less than 94%; use of noninvasive positive-pressure ventilation support);<br>history of known life threatening cardiac arrhythmias;<br>long QT syndrome;<br>known allergy to study drugs.				
Outcomes of 3,737 COVID-19 patients treated with hydroxychloroquine/azithromycin and other regimens in Marseille, France: A retrospective analysis. Lagier JC. Travel Med	HCQ + AZM for at least 3 days or "Other treatments" (HCQ alone, AZM alone, HCQ+AZM for less than 3 days, and those receiving	3737<br><br><u>Groups</u>:<br>HCQ + AZM ≥ 3 days: 3119<br><br>Other treatments: 618<br>- HCQ: 101<br>- AZM: 137	Outcomes were death, transfer to ICU, hospitalization stay ≥10 days and viral shedding persistence	<u>Age (years):</u><br>45 (17)[b]<br><br><u>Gender (male):</u><br>1,704 (45.6)[c]	<u>Inclusion criteria:</u><br>- >18 years old<br>- PCR-documented SARS-CoV-2 RNA from a nasopharyngeal sample<br><br><u>Exclusion criteria:</u><br>- Patients whose samples were sent to our laboratory but who were followed up outside Marseille<br>- Patients who were managed in Marseille, outside Institut Hospitalo Universitaire<br>- < 18 years old	(a) Observational<br>(b) Longitudinal<br>(c) Retrospective<br>(d) NA<br>(e) From March 3rd to April 27th 2020	<u>HCQ + AZM ≥ 3 days:</u><br>HCQ 0.2 g three times daily for 10 days + AZM 0.5 g on day 1 followed by 0.25 g daily for the next 4 days<br><br><u>HCQ:</u> NA<br><br><u>AZM:</u> NA<br><br><u>HCQ + AZM < 3 days:</u><br>NA<br><br><u>No HCQ, no AZM:</u> NA	(a) <u>AE:</u> observed in 167 (4.5%) patients. All were mild and included mostly gastrointestinal symptoms.<br><u>Discontinuation of treatment:</u> 35 (0.93%) patients, mostly because of gastrointestinal symptoms.<br><u>QTc prolongation</u> (>60 ms): 25 (0.67%) patients, including 2 treated with HCQ (2%), 3 treated with AZM (2.2%) and 20 treated with HCQ+AZM (0.6%). The cessation of treatment for QT prolongation was needed in 12 cases including 3 cases with a QTc ≥500 ms (2 treated with AZM and 1 treated	84 (Strobe)

Infect Dis. 2020	neither HCQ either AZM)	- HCQ + AZM < 3 days: 218<br>- No HCQ, no AZM: 162						with HCQ+AZM).<br>No cases of TdP or sudden death were observed.	
Compassionate use of hydroxychloroquine in clinical practice for patients with mild to severe COVID-19 in a French university hospital. Paccoud O. Clin Infect Dis. 2020	HCQ + SOC or SOC	84<br><br><u>Groups</u>:<br>HCQ + SOC: 38<br><br>SOC: 46	To evaluate the efficacy of HCQ treatment as compared to SOC in patients hospitalized with a diagnosis of COVID-19<br><br>Primary endpoint: time to unfavorable outcome (defined as: death, admission to an ICU, or decision to withdraw or withhold life-sustaining treatments, whichever came first).<br><br>Secondary outcomes: time to death, time to hospital discharge for a return home or in an aftercare and rehabilitation unit, fever and cough at day 5 and AEs recorded in the	<u>Age (years):</u><br>66 (16)[b]<br><br><u>Gender (male):</u><br>62%	<u>Inclusion criteria:</u><br>- All patients hospitalized in the Infectious Diseases ward of the Pitié-Salpêtrière University hospital from January 2020 with a diagnosis of COVID-19<br><br><u>Exclusion criteria:</u><br>- Patients admitted in the Infectious Diseases ward after a stay in an ICU<br>- Patients treated with another experimental treatment<br>- Patients who presented a contraindication to receiving HCQ (QTc interval longer than 440ms on the ECG performed at admission; known hypersensitivity to CQ or HCQ; history of elongated QT interval or severe cardiopathy, G6PD deficiency, or retinopathy; and patients receiving comedications known to elongate the QT interval or potentially responsible for drug-drug interactions that would require close monitoring)	(a) Observational<br>(b) Longitudinal<br>(c) Retrospective<br>(d) Median follow-up of patients was 10 days (CI95% = [10-10])<br>(e) From March 11th 2020	<u>Treatment group:</u><br>HCQ 0.2 g three times a day for 10 days + SOC<br><br><u>Control group:</u><br>SOC: supplemental oxygen therapy in order to maintain an oxygen saturation of >96%, intravenous or oral acetaminophen, and antibiotics if deemed necessary. No AZM.	(a) <u>HCQ</u>: 6 (14)[c] patients reported side effects:<br>- Premature discontinuation of treatment 4 (7)[c]:<br>- QTc interval elongation: 2<br>- Cytopenia: 1<br>- Paresthesia: 1<br>- Headaches: 1<br>- Diarrhea: 1	92 (Strobe)

			patients receiving HCQ						
Experience of short-term hydroxychloroquine and azithromycin in COVID-19 patients and effect on QTc trend. Hor CP. J Infect. 2020	HCQ or HCQ + AZM	13<br><br>Groups: HCQ: 2<br><br>HCQ + AZM: 11	To report risk profile, QTc trend and outcomes of COVID-19 confirmed patients with mild to moderate diseases	Age (years): 52 (27.5, 56.5) [d]<br><br>Gender (male): 53.8%	Inclusion criteria: COVID-19<br><br>Exclusion criteria: NA	(a) Observational<br>(b) Longitudinal<br>(c) Retrospective<br>(d) NA<br>(e) From March 21th to 13th April 2020	HCQ: 0.4 g twice daily the first day followed by 0.2 g twice daily for 5 days, or adjusted based on risk assessment, at the discretion of treating physician<br><br>HCQ + AZM: HCQ 0.4 g twice daily the first day followed by 0.2 g twice daily for 5 days, or adjusted based on risk assessment, at the discretion of treating physician + AZM initiate at 0.5 g followed by 0.25 g daily for5 days	(a) AEs (none of the patients were commenced on other QT-prolonging medications):<br>- QT prolongation: 38.5% whom were profiled as low to intermediate Tisdale and normalized after treatment completion or discontinuation<br>- 2 end stage renal failure patients with elevated baseline QTc intervals had HCQ initiated at 0.2 g twice daily without AZM but<br>were discontinued after 3 days of therapy due to persistent QT prolongation<br>- 1 patient with concomitant sepsis<br>had a rise in QTc interval > 60 ms on day 2 of treatment and persisted<br>despite discontinuation of adjusted lower dose of HCQ and AZM<br>- 0 patients developed cardiac arrhythmia or<br>therapy-related side effects during hospitalization	35 (Strobe)

Enhanced ECG monitoring of COVID-19 patients. Jain S. Heart Rhythm. 2020	HCQ or others	459<br><br><u>Groups</u>:<br>COVID-19 patients with QT prolongation at baseline: 103<br><br>COVID-19 patients without QT prolongation at baseline :356	To establish an enhanced process for ECG monitoring of patients being treated for COVID-19	<u>Age (years):</u><br>68.2 (15.2)[b]<br><br><u>Gender (male):</u><br>64 (62.1)[c]	<u>Inclusion criteria:</u><br>- Patients with a diagnosis of COVID-19 or from a nursing unit designated to care for patients with COVID-19<br><br><u>Exclusion criteria:</u><br>NA	(a) Observational<br>(b) Longitudinal<br>(c) Retrospective<br>(d) A 2-w period<br>(e) From March 28th to April 10th 2020	<u>Medications in patients with COVID-19 and QT prolongation:</u><br><br>- HCQ<br>- HCQ + atazanavir<br>- Tocilizumab<br>- Methylprednisolone<br>- REM<br>- AZM<br>- Nivolumab<br>- Ritonavir/lopinavir<br>- Amiodarone<br>- Proton pump inhibitor<br>- Propofol<br>- Sedative<br>- Selective serotonin reuptake inhibitors<br>- Antipsychotic<br>- Antidepressant<br>- Tacrolimus<br>- Antibiotic<br>- Antiemetic<br>- Other QT-prolonging medications	(a)<br><u>103 COVID-19 patients with QT-prolongation</u>:<br>- HCQ: 98 (95.1)[c]<br>- HCQ + atazanavir: 21 (20.4)[c]<br><br><u>356 COVID-19 patients without QT-prolongation</u>:<br>- HCQ: 317 (89.0%)[c]<br><br><u>Discontinuation of treatment as a result of QT prolongation</u> : 31 (30.1)[c] most commonly HCQ rarely in association with atazanavir or AZM<br><br>Serious clinical arrhythmias were rare<br><br>No episodes of TdP were reported	75 (Strobe)
COVID-19 in solid organ transplant recipients: initial report from the US epicenter. Pereira MR. Am J Transplant. 2020	HCQ or others	90 (68 hospitalized)<br><br><u>Groups</u>:<br>HCQ: 62<br>AZ: 45<br>REM: 2<br>Tocilizumab: 14<br>Bolus steroids: 16<br>Unknown: 2	To present the clinical characteristics of solid organ transplant recipients with COVID-19 at two large academic centers during the initial 3 w of the epidemic in New York City	<u>Age (years):</u><br>57 (46-68)[g]<br><br><u>Gender (male):</u><br>53 (59)[c]	<u>Inclusion criteria:</u><br>- Adult (age >18 years) solid organ transplant recipients with a positive test for SARS-CoV-2 in an inpatient or outpatient setting<br><br><u>Exclusion criteria:</u><br>NA	(a) Observational<br>(b) Longitudinal<br>(c) Retrospective<br>(d) Overall follow up time from positive test until death or last follow-up was 20 (14-24)[g] days<br>(e) From March 13th to April 3th 2020	<u>HCQ:</u> 0.6 g twice daily (load) on day 1, then 0.4 g daily on days 2-5<br><br><u>AZM:</u> 0.5 g once on day 1, then 0.25 g daily on days 2-5<br><br><u>Tocilizumab</u>: one-time dose of either 0.4 g or 8 mg/kg (maximum 0.8 g) IV once, and a second dose was given in select cases<br><br><u>REM</u><br><u>Bolus steroids</u>	(a) There were no adverse reactions reported, including no significant QT interval prolongation in the short term	78 (Strobe)
Experience with	HCQ<br>AZM	98	The outcome of interest was	<u>Age (years):</u><br>62.3 (17.0)[b]	<u>Inclusion criteria:</u>	(a) Observational<br>(b) Longitudinal	<u>HCQ</u>: most patients (87.0%) received 0.4 g	(a) At baseline, the mean QTc (Bazett) was 448±29 ms, and 20% of patients had QTc	86 (Strobe)

hydroxychloroquine and azithromycin in the coronavirus disease 2019 pandemic: implications for QT interval monitoring. Ramireddy A. J Am Heart Assoc. 2020	HCQ + AZM	Groups:<br>HCQ: 10<br>AZM: 27<br>HCQ + AZM: 61	postmedication critical QTc prolongation, defined as follows: (1) maximum postmedication QTc ≥500 ms (if QRS <120 ms) or QTc ≥550 ms (if QRS ≥120 ms) and (2) mean increase from baseline QTc to maximum postmedication QTc of ≥60 ms	Gender (male):<br>60 (61)[c]	- COVID-19–positive/suspected patients who had at least 2 12-lead ECGs performed in our MUSE system between January 1, 2020, and April 5, 2020<br><br>Exclusion criteria:<br>- Patients with paced ventricular rhythms, atrial fibrillation, atrial flutter, supraventricular tachycardia, or ECGs otherwise unsuitable for accurate QT interval measurement<br>- Patients without ECGs performed on day 2 of medication administration or later	(c) Retrospective<br>(d) NA<br>(e) From February 1th to April 4th 2020	orally twice daily before receiving a dosage of 0.2 g orally twice daily on days 2 to 5<br><br>AZM: either 0.5 g daily or 0.5 g on day 1 followed by 0.25 g daily on days 2 to 5. Either oral or IV	≥470 ms. With drug administration, overall QTc (Bazett) increased to 459±36 ms (p 0.005)<br><br>BASELINE ECG INTERVALS:<br>- Ventricular rate:<br>AZM: 102±23 bpm<br>HCQ + AZ: 89±17 bpm<br>p 0.01<br>- RR:<br>AZM: 622±168 ms<br>HCQ + AZ: 701±135 ms<br>p 0.02<br>- QRS:<br>AZM: 94±24 ms<br>HCQ + AZ: 93±17 ms<br>p 0.84<br>- QT:<br>AZM: 364±61 ms<br>HCQ + AZ: 367±37 ms<br>p 0.84<br>- QTc (Bazett):<br>AZM: 463±39 ms<br>HCQ + AZM: 439±20 ms<br>p 0.005<br><br>POSTDRUG ECG:<br>- Ventricular rate:<br>AZM: 92±23 bpm<br>HCQ + AZM: 78±15 bpm<br>p 0.006<br>- RR:<br>AZM: 686±168 ms<br>HCQ + AZM: 794±148 ms<br>p 0.003<br>- QRS:<br>AZM: 93±24 ms<br>HCQ + AZM: 94±18 ms<br>p 0.76<br>- QT:<br>AZM: 383±62 ms	

								HCQ + AZM: 405±43 ms<br>p 0.10<br>- QTc (Bazett):<br>AZM: 464±38 ms<br>HCQ + AZM: 457±38 ms<br>p 0.41<br>- Change in QTc:<br>AZM: 0.5±40.3 ms<br>HCQ + AZM: 17.2±39.0 ms<br>p 0.07<br>- Patients meeting critical QTc threshold (for ≥500 ms (QRS interval <120 ms) or ≥550 ms (QRS interval ≥120 ms)):<br>AZM: 3 (11)[c]<br>HCQ + AZM: 5 (8)[c]<br>p 0.66<br>- Patients with absolute ΔQTc ≥60 ms:<br>AZM: 4 (15)[c]<br>HCQ + AZM: 7 (12)[c]<br>p 0.66<br>- Patients with critical QTc prolongation overall (by QTc threshold or absolute change criteria):<br>AZM: 5 (19)[c]<br>HCQ + AZM: 7 (12)[c]<br>p 0.37<br><br>No patients had syncope, TdP, or other lethal arrhythmias during or after drug administration	
Incidence and determinants of QT interval prolongation in COVID-19 patients treated with hydroxychloroquine and azithromycin. Maraj I. J Cardiovasc	HCQ + AZM	91	Primary outcome: development of significant QTc prolongation defined as increase in baseline QTc 60 ms and/or absolute QTc > 500 ms	Age (years): 62.7 (15.1)[b]<br><br>Gender (female): 44%	Inclusion criteria:<br>- Symptomatic patients who were hospitalized for COVID-19 infection and received treatment with combination HCQ/AZM<br><br>Exclusion criteria:<br>NA	(a) Observational<br>(b) Longitudinal<br>(c) Retrospective<br>(d) NA<br>(e) NA	HCQ + AZM	(a) Excessive QTc prolongation: 23% of patients receiving HCQ/AZM, increasing from 437±37 to 504±41 ms<br><br>QTc >500 ms: 14%<br><br>An additional concurrent QT-prolonging medication was administered in 42% of patients.	59 (Strobe)

Electrophysiol. 2020			Secondary outcomes: ventricular tachyarrhythmias (TdP, polymorphic VT or VF)					Among patients with excessive QT prolongation, a concurrent QT prolonging drug was used in 67% of patients versus only 34% in patients without excessive QT prolongation (p 0.01). Most was due to IV propofol, which was used in 48% of patients with excessive QT prolongation compared to only 19% of patients without (p 0.01).<br><br>Multiple regression demonstrated the use of additional QT prolonging agents (especially propofol) to be independently associated with QTc prolongation [any drug: adjusted OR 3.69, CI (1.22, 11.20), p 0.02; propofol: adjusted OR 3.28, CI (1.06, 10.17), p 0.04].<br><br>2 Significant ventricular arrhythmias:<br>- Classic TdP: 1<br>- Polymorphic VT that degenerated into VF in the setting of severe multisystem disease: 1	
Subcutaneous administration of interferon beta-1a for COVID-19: a non-controlled prospective trial. Dastan F. Int Immunopharmacol. 2020	HCQ + INF-β-1a + LPVr	20	To evaluate the therapeutic effects of INF-β-1a administration in COVID-19	Age (years): 58.55 (37-86)[d]<br><br>Gender (male): 16 (80)[c]	Inclusion criteria:<br>- ≥18 years<br>- Laboratory (reverse transcription polymerase chain reaction)-confirmed COVID-19 infection in the throat swab<br>- Onset of symptoms for <7 days<br>- Severe disease (a respiratory rate of ≥30 breaths/min or an oxygen saturation of ≤90% or a partial pressure of arterial oxygen to percentage of inspired oxygen ratio (PaO2/FiO2) of ≤300 mmHg)<br><br>Exclusion criteria:	(a) Non-controlled trial<br>(b) Longitudinal<br>(c) Prospective<br>(d) 14 days<br>(e) NA	INF-β-1a: 44 µg (equivalent to 12 million international units) subcutaneously every other day up to 10 days<br><br>HCQ: 0.2 g twice daily for 5 days<br><br>LPVr: 0.2/0.05 g two tablets 4 times a day for 5 days	(a) There were no deaths or significant adverse drug reactions in the 14-day period.<br><br>No abnormality was noted in hemoglobin, platelet, urea, creatinine, aspartate transaminase, ALT and alkaline phosphatase levels	1 (Jadad)

					- Pregnant or breast-feeding<br>- Allergic to INF<br>- Had other evidence that could explain the cause for pneumonia such as influenza A virus infection, influenza B virus infection, bacterial pneumonia, or fungal pneumonia or noninfectious causes				

QT interval prolongation under hydroxychloroquine/azithromycin association for inpatients with SARS-CoV-2 lower respiratory tract infection. Bun SS. Clin Pharmacol Ther. 2020	HCQ + AZM	73 (but only 71 received HCQ + AZM)	- To assess the proportion of inpatients (outside the critical care unit) suffering from lower respiratory tract infection, and potentially eligible for a combined therapy HCQ/AZM; according to strict predetermined baseline clinical and ECG criteria<br>- To evaluate QT variation/prolongation 48 h after initiation of HCQ/AZM association in this specific population of SARS-CoV-2 lower respiratory tract infection (safety profile with the use of a dedicated institutional protocol)<br>- To assess the accuracy of automated QTc measurement in patients hospitalized in this specific setting, in comparison	Age (years):<br>62 (14)[b]<br><br>Gender (male):<br>49 (67)[c]	Inclusion criteria:<br>- Patients with baseline QTc ≤ 480ms and potassium level > 4.0 mmol/L.<br><br>Exclusion criteria:<br>- Outpatients	(a) Observational<br>(b) Longitudinal<br>(c) Prospective<br>(d) NA<br>(e) From March 24th to April 20th 2020	HCQ sulfate: 0.2 g three times per day for 10 days<br>+ AZM: 0.5 g on day one, followed by 0.25 g per day for the next 4 days	(a) Baseline average automated QTc was 415 ± 29 ms and lengthened to 438 ± 40 ms after 48 h of combined therapy.<br><br>Patients not eligible for drug initiation (contra-indicated according to ECG and/or structural heart disease): 2 (2.7)[c].<br><br>Treatment stopped because of significant QTc prolongation (≥ 500 ms): 2 (2.8)[c].<br>Concurrent QT-prolonging medication polypharmacy in both patients:<br>- One patient was receiving an association of alimemazine/levomepromazine/zuclopenthixol<br>- One patient was treated with sotalol (interrupted 24 h before HCQ/AZM initiation) for paroxysmal atrial fibrillation. On day two, the patient presented a non-documented syncopal episode, with a significantly prolonged QTc calculated at 503 ms (Bazett's correction).<br><br>No patient (but one) presented syncope, TdP or cardiac arrest under treatment (HCQ/AZM).<br><br>One inpatient experienced a persistent counterclockwise atrial flutter, treated with rate-control agent and anticoagulation. Then the patient was planned for a cavotricuspid isthmus radiofrequency ablation.<br><br>No drug-induced life-threatening arrhythmia, nor death was observed.	70 (Strobe)

			with a manual method of QT measurement					HCQ/AZM could not be initiated or had to be interrupted in less than 6% of the cases.	

Treatment with hydroxychloroquine, azithromycin, and combination in patients hospitalized with COVID-19. Arshad S. Int J Infect Dis. 2020	HCQ, HCQ + AZM, AZM, or other (no HCQ or AZM)	2,541<br><br>Groups:<br>- HCQ: 1202<br>- HCQ + AZM: 783<br>- AZM: 147<br>- Other (no HCQ or AZM): 409	- To assess treatment experience with HCQ versus HCQ + AZM, AZM alone, and other treatments for COVID-19<br><br>- The primary endpoint was in-patient hospital mortality in each treatment group	Age (years): 64 (53–76)[g]<br><br>Gender (male): 51%	Inclusion criteria:<br>- All consecutive patients hospitalized at the Henry Ford Health System in Southeast Michigan being treated for COVID-19<br>- Only the first admission was included for patients with multiple admissions<br>- Diagnosis with SARS-CoV-2 confirmed by a positive reverse-transcriptase polymerase-chain-reaction assay from a nasopharyngeal sample<br>- ≥ 18 years of age<br>- Treated as inpatients for at least 48 h unless they died within the time period<br><br>Exclusion criteria:<br>- Patients who had not been discharged<br>- Patients who left against medical advice<br>- Patients who were transferred to another healthcare facility<br>- Readmissions	(a) Observational<br>(b) Longitudinal<br>(c) Retrospective<br>(d) Follow-up was 28.5 (3–53)[g] days<br>(e) From March 10th to May 2nd 2020	4 medication groups:<br>- HCQ: 0.4 g twice daily for 2 doses on day 1, followed by 0.2 g twice daily on days 2–5<br><br>- AZM: 0.5 g once daily on day 1, followed by 0.25 g once daily for the next 4 days<br><br>- HCQ + AZM: reserved for selected patients with severe COVID-19 and with minimal cardiac risk factors<br><br>- Other: no HCQ or AZM<br><br>The clinical guidelines included adjunctive immunomodulatory therapy with corticosteroids and tocilizumab	(a) Overall in-hospital mortality:18.1% (95% CI:16.6%–19.7%).<br><br>Mortality by treatment:<br>- HCQ+ AZM: 157/783 (20.1% [95% CI: 17.3%–23.0%])<br>- HCQ alone: 162/1202 (13.5% [95% CI: 11.6%–15.5%])<br>- AZM alone: 33/147 (22.4% [95% CI: 16.0%–30.1%])<br>- Neither drug: 108/409 (26.4% [95% CI: 22.2%–31.0%])<br>$p < 0.001$<br>Adjunct therapy with corticosteroids (methylprednisolone and/or prednisone) and anti-IL-6 tocilizumab was provided in 68% and 4.5% of patients, respectively<br><br>Primary cause of mortality (460):<br>- Respiratory failure: 88%<br>- Cardiac arrest (with mean QTc interval from last ECG reading 471 ms): 4%<br>- Other cardiopulmonary arrest and multi-organ failure: 8%<br><br>No patient had documented TdP<br><br>In the multivariable Cox regression model of mortality using the group receiving neither HCQ or AZM as the reference, treatment with HCQ alone decreased the mortality hazard ratio by 66% ($p < 0.001$), and HCQ + AZM decreased the mortality hazard ratio by 71% ($p < 0.001$). Authors did not find statistical significance in the relative effect of adjunct therapy and mortality.	88 (Strobe)

								Treatment with HCQ resulted in a mortality hazard ratio decrease of 51% (p = 0.009). The resulting Kaplan–Meier survival curves within the propensity matched setting displayed significantly better survival in the HCQ treated group, with the enhanced survival persisting all the way out to 28 days from admission	
Hydroxychloroquine plus azithromycin and early hospital admission are beneficial in COVID-19 patients: Turkish experience with real-life data. Tanriverdİ E. Turk J Med Sci. 2021	HCQ, HCQ + AZM or others	83<br><br>Groups:<br>- HCQ + AZM: 26<br>- HCQ + other antibiotics: 30<br>- LPVr: 18<br>- Favipiravir: 9	To evaluate factors affecting the treatment process of the first cases followed in our clinic	Age (years):<br>47.7 (15)[b]<br><br>Gender (male):<br>60 (72.2)[c]	Inclusion criteria:<br>- Consecutive hospitalized patients with COVID-19 pneumonia<br><br>Exclusion criteria:<br>- Patients who were still undergoing inpatient treatment<br>-Patients with positive influenza test results<br>- Patients diagnosed with bacterial infection	(a) Observational<br>(b) Transversal<br>(c) Retrospective<br>(d) NA<br>(e) From March 13th to April 15th 2020	HCQ: 0.4 g twice a day for the first day; 0.2 g twice a day for the following 4 days<br><br>AZM: 0.5 g per day for the first day; 0.25 g per day for the following 4 days<br><br>Favipiravir: 1.6 g loading dose twice a day; 0.6 g twice for the following 4 days or LPVr: 0.4/0.1 g twice a day for 10–14 days were mostly preferred in patients with oxygen saturation <90% on room air, respiration rate over 30 breaths at rest, and rapid clinical and radiological progression of the disease<br><br>Third-generation cephalosporin and quinolone were added as an empiric antibiotic for all patients.<br><br>From March 25th: HCQ + AZM was preferred	The overall mortality was 15%, and 85% of the patients were discharged.<br><br>Unexpected arrhythmia or cardiac events were not observed during hospitalization period<br><br>Mortality:<br>-HCQ + AZM: 5 (19)[b]<br>- HCQ + other antibiotics: 3 (10)[b]<br>p 0.324	69 (Strobe)

A pilot study of hydroxychloroquine in treatment of patients with moderate COVID-19. Chen J. Journal of Zhejiang University. 2020	HCQ	30<br><br>Groups:<br>HCQ + conventional treatment: 15<br><br>Conventional treatment: 15	Efficacy and safety of HCQ	Age (years):<br>50.5(3.8)[b]<br><br>Gender (male):<br>9(60)[c]	Inclusion criteria:<br>- Treatment-naïve patients with confirmed COVID-19<br>18 years old<br><br>Exclusion criteria:<br>-Allergy to CQ/HCQ<br>-Pregnancy<br>-Complicated with severe diseases of the heart, lung, kidney, brain or blood or other important organs<br>-Retinal disease, hearing loss<br>-Severe neurological or mental illness<br>-Those who cannot complete the research	(a) Observational<br>(b) Longitudinal<br>(c) Prospective<br>(d) 2 weeks<br>(e) 6th to 22th February 2020	HCQ + conventional treatment:<br>HCQ 0.4g QD for 5 days	a) Incidence of adverse reactions:<br>No statistically significant differences<br>Transient AST increase:<br>HCQ +conventional treatment: 1(6.66)[c]<br>Conventional treatment: 1(6.66)[c]<br>Elevated serum creatinine:<br>HCQ +conventional treatment: Not observed<br>Conventional treatment: 1(6.66)[c]<br>Diarrhea:<br>HCQ +conventional treatment: 2(13.33)[c]<br>Conventional treatment: Not observed<br>Weakness:<br>HCQ +conventional treatment: 1(6.66)[c]<br>Conventional treatment: Not observed	80 (Strobe)
Clinical efficacy and safety of different antiviral regimens in patients with coronavirus disease 2019. Gao X. Zhonghua Wei Zhong Bing Ji Jiu Yi Xue. 2020	INF-alpha + LPVr + arbidol or rivabirin or CQ	73<br><br>Groups:<br>INF-alpha + LPVr: 47<br><br>INF-alpha + LPVr + arbidol or rivabirin or CQ: 26	Efficacy and short-term and long-term adverse reactions of different antiviral programs	Age (years):<br>40.8(17.0)[b]<br><br>Gender (male): 41 (56.2)[c]	Inclusion criteria:<br>- Diagnostic criteria for new coronary pneumonia<br>- 18 years old<br>- Hospitalization time 5 days<br><br>Exclusion criteria:<br>- Past blood system disease and liver disease<br>- Digestive system symptoms caused by improper diet or other after admission	(a) Observational<br>(b) Longitudinal<br>(c) Retrospective<br>(d) 3-month follow-up<br>(e) 20th January to 15th March 2020	Conventional antiviral group:<br>INF-alpha $5x10^5$ U nebulized BD + LPVr 400/100 mg BD for 7 days<br><br>Unconventional antiviral group:<br>INF-alpha $5x10^5$ U nebulized BD + LPVr 400/100 mg BD for 7 days + arbidol 0.2g TD for 7 days or ribavirin 0.5g BD not exciding 10 days or CQ 0.5 g BD not exciding 10 days	a)Adverse reactions (hospitalization):<br>Abnormal liver function:<br>INF-alpha + LPVr + arbidol or rivabirin or CQ: 16(61.5)[c]<br><br>Dyslipidemia:<br>INF-alpha + LPVr + arbidol or rivabirin or CQ: 11(42.3)[c]<br><br>Diarrhea:<br>INF-alpha + LPVr + arbidol or rivabirin or CQ: 3(11.5)[c]<br><br>Rash:<br>INF-alpha + LPVr + arbidol or rivabirin or CQ: 2(7.7)[c]<br><br>Other:	81.5 (Strobe)

								INF-alpha + LPVr + arbidol or rivabirin or CQ: 0(0)[c]<br><br>No statistically significant differences in ALT, AST and total cholesterol between the two groups.	
Hydroxychloroquine and tocilizumab therapy in COVID-19 patients-An observational study. Ip A. PLoS ONE. 2020	HCQ or HCQ + AZM AZM or Neither HCQ/AZM	2512<br><br>Groups:<br>HCQ: 1914<br><br>(HCQ: n=441 HCQ+AZM n=1473)<br><br>No HCQ: 598	Report our survival outcomes with HCQ and TCZ among hospitalized patients with COVID-19<br><br>To analyze the effect of HCQ in hospitalized patients.<br><br>To investigate the effect of TCZ in the ICU population	Age (years):<br>HCQ: 64 (53-75)[e]<br><br>No HCQ: 66 (52-80)[e]<br><br>Gender (male):<br>HCQ: 1211 (78)[c]<br><br>No HCQ: 354 (22)[c]	Inclusion criteria:<br>-Positive SARS-CoV-2 diagnosis by PCR<br>-Hospitalized within the time frame of March 1, 2020 until May 5, 2020<br>-Non-pregnant<br>-Not on a randomized clinical trial<br>-Did not die during first day of hospitalization<br>-Were not discharged to home within 24 hours	(a) Observational<br>(b) Longitudinal<br>(c) Retrospective<br>(d) Patients still alive in the hospital were censored as of May 5, 2020. Patients who had been discharged from the hospital were censored as of day 36 following hospital admission.<br><br>(e) March 1, 2020 until May 5, 2020	Dosing and duration was at prescriber's discretion.<br><br>HCQ:<br>-The majority of patients received 0.8 g on day 1, and 0.4 g on day 2-5.<br><br>-Median duration: (4-5)[e] days	(a) Discontinuation of HCQ due to prolongation of QTc or arrhythmias in 76 (4)[c] and 33 (2)[c] patients.<br><br>Arrhythmias:<br>HCQ: 101 (5)[c]; no HCQ: 22 (4)[c]<br><br>Cardiomyopathy:<br>HCQ: 20 (1)[c]; no HCQ: 7 (1)[c]<br><br>Deaths were attributed to cardiac causes:<br>HCQ: 89 of 432 (21)[c];<br>no HCQ: 19 of 115 (16)[c]	90<br>(Strobe)

Effect of combination therapy of hydroxychloroquine and azithromycin on mortality in patients with COVID-19.Lauriola M. Clin Transl Sci. 2020	HCQ or HCQ + AZM	377<br><br>Groups:<br>HCQ: 17<br><br>HCQ+AZM: 297<br><br>No treatment: 63	To assess the efficacy of the combination of HCQ plus AZM for hospitalized patients with medium-severe COVID-19	Age (years):<br>HCQ: 76.3 (13.1)[b]<br><br>HCQ+AZM: 70.8 (13.6)[b]<br><br>None: 75.4 (11.9)[b]<br><br>Gender (female):<br>HCQ: 52.9%<br><br>HCQ+AZM: 33.3%<br><br>None: 33.3%	Inclusion criteria:<br>-Adult patients aged at least 18 years<br>-Positive to SARS-CoV-2 PCR testing of oropharyngeal or nasopharyngeal swab specimen<br>-Radiographic evidence of pulmonary infiltrates at computed tomography scan and clinical documentation of lower respiratory symptoms<br>-SpO2 ≤ 94% on room air<br><br>Exclusion criteria:<br>-Presence of contraindications to HCQ or AZM	(a) Observational<br>(b) Longitudinal<br>(c) Retrospective<br>(d) NA<br>(e) February 27, 2020 to the April 20, 2020	Treatment group:<br>HCQ alone: HCQ: 0.2 g t.i.d. for 10 days<br><br>HCQ + AZM: HCQ: 0.2 g t.i.d. and AZM at the dose of 0.5 g q.d. for 10 days	(a) Fatal arrhythmias:<br>HCQ alone: 0<br>HCQ + AZM: 0<br>No treatment: 0	68 (Strobe)
Baricitinib improves respiratory function in patients treated with corticosteroids for SARS-CoV-2 pneumonia: an observational cohort study. Rodriguez-Garcia JL. Rheumatology. 2021	HCQ+LPVr +corticosteroids ± BCT	112<br><br>Groups:<br>HCQ + LPVr + corticosteroids: 50<br><br>HCQ + LPVr + corticosteroids + BCT: 62	To determine whether the JAK inhibitor BCT could offer a beneficial or additive effect to corticosteroids on respiratory function in patients with moderate to severe acute respiratory distress syndrome due to SARS-CoV-2 pneumonia	Age (years):<br>CS: 64 (57-69)[e]<br><br>BCT + CS: 63 (52-72)[e]	Inclusion criteria:<br>-Patients older than 18 years<br>-Admitted to the hospital during observation period with SARS-CoV-2 pneumonia insufficiency[oxygen saturation as measured by pulse oximetry (SpO2)92% breathing room air]<br><br>Exclusion criteria:<br>-Major comorbidities (chronic heart failure, chronic obstructive pulmonary disease on oxygen therapy, obstructive sleep apnoea syndrome with continuous positive airway pressure, advanced chronic kidney disease, active malignancies)<br><br>-Previously treated with other immunomodulators<br>-Admitted to ICU	(a) Observational<br>(b) Longitudinal<br>(c) Prospective<br>(d) NA<br>(e) 15th March to 26th April 2020	Standard treatment for all patients:<br>7-10 days of LVPr 0.2/0.05 g, two tablets/12h and HCQ 0.2 g, a loading dose of two tablets/12 h for the first day followed by one tablet/12h<br><br>Patients with respiratory failure: IVIG, interferon β-1b, methylprednisolone with one or more of the following immunomodulators: BCT, anakinra and/or TCZ<br><br>Treatment group:<br>CS: 3 consecutive days of pulse CS therapy followed by prednisone at a starting dose of 0.03 g/day. Therapy was discontinued by	(a) Interiginous candidiasis in the inguinal region:<br>HCQ + LPVr + corticosteroids: 1 (2)[c]<br>HCQ + LPVr + corticosteroids + BCT: 0<br><br>Oral candidiasis:<br>HCQ + LPVr + corticosteroids: 0<br>HCQ + LPVr + corticosteroids + BCT: 2 (3)[c]<br><br>Intravascular catheter-related methicillin-resistant *S. epidermidis* bacteremia:<br>HCQ + LPVr + corticosteroids: 2 (4)[c]<br>HCQ + LPVr + corticosteroids + BCT: 0<br><br>Co-infection with *S. pneumoniae*:<br>HCQ + LPVr + corticosteroids: 1 (2)[c]<br>HCQ + LPVr + corticosteroids + BCT: 0<br><br>Hyperglycaemic decompensation in non-diabetic patients:<br>HCQ + LPVr + corticosteroids: 5 (10)[c]<br>HCQ + LPVr + corticosteroids + BCT: 5 (8)[c]	89 (Strobe)

					-Patients who died		tapering after 7-10 days of treatment.<br><br><u>BCT + CS:</u> CS for 3 days and then prednisone, combined with BCT for 5-10 days. BCT was administered under two schemes:<br>low-dose BCT: a loading dose of 0.04 g the first day and then 0.02 g daily<br>high-dose BCT: 0.04 g daily each day	<u>Ketoacidotic decompensation in a diabetic patient:</u><br>HCQ + LPVr + corticosteroids: 0<br>HCQ + LPVr + corticosteroids + BCT: 1 (2)[c]<br><br><u>Delirium:</u><br>HCQ + LPVr + corticosteroids: 2 (4)[c]<br>HCQ + LPVr + corticosteroids + BCT: 2 (3)[c]<br><br><u>Acute renal failure:</u><br>HCQ + LPVr + corticosteroids: 1 (2)[c]<br>HCQ + LPVr + corticosteroids + BCT: 0<br><br><u>Anaemia due to a large arm haematoma:</u><br>HCQ + LPVr + corticosteroids: 1 (2)[c]<br>HCQ + LPVr + corticosteroids + BCT: 0<br><br><u>Transient bradycardia associated with prolonged QT interval:</u><br>HCQ + LPVr + corticosteroids: 1 (2)[c]<br>HCQ + LPVr + corticosteroids + BCT: 0<br><br><u>Paroxymal atrial fibrillation:</u><br>HCQ + LPVr + corticosteroids: 2 (4)[c]<br>HCQ + LPVr + corticosteroids + BCT: 0<br><br><u>Third degree atrioventricular block:</u><br>HCQ + LPVr + corticosteroids: 0<br>HCQ + LPVr + corticosteroids + BCT: 1 (2)[c]	

Psychiatric disorders and hydroxychloroquine for coronavirus disease 2019 (COVID-19): a VigiBase study. Garcia P. Drug Saf. 2021	HCQ or Other drugs prescribed	1754 reports<br><br>Psychiatric reports: 73<br><br>HCQ: 56<br><br>Other drugs: 17	To investigate the risk of reporting psychiatric disorders with HCQ compared with other drugs prescribed in COVID-19 patients.<br><br>For completeness, we sought to examine the psychiatric safety profile of HCQ in other indications and before the COVID-19 pandemic	Age (years):<br>For COVID:<br><br>HCQ:<br>54.9 (23-86)[e]<br><br>Other drugs:<br>61.9 (17-85)[e]<br><br>Gener (male):<br>For COVID:<br>HCQ:<br>27(48)[c]<br><br>Other drugs: 7 (41)[c]	Inclusion criteria:<br>-Psychiatric case reports selected using the terminology "Psychiatric disorders" in the System Organ Class view found in MedDRA®, or terms classified as "Depression and suicide/self-injury"(broad) by the Standardized MedDRA® queries.<br>-COVID-19 patients: based on the indication of HCQ (or other drugs), including the terms "COVID-19", "COVID-19 respiratory", "COVID-19 treatment", "Coronavirus disease 2019", "Coronavirus infection" " Coronavirus infection" or "SARS-Cov-2 infection"<br><br>Exclusion criteria (first analysis):<br>-Psychiatric reports not related to a COVID-19 indication.	(a) Observational<br>(b) NA<br>(c) NA<br>(d) NA<br>(e) First analysis: 1 January 2020 to 16 June 2020<br><br>Analysis including only cases reported before the Spanish safety alert: until 10 May 2020<br><br>Second analysis:<br>From 1 January 1983 to 31 december 2019	HCQ: 0.4 g/day in 70% of reports (range 0.2-0.8 g)<br><br>AZM was co-reported in 20 (36)[c] cases<br><br>HCQ Treatment duration: 10 (1-27)[e] days	(a) Psychiatric adverse effects: 56<br><br>-Serious adverse events: 28<br><br>-Complete suicides:<br>4 (between 2 and 4 days after the first HCQ prescription)<br><br>-Intentional self-injury: 3<br><br>-Serious cases of psychotic disorders (mostly associated with visual hallucinations, agitations or aggressions): 12<br><br>-Insomnia or anxiety: 7<br><br>-Confusions: 2<br><br>Non-serious cases-<br>-Insomnia or anxiety: 19<br><br>Intentional self-injury or suicide with remdesivir, tocilizumab or Lp/r: No cases<br><br>Odds ratios for the association between psychiatric disorder reports and the use of HCQ for COVID-19, in Vigibase:<br><br>Analysis including only cases reported before the Spanish safety alert:<br>Other drugs prescribed for COVID-19: 1 (reference)<br>HCQ: 6.27 (2.74-14.35)<br><br>Analysis including all cases reported until 16 june 2020:<br>Other drugs prescribed for COVID-19: 1 (reference)	90 (Strobe)

								HCQ: 2.48 (1.43-4.29)<br><br>Psychiatric reports with HCQ registered in VigiBase until december 2019:<br>-Psychiatric adverse effects: 751<br>-Insomnia or sleep disorders: 237<br>-Anxiety: 166<br>-Depression: 148<br>-Psychotic disorders: 104<br>-Suicide or self-injury: 92<br>-Cognitive disturbances: 74	
The effect of favipiravir on QTc interval in patients hospitalized with coronavirus disease 2019. Çap M. 2020	HCQ or HCQ + FVP	189<br><br>Groups:<br>HCQ: 66<br><br>HCQ + FVP: 66<br><br>FVP only: 57	To investigate any change in the QTc interval in patients hospitalized due to COVID-19 receiving favipiravir therapy	Age (years):<br>HCQ: 43 (29-59)[e]<br><br>HCQ + FVP: 58 (49-70)[e]<br><br>FVP only: 50 (40-67)[e]<br><br>Gender (female):<br>HCQ: 32 (48)[c]<br><br>HCQ + FVP: 32 (48)[c]<br><br>FVP only: 33 (58)[c]	Inclusion criteria:<br>-COVID-19 adult patients<br>A COVID-19 diagnosis constituted a positive finding from RT-PCR of nasal and pharyngeal swab specimens.<br><br>Exclusion criteria:<br>-Negative RT-PCR results<br>-Received AZM therapy<br>-Had a pacemaker rhythm<br>-No ECG recordings	(a) Observational<br>(b) Longitudinal<br>(c) Retrospective<br>(d) NA<br>(e) April 15 and May 31 2020	Treatment group:<br>HCQ: 0.4 g BD followed by 0.2 g bid for 4 days<br><br>FVP: 1.6 g BD followed by 0.6 g bid for 4 days	(a)QTc-AT(Bazett) intervals exceeding 500 ms or a change in the QTc intervals of >60 ms:<br>HCQ: 4 (6)[c]<br>HCQ + FVP: 2 (3)[c]<br><br>Hydroxychloroquine was discontinued in four of these patients.<br><br>TdP:<br>HCQ: 0<br>HCQ + FVP: 0<br><br>Arrhythmic deaths:<br>HCQ: 0<br>HCQ + FVP: 0<br><br>(b) Standard 12-lead ECG. The electrocardiographic analysis was performed by two experienced cardiologists who were blinded to the patient data. The opinion of a third cardiologist was sought in instances where consensus was lacking. The demographic, clinical characteristics, and laboratory parameters during hospitalization were	71 (Strobe)

								obtained from the hospital's electronic medical records.	
Hydroxychloroquine with or without azithromycin in covid-19. Cavalcanti AB. N Engl J Med. 2021	HCQ<br>HCQ + AZM	665<br><br>Groups:<br>HCQ: 221<br><br>HCQ + AZM: 217<br><br>Control: 227	To assess whether HCQ, either alone or in combination with AZM, would be effective in improving clinical status at 15 days after hospital admission due to mild-to-moderate Covid-19	<u>Age (years):</u><br>HCQ: 51.3 (14.5)[b]<br><br>HCQ + AZM: 49.6 (14.2)[b]<br><br>Control: 49.9 (15.1)[b]<br><br><u>Gender (male):</u><br>HCQ: 142 (64.3)[c]<br><br>HCQ + AZM: 123 (56.7)[c]<br><br>Control: 123 (54.2)[c]	<u>Inclusion criteria.</u><br>- ≥ 18 years<br>-Suspected or confirmed Covid-19 with 14 or fewer days since symptom onset<br>-Written or electronic informed consent before randomization<br><br><u>Exclusion criteria:</u><br>-Use of supplemental oxygen a rate > 4 l/min as administered by a nasal cannula or at a level of at least 40% as administered by a Venturi mask<br>-Use of supplemental oxygen administered by a high-flow nasal cannula or invasive or noninvasive ventilation<br>-History of sever ventricular tachycardia or electrocardiographic findings with a corrected QT interval of at least 480 msec	(a) Randomized, open-label, controlled trial<br>(b) Longitudinal<br>(c) Prospective<br>(d) From randomization until day 15<br>(e) NA	<u>Treatment group:</u><br><br><u>HCQ:</u><br>SOC + HCQ 0.4 g twice daily for 7 days<br><br><u>HCQ + AZM:</u> SOC + HCQ 0.4 g twice daily plus AZ 0.5 g once a day for 7 days<br><br><u>Control:</u><br>SOC at the discretion of the treating physicians. The use of glucocorticoids, other immunomodulators, antibiotic agents, and antiviral agents was allowed	(a)<u>Reported serious adverse event:</u><br>HCQ: 2 (1)[c]<br>HCQ + AZM: 5 (2.1)[c]<br>AZ: 0<br>Neither HCQ nor AZM: 2 (1.1)[c]<br><br><u>Risk to life:</u><br>HCQ: 1 (0.5)[c]<br>HCQ+AZM: 1 (0.4)[c]<br>AZM: 0<br>Neither HCQ nor AZM: 0<br><u>Extension of hospitalization:</u><br>HCQ: 0<br>HCQ + AZM: 2 (0.8)[c]<br>AZM: 0<br>Neither HCQ nor AZM: 1 (0.6)[c]<br><br><u>Clinically significant event:</u><br>HCQ: 1 (0.5)[c]<br>HCQ + AZM: 0<br>AZM: 0<br>Neither HCQ nor AZM: 1 (0.6)[c]<br><br><u>Any adverse event:</u><br>HCQ: 67 (33.7)[c]<br>HCQ + AZM: 94 (39.3)[c]<br>AZM: 9 (18)[c]<br>Neither HCQ nor AZM: 40 (22.6)[c]<br><br><u>QTc interval >480 msec within 7 days:</u><br>HCQ: 13/89 (14.6)[c]<br>HCQ + AZM: 17/116 (14.7)[c]<br>AZM: 0/6<br>Neither HCQ nor AZM: 1/58 (1.7)[c]<br><br><u>Arrhythmia:</u><br>HCQ: 3 (1.5)[c]	3 (Jaddad)

								HCQ + AZM: 3 (1.3)c<br>AZ: 0<br>Neither HCQ nor AZM: 1 (0.6)c<br><br>Bradycardia:<br>HCQ: 1 (0.5)c<br>HCQ + AZM: 2 (0.8)c<br>AZ: 0<br>Neither HCQ nor AZM: 1 (0.6)c<br><br>Supraventricular tachycardia:<br>HCQ: 2 (1.0)c<br>HCQ + AZM: 1 (0.4)c<br>AZM: 0<br>Neither HCQ nor AZM: 0<br><br>Ventricular tachycardia:<br>HCQ: 0<br>HCQ + AZM: 0<br>AZ: 0<br>Neither HCQ nor AZ: 0<br><br>Myocardial infarction: HCQ: 0<br>HCQ + AZ: 1 (0.4)c<br>AZM: 0<br>Neither HCQ nor AZM: 0<br><br>Abdominal-wall hemorrhage:<br>HCQ: 0<br>HCQ + AZM: 1 (0.4)c<br>AZM: 0<br>Neither HCQ nor AZM: 0<br><br>Pulmonary embolism:<br>HCQ: 0<br>HCQ + AZM: 2 (0.8)c<br>AZM: 0<br>Neither HCQ nor AZM: 0<br><br>Pneumothorax:	

								HCQ: 1 (0.5)[c]<br>HCQ + AZM: 0<br>AZ: 0<br>Neither HCQ nor AZM: 0<br><br><u>Bronchospasm:</u><br>HCQ: 0<br>HCQ + AZM: 0<br>AZM: 0<br>Neither HCQ nor AZM: 1 (0.6)[c]<br><br><u>Epistaxis:</u><br>HCQ: 0<br>HCQ + AZM: 2 (0.8)[c]<br>AZ: 0<br>Neither HCQ nor AZM: 0<br><br><u>Bloodstream infection:</u><br>HCQ: 1 (0.5)[c]<br>HCQ + AZM: 0<br>AZM: 0<br>Neither HCQ nor AZM:<br><br><u>Itching:</u><br>HCQ: 1 (0.5)[c]<br>HCQ + AZM: 0<br>AZM: 0<br>Neither HCQ nor AZM: 0<br><br><u>Nausea:</u><br>HCQ: 9 (4.5)[c]<br>HCQ + AZM: 6 (2.5)[c]<br>AZM: 0<br>Neither HCQ nor AZM: 2 (1.1)[c]<br><br><u>Vomiting:</u><br>HCQ: 0<br>HCQ + AZM: 0<br>AZ: 0<br>Neither HCQ nor AZM: 1 (0.6)[c]	

								<u>Anemia:</u><br>HCQ: 14 (7)[c]<br>HCQ + AZM: 23 (9.6)[c]<br>AZM: 5 (10)[c]<br>Neither HCQ nor AZM: 11 (6.2)[c]<br><br><u>Elevated ALT or AST level:</u><br>HCQ: 17 (8.5)[c]<br>HCQ + AZM: 26 (10.9)[c]<br>AZM: 2 (4)[c]<br>Neither HCQ nor AZM: 6 (3.4)[c]<br><br><u>Hypoglycemia:</u><br>HCQ: 1 (0.5)[c]<br>HCQ + AZM: 0<br>AZM: 0<br>Neither HCQ nor AZM: 0<br><br><u>Elevated bilirubin level:</u><br>HCQ: 5 (2.5)[c]<br>HCQ + AZM: 1 (0.4)[c]<br>AZM: 0<br>Neither HCQ nor AZM: 2 (1.1)[c]<br><br><u>Leukopenia:</u><br>HCQ: 3 (1.5)[c]<br>HCQ + AZM: 6 (2.5)[c]<br>AZM: 2 (4)[c]<br>Neither HCQ nor AZM: 3 (1.7)[c]<br><br><u>Low lymphocyte level:</u><br>HCQ: 17 (8.5)[c]<br>HCQ + AZM: 29 (12.1)[c]<br>AZ: 2 (4)[c]<br>Neither HCQ nor AZM: 16 (9)[c]<br><br><u>Thrombocytopenia:</u><br>HCQ: 14 (7)[c]<br>HCQ + AZM: 17 (7.1)[c]	

								AZM: 1 (2)[c]<br>Neither HCQ nor AZM: 18 (10.2)[c]<br><br>Hypoacusis:<br>HCQ: 0<br>HCQ + AZM: 0<br>AZM: 0<br>Neither HCQ nor AZM: 0	
Hydroxychloroquine/azithromycin treatment, QT interval and ventricular arrhythmias in hospitalized patients with COVID-19. Özdemir IH. Int J Clin Pract. 2021	HCQ<br>HCQ+AZM	101	To assess the implications of HCQ and HCQ + AZM treatment regimens on QTc interval and malignant ventricular arrhythmias in hospitalized patients with COVID-19	Age (years):<br>HCQ: 46 (16)[b]<br><br>HCQ+AZM: 53.5 (19)[b]<br><br>Gender (male): HCQ: 29 (64.4)[c]<br><br>HCQ+AZM: 26 (46.4)[c]	Inclusion criteria:<br>-Hospitalized adults diagnosed with COVID-19 by PCR<br><br>Exclusion criteria:<br>-Patients with QRS width ≥ 120 ms before treatment<br>-Left bundle branch block<br>-Right bundle branch block<br>-Pre-excitation syndromes<br>-With implantable-cardioverter defibrillator or cardioverter defibrillator or cardiac resynchronization therapy<br>-Cardiac pacemaker<br>-Pregnants<br>-Patients who have to use other drugs that may cause QTc prolongation<br>-Patients requiring intensive care unit	(a) Observational<br>(b) Longitudinal<br>(c) Retrospective<br>(d) The maximum follow-up time was 7 days. ECG follow-up time was 5 days for all participants<br>(e) March 20, 2020 and April 20, 2020	Treatment group:<br><br>HCQ: HCQ loading dose 2x0.4 g, maintenance dose 2x0.2 g for five days<br><br>HCQ + AZ:<br>HCQ loading dose 2x0.4 g, maintenance dose 2x0.2 g in addition to AZ loading dose 1x0.5 g, maintenance dose 1x0.25 g for five days	(a) New-onset atrial fibrillation:<br>HCQ: 0<br>HCQ + AZM: 0<br><br>Sinus bradycardia:<br>HCQ: 5 (11.1)[c]<br>HCQ + AZM: 7 (12.5)[c]<br><br>Non-sustained ventricular tachycardia:<br>HCQ: 0<br>HCQ + AZM: 0<br><br>Sustained ventricular tachycardia:<br>HCQ: 0<br>HCQ + AZM: 0<br><br>TdP/ventricular fibrillation:<br>HCQ: 0<br>HCQ + AZM: 0<br><br>In-hospital mortality secondary to ventricular arrhythmia:<br>HCQ: 0<br>HCQ + AZM: 0	68 (Strobe)

Absence of relevant QT interval prolongation in not critically ill COVID-19 patients. Jiménez-Jáimez J. Sci Rep. 2020	HCQ and/or AZM or HCQ + AZM + ART	219<br><br>Groups: HCQ and/or AZM: 105<br><br>HCQ + AZM + ART: 114	To determine the prevalence of clinically significant QT interval prolongation under medical therapy for COVID-19, especially QT greater than 500 ms involving a higher arrhythmic risk.<br><br>To detect clinical or analytical independent predictors of QT interval prolongation and quantify the QTc interval increasing from basal ECGs	<u>Age (years):</u><br>HCQ, and/or AZM : 67.1 (19.3)[b]<br><br>HCQ, + AZM + ART: 60.3 (14.7)[b]<br><br><u>Gender (male):</u><br>HCQ and/or AZM: 46 (43.8)c<br><br>HCQ + AZM + ART: 66 (57.9)	<u>Inclusion criteria:</u><br>-Patients presenting to our emergency department with confirmed diagnosis or high clinical suspicion of SARS-CoV-2 infection and treated whether an inpatient or outpatient management<br><br><u>Exclusion criteria:</u><br>-Patients readmitted for SARS-Cov-2<br>-Admitted to the Intensive Care Unit (ICU) or with mechanical ventilation at the time of baseline and control ECG<br>-Patients with generalized flat T waves and not reliable ECG recordings	(a) Observational<br>(b) Transversal<br>(c) NA<br>(d) Twelve lead ECG was performed in every patient 48 h after treatment initiation. Most of the patients had a basal ECG collected before the beginning of medical therapy at the ED admission.<br>(e) March 23 and April 24 2020	Combination of two or more of the following agents:<br><br>HCQ 0.4 g BID (loading dose) and 0.2 g BID during 5 days<br><br>AZM 0.5 g SID (loading dose) and 0.25 g SID during 5 days<br><br>LVPr 0.4/0.1 g BID or DVRr 0.6/0.1 g BID during 7 to 14 days at physician criteria based on severity illness	31 (14)[c] presented QTc> 460ms after treatment:<br><br>(a) <u>TdP:</u><br>HCQ, and/or AZM: 0<br>HCQ + AZM + ART: 0<br><br><u>Arrhythmic death:</u><br>HCQ, and/or AZM: 0<br>HCQ + AZM + ART: 0	76 (Strobe)
Effect of hydroxychloroquine, azithromycin and lopinavir/ritonavir on the QT corrected interval in patients with COVID-19. Echarte-Morales J. J Electrocardiol. 2021	HCQ + AZM or HCQ + AZM + LPVr	168<br><br>Groups: HCQ + AZM: 54<br><br>HCQ + AZM + LPVr: 114	To evaluate the risk of prolonged QTc and ventricular arrhythmias in COVID-19 patients receiving triple therapy with LPVr on top of HCQ and AZM compared to those treated with HCQ and AZM alone	<u>Age (years):</u><br>HCQ + AZM: 68.7 (18.4)[b]<br><br>HCQ + AZM + LPVr: 65 (12.8)[b]<br><br><u>Gender (male):</u><br>HCQ + AZM: 32 (59.3)[c]<br><br>HCQ + AZM + LPVr: 66 (59.5)[c]	<u>Inclusion criteria:</u><br>-All consecutive patients admitted in our institution<br>-Diagnosis of COVID-19<br><br><u>Exclusion criteria:</u><br>-Non-confirmed test for COVID-19<br>-No ECG at baseline and at least one ECG during follow up<br>-Did not receive at least one dose of either prespecified combined therapy	(a) Observational<br>(b) Longitudinal<br>(c) Prospective<br>(d) ECG was performed prior to the initiation of medical therapy and at 48 and 96 h<br>(e) March 31st and May 8th	The choice of treatment in each patient was based on physician's criteria and local guidelines.<br><br>Standard prescription of the 3 analyzed drugs: 0.4 g HCQ twice on day 1 and 0.2 g twice a day on days 2 to<br>0.5 g of AZM once on day 1 and 0.25 g daily for 4 days more<br>02/0.05 g of LPVr twice a day during two weeks	(a) <u>Bundle branch block:</u><br>HCQ + AZM: 5 (9.3)[c]; HCQ + AZM + LPVr: 13 (11.4)[c]	76 (Strobe)

HyPE study: hydroxychloroquine propylaxis-related adverse events' analysis among healthcare, workers during COVID-19 pandemic: a rising public health concern. Nagaraja BS. J Public Health. 2020	HCQ or HCQ+AZM	166<br><br>Other prophylaxis practices: 10 (6)[c]<br><br>HCQ + AZM: 7 (4.2)[c]<br><br>-Non-allopathy users: 3 (1.8)[c]	Safety profile of the drug in asymptomatic population	Age (years): 36.3 (11.8)[b]<br><br>Gender (male): 122 (73.5)[c]	Inclusion criteria:<br>-Healthcare workers involved in COVID-19 -related services<br>-Have taken at least one dose of hydroxychloroquine<br>-Were either negative for COVID-19 RT-PCR test or not tested<br><br>Exclusion criteria:<br>-Tested positive for COVID-19 rt-PCR<br>-Report of COVID-19 test was awaited<br>-Had symptoms of pre-existing disease in the last 4 weeks prior to the first dose of hydroxychloroquine or in case of any recent change in dose of chronic medications or addition of new medications.	(a) Observational<br>(b) Transversal<br>(c) NA<br>(d) NA<br>(e) 22nd April to 27th April 2020	Cumulative dose:<br><1 g: 43 (25.9)[c]<br>1-2 g: 108 (65.1)[c]<br>>2 g: 15 (9)[c]<br><br>Duration of prophylaxis taken:<br>1st week: 43 (25.9)[c]<br>2nd week: 20 (12)[c]<br>3rd week: 50 (30.1)[c]<br>4th week: 38 (22.9)[c]<br>≥ 5 th week: 15 (9)[c]	(a) HCQ:<br><br>Gastrointestinal:<br>HCQ: 51 (30.7)[c]<br>HCQ + AZM: 2 of 7<br><br>-Abdominal pain:<br>HCQ: 12 (7.22)[c]<br><br>-Nausea:<br>HCQ: 17 (10.24)[c]<br><br>-Vomiting:<br>HCQ: 2 (1.20%)[c]<br><br>-Diarrhea:<br>HCQ: 12 (7.22)[c]<br><br>-Decreased appetite:<br>HCQ: 8 (4.80)[c];<br><br>Cardiovascular:<br>HCQ: 6 (3.6)[c]<br><br>-Palpitations:<br>HCQ: 4 (2.4)[c]<br>Three of them had the symptoms with first dose of the drug<br><br>-Chest pain:<br>HCQ: 2 (1.20)[c]<br><br>Psychiatric:<br>HCQ: 8 (4.8)[c]<br><br>-Nightmare:<br>HCQ: 1 (0.6)c<br><br>-Hypersomnolence:<br>HCQ: 4 (2.40)[c]	68 (Strobe)

								-Nervousness:<br>HCQ: 2 (1.20)[c]<br><br>-Anxiety:<br>HCQ: 1 (0.6)[c]<br><br>Respiratory:<br>HCQ: 1 (0.6)[c]<br><br>-Shortness of breath:<br>HCQ: 1 (0.6)[c]<br><br>Neurological:<br>HCQ: 19 (11.4)[c]<br><br>-Headache:<br>HCQ: 10 (6)[c]<br><br>-Abnormal movements with extra-pyramidal symptoms:<br>HCQ: 2 (1.20)[c]<br><br>-Tinnitus:<br>HCQ: 1 (0.6)[c]<br><br>-Dizziness:<br>HCQ: 6 (3.6)[c]<br><br>Dermatological:<br>HCQ: 6 (3.6)[c]<br><br>-Hair fall:<br>HCQ: 3 (1.8)[c]<br><br>-Oral ulcer:<br>HCQ: 2 (1.2)[c]<br><br>-Itching:<br>HCQ: 1 (0.6)[c]	

								Ophthalmological: 4 (2.4)c<br><br>-Transient visual blurring:<br>HCQ: 4 (2.4)[c]<br><br>-Non-specific:<br>HCQ: 27 (16.2)[c]<br>HCQ + AZM: 1 of 7<br><br>-Fatigue, leathergy, weakness:<br>HCQ: 12 (7.2)[c]<br><br>-Lightheadedness:<br>HCQ: 12 (7.2)[c]<br><br>-Excessive sweating:<br>HCQ: 2 (1.2)[c]<br><br>-Weight loss:<br>HCQ: 1 (0.6)[c]<br><br>Non-allopathy users :<br>Diarrhea, hair-fall and abnormal movement: 1 of 3	

Effect of hydroxychloroquine in hospitalized patients with COVID-19. RECOVERY Collaborative Group. N Engl J Med. 2020	HCQ sulfate ± AZM or usual care ± AZM	4716<br><br>Groups:<br>HCQ± AZM: 1561<br><br>Usual care ± AZM: 3155	Evaluate the effects of potential treatments in patients hospitalized with COVID-19.	Age (years):<br>65.4(15.3)[b]<br><br>Gender (male):<br>62%	Inclusion criteria:<br>-Clinically-suspected or laboratory-confirmed SARS-CoV-2 infection<br>-No medical history that might, in the opinion of the attending clinician, put patients at substantial risk if they were to participate in the trial.	(a) Randomized, open-label, controlled<br>(b) Longitudinal<br>(c) Prospective<br>(d)28 days<br>(e) 15th to 5th June 2020	Treatment group:<br>HCQ 0.8g at baseline and at 6 h, which was followed by 0.4g starting at 12 h after the initial dose and then every 12 h for the next 9 days or until discharge.<br><br>Control group:<br>Usual care	a)Data on new major cardiac arrhythmia collected for 47.1% (HCQ group) and 45.0% (usual care group) of patients:<br><br>Supraventricular tachycardia:<br>HCQ± AZM: 7.6%<br>Usual care± AZM: 6.0%<br>No significant differences<br><br>Ventricular tachycardia or fibrillation:<br>HCQ± AZM: 0.7%<br>Usual care± AZM: 0.4%<br>No significant differences<br><br>Atrioventricular block requiring intervention:<br>HCQ± AZM: 0.1%<br>Usual care± AZM: 0.1%<br>No significant differences<br><br>TdP:<br>HCQ± AZM: 1(0.064)[c]<br>Usual care± AZM: 0 (0.0)[c]<br>No significant differences	3 (Jadad)
QTc prolongation among hydroxychloroquine sulfate-treated COVID-19 patients: An observational study. Fteiha B. Int J Clin Pract. 2021	HCQ sulfate ± AZM	90	ECG changes potentially attributable to HCQ among moderate and severely ill COVID-19 patients	Age (years):<br>65(55-75)[e]<br><br>Gender (male):<br>57(63)[c]	Inclusion criteria:<br>-Manual verification of a positive PCR nasopharyngeal swab specimen tested for SARS-CoV-2 and receipt >2days of HCQ and had at least one ECG after treatment initiation	(a) Observational<br>(b) Longitudinal<br>(c) NA<br>(d) Hospitalization<br>(e) 1st Mar to 14th Apr 2020	HCQ 0.4mg BD for 1 day followed by 0.2g BD for 5 to 10 days 50% reduced if creatinine clearance less than 30ml/min)	a) QTc prolonged ≥500 ms:<br>HCQ± AZM: 7(7.8)[c]<br>QTc change ≥60 ms:<br>HCQ± AZM: 11(12)[c]	91.6 (Strobe)

Effect of hydroxychloroquine on clinical status at 14 days in hospitalized patients with COVID-19: A Randomized Clinical Trial. Self WH. JAMA. 2020	HCQ ± AZM ± REM ± corticosteroids	479<br><br>Groups:<br>HCQ: 242<br><br>Placebo: 237	Determine whether HCQ is an efficacious treatment for adults hospitalized with COVID-19	Age (years):<br>57(44-68)[e]<br><br>Gender (female):<br>44.3%	Inclusion criteria:<br>-Adult patient hospitalized with respiratory symptoms from severe acute respiratory syndrome coronavirus 2 infection	(a) Randomized, placebo-controlled<br>(b) Longitudinal<br>(c) Prospective<br>(d) 28 days<br>(e) 2nd April to 19th June 2020	Treatment group:<br>HCQ 0.4g BD for 2 doses, and 0.2g BD for 8 doses.<br><br>Control group:<br>Placebo	a) QTc prolonged ≥500 ms:<br>HCQ± AZM±REM± corticosteroids : 13(5.9)[c]<br>Placebo: 7(3.3)[c]<br>AST or ALT ≥ 2 times upper limit of normal:<br>HCQ± AZM±REM± corticosteroids : 50(20.7)[c]<br>Placebo: 65(27.4)[c]<br>Cardiac arrest treated with CPR:<br>HCQ± AZM±REM± corticosteroids : 10(4.1)[c]<br>Placebo: 4(1.7)[c]<br>Symptomatic hypoglycemia:<br>HCQ± AZM±REM± corticosteroids : 10(4.1)[c]<br>Placebo: 8(3.4)[c]<br>Ventricular tachyarrhythmia:<br>HCQ± AZM±REM± corticosteroids : 5(2.1)[c]<br>Placebo: 6(2.5)[c]<br>Seizure:<br>HCQ± AZM±REM± corticosteroids : 1(0.4)[c]<br>Placebo: 0	5 (Jadad)

Safety of hydroxychloroquine and darunavir or lopinavir in COVID-19 infection. Meriglier E. J Antimicrob Chemother. 2021	HCQ + DRVr or LPVr	46<br><br>Groups:<br>HCQ + LPVr: 21<br><br>HCQ + DRVr: 25	Safety of combination therapy with HCQ and DRVr or LPVr	Age (years):<br>68(57-81)[e]<br><br>Gender (male):<br>25(54)[c]	Inclusion criteria:<br>-Confirmed COVID-19 infection hospitalized with specific conditions	(a) Observational<br>(b) Longitudinal<br>(c) NA<br>(d) NA<br>(e) 15th Mar to 30th Apr 2020	HCQ 0.4g BD for 1 day followed by 0.4g morning and 0.2g evening if weight ≥ 60kg or 0.2g BD if weight < 60kg from day 2 to 7 plus<br>LPVr 0.4/0.1g BD or DRV/r 0.8/0.1g QD from day 1 to 7	a)<br>ECG abnormalities:<br>HCQ+DRVr: 4(16.0)[c]<br>HCQ+LPVr: 4(19.0)[c]<br>Repolarization disorder:<br>HCQ+DRVr: 3(12)[c]<br>HCQ+LPVr: 3(14.2)[c]<br>Conduction disorder:<br>HCQ+DRVr: 0(0)[c]<br>HCQ+LPVr: 1(4.76)[c]<br>Repolarization and conduction disorder:<br>HCQ+DRVr: 1(4)[c]<br>HCQ+LPVr: 0(0)[c]<br>Bradycardia:<br>HCQ+DRVr: 0(0)[c]<br>HCQ+LPVr: 0(0)[c]<br>Diarrhoea:<br>HCQ+DRVr: 8(32)[c]<br>HCQ+LPVr: 5(23.8)[c]<br>Nausea:<br>HCQ+DRVr: 0(0)[c]<br>HCQ+LPVr: 2(9.52)[c]<br>Hypoglycaemia:<br>HCQ+DRVr: 0(0)[c]<br>HCQ+LPVr: 0(0)[c]<br>Rash:<br>HCQ+DRVr: 0(0)[c]<br>HCQ+LPVr: 0(0)[c]<br>Headache:<br>HCQ+DRVr: 0(0)[c]<br>HCQ+LPVr: 0(0)[c]<br>Hepatic enzymes increased:<br>HCQ+DRVr: 1(4)[cc]<br>HCQ+LPVr: 0(0)[c]	88.7 (Strobe)

A multicenter, randomized, open-label, controlled trial to evaluate the efficacy and tolerability of hydroxychloroquine and a retrospective study in adult patients with mild to moderate coronavirus disease 2019 (COVID-19. Chen CP. PLoS One. 2020	HCQ ± AZM ± OSMV ± LEV	33<br><br><u>Groups:</u><br>HCQ: 21<br>SOC: 12	Efficacy of HCQ against COVID-19	<u>Age (years):</u><br>32.9(10.7)[b]<br><br><u>Gender (male):</u><br>57.6%	<u>Inclusion criteria:</u><br>-Confirmed positive for SARS-CoV-2 infection by real time RT-PCR<br><br><u>Exclusion criteria:</u><br>-Severe illness with respiratory distress, oxygen supplementation, and evidence of infiltration according to chest X-ray<br>-History of hypersensitivity to quinine derivates<br>-Retinal disease<br>-Hearing loss<br>-Severe neurological or mental illness<br>-Pancreatitis<br>-Lung disease<br>-Liver disease (ALT/AST >3x the normal upper limit)<br>-Kidney disease<br>-Hematological disease<br>-Cardiac conduction abnormalities<br>-Known HIV infection<br>-Active hepatitis B or C<br>-G6DP<br>-Psychiatric disorders and alcohol/substance dependence /abuse<br>-Pregnant or breast-feeding	(a) Randomized, open label<br>(b) Longitudinal<br>(c) Prospective<br>(d) 14 days<br>(e) 1st April to 31th May 2020	HCQ 0.4g BD on day 1 followed by 0.2g BD for 6 days.	a)<br><u>Headache:</u><br>HCQ±AZM±OSMV±LEV: 21.1%<br><u>Dizziness:</u><br>HCQ±AZM±OSMV±LEV: 5.3%<br><u>Gastritis:</u><br>HCQ±AZM±OSMV±LEV: 5.3%<br><u>Diarrhea:</u><br>HCQ±AZM±OSMV±LEV: 5.3%<br><u>Nausea:</u><br>HCQ±AZM±OSMV±LEV: 5.3%<br><u>Photophobia:</u><br>HCQ±AZM±OSMV±LEV: 5.3%<br><u>Severe QT prolongation:</u><br>HCQ±AZM±OSMV±LEV: 0%	3 (Jadad)

Hydroxychloroquine as postexposure prophylaxis to prevent severe acute respiratory syndrome coronavirus 2 infection. Barnabas RV. Ann Intern Med. 2021	HCQ	407	Test HCQ as postexposure prophylaxis for SARS-CoV-2 infection	Age (years):<br>40 (27-51)[e]<br><br>Gender (male):<br>138(39)[c]	Inclusion criteria:<br>-Participants were able to provide informed consent<br>-18 to 20 years old<br>-Close contacts with a person with recent known SARS-CoV-2 infection<br>-Exposure within the prior 96 hours<br>-Able to conduct study visits via telehealth<br>-Not planning to take HCQ outside the study<br><br>Exclusion criteria:<br>-Symptoms consistent with SARS-CoV-2 infection<br>-Medical contraindication to HCQ	(a) Randomized, double-blind, controlled trial<br>(b) Longitudinal<br>(c) Prospective<br>(d) 14 days<br>(e) 31th March to 21th August 2020	HCQ 0.4g QD for 3 days followed by 0.2g QD for 11 days	a)<br>Diarrhea, abdominal discomfort or vomiting:<br>25(6.1)[c]<br>Nausea or upset stomach:<br>14(3.4)[c]<br>Skin reaction/rash:<br>11(2.7)[c]<br>Neurologic reaction: irritability, dizziness or vertigo:<br>6(1.5)[c]<br>Headache:<br>5(1.2)[c]<br>Hot flashes, night sweats, or palpitations:<br>2(0.5)[c]<br>Taste change or dry mouth:<br>1(0.2)[c]<br>Fatigue:<br>4(1.0)[c]<br>Visual changes:<br>4(1.0)[c]	5 (Jadad)

								Allergic reaction:<br>2(0.5)[c]<br>Tinnitus:<br>0(0)[c]	
A cluster-randomized trial of hydroxychloroquine for prevention of covid-19. Mitjà O. N Engl J Med. 2021	HCQ	1116	Efficacy and safety of HCQ to prevent secondary PCR-confirmed, symptomatic COVID-19 and SARS-CoV-2 infection in contacts exposed to a PCR-positive case patient with COVID-19.	Age (years):<br>48.6(18.7)[b]<br><br>Gender (female):<br>813(72.8)[c]	Inclusion criteria:<br>-Asymptomatic adults (≥18 years)<br>-Recent history of close-contact exposure to a COVID-19 PCR-confirmed case patient<br>-No COVID-19 symptoms 2 weeks before enrollment<br>-Increased risk of infection (e.g. health care and nursing-home workers, household contacts and nursing home residents)<br>-Negative or positive PCR test at baseline	(a) Randomized, open-label<br>(b) Longitudinal<br>(c) Prospective<br>(d) 28 days<br>(e) 17th March to 28th April 2020	HCQ 0.8g once, followed by 0.4g QD for 6 days	a)<br>Cardiac disorder: Palpitations:<br>5(0.4)[c]<br>Gastrointestinal disorder: diarrhea, abdominal pain, vomiting:<br>510(42.6)[c]<br>Nervous system disorder: headache, taste change, dizziness:<br>260(21.7)[c]<br>General disorder: myalgia, fatigue, malise:<br>103(8.6)[c]	3 (Jadad)
Investigation of QT prolongation with hydroxychloroquine and azithromycin for the treatment of COVID-19. Seyhan AU. J Coll Physicians Surg Pak. 2020	HCQ ± AZM	144<br><br>HCQ: 51<br><br>HCQ+AZM: 93	Assess and identify the risk of prolonged QT about HCQ and AZM used in the treatment of patients with COVID-19	Age (years):<br>55.81 (19.32)[b]<br><br>Gender (female):<br>48.6%	Inclusion criteria:<br>-Hospitalized COVID-19 patients confirmed by RT-PCR<br>-A baseline ECG and at least one ECG after treatment<br><br>Exclusion criteria:<br>-Patients with missing data	(a) Observational<br>(b) Longitudinal<br>(c) Retrospective<br>(d) NA<br>(e) March to May 2020	HCQ 0.4g BD on day 1 followed by 0.2g BD for 4 days<br>AZM 250 mg QD for 5 days	a)<br>Ventricular Arrhythmia:<br>-HCQ: 0(0)[c]<br>-HCQ+AZM: 0(0)[c]<br>QTc change >60 ms:<br>-HCQ: 1(1.96)[c]<br>-HCQ+AZM: 2(2.15)[c]<br>QTc ≥500ms:<br>-HCQ: 1(1.96)[c]<br>-HCQ+AZM: 1(1.07)[c]	94.2 (Strobe)

Safety and efficacy of hydroxychloroquine in 152 outpatients with confirmed COVID-19: A pilot observational study. Sogut O. Am J Emerg Med. 2021	HCQ	152	Efficacy and safety of HCQ empirical treatment of outpatients with confirmed COVID-19	Age (years):<br>47.0 (36.2-62)[e]<br><br>Gender (male):<br>78(51.31)[c]	Inclusion criteria:<br>-Adult patient (≥18 years) with complaints of fever and/or cough, and shortness of breath, admitted to the emergency department<br>-Confirmed COVID-19 by molecular assay<br>-Identified for ambulatory follow-up and treatment<br><br>Exclusion criteria:<br>-Allergy to HCQ or CQ<br>-Contraindication to study drug (retinopathy, history of long QT syndrome, and QT interval prolongation)<br>-Tisdale risk score ≥11<br>-Breastfeeding and pregnant	(a) Observational<br>(b) Longitudinal<br>(c) Prospective<br>(d) 2 weeks<br>(e) 16th March to 15th August 2020	HCQ 0.4g BD on day 1 followed by 0.2g BD for 4 days	a)<br>QT interval prolongation:<br>98(64.5)[c]<br>QT interval shortened:<br>54(35.5)[c]<br>QTc change ≥30ms:<br>22(14.5)[c]<br>QTc change ≥60ms:<br>0(0)[c]<br>QTc >500ms:<br>0(0)[c]<br>Gastrointestinal symptoms (e.g. diarrhea, non-specific abdominal pain, dyspepsia, bloating, nausea, vomiting):<br>34(22.3)[c]<br>Headache and dizziness:<br>25(16.4)[c]<br>Itching and redness:<br>4(2.6)[c]<br>Anaphylaxis:<br>0(0)[c]<br>Malignant cardiac arrhythmia:<br>0(0)[c]	86.2 (Strobe)
A prospective, observational study to evaluate adverse drug reactions in patients with COVID-19 treated with remdesivir or hydroxychloroquine: a preliminary report. Falcao F. Eur J Hosp Pharm. 2020	HCQ ± AZM ± LPVr	101<br><br>HCQ: 20<br><br>HCQ+AZM: 52<br><br>HCQ+LPVr: 22<br><br>HCQ+AZM+LPVr: 7	Adverse drug reactions attributed to either REM or HCQ in patients hospitalized for COVID-19	Age (years):<br>65.5 (15.7)[b]<br><br>Gender (male):<br>56,4%	Inclusion criteria:<br>-Positive RT-PCR for SARS-CoV-2<br>-Treated with either REM or HCQ<br><br>Exclusion criteria:<br>-HCQ or LPVr prescribed for labeled indicationstal	(a) Observational<br>(b) Longitudinal<br>(c) Prospective<br>(d) Treatment duration<br>(e) 25th April to 30th May 2020	0.4 g on day one followed by 0.2 g BD for 5-10 days.	a)<br>Transaminase increase:<br>HCQ: 2(10)[c]<br>HCQ+AZM: 2(3.84)[c]<br>HCQ+LPVr: 12(54.54)[c]<br>HCQ+AZM+LPVr: 3(42.8)[c]<br>Bilirubin increase:<br>HCQ: 0(0)[c]<br>HCQ+AZ: 0(0)[c]<br>HCQ+LPVr: 3(13.6)[c]<br>HCQ+AZM+LPVr: 1(14.3)[c]<br>GGT increase:<br>HCQ: 0(0)[c]<br>HCQ+AZM: 1(1.9)[c]<br>HCQ+LPVr: 3(13.6)[c]<br>HCQ+AZM+LPVr: 1(14.2)[c]	78.6 (Strobe)

								Liver cholestasis:<br>HCQ: 0(0)[c]<br>HCQ+AZM: 4(7.7)[c]<br>HCQ+LPVr: 1(4.5)[c]<br>HCQ+AZM+LPVr: 1(14.2)[c]<br>Hepatotoxicity:<br>HCQ: 0(0)[c]<br>HCQ+AZM: 4(7.7)[c]<br>HCQ+LPVr: 0(0)[c]<br>HCQ+AZM+LPVr: 1(14.2)[c]<br>Diarrhoea:<br>HCQ: 1(5)[c]<br>HCQ+AZM: 1(1.9)[c]<br>HCQ+LPVr: 9(40.9)[c]<br>HCQ+AZM+LPVr: 5(71.4)[c]<br>Nausea:<br>HCQ: 2(10)[c]<br>HCQ+AZM: 2(3.8)[c]<br>HCQ+LPVr: 1(4.5)[c]<br>HCQ+AZM+LPVr: 1(14.2)[c]<br>Vomiting:<br>HCQ: 1(5)[c]<br>HCQ+AZM: 1(1.9)[c]<br>HCQ+LPVr: 1(4.5)[c]<br>HCQ+AZM+LPVr: 0(0)[c]<br>Other gastrointestinal disorders:<br>HCQ: 0(0)[c]<br>HCQ+AZM: 2(3.8)[c]<br>HCQ+LPVr: 1(4.5)[c]<br>HCQ+AZM+LPVr: 0(0)[c]<br>Acute renal failure:<br>HCQ: 0(0)[c]<br>HCQ+AZM: 2(3.8)[c]<br>HCQ+LPVr: 6(27.3)[c]<br>HCQ+AZM+LPVr: 0(0)[c]<br>QT interval prolongation:<br>HCQ: 2(10)[c]<br>HCQ+AZM: 3(5.7)[c]<br>HCQ+LPVr: 0(0)[c]<br>HCQ+AZM+LPVr: 1(14.2)[c]	

								Atrial fibrillation:<br>HCQ: 0(0)[c]<br>HCQ+AZM: 1(1.9)[c]<br>HCQ+LPVr: 0(0)[c]<br>HCQ+AZM+LPVr: 1(14.2)[c]<br>Thrombocytopenia:<br>HCQ: 0(0)[c]<br>HCQ+AZM: 0(0)[c]<br>HCQ+LPVr: 1(4.5)[c]<br>HCQ+AZM+LPVr: 0(0)[c]<br>Lymphopenia:<br>HCQ: 0(0)[c]<br>HCQ+AZM: 0(0)[c]<br>HCQ+LPVr: 1(4.5)[c]<br>HCQ+AZM+LPVr: 0(0)[c]<br>Skin and subcutaneous disorders:<br>HCQ: 2(10)[c]<br>HCQ+AZM: 1(1.9)[c]<br>HCQ+LPVr: 0(0)[c]<br>HCQ+AZM+LPVr: 0(0)[c]<br>Ocular disorders:<br>HCQ: 0(0)[c]<br>HCQ+AZM: 1(1.9)[c]<br>HCQ+LPVr: 1(4.5)[c]<br>HCQ+AZM+LPVr: 0(0)[c]<br>Nervous system disorders:<br>HCQ: 0(0)[c]<br>HCQ+AZM: 0(0)[c]<br>HCQ+LPVr: 0(0)[c]<br>HCQ+AZM+LPVr: 0(0)[c]	

Hydroxychloroquine/Azithromycin Therapy and QT prolongation in hospitalized patients with COVID-19. O'Connell TF. JACC Clin Electrophysiol. 2021	HCQ + AZM	415	Characterized QTc prolongation in a cohort of hospitalized patients with COVID-19	Age (years):<br>65 (15)[b]<br><br>Gender (FEmale):<br>178(43)[c]%	Inclusion criteria:<br>-Adult patient (≥18 years)<br>-COVID-19 admitted to Beaumont Hospital<br>-At least one day of corrected QT interval evaluation after initiation<br>-Interpretable baseline ECG	(a) Observational<br>(b) Longitudinal<br>(c) Retrospective<br>(d) Treatment duration<br>(e) 13th March to 6th April 2020	HCQ 0.4 g BD for 2 doses followed by 0.2g BD for 4 days<br>AZM 0.5 g once followed by 0.25g QD for 4 days	a)<br>QTc ≥500ms:<br>87(21)[c]<br>High-grade ventricular Arrhythmia:<br>0(0)[c]<br>Ventricular tachycardia:<br>0(0)[c]<br>Ventricular fibrillation >30 s:<br>0(0)[c]<br>b) ECG, telemetry monitoring	94.6 (Strobe)
Hydroxychloroquine with azithromycin in patients hospitalized for mild and moderate COVID-19. Lamback EB. Braz J Infect Dis. 2021	HCQ + AZM	193<br><br>HCQ+AZM: 101<br><br>Control group: 92	Efficacy of HCQ in combination with AZM and describe the occurrence of adverse events	Age (years):<br><br>68 (47-72)<br><br>Gender (male): 117 (61)[c]	Inclusion criteria:<br>-Adult patients (≥18 years) hospitalized for mild and moderate COVID-19 related ARSD<br><br>Exclusion criteria:<br>-Initially admitted to the ICU<br>-No confirmation by RT-PCR for SARS-CoV-2 infection	(a) Observational<br>(b) Longitudinal<br>(c) Retrospective<br>(d)<br>(e) March to June 2020	HCQ 0.4 g BD for 2 doses followed by 0.2g BD for 4 days<br>AZM 0.5 g QD for 5 days	a)<br>Gastrointestinal symptoms:<br>8(7.9)[c]<br>QTc prolongation:<br>8(7.9)[c]<br>Arrhythmia:<br>0(0)[c]	83.9 (Strobe)

A Randomized Trial of Hydroxychloroquine as Postexposure Prophylaxis for Covid-19. Boulware DR. N Engl J Med. 2020	HCQ	821 asymptomatic participants<br><br>HCQ: 414<br><br>Placebo: 407	To evaluate postexposure prophylaxis with hydroxychloroquine after exposure to Covid-19	Age (years):<br>40 (33-50)[e]<br><br>Gender (male):<br>397.36 (48.4%)[c]	Inclusion criteria:<br>Participants who had household or occupational exposure to a person with confirmed Covid-19 at a distance of less than 6 ft for more than 10 minutes while wearing neither a face mask nor an eye shield (high-risk exposure) or while wearing a face mask but no eye shield (moderate-risk exposure).<br><br>Exclusion criteria:<br>- Younger than 18 years of age<br>- Hospitalized patients<br>-Persons with symptoms of Covid-19 or PCR-proven SARS-CoV-2	(a) Randomized, double-blind, placebo-controlled<br>(b) Longitudinal<br>(c) Prospective<br>(d) Trial enrolment began on March 17, 2020, with an eligibility threshold to enrol within 3 days after exposure<br>(e) The objective was to intervene before the median incubation period of 5 to 6 days.	Treatment group:<br>HCQ 800 mg once, followed by 600 mg in 6 to 8 hours, then 600 mg daily for 4 additional days.<br>If participants had gastrointestinal upset, they were advised to divide the daily dose into two or three doses<br><br>Control group:<br>Placebo folate tablets, which were similar in appearance to the HCQ tablets, were prescribed as an identical regimen for the control group	Among the participants who took any HCQ, 40.1% (140 of 349) reported a side effect by day 5, as compared with 16.8% (59 of 351) receiving placebo (P<0.001).<br><br>(a)<br>Any:<br>140 (40.1)[c]<br>Nausea:<br>80 (22.9)[c]<br>Diarrhoea, abdominal discomfort, or vomiting:<br>81 (23.2)[c]<br>Neurologic reaction: irritability, dizziness, or vertigo:<br>19 (5.4) [c]<br>Headache:<br>13 (3.7)[c]<br>Tinnitus:<br>8 (2.3)[c]<br>Visual changes:<br>3 (0.9)[c]<br>Skin reaction:<br>4 (1.1)[c]<br>Allergic reaction:<br>1(0.3)[c]<br>Fatigue:<br>1(0.3)[c]<br>Taste change or dry mouth:<br>3 (0.9)[c]<br>Hot flashes, night sweats, or palpitations:<br>0<br>* Values are through day 5, the date of the scheduled completion of the trial intervention.	5 (Jadad)

QT prolongation in a diverse, urban population of COVID-19 patients treated with HCQ, CQ, or AZ. Hsia BC. J Interv Card Electrophysiol. 2020	HCQ, CQ, or AZM	105	Risk of QT prolongation and subsequent outcomes after administration of these medications in largely underrepresented minority COVID-19 patients	Age (years):<br>67(52-82)[d]<br><br>Gender (female):<br>42.6 (44.8%)[c]	Inclusion criteria:<br>- Adults 18 years or older who met the following criteria: (I) received a positive SARS-CoV-2 test result confirmed by RT-PCR; (II) hospitalized within the Montefiore Health System; (III) received chloroquine (250 mg or 500 mg), hydroxychloroquine (400 mg), and/or AZM (250 mg or 500 mg) for treatment of COVID-19 infection; (IV) had a standard 12-lead, 10 s electrocardiogram (ECG) before and after COVID-19 treatment initiation.<br><br>Exclusion criteria:<br>- Patients that were under the age of 18<br>- Patients that had only a single ECG or did not have an ECG after medication initiation, or did not receive the medications of interest.	(a) Observational<br>(b) Longitudinal<br>(c) Retrospective<br>(d) The median time from the first dose of any treatment to post-medication ECG was 2 days<br>(e) Between February 13, 2020, and April 5, 2020	HCQ: 400 mg twice on the 1st day, followed by once per day for 4 days afterward or as determined by the physician.<br><br>CQ: 250 mg or 500 mg.<br><br>AZM: 500 mg on the 1st day, followed by 250 mg once per day for 4 days afterward.	(a)<br>- QTc in men increased from baseline (440 vs 455 ms, $p < 0.001$), as well as in women (438 vs 463 ms, $p < 0.001$).<br>- The proportion of patients with QT prolongation increased significantly (14.3% vs 34.3%, $p < 0.001$) even when adjusted for electrolyte abnormalities.<br>- The number of patients whose QTc > 500 ms was significantly increased after treatment (16.2% vs. 4.8%, $p < 0.01$).<br>- Patients with either QTc > 500 ms or an increase of 60 ms had a higher frequency of death (47.6% vs. 22.6%, $p = 0.02$) with an odds ratio of 3.1 (95% CI: 1.1–8.7).	81 (Strobe)

Hydroxychloroquine in Nonhospitalized Adults With Early COVID-19: A Randomized Trial. Skipper CP. Ann Intern Med. 2020	HCQ	423<br><br>HCQ: 212<br><br>Placebo: 211	To investigate whether HCQ could reduce COVID-19m severity in adult outpatients. The primary end point was change in overall symptom severity over 14 days	Age (years):<br>40 (32–50)[e]<br><br>Gender (female):<br>238 (56)[c]	Inclusion criteria:<br>Nonhospitalized adults who were required to have 4 or fewer days of symptoms and either PCR-confirmed SARS-CoV-2 infection or compatible symptoms after a high-risk exposure to a person with PCR-confirmed COVID-19 within the past 14 days.<br><br>Exclusion criteria:<br>- Younger than 18 years,<br>- Hospitalized patients	(a) Randomized, double-blind, placebo-controlled<br>(b) Longitudinal<br>(c) Prospective<br>(d) If participants were hospitalized within 14 days, we continued follow-up past study completion to assess outcomes.<br>(e) From 22 March through 20 May 2020.<br>Final hospital outcomes were known by 15 June 2020.	HCQ (800 mg once, followed by 600 mg in 6 to 8 h, then 600 mg daily for 4 more days) or masked placebo	Medication adverse effects occurred in 43% (92 of 212) of participants receiving HCQ versus 22% (46 of 211) receiving placebo (P < 0.001). Adverse effect prevalence decreased markedly after day 5<br><br>(a)<br>Upset stomach / nausea:<br>66(31.1)[c]<br>Diarrhea, other GI symptoms, vomiting:<br>50(23.6)[c]<br>Nervousness, irritability, dizziness, or vertigo<br>20(9.4)[c]<br>Skin reaction, rash:<br>6(2.8)[c]<br>Ringing in ears:<br>8(3.8)[c]<br>Allergic reaction, self-reported:<br>5(2.4)[c]<br>Changes in vision:<br>4(1.9)[c]<br>Warmth, hot flashes, night sweats:<br>2 (0.9)[c]	4 (Jadad)

								Headache:<br>2 (0.9)[c]<br>Taste, dry mouth:<br>0%<br>Heart racing, anxiety, panic attack:<br>0%<br>Cardiac Arrhythmia:<br>0%	
Effects on QT interval of hydroxychloroquine associated with ritonavir/darunavir or azithromycin in patients with SARS-CoV-2 infection. Moschini L. Heart Vessels. 2021	HCQ + AZM<br>or<br>HCQ + DRVr	113<br><br>HCQ/DRVr: 61<br><br>HCQ/AZM: 52	To evaluate the effects of an association therapy of HCQ plus DRVr or AZM Mon QTc intervals	Age (years):<br>68 (61–74)[e]<br><br>Gender (male): 85 (75)[c]	Inclusion criteria:<br>- Confirmed clinical and radiological diagnosis of SARS-CoV-2 pneumonia<br>- Electrocardiogram (ECG) recordings at baseline and then three and seven days after starting therapy<br>-Full treatment with the prescribed drugs for 7 days, unless onset of malignant ventricular arrhythmias<br><br>Exclusion criteria:<br>- QTc > 500 ms on baseline ECG<br>- History of severe systolic dysfunction (left ventricular ejection fraction<35%)<br>- History of arrhythmias, bradycardia<50 bpm<br>- Use of concomitant drugs that could cause QTc prolongation<br>- Early interruption of the medical therapy due to side effects.	(a) Observational<br>(b) Longitudinal<br>(c) Prospective<br>(d) Standard 12-lead ECG was recorded at screening time before the beginning of the therapy (day 0) and after three (day 3) and 7 days (day 7)<br>(e) From 22 March through 20 May 2020	HCQ: 200 mg b.i.d<br><br>DRVr: 100/800 mg q.d<br><br>AZM: 500 mg q.d	(a)<br>Tc interval>500 ms:<br>15 (13)[c] patients on day 3 and in 23 (20)[c] on day 7.<br>QTc>40 ms:<br>18 (16)[c]<br>Increase in median QTc:<br>From 438 to 452 ms in HCQ/DRVr patients.<br>From 433 to 440 ms in HCQ/AZM patients (p=0.001 for both).<br>Malignant ventricular arrhythmia:<br>HCQ/DRVr: 1(1.6)[c]<br>HCQ/AZM: 1(1.9)[c]	73 (Strobe)

Clinical outcomes and adverse events in patients hospitalised with COVID-19, treated with off-label hydroxychloroquine and azithromycin. 29. Kelly M. Br J Clin Pharmacol. 2021	HCQ + AZM	134<br><br>HCQ+AZM: 82<br><br>No targeted therapy: 52	The primary end point was clinical improvement on day 7. Secondary outcomes included mortality at day 28, intensive care admission, requirement for mechanical ventilation and incidence of adverse event	<u>Treatment group (82)</u><br><br><u>Age (years):</u> 64.8 (29–93)[e]<br><br><u>Gender</u> (female): 27 (33)[c]<br><br>No targeted therapy<u>(52)</u><br><br><u>Age (years):</u> 68 (21–91)[e]<br><br><u>Gender (female):</u>24 (46)[c]	<u>Inclusion criteria:</u><br>- Patients who had a positive polymerase chain reaction test for SARS-CoV-2<br><br><u>Exclusion criteria:</u> NA	(a) Observational<br>(b) Longitudinal<br>(c) Retrospective<br>(d) Patients who had a positive polymerase chain reaction test for SARS-CoV-2 and received HCQ plus AZM over a 2-week period from 20 March to 3 April 2020; compared to a group of SARS-CoV-2-positive patients who did not receive targeted therapy between 1 March and 9 May 2020.<br>(e) Between 1 March and 9 May 2020	HCQ 400 mg twice daily on day 1 followed by 200 mg twice daily on day 2–5 (if creatinine clearance was >30 mL/min) + AZM 500 mg on day 1 followed by 250–500 mg on days 2 and 3	The overall incidence of adverse events was 35 (42)[c] in the treatment group 22 (42)[c] (22/52) in the nontreatment group.<br><br>(a)<br><u>Elevated liver function tests</u>:<br>- 53(65)[c] in treatment group<br>- 27(52)[c] in placebo group<br><br><u>Development of prolonged QTc:</u><br>-11(13.4)[c] patients in the treatment group<br>-1 (1.2)[c] patients in placebo<br>This resulted in the discontinuation of therapy<br>in 4 cases.	68 (Strobe)

Safety, tolerability, and clinical outcomes of hydroxychloroquine for hospitalized patients with coronavirus 2019 disease. Satlin MJ. PLoS One. 2020 Jul	HCQ	153	Primary clinical outcome: improvement in hypoxia between the day of HCQ initiation and 10 days after HCQ initiation. Secondary outcomes: need for invasive mechanical ventilation within 10 days after HCQ initiation among patients who were not receiving mechanical ventilation at the start of HCQ therapy	Age (years): 62 (42–74)[e]<br><br>Gender (female): 56 (37)[c]	Inclusion criteria:<br>- Patients with COVID-19<br>- QT (QTc) interval of <500 msec<br><br>Exclusion criteria:<br>- Patients who had mild illness and short inpatient admissions	(a) Observational<br>(b) Longitudinal<br>(c) Retrospective<br>(d) Hospitalized patients with COVID-19<br>(e) Between March 05 2020 and March 25 2020	HCQ: 600 mg of HCQ every 12 hours for two doses, followed by 400 mg daily for four additional days<br><br>(patients with COVID-19 who received ≥1 dose of HCQ)	Forty-seven (40%) of the 117 patients who had a baseline EKG had a follow-up EKG between 1–10 days after HCQ initiation.<br><br>(a)<br>QTc increase of >30 msec: 55(36)[c]<br>Incident arrhythmia: 13(9)[c]<br>Monomorphic ventricular tachycardia: 1(0.6)[c]<br>Supraventricular tachyarrhythmias: 15(10)[c]<br>TdP: 0%<br>Grade 3 anemia: 2 3(15)[c]<br>Grade 3 lymphopenia: 15(10)[c]<br>Grade 4 incident blood count abnormality: 3(2)[c]<br>Grade 3 or 4 AST and ALT increases occurred in 17(11)[c] and 14(9)[c] of patients, respectively.	87 (Strobe)
Hydroxychloroquine use in hospitalised patients with COVID-19: An observational matched cohort study. Kalligeros M. J Glob Antimicrob Resist. 2020	HCQ ± AZM	108<br><br>HCQ+AZM: 32<br><br>HCQ monotherapy:4 (due to prolonged QTc interval at baseline)<br><br>Supportive care: 72	The primary end point was to assess the impact of hydroxychloroquine with or without AZM, on outcome, length of hospitalization, and time to clinical improvement	Age (years): 58.5 (45–69.5)[e]<br><br>Gender (male): 57 (52.8)[c]	Inclusion criteria: adult patients with COVID-19<br><br>Exclusion criteria: NA	(a) Observational<br>(b) Longitudinal<br>(c) Prospective<br>(d) NA<br>(e) NA	5-day regimen	(a)<br><br>QTc prolongation (484 ms): 1(3.1)[c]<br>QTc > 500 ms: 3(9.4)[c]<br>TdP: 0%<br>Bradycardia: 1(3.1)[c]<br>Altered mental status: 1(3.1)[c]<br>Seizure: 1(3.1)[c]<br>Ventricular contractions: 1(0.9)[c]	56 (Strobe)

Hydroxychloroquine and azithromycin tolerance in haemodialysis patients during COVID-19 infection. Giaime P. Nephrol Dial Transplant. 2020	HCQ+AZM	21	To report tolerance of treatment with AZM and HCQ prescribed for COVID-19 treatment in haemodialysis patients.	Age (years):<br>68 (52.3-83.7)[e]<br><br>Gender (male): (57.1)[c]	Inclusion criteria: COVID-19 haemodialysis patients<br><br>Exclusion criteria: NA	(a) Observational<br>(b) Longitudinal<br>(c) Retrospective<br>(d) Hospitalized COVID-19 haemodialysis patients<br>(e) Between 17 March and 10 April 2020	HCQ: 200 mg thrice daily during 10 days<br><br>AZM: 500 mg on Day 1, and 250 mg on the four following days	(a)<br>Nausea or vomiting:<br>4 (19) [c]<br>Allergy or dermatitis:<br>0%<br>Visual impairment:<br>0%<br><br>Cardiac<br>Long QTc syndrome (>500 ms):<br>1 (4.8)[c]<br>Cardiac rhythm disturbances:<br>0%<br><br>Hypoglycaemia<br>Insulin treated:<br>3 (14.3)[c]<br>Oral antidiabetic:<br>1 (4.8)[c]<br>No treatment:<br>1 (4.8)[c]	82 (Strobe)
Azithromycin in addition to standard of care versus standard of care alone in the treatment of patients admitted to the hospital with severe COVID-19 in Brazil (COALITION II): a randomised clinical trial. Furtado RHM. Lancet. 2020	HCQ ± AZM	397<br><br>AZM+HCQ: 214<br><br>HCQ: 183 (control group)	To assess the effect of AZM added to a standard of care regimen that includes HCQ on a patient-centred outcome in patients with severe COVID-19	AZM (+HCQ)<br><br>Age (years): 59·4 (49·3–70·0) [e]<br><br>Gender (male): 140 (65) [c]<br><br>Control (HCQ)<br><br>Age (years): 60·2 (52·0–70·1)[e]<br><br>Gender (male): 122 (67) [c]	Inclusion criteria:<br>- Patients who were at least 18 years and were admitted to hospital with suspected or confirmedCOVID-19 with fewer than 14 days since symptom onset.<br><br>Patients admitted to hospital with suspected or confirmed COVID-19 and at least one additional severity criteria as follows:<br>- use of oxygen supplementation of more than 4 L/min flow<br>- use of high-flow nasal cannula<br>- use of non-invasive mechanical ventilation<br>- use of invasive mechanical ventilation.<br><br>Exclusion criteria:	(a) Randomized, open-label<br>(b) NA<br>(c) NA<br>(d) 15-day clinical status and 29-day survival of patients hospitalised with severe COVID-19<br>(e) From March 28 to May 19, 2020	AZM: 500 mg via oral, nasogastric, or intravenous administration once daily for 10 days plus standard of care or to standard of care without macrolides.<br><br>HCQ: 400 mg twice daily for 10 days)	(a)<br>Serious adverse events suspected to be related to study drug<br>AZM+ HCQ: 12 (5)[c]<br>HCQ: 8 (4)[c]<br>QTc interval prolongation<br>AZM+ HCQ: 47 (20)[c]<br>HCQ: 42 (21)[c]<br>Gastrointestinal intolerance<br>AZM+ HCQ: 61 (25)[c]<br>HCQ: 48 (24)[c]<br>Clinically relevant ventricular arrhythmias<br>AZM+ HCQ: 8 (3)[c]<br>HCQ: 5 (3)[c]<br>Resuscitated cardiac arrest<br>AZM+ HCQ: 16 (7)[c]<br>HCQ: 13 (7)[c]<br>Death due to ventricular arrhythmia<br>AZM+ HCQ: 0%<br>HCQ: 0 %	76 (Strobe)

					- Use of hydroxychloroquine, chloroquine, or macrolides for more than 48 h before enrolment and since symptom onset<br>- History of severe ventricular cardiac arrhythmia or electrocardiogram with QTc interval of 480 ms or longer; and known allergy to any of the trial drugs.			Acute kidney failure<br>AZM+ HCQ: 147 (61)[c]<br>HCQ: 103 (52)[c]<br><br>Need for dialysis (in patients not on dialysis at baseline)<br>AZM+ HCQ: 86/222 (39)[c]<br>HCQ: 64/189 (34)[c]<br><br>Death due to acute kidney failure<br>AZM+ HCQ: 2 (1)[c]<br>HCQ: 3 (2)[c]<br><br>Decrease in white blood cell count of >50% on at least one occasion<br>AZM+ HCQ: 10 (4)[c]<br>HCQ: 4 (2)[c]<br><br>Decrease in lymphocytes >50%on at least one occasion<br>AZM+ HCQ: 27 (11)[c]<br>HCQ: 21 (11)[c]<br><br>Decrease in platelets >50%on at least one occasion<br>AZM+HCQ: 10 (4)[c]<br>HCQ: 8 (4)[c]<br><br>Increase in bilirubin >50% on at least one occasion<br>AZM+ HCQ: 10 (4)[c]<br>HCQ: 6 (3)[c]	

Assessing QT interval in COVID-19 patients:safety of hydroxychloroquine-azithromycin combination regimen. Bernardini A. Int J Cardiol. 2021	HCQ or HCQ+AZM or no treatment	112<br><br>Divided in 3 groups:<br><br>Group 1; no treatment: 19 (17)[c]<br><br>Group 2; HCQ: 40 (36)[c]<br><br>Group 3; HCQ+AZM: 53 (47)[c]	The effects of the use of HCQ alone or in combination with AZM in a real world COVID-19 population admitted to a tertiary Hospital in Lombardy	Age (years): 66.9 (54.2-79.6)[e]<br><br>Gender (male): (79 (71)[c]	Inclusion criteria: COVID-19 patients<br><br>Exclusion criteria: History of long QT syndrome neither long QT in ECG recorded prior to the hospital admission.	(a) Observational<br>(b) Longitudinal<br>(c) Retrospective<br>(d) Average follow-up period of 13.6 ± 7.4 days<br>(e) All consecutive patients admitted from the Emergency Department with confirmed COVID-19 diagnosis from March 28th	HCQ: 400 mg twice for the first day, then 200 mg twice.<br><br>AZM: 500 mg daily on the first day followed by 250 mg daily, thereafter	(a)<br>Atrial fibrillation:<br>HCQ: 1(3)[c]<br>HCQ+AZM: 6(11)[c]<br><br>QTc prolonged:<br>HCQ: 16 (40)[c]<br>HCQ+AZM: 37(70)[c]<br><br>QTc>500ms:<br>HCQ: 0%<br><br>HCQ+AZM: 4 (8)[c]	81 (Strobe)
Efficacy and Safety of Hydroxychloroquine vs Placebo for Pre-exposure SARS-CoV-2 Prophylaxis Among Health Care Workers: A Randomized Clinical Trial. Abella BS. JAMA Intern Med. 2021	HCQ	132	Primary outcome: incidence of SARS-CoV-2 infection as determined by a nasopharyngeal swab during the 8 weeks of treatment. Secondary outcomes: adverse effects, treatment discontinuation, presence of SARS-CoV-2 antibodies, frequency of QTc prolongation, and clinical outcomes for	Age (years): 33 (20-66)[e]<br><br>Gender (female): 91 (69)[c]	Inclusion criteria:<br>Health care workers that:<br>- worked 20 h or more per week in hospital-based units<br>- had no known history of SARS-CoV-2 infection<br>- did not have symptoms suggestive of COVID-19 in the 2 weeks before enrollment, including cough, fever, or shortness of breath<br><br>Exclusion criteria:<br>- History of a positive SARS-CoV-2 test<br>- Allergy or sensitivity to HCQ<br>- Glucose-6-phosphate dehydrogenase deficiency<br>- Retinal diseases<br>- Substantial cardiac disease	(a) Randomized, double-blind, placebo-controlled<br>(b) Longitudinal<br>(c) Prospective<br>(d) At the time of randomization (baseline), 4 weeks, and 8 weeks, participants underwent study-specific NP swab testing for SARS-CoV-2<br>(e) From April 9, 2020, to July 14, 2020; follow-up ended August 4, 2020	HCQ: 600 mg, daily, or size-matched placebo taken orally for 8 weeks or placebo	Mild adverse events were more common in participants taking HCQ compared with placebo (45% vs 26%;)<br><br>(a)<br>Adverse effects of any grade in HCQ group:<br><br>Abdominal pain:<br>4(6)[c]<br>Anorexia:<br>7(11)[c]<br>Chest pain:<br>1(2)[c]<br>Constipation:<br>0<br>Diarrhea:<br>21(32)[c]<br>Dizziness:<br>1(2)[c]<br>Fatigue:<br>2(3)[c]<br>Gastroesophageal reflux:<br>2 (3)[c]<br>Headache:	3 (Jadad)

			SARS-CoV-2–positive participants					0<br>Nausea:<br>6(9)[c]<br>Paresthesia:<br>1(2)[c]<br>Rash:<br>3(5)[c]<br>Throat tightness:<br>0	
Safely administering potential QTc prolonging therapy across a large health care system in the COVID-19 era. Saleh M. Circ Arrhythm Electrophysiol. 2020	HCQ ± AZM<br><br>Over the course of the study period AZM was dropped from the regimen due to perceived lack of efficacy	6476<br><br>HCQ+AZM:3.629<br><br>HCQ: 2847	Primary clinical outcome: QT prolongation resulting in TdP. Secondary outcomes: QT prolongation resulting in premature discontinuation of HCQ and AZM, incidence of ventricular arrhythmias, and arrhythmogenic death	Age (years):<br>63.9 (48.7-79.1)[e]<br><br>Gender (male): 3.980 (61.5)[c]	Inclusion criteria: All patients >18 years of age with a polymerase chain reaction test confirmed diagnosis of COVID19 illness admitted across 13 hospitals within the Northwell Health system.<br><br>Exclusion criteria: Patients with COVID-19 positive who were chronically on HCQ for autoimmune diseases such as lupus	(a) Observational<br>(b) Longitudinal<br>(c) Prospective<br>(d) All ECGs performed were tabulated for the 10 days before April 5, 2020, and the 10 days after as a marker of HCP exposure and PPE usage<br>(e) Between March 1 and April 15 w	HCQ: either 400 mg by mouth twice daily or 800 mg by mouth once daily for 1 day followed by 200 mg by mouth twice daily or 400 mg by mouth once daily for 4 days.<br><br>AZM: 500 mg by mouth or intravenous daily for up to 5 days.	(a) 29 patients (0.45%) who were found to have ventricular arrhythmias while receiving therapy:<br><br>TdP:<br>1 (0.015)[c]<br>Ventricular fibrillation:<br>4 (0.06)[c]<br>Sustained polymorphic ventricular tachycardia:<br>1 (0.015)[c]<br>Nonsustained polymorphic ventricular tachycardia:<br>0<br>Sustained monomorphic ventricular tachycardia:<br>5 (0.08)[c]<br>Nonsustained monomorphic ventricular tachycardia:<br>18 (0.27)[c]	68 (Strobe)

ADE: adverse drugs events; AEs: adverse events; ALT: alanine aminotransferase; ARDS: acute respiratory distress syndrome; AST: aspartate aminotransferase; AVB: atrioventricular block; AZM: azithromycin; BCT: baricitinib; BD: twice a day; bpm: beats per minute; CCU: critical care unit; CPR: cardiopulmonary resuscitation; CT: computed tomography; CQ: chloroquine; COVID-19: coronavirus disease 2019; DRVr: darunavir/ritonavir; ECG: electrocardiograph; e.g.: for example; eGFR :estimated glomerular filtration rate; FVP: favipiravir; g: grams; G6PD: glucose-6-phospate dehydrogenase; GGT: gamma-glutamyl transferase; h: hours; ICU: intensive care unit; INF-alpha: interferon alpha; INF-β: interferon beta; IV: intravenous; IVIG: intravenous immunoglobulin; HCQ: hydroxychloroquine; LEV: levofloxacin; LPVr: lopinavir/ritonavir; LWH: low weight heparin; mEq/l: milliequivalents per litre; mg: milligrams; mg/dl: milligrams per decilitre; ml/min/1.73m^2: millilitre per minute per 1.73 square meter; mmHg: millimetre of mercury; MPDN: metilprednisolone; ms:milliseconds; NA: not available/not applicable; OSMV: Oseltamivir; PaO2/FIO2: ratio of arterial oxygen partial pressure to fractional inspired oxygen; PT: posttreatment; REM: remdesivir; RT-PCR: reverse transcription polymerase chain reaction; QD: once a day; QTc: corrected QT interval; QTD: QTc dispersion; SARS-CoV-2: Severe acute respiratory syndrome coronavirus 2; SaO2/SPO2: ratio of arterial oxygen saturation to peripheral capillary oxygen saturation; SIRS: systemic inflammatory response syndrome; SOC: standard of care; TdP: Torsade de Pointes; TD: three times a day; TDS: three times per day; TCZ: tocilizumab; Tp-e: T wave peak-to-end intervalWHO: World Health Organization; JTc: corrected JT interval; μg: microgram; VT: ventricular tachycardia; w: week.

[a]Jadad, AR et al. Assessing the quality of reports of randomized clinical trials: is blinding necessary? Control Clin Trials. 1996; 17(1):1–12. Strobe. Quality assessment, a higher score indicates a higher quality: [Yes (1) + Partly (0.5)/Total applicable] x100.

Values are: [b]mean (SD); [c]n (%); [d]mean (range); [e] median (interquartile range); [f]median (range); [g]median (IQR), rang[e]
